# Design and synthesis of thiazolidine-2,4-diones hybrids with 1,2-dihydroquinolones and 2-oxindoles as potential VEGFR-2 inhibitors: *in-vitro* anticancer evaluation and *in-silico* studies

**DOI:** 10.1080/14756366.2022.2085693

**Published:** 2022-07-08

**Authors:** Mohammed S. Taghour, Hazem Elkady, Wagdy M. Eldehna, Nehal M. El-Deeb, Ahmed M. Kenawy, Eslam B. Elkaeed, Aisha A. Alsfouk, Mohamed S. Alesawy, Ahmed M. Metwaly, Ibrahim. H. Eissa

**Affiliations:** a Pharmaceutical Medicinal Chemistry & Drug Design Department, Faculty of Pharmacy (Boys), Al-Azhar University, Cairo, Egypt; b Department of Pharmaceutical Chemistry, Faculty of Pharmacy, Kafrelsheikh University, Kafrelsheikh, Egypt; c School of Biotechnology, Badr University in Cairo, Badr City, Cairo, Egypt; d Biopharmaceutical Products Research Department, Genetic Engineering and Biotechnology Research Institute, City of Scientific Research and Technological Applications (SRTA-City), Alexandria, Egypt; e Nucleic Acids Research Department, Genetic Engineering and Biotechnology Research Institute, City of Scientific Research and Technological Applications (SRTA-City), Alexandria, Egypt; f Department of Pharmaceutical Sciences, College of Pharmacy, AlMaarefa University, Riyadh, Saudi Arabia; g Department of Pharmaceutical Sciences, College of Pharmacy, Princess Nourah bint Abdulrahman University, Riyadh, Saudi Arabia; h Pharmacognosy and Medicinal Plants Department, Faculty of Pharmacy (Boys), Al-Azhar University, Cairo, Egypt

**Keywords:** Apoptosis, anticancer, VEGFR-2 inhibitors, 2-Oxo-1,2-dihydroquinoline, Thiazolidine-2,4-dione, 2-Oxoindoline

## Abstract

A thiazolidine-2,4-dione nucleus was molecularly hybridised with the effective antitumor moieties; 2-oxo-1,2-dihydroquinoline and 2-oxoindoline to obtain new hybrids with potential activity against VEGFR-2. The cytotoxic effects of the synthesised derivatives against Caco-2, HepG-2, and MDA-MB-231 cell lines were investigated. Compound **12a** was found to be the most potent candidate against the investigated cell lines with IC_50_ values of 2, 10, and 40 µM, respectively. Furthermore, the synthesised derivatives were tested *in vitro* for their VEGFR-2 inhibitory activity showing strong inhibition. Moreover, an *in vitro* viability study against Vero non-cancerous cell line was investigated and the results reflected a high safety profile of all tested compounds. Compound **12a** was further investigated for its apoptotic behaviour by assessing the gene expression of four genes (Bcl2, Bcl-xl, TGF, and Survivin). Molecular dynamic simulations authenticated the high affinity, accurate binding, and perfect dynamics of compound **12a** against VEGFR-2.

## 1. Introduction

Cancer is the second health burden around the world after cardiovascular diseases, in accordance with estimates of the world health organisation (WHO)^1^. It is forecasted to become the major cause of death in the impending years^2^. Cancer therapy is a challenging area for medicinal chemists, in which they need to discover safe and effective targeted chemotherapeutic agents^3^. Such agents are designed for inhibiting cancer cell growth by interacting with specific molecular targets resulting in significant damage to the cancerous cells^4^.

Among the most efficient targets in cancer management, the vascular endothelial growth factor receptor-2 (VEGFR-2) is a vital transmembrane tyrosine kinase receptor^5^. VEGFR-2 orchestrates important steps in cell proliferation, division, motility, adhesion, and angiogenesis^6^. Accordingly, stopping the VEGFR-2 signalling cascade reduces the proliferation of different types of cancer cells^7^.

Over the past few decades, many anti-angiogenic drugs targeting VEGFR-2 were approved by FDA as anticancer drugs e.g. sunitinib, cabozantinib, and sorafenib^8^. VEGFR-2 has an advantage of the weak expression in the healthy tissue and overexpression is reported in several types of cancer namely, breast cancer, prostate cancer, colon cancer, cervical cancer, NSCLC, kidney clear cell cancer, brain glioma, bladder carcinoma, pancreatic cancer, oral cancer, skin melanoma oesophageal cancer, and ovarian cancer^9^^,^^10^. In addition, it was reported that the selective blockage of VEGFR-2 rather than of both receptors (VEGFR-1 and VEGFR-2), ideally overcomes the disadvantageous haematologic consequences that occurred in malignancy due to the elevated VEGF levels^11^.

**Figure 1.**
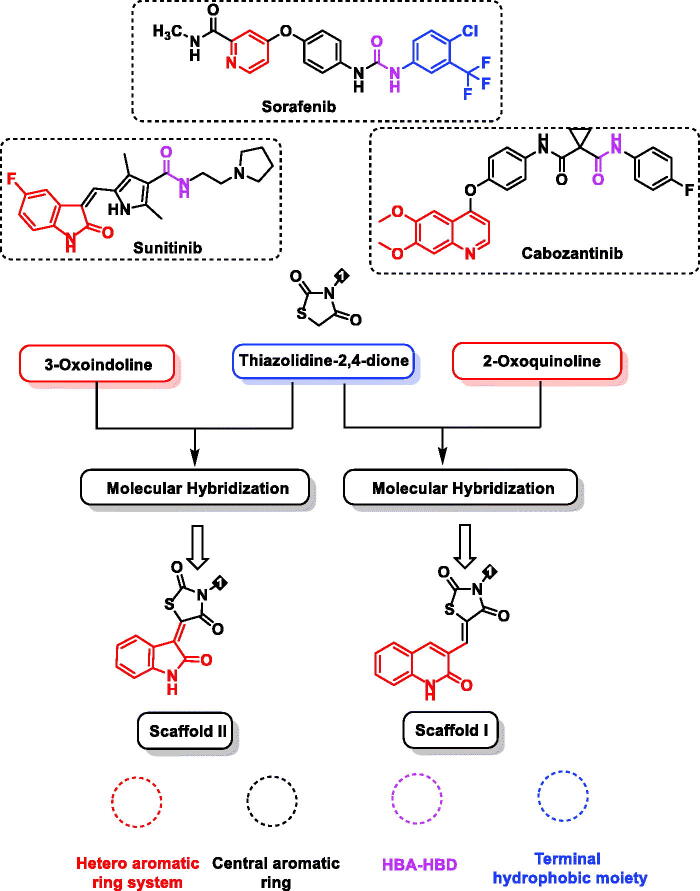
Design of target compounds based on FDA-approved VEGFR-2 inhibitors and molecular hybridisation strategy.

Studying the pharmacophoric features of these drugs showed four essential features that should be included. First, a heteroaromatic moiety binds to Cys919 in the hinge part of the ATP binding site of VEGFR-2. Secondly, a spacer group to occupy the gatekeeper region^12^. Third, a pharmacophore moiety that can be incorporated in hydrogen-bonds with Glu885 and Asp1046 in the DFG domain of the receptor^13^. Finally, a terminal hydrophobic moiety to be incorporated with the allosteric hydrophobic pocket of the ATP binding site by some hydrophobic interactions (Figure 1)^14–17^.

Indoline scaffold, a special class in the drug design and discovery^18–22^, was among the first kinase inhibitors that reached the clinic and their inhibition of VEGFR-2 has been extensively investigated^23^^,^^24^. Furthermore, different compounds carrying the quinoline skeleton were reported as tyrosine kinase inhibitors^25^. Among the quinoline derivatives, 2-oxoquinoline scaffolds attracted great attention because of their intersting pharmacological activities, such as antioxidant^26^, antimicrobial^27^, anti-inflammatory^28^, and virtuous anticancer effects^29^^,^^30^. Additionally, thiazolidine-2,4-dione moiety was a fascinating heterocyclic core in medicinal chemistry for the design and discovery of active compounds particularly anticancer agents^31–33^.

Keeping in mind the aforementioned findings and continuing our trip in the discovery of novel VEGFR-2 inhibitors^34–41^, we hybridised 2-oxoindoline and 2-oxoquinolin with thiazolidine-2,4-dione moiety to display kinase inhibitory and anti-proliferative activities introducing a new class of VEGFR-2 inhibitors (Figure 1).

### 1.1. Design concept

The basic idea of molecular design’s rationale is the modification of lead VEGFR-2 inhibitors keeping the same essential pharmacophoric features of sunitinib, cabozantinib, and sorafenib. Also, employing ligand-based drug design, in particular the molecular hybridisation strategy^42–46^, a molecular hybridisation of thiazolidine-2,4-dione with the effective antitumor moieties 2-oxoindoline and 2-oxoquinolin was conducted to get new hybrids of prospective VEGFR-2 inhibitory effects. The approach utilised for designing our target candidates is illustrated in Figure 1.

In detail, for the hinge regions, 2-oxo-1,2-dihydroquinolin (**scaffold I**) and 2-oxoindoline (**scaffold II**), were used as hetero-aromatic moieties. The bicyclic structures of such moieties are suitable for the big space of the ATP binding region^47^. The gatekeeper region was targeted to be occupied by thiazolidine-2,4-dione moiety as a spacer group. The many hydrogen bond acceptor atoms in thiazolidine-2,4-dione moiety may give a good chance for efficient binding to the targeted receptor. With regards to the DFG-motif region, we selected an amide group as apharmacophore moiety to be buried into it. Additionally, several aromatic derivatives were selected to occupy the allosteric hydrophobic region to investigate the structure-activity relationship (Figure 1).

This kind of work—pharmacophoric features based synthesis—was a goal for our team to achieve rather than the usual SAR-based synthesis. It enables getting promising results through the syntheses of fewer compounds. saves time, effort, and costs as well as protects the environment from the drawbacks of chemical synthesis and the use of excessive organic solvents.

All tested products were screened for their *in vitro* antiproliferative activity against three cancer cell lines and one non-cancerous cell line. Besides, the VEGFR-2 inhibitory potentialities of the tested compounds were assessed. In addition, deep biological testing was investigated for the most promising candidates to have a good insight into the mechanism of action against cancerous cell lines. Furthermore, an *in silico* docking, MD, MM-PBSA, ADMET, and toxicity studies were also performed to predict the binding mode of the synthesised compounds within the binding site of VEGFR-2 kinase as well as their drug-likeness degrees.

## 2. Results and discussion

### 2.1. Chemistry

**Scheme 1.**
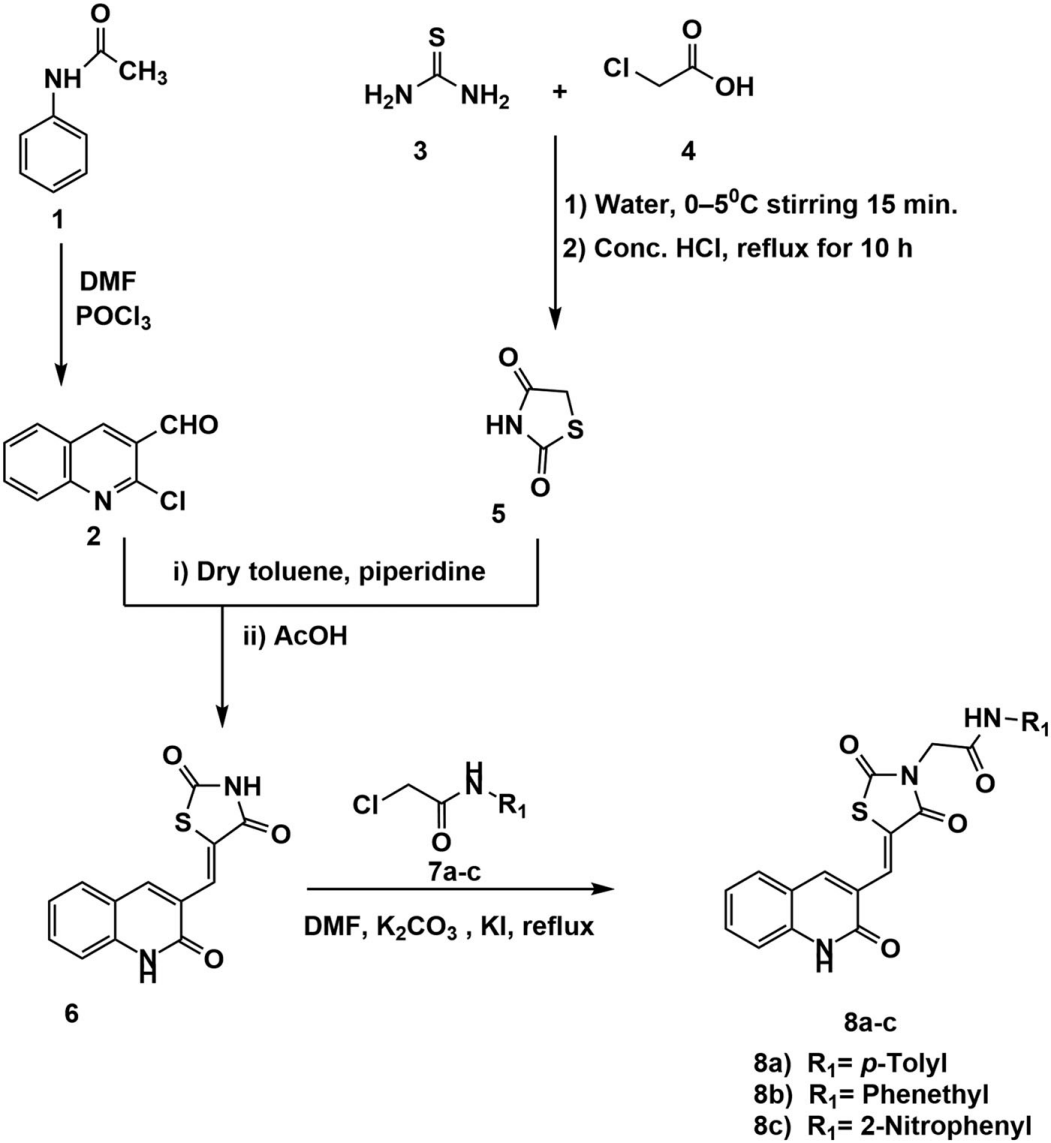
Synthesis of compounds **8a**–**c**.

**Scheme 2.**
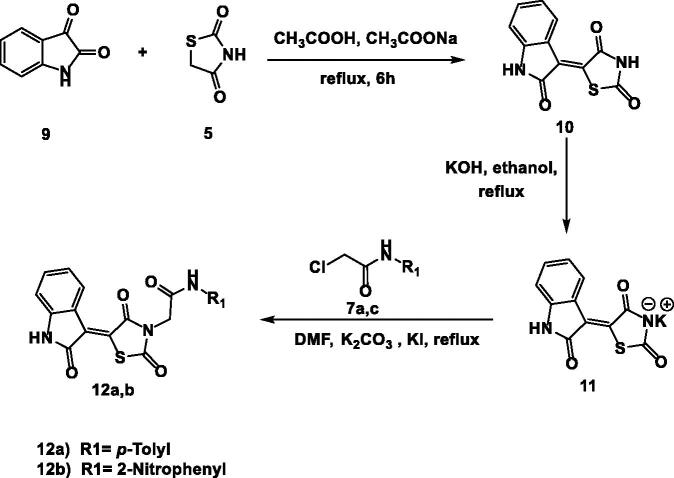
Chemical synthesis of compounds **12a** and **b.**

The sequence of chemical synthesis reactions that clarified in Schemes 1 and 2 was utilised to synthesise the hybrid compounds. Initially, 2-chloroquinoline-3-carbaldehyde **2** was obtained via the Vilsmeier–Haack–Arnold reaction, which included the condensation of *N*-phenylacetamide **1** with *N,N*-dimethylformamide (DMF) in the presence of phosphorus oxychloride^48^^,^^49^. On the other hand, thiazolidine-2,4-dione (TZD) **5** was obtained following the reported procedures^50^ through the reaction of thiourea **3** with chloroacetic acid **4** under reflux in conc. HCl. Compounds **6** was obtained from the condensation of 2-chloroquinoline-3-carbaldehyde **2** with thiazolidine-2,4-dione **5** in dry toluene in the presence of piperidine as a base. Subsequent heating of compound **6** with 2-chloro-*N*-substitutedacetamide derivatives (**7a**–**c**) in DMF in the presence of potassium carbonate produced the corresponding final compounds **8a**–**c**, respectively (Scheme 1).

The chemical structures of compounds **8a**–**c** were authincated by IR, ^1^H NMR, and ^13 ^C NMR spectroscopic techniques. IR spectra showed strong bands ranging from 3449 to 3142 cm^−1^ due to the presence of the NHs. Further, IR spectra displayed strong C = O signals ranging from 1748 to1672 cm^−1^. Moreover, ^1^H NMR spectral data displayed singlet signals around *δ* 12.18 and 10.25 ppm of the two amidic NHs. Following such results, the ^13^C NMR spectral data also confirmed the validity of the suggested chemical structures, where characteristic signals were appeared at their fingerprint region.

Condensation of isatin **9** with thiazolidine-2,4-dione (TZD) **5** was better with sodium acetate in acetic acid, to provide the isatin derivative **10** in a satisfactory yield^51^. Treatment of compounds **10** with the alcoholic solution of potassium hydroxide resulted in its potassium salt **11**. Then, heating a mixture of compound **11** with the 2-chloro-*N*-substituted acetamide derivatives (**7a**, **c**) in dry DMF, in the presence of KI, afforded the corresponding compounds **12a**, **b**, respectively (Scheme 2).

The generated spectral data confirmed the chemical structures of the synthesised derivatives. In detail, the ^1^H NMR spectra of compounds **12a** and **b** showed typical downfield singlet signals around *δ* 11.32 and 10.74 ppm, respectively, corresponding to NHs. Considering compound **12a** as an example, the IR spectrum exhibited stretching bands at 2992 and 2936 cm^−1^ due to the aliphatic CH bonds. Furthermore, the ^1^H NMR spectrum displayed an up-field singlet signal at *δ* 2.26 ppm of the aliphatic protons of the CH_3_. Likewise, the ^13^C NMR spectrum exhibited two peaks at *δ* 44.06 and 20.91 of the CH_2_ and CH_3,_ respectively.

### 2.2. Biological evaluation

#### 2.2.1. *In-vitro* anticancer effects

Target quinolines **8a**–**c,** indolines **12a** and **12b** were assessed for their potential anti-proliferative effects against a panel of three different cancer cell lines; colon (Caco-2), hepatocellular (HepG2), and breast (MDA-MB-231) cancer cell lines using standard MTT assay protocol^52–54^. The obtained results confirmed the anti-cancer effects of the tested compounds against the three types of cancerous cell lines, expressed as the median growth inhibitory concentration (IC_50_), and were presented in Table 1. The obtained results indicated that compound **12a** was the most potent candidate against Caco-2, HepG-2 and MDA-MB-231 cell lines exhibiting IC_50_ values of 2, 10, and 40 µM, respectively. This indicated that hybridisation of 2-oxoindolin with terminal *p*-tolyl moiety improved the *in vitro* anti-proliferative activities of the synthesised derivatives.

**Table 1.** *In vitro* anti-proliferative activities of **8a–c** and **12a,b** against Caco-2, HepG2, and MDA-MB-231 cell lines.

Compounds	Anti-proliferative activity (IC_50_ µM)^a^		
	Caco-2	HepG2	MDA-MB-231
**8a**	9 ± 0.001	86 ± 0.001	150 ± 0.01
**8b**	150 ± 0.013	60 ± 0.008	60 ± 0.003
**8c**	90 ± 0.004	156 ± 0.004	70 ± 0.009
**12a**	2 ± 0.005	10 ± 0.001	40 ± 0.002
**12b**	120 ± 0.001	49 ± 0.008	37 ± 0.002
Doxorubicin	3.46 ± 0.003	1.15 ± 0.02	0.98 ± 0.01

^a^The results were the mean of three replicates.

With respect to the 2-oxo-1,2-dihydroquinolin based compounds, compound **8a** incorporating *p-*tolyl moiety showed the best cytotoxic activity against Caco-2 cell line with IC_50_ value of 9 µM. Meanwhile, 2-oxo-1,2-dihydroquinolin-based derivatives, the phenethyl containing compound, **8b** was the most potent member against HepG-2 and MDA-MB-231 cells and displayed equipotent cytotoxic activities against the two cell lines (IC_50_ = 60 µM). The remaining substituents, on the other hand, showed moderate IC_50_ values against the tested cell lines.

#### 2.2.2. *In vitro* VEGFR-2 enzyme assay inhibition

To explore the molecular mechanism of the tested compounds as anticancer agents, the compounds were further evaluated to assess their enzymatic inhibitory potential against VEGFR-2. For the derivative of scaffold **I (**compounds **8a**–**c)**, compound **8a** had the highest activity with an IC_50_ value of 87.37 nM. Regarding scaffold II (compounds **12a, b**), compound **12b** exhibited the highest enzymatic inhibitory potential with an IC_50_ value of 84.05 nM. The reference drug, sorafenib, exhibited an IC_50_ value of 53.65 nM (Table 2).

**Table 2.** IC_50_ values of the tested compounds against VEGFR-2 and Vero cell line and their selectivity index (SI) against different cancer cell lines.

Compounds	VEGFR-2 IC_50_ (nM)	Cytotoxicity against Vero (IC_50_ µM)	Selectivity index (SI)		
			(Caco-2)^a^	(HepG2)^b^	(MDA-MB-231)^c^
**8a**	96.64	1590 ± 0.068	176.67	18.49	10.60
**8b**	87.37	890 ± 0.015	5.93	14.83	14.83
**8c**	317.7	390 ± 0.020	4.33	2.50	5.57
**12a**	116.3	730 ± 0.015	365	73.00	18.25
**12b**	84.05	480 ± 040	4	9.80	12.97
Sorafenib	53.65	–	–		

^a^SI = Cytotoxicity against Vero cells / Cytotoxicity against Caco-2 cell line.

^b^SI = Cytotoxicity against Vero cells / Cytotoxicity against HepG2 cell line.

^c^SI = Cytotoxicity against Vero cells / Cytotoxicity against MDA-MB-231 cell line.

*An in-vitro* viability test was used to investigate the safety patterns of the tested compounds at different concentrations (1.0 mM/mL to 50 µM/mL). The Vero cell line was used as a non-cancerous cell line model to investigate the safety of the synthesised compounds. Using MTT assay, the tested compounds recorded IC_50_ values ranging from 390 to 1590 µM. Such values were very high in comparison to the corresponding values on cancer cell lines, which reflect the high *in vitro* safety profile of the tested members towards non-cancerous cell lines (Table 2).

The selectivity index (SI) of a particular compound is the ratio of its toxic concentration against the effective concentration (SI = IC_50_ on non-cancer cell/IC_50_ on cancer cell)^55^. The ideal compound should have an SI value ≥10^56^. Relatively, low SI (<1) means that the tested compound is toxic and cannot be used as a safe drug. If the SI value is between 1 and 10, further evaluation using other biological systems is encouraged for confirming the findings^57^.

**Figure 2.**
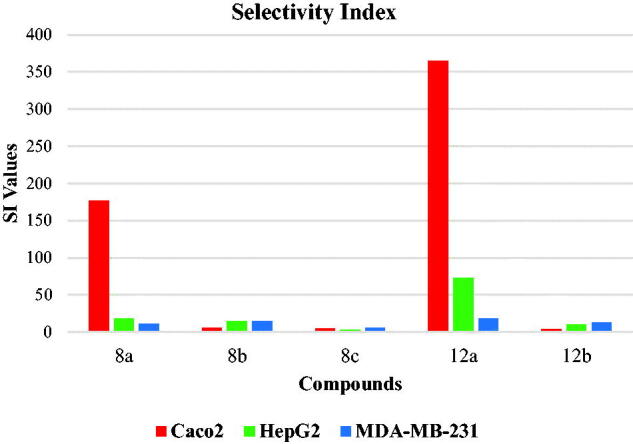
Selectivity indices of the synthesised compounds.

Fascinatingly, as aforementioned, all the tested hybrids showed decreased potency against Vero cell lines. This finding encouraged us to investigate the selectivity profile of the synthesised compounds. The selectivity index values of the synthesised compounds against cancer cells were indicated in Figure 2. In general, all compounds showed SI of more than 2.5 indicating high selectivity of the tested compounds against cancer cell lines. Interestingly, compound **12a** showed the highest SI values of 365, 73, and 18 against the Caco-2, HepG2, and MDA-MB-231 cell lines, respectively (Figure 2). Therefore, we selected compound **12a** for further biological testing.

#### 2.2.3. Effect of compound 12a on Caco-2 cells migration

**Figure 3.**
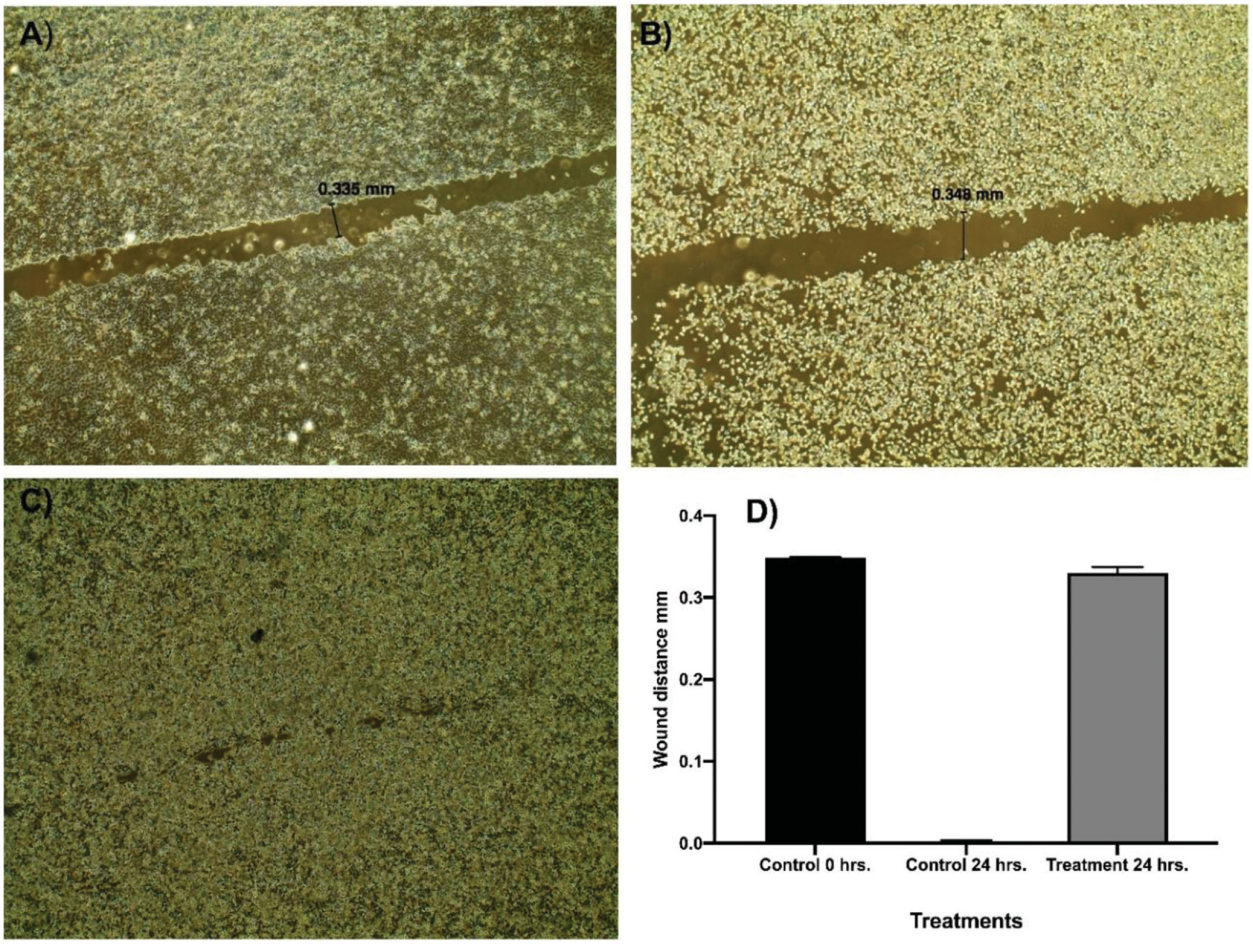
Effect of compound **12a** on cells migration and healingefficacy of Caco-2 cells.

The potential of compound **12a** to inhibit the ability of Caco-2 cells to migrate and heal was investigated through the *in-vitro* scratch assay which is a low-cost easy and well-developed method^58^. The basic concept of this method involves the generation of a scratch in a cancer cell line monolayer, recording the diameter at the beginning, and at regular intervals during cell migration to close that scratch. The obtained results of the treated cell line are then compared to the untreated cell line. Images of scratched areas from the time points; 0, 24 are illustrated in Figure 3.

Regarding the representative control, the scratch was completely closed within 24 h (Figure 3(C)). While the wound diameter of the treated Caco-2 cells with **12a** (sub IC_50_ value) slightly decreased from 0.348 (Figure 3(B)) to 0.335 mm (Figure 3(A)). These results confirmed that compound **12a** significantly inhibited the scratch closure and cancer cell migration at a low concentration of 1 µM. Interestingly, compound **12a** changed the Caco-2 cell shape from an irregular into the normalised round form within 24 h. This significant morphological change could be linked to the incidence of apoptosis in the treated cancer cells^59^.

#### 2.2.4. Alternation of cancer cells gene expression after Caco-2 treatment with 12 a using RT-qPCR

Apoptosis is a homeostatic mechanism that keeps the number of cells in an organism constant and eliminates unwanted cells which are damaged or unmanageable during different developmental stages. Different gene families such as the p53 gene, caspases, tumour necrosis factor (TNF) receptor gene superfamily, or B cell lymphoma (Bcl)-2 family of genes are involved and/or collaborate in the apoptosis process.

The balance between both the proapoptotic and anti-apoptotic molecules keeps stabilising the cellular homeostasis and defines cell destiny, either to apoptosis or growth and proliferation. Mitochondria have an essential role in releasing several vital apoptosis molecules such as SMAC, cytochrome c, apoptosis-inducing factor, and endonuclease G as a result of mitochondrial membrane permeabilization, which is activated by proapoptotic B cell lymphoma (Bcl)-2 family proteins. Meanwhile, the anti-apoptotic members of the Bcl-2 family (Bcl2 and Bcl-xl) maintains the integrity of the mitochondrial membrane

Overexpression of the Bcl2 gene can inhibit the apoptotic cell death and partially the nonapoptotic cell death, which has a role in arrest of cell cycle. Meanwhile, overexpression of Bcl-xL enhances autophagic cell death^60^. Moreover, Survivin is a pro-survival protein that is overexpressed in many cancer cells in the G2-M phase. This protein has been linked to tumour progression control and resistance to cancer chemotherapeutics. Furthermore, the transforming growth factor (TGF) is one of various proteins that secreted by transformed cells and stimulate the growth of non-cancerous cells in addition to its role as initiators of the signalling pathway that suppresses the early development of cancer cells^61^. Dysregulation of TGF-β activation and signalling may result in apoptosis.

**Figure 4.**
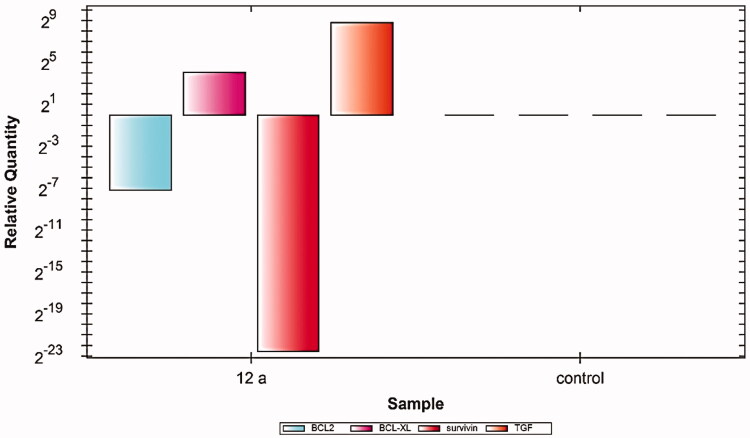
Relative gene expression levels of 4 different genes (BCL2, BCLXL, Survivin, and TGF) in Caco-2 cell line treated with **12a** using RT-qPCR.

In this study, the Caco-2 cell line was treated with 2 µM (IC_50_ value) of compound **12a**. The results showed noticeable variations in the expression levels of the four cancer correlated genes (Bcl2, Bcl-xl, TGF, and Survivin). In detail, compared to control cells, compound **12a** caused significant down-regulation of Bcl2, Bcl-xl, and Survivin genes, meanwhile, it showed an upregulation effect of the TGF gene. These findings indicate the efficiency of compound **12a** in the induction of apoptosis (Figure 4).

### 2.3. *In silico* studies

#### 2.3.1. Molecular docking

In terms of binding energy, molecular docking analysis can assist in predicting the most energetically favourable binding pose of a ligand to its receptor^62^^,^^63^. Docking simulations of compounds **8a–c** and **12a**, **b** were adopted against VEGFR-2 protein (PDB: 4ASD) using the co-crystallised ligand (sorafenib) as a reference molecule. To ensure the accuracy of the docking process, a re-docking validation step was successfully regenerated. The valid binding of the sorafenib was authenticated by the low RMSD value (1.11) ^ᵒ^A. Table 3 outlines the computed ΔG (binding free energies) of both synthesised candidates and sorafenib against VEGFR-2.

**Table 3.** Docking binding free energies (**ΔG)** of the synthesised candidates with VEGFR-2 enzyme.

Compounds	ΔG (kcal/mol)
**8a**	–26.60
**8b**	–23.97
**8c**	–23.96
**12a**	–27.44
**12b**	–26.44
Sorafenib	–26.30

**Figure 5.**
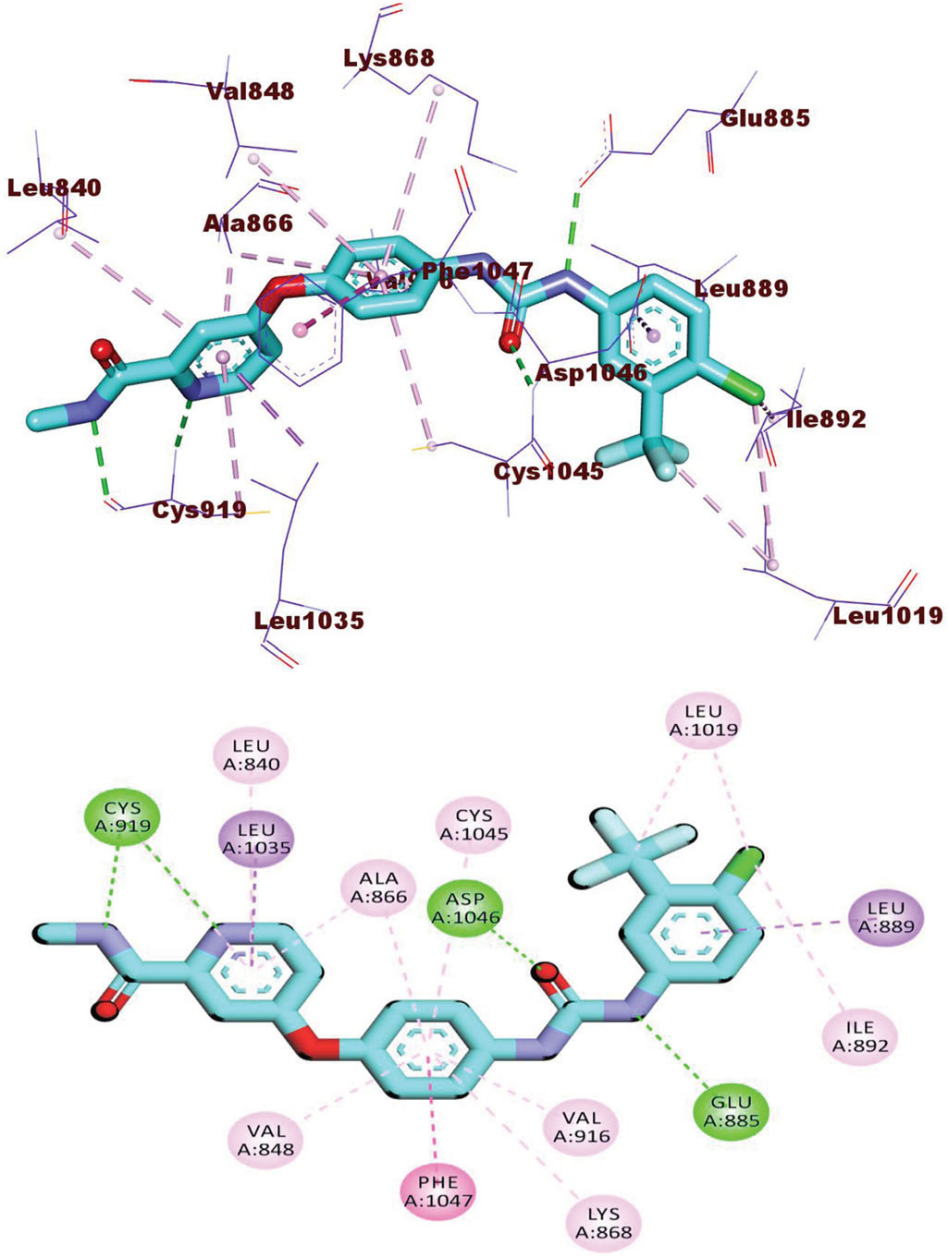
3D and 2D binding mode of sorafenib into VEGFR-2 active site.

The docking pose accomplished by sorafenib involved the H-bonding of Cys919 with the nitrogen of the pyridine ring, as well as the NH of the amide group. Additional H-bonding interaction was observed between Glu885 and NHs of the urea moiety from one side and the carbonyl oxygen of urea moiety and Asp1046 on the other side. Also, sorafenib exhibited several hydrophobic interactions with the hydrophobic pocket that formed by Leu889, Leu1019, and Ile892 (Figure 5).

**Figure 6.**
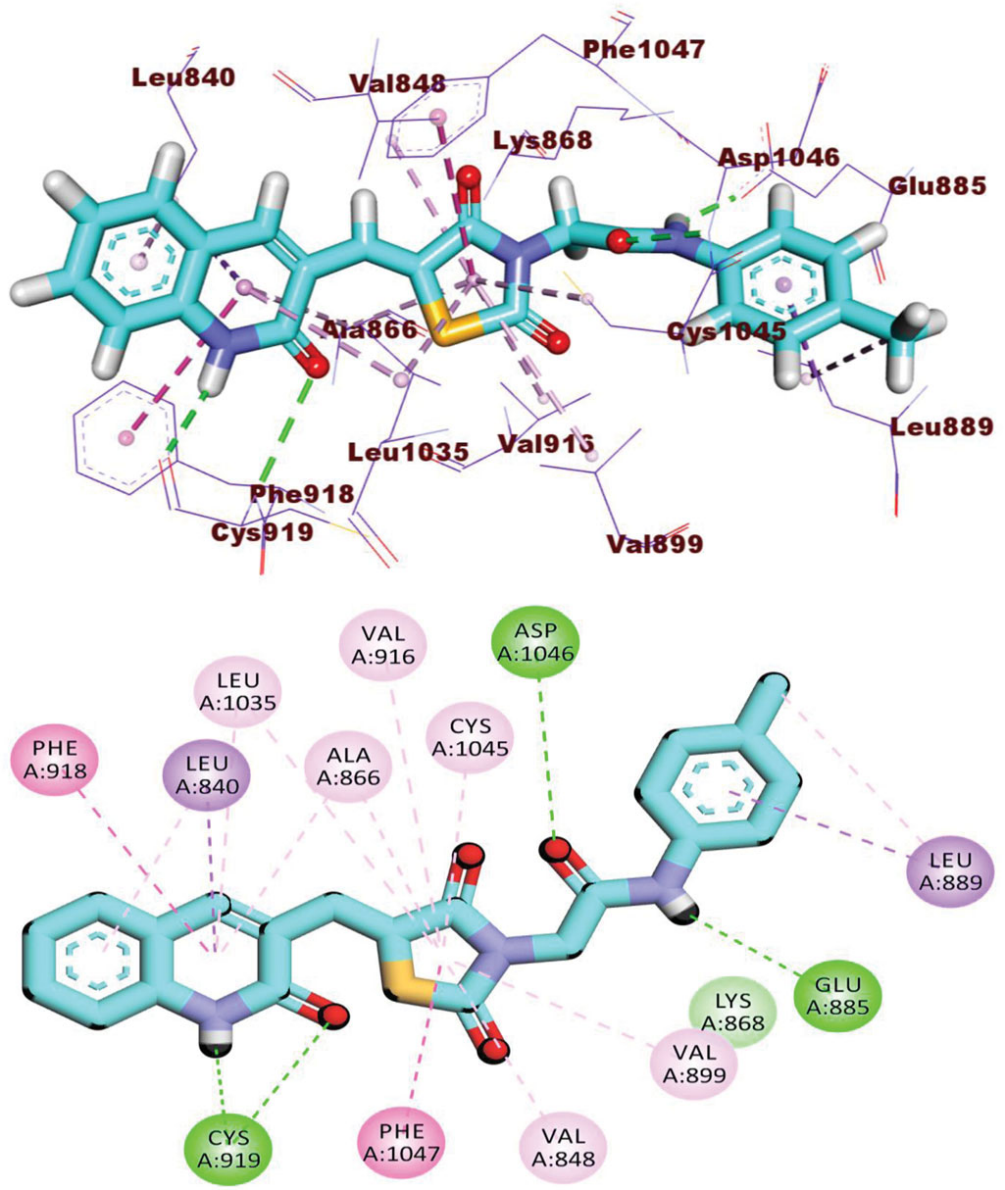
3D and 2D binding mode of **8a** with the active site of VEGFR-2.

Docking studies of compound **8a** with VEGFR-2 revealed that it has an affinity value of − 26.60 kcal/mol and formed four H-bonds. The 2-oxoquinoline arm occupied the hinge region by incorporating in two H-bonds with Cys919 and five hydrophobic interactions with Leu840, Leu1035, Ala866, and Phe918. Next, thiazolidine-2,4-dione moiety occupied the linker region by the formation of seven hydrophobic interactions with Val916, Val848, Val899, Cys1045, Leu1035, Ala866, and Phe1047. Moreover, the acetamide (pharmacophore) moiety interacted with the DFG motif region and formed two H-bonds with the vital amino acid residues Asp1046 (2.43 Å) and Glu885 (2.22 Å). Finally, the *p*-tolyl arm was buried in the hydrophobic back pocket making two hydrophobic interactions with Leu889 (Figure 6).

**Figure 7.**
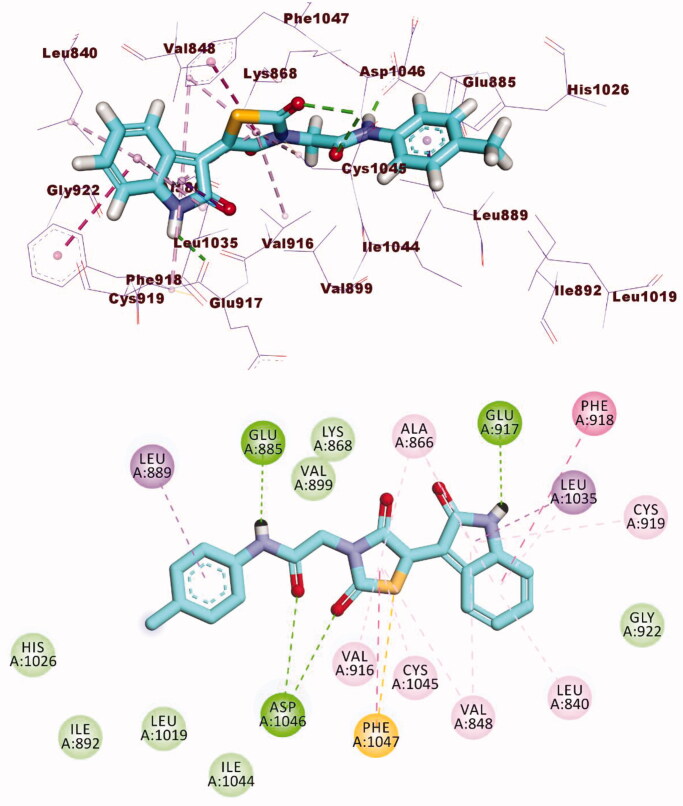
3D and 2D binding mode of **12a** with the active site of VEGFR-2.

The docking findings of compound **12a** revealed that it can interact with the essential amino acids in the VEGFR-2 active site. As displayed in Figure 7, both thiazolidine-2,4-dione and 2-oxoindoline moieties were oriented towards the gate keeper area and the hinge region, respectively. Also, compounds **12a** incorporated in two H bonds with the key amino acids; Glu885 and Asp1046 in the DFG motif. Additionally, the of the later moieties’ orientation allowed the hydrophobic substituents in the docked compounds to fit inside the hydrophobic allosteric pocket. Such a binding pattern of compound **12a** encouraged us to study its MD simulations.

#### 2.3.2. Molecular dynamics (MD) simulations

The methods of Molecular dynamics (MD) simulations are nearly to utilised as routine *in silico* work in the field of drug discovery^64^. The main two advantages of these experiments can be described as firstly, an extremely accurate ability to analyse every structural and entropic change in the examined ligand-protein system. Additionally, this analysis occurred through a decided time at an extraordinary resolution of atomic level^65^. Correspondingly, MD simulations can exactly compute the resulting changes after the ligand-protein binding in both thermodynamics and kinetics levels^66^. The previous advantages made the MD a flourishing tool to understand the structure-function relationship of the tested ligand-protein complex. It declares essential factors as the stability of the tested complex, the binding free energy, and the kinetics of the tested ligand^67^.

**Figure 8.**
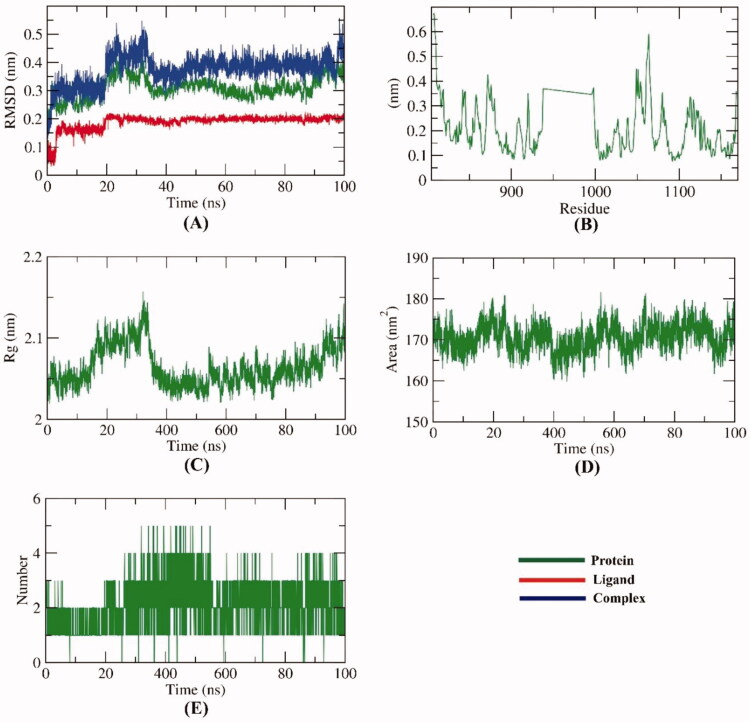
M D simulations experiment: (**A**) RMSD values of compound VEGFR-2-compound **12a** complex before and after binding, (**B**) RMSF of VEGFR-2-compound **12a** complex, (**C**) R_g_ of VEGFR-2-compound **12a** complex, **D**) SASA of VEGFR-2-compound **12a** complex, E) H- bonding between VEGFR-2-compound **12a** complex.

To compute the conformational variations that occurred in VEGFR-2- compound **12a** complex due to binding, RMSD values were calculated before and after **12a** bonding with VEGFR-2. Figure 8(A) shows that VEGFR-2, compound **12a**, and the VEGFR-2-compound **12a** complex had low values of RMSD and didn’t show any major fluctuations through the examination time (100 ns). At first 20 ns, both of VEGFR-2 and the complex were stable. Then, starting from 21 ns till 38 ns, comparatively higher RMSD has been demonstratedremained in the stable state. The RMSD values were normalised again starting from the39 ns till the end of the experiment. The achieved results reveal a great degree of stability. The flexibility of VEGFR-2 protein was examined in terms of RMSF to explore the fluctuated regions through 100 ns. It was observed that compound **12a** binding makes the VEGFR-2 slightly flexible in 1050–1100 residue areas (Figure 8(B)). The radius of gyration (Rg) of VEGFR-2 was estimated to figure out the compactness of the VEGFR-2-compound **12a** complex. Although a slight fluctuation occurred at 30 ns, the Rg of the VEGFR-2-compound **12a** complex reduced again and remained stable till the end of the experiment (Figure 8(C)). VEGFR-2-compound **12a** complex interaction with the surrounding solvents was calculated by solvent accessible surface area (SASA) over 100 ns. Interestingly, VEGFR-2 protein didn’t show a marked reduction nor expansion of its surface area showing almost similar SASA values at 0 and 100 ns (Figure 8(D)). Such results show that there are no dramatic conformational changes occurred due to the binding of compound **12a**. Hydrogen bonding among the VEGFR-2-compound **12a** complex was estimated and the maximum number of incorporated H-bonds was four (Figure 8(E)).

#### 2.3.3. MM-Pbsa

The Molecular Mechanics Poisson–Boltzmann Surface Area (MM-PBSA) is a popular *in silico* method for accurate calculation of the binding free energy of an examined compound (ligand) in the active site of a specific protein^68^. Because MM-PBSA is more precise than usual molecular docking methods and less computationally complicated than other MD free energy prediction methods, it has been widely employed in different biomolecular experiments such as protein-ligand-binding, protein-protein-interaction as well as protein folding^69^.

**Figure 9.**
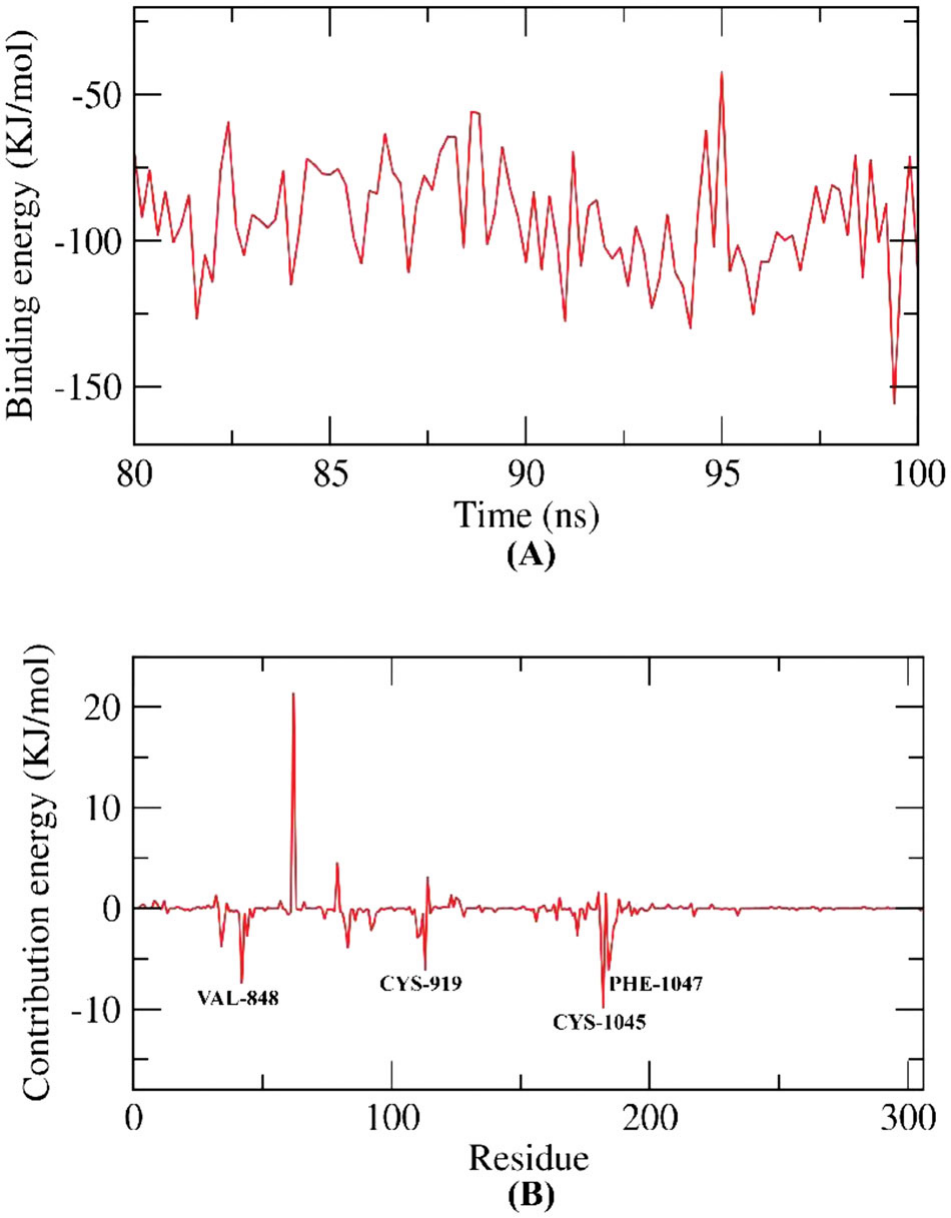
MM-PBSA analysis.

The main difference between MM/PBSA and MM/GBSA is the first experiment calculates the energies coupled with Poisson–Boltzmann, and surface area continuum solvation. While the second computes the energies coupled with the generalised Born, and surface area continuum solvation). Both methods have been employed favourably to reproduce, rationalise and improve the results obtained by virtual screening and molecular docking^70^. However, Hou et al reported a better performance for MM/PBSA over MM/GBSA in the calculation of absolute binding free energies in a comprehensive study of different 59 ligands with six proteins^71^. In this research, MM/PBSA was employed to investigate the binding free energy of the VEGFR-2-compound **12a** complex over the final 20 ns of the MD trajectories with an interval of 100 ps. Compound **12a** demonstrated a low binding free energy of −92 KJ/mol with VEGFR-2 (Figure 9(A)). Followingly, the participation of every amino acid residue of VEGFR-2 in the obtained binding free energy was computed by breaking down the total binding energy of the VEGFR-2-compound **12a** complex into per amino acid residue share energy. The fulfilled findings indicate the fundamental residues that contribute auspiciously to the binding of the VEGFR-2-compound **12a** complex. The obtained binding declared that VAL-848, CYS-919, CYS-1045 and PHE-1047 residues of VEGFR-2 contributed higher than −5 KJ/mol binding energy (Figure 9(B)). Consequently, the mentioned amino acids are considered fundamental residues in the binding with compound **12a**.

#### 2.3.4. Admet profiling study

**Figure 10.**
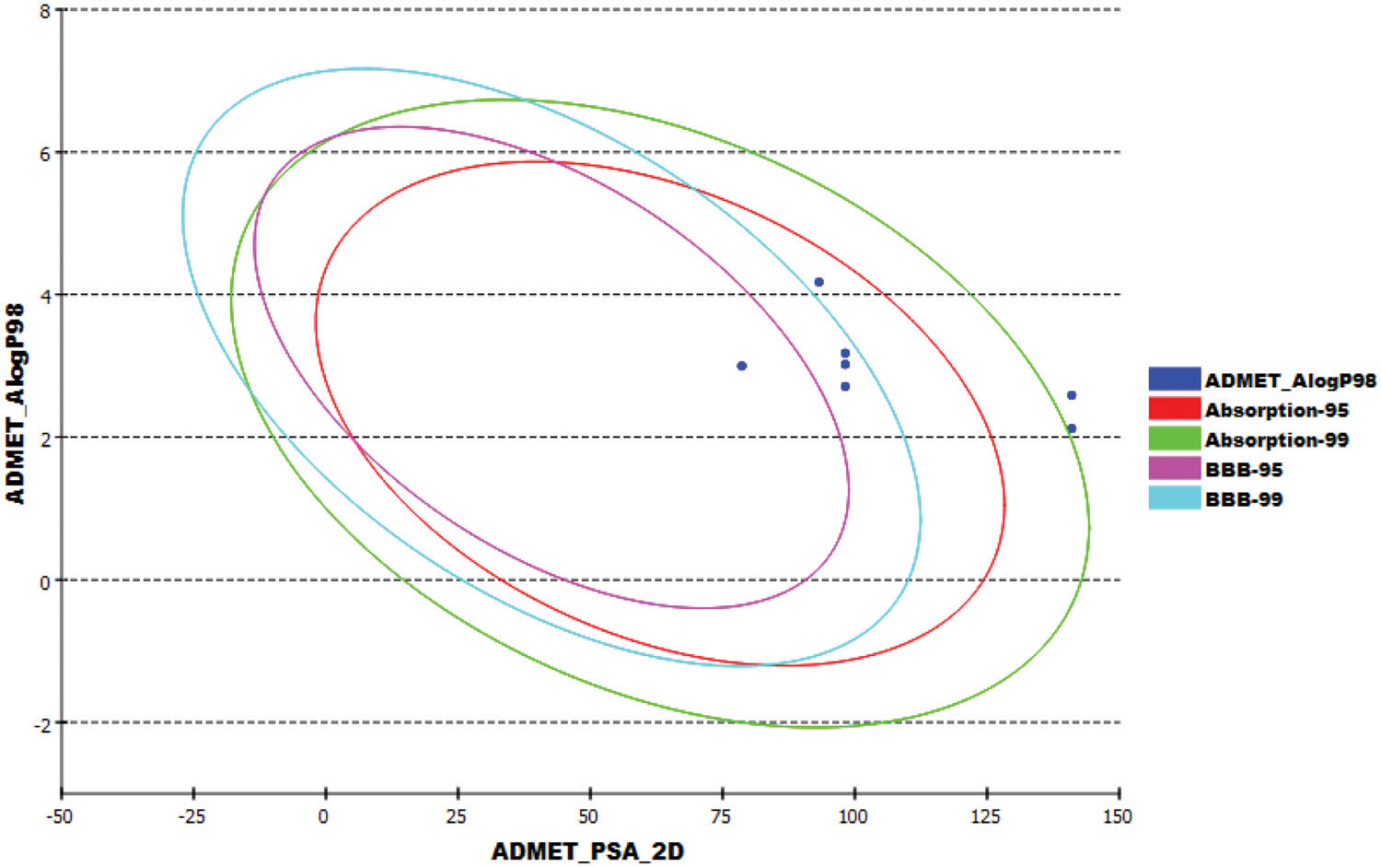
*In silico* predicted ADMET parameters for the synthesised compounds and references.

`The ADMET parameters presented in Table 4 and Figure 10 were determined computationally for compounds **8a**–**c** and **12a**, **b** using Discovery studio 4.0. Sorafenib and sunitinib were used as reference molecules.

**Table 4.** ADMET parameters for the synthesised compounds and reference molecules.

Compound	BBB^a^	Sol.^b^	Ab.^c^	CYP2D6^d^	PPB^e^
**8a**	3	2	0	NI	M
**8b**	3	2	0	NI	M
**8c**	4	2	2	NI	M
**12a**	3	2	0	NI	M
**12b**	4	2	2	NI	M
**Sorafenib**	4	1	0	NI	M
**Sunitinib**	2	2	0	NI	L

^a^BBB, blood brain barrier penetration level, 0 = very high, 1 = high, 2 = medium, 3 = low, 4 = very low.

^b^Sol, Solubility level, 1 = very low, 2 = low, 3 = good, 4 = optimal.

^c^Abs., Absorption level, 0 = good, 1 = moderate, 2 = poor, 3 = very poor.

^d^CYP2D6, cytochrome P2D6 inhibition, I = inhibitor, NI = non inhibitor.

^e^PBB, plasma protein binding, L = less than 90%, M = more than 90%.

The tested compounds exhibited low to very low BBB penetration levels. So, undesirable CNS adverse effects were expected to be absent after the administration of such molecules. For the aqueous solubility, the experimented compounds were expected to have poor solubility levels. Furthermore, compounds **8a**, **8b**, and **12a** displayed good predicted absorption levels, while compounds **8c** and **12b** demonstrated poor range. Further, all compounds didn’t displayan inhibitory potential against theCYP2D6. Finally, all experimented compounds expressedan *in silico* bind plasma protein level of more than 90%.

#### 2.3.5. *In silico* toxicity studies

In this study, seven toxicity parameters were computed based on the toxicity models created in Discovery studio 4.0^72^^,^^73^. The constructed models were presented in Table 5. Sorafenib and sunitinib were used as reference molecules.

**Table 5.** *In silico* toxicity of the synthesised compounds and reference molecules.

Compound	FDA Rodent Carcinogenicity (Rat- Female)	TD_50_ (Rat)^a^	MTD (Feed)^b^	Rat Oral LD_50_^b^	Rat Chronic LOAEL^b^	Ocular Irritancy	Skin Irritancy
**8a**	Non-Carcinogen	122.38	0.04	2.01	0.020	Mild	None
**8b**	Non-Carcinogen	812.63	0.04	0.95	0.030	Moderate	None
**8c**	Non-Carcinogen	75.48	0.03	0.77	0.014	Mild	None
**12a**	Non-Carcinogen	18.26	0.04	4.07	0.046	Mild	None
**12b**	Non-Carcinogen	11.31	0.04	1.38	0.039	Mild	None
**Sorafenib**	Non-Carcinogen	14.24	0.09	0.82	0.005	Mild	None
**Sunitinib**	Non-Carcinogen	4.13	0.18	2.88	0.040	Severe	None

^a^Unit: mg/kg body weight/day.

^b^Unit: g/kg body weight.

All investigated compounds were predicted to be non-carcinogenic showing carcinogenic potency TD_50_ values ranging from 18.26 to 812.63 mg/kg/day. Such values were higher than that of sorafenib and sunitinib (14.24, 4.13 mg/kg/day, respectively). All tested members had rat maximum tolerated doses (MTD) lower than that of sorafenib and sunitinib. Compound **12a** displayed an oral LD_50_ value of 4.07 g/kg. Such value is far more than that of sorafenib (0.82 g/kg) and sunitinib (2.88 g/kg). Also, the same compound showed rat chronic LOAEL of 0.046 g/kg which was higher than that of sorafenib and sunitinib Moreover, all the examined compounds expressed mild to moderate predicted irritation against the eye and skin.

## 3. Conclusion

A new series of 2-oxo-1,2-dihydroquinolin and 2-oxoindoline derivatives were designed and synthesised as potential anticancer agents and VEGFR-2 inhibitors. The anticancer activities of these compounds was evaluated against Caco-2, HepG-2 and MDA-MB-231 cell lines. Compound **12a** (IC_50_ = 2, 10, and 40 µM) was found to be the most active antiproliferative member against Caco-2, HepG-2 and MDA-MB-23, respectively. In addition, kinase inhibition assay results showed that all compounds had good inhibitory activity against VEGFR-2, compared to the reference drug, sorafenib. Going further, derivative **12a** was selected for further evaluation owing to its notable high selectivity index to determine whether it can also exert anticancer effects against Caco-2 cell line (chosen as the most sensitive cancer cell line). Cell migration assay confirmed that **12a** significantly inhibited the ability of cancer cells to migrate and heal at a low concentration of 1 µM. Additionally,**12a** changed the cancer cell shape from irregular to a normalised round form. The further biological assay revealed the ability of **12a** to downregulate Bcl2, Bcl-xl, and Survivin expression levels, and upregulate the TGF expression level. Molecular docking demonstrated the ability of **12a** to recognise the ATP binding pocket of VEGFR-2 and to elicit significant interactions with its key amino acids in a fashion comparable to that of the well-known VEGFR-2 inhibitor sorafenib. Molecular dynamic (MD) simulation experiments revealed that **12a** has a high potential and optimal dynamics to fit inside VEGFR-2 active site. The MM-PBSA studies determined the binding free energy against VEGFR-2 precisely to be-92 KJ/mol. Finally, the *in silico* ADMET and toxicity assessment of the synthesised candidates indicated the favourable properties as well as the satisfactory drug-like profiles. To conclude, the above results demonstrated that compound **12a** had emerged as a promising candidate and can be further adapted for hit optimisation and/or drug lead discovery.

## 4. Experimental

### 4.1. Chemistry

All the reagents, chemicals, and apparatus were described in Supplementary data. Compounds **2, 3, 5, 6,** and **10** were furnished following the reported methods^48–51^.

#### 4.1.2. Preparation of the target compounds 8a–c

A mixture of potassium 5-((2-oxo-1,2-dihydroquinolin-3-yl)methylene) thiazolidine-2,4-dione **6** (0.001 mol) and the appropriate 2-chloro-*N*-substitutedacetamide derivatives **7a–c** (0.001 mol), K_2_CO_3_ (0.001 mol) and KI(0.001 mol) in DMF (10 mL) was refluxed on a water bath for 6 h. The reaction mixture was then poured on a crushed ice. The precipitate was filtered, dried, and crystallised from ethanol to give compounds **8a–c**.

##### 4.1.2.1. 2–(2,4-Dioxo-5-((2-oxo-1,2-dihydroquinolin-3-yl)methylene)thiazolidin-3-yl)-N-(p-tolyl)acetamide 8a


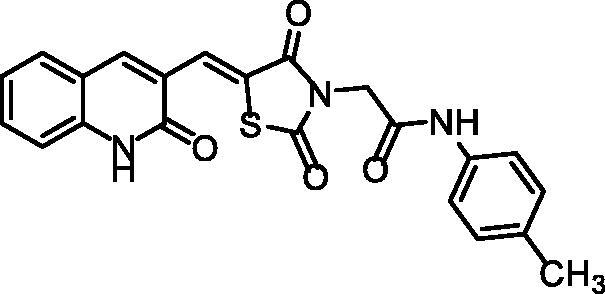


Yellowish white powder **(**yield, 71%); m. p. = 255–257 °C; IR (KBr, cm^−1^): 3447, 3273 (NH), 2984, 2920 (CH aliphatic), 1672 (C = O); ^1^H NMR (400 MHz, DMSO-d6) *δ* 12.10 (s, 1H), 10.25 (s, 1H), 8.45 (s, 1H), 7.47 (s, 1H), 7.43 (d, *J* = 2.7 Hz, 2H), 7.33 (s, 1H), 7.26 (d, *J* = 6.8 Hz, 2H), 7.21 (t, *J* = 5.4 Hz, 3H), 4.60 (s, 2H), 2.35 (s, 3H); ^13 ^C NMR (101 MHz, DMSO-d6) *δ* 171.63, 161.56, 160.73, 157.08, 155.01, 154.98, 142.25, 137.60, 136.51, 130.14, 130.08, 129.64, 127.02, 126.19, 124.16, 120.06, 119.61, 119.16, 117.30, 111.56, 56.03, 21.16; C_22_H_17_N_3_O_4_S (419.46).

##### 4.1.2.2. 2–(2,4-Dioxo-5-((2-oxo-1,2-dihydroquinolin-3-yl)methylene)thiazolidin-3-yl)-N-phenethylacetamide 8b


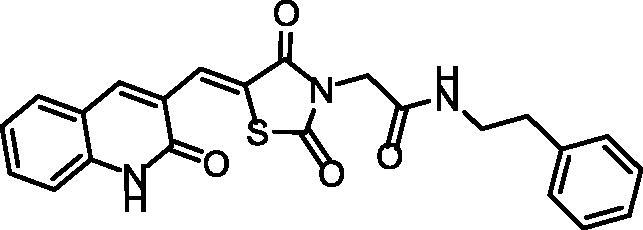


Yellowish White powder **(**yield, 69%); m. p. = 249–251 °C; IR (KBr, cm^−1^): 3302, 3142 (NH), 2982, 2914 (CH aliphatic), 1735, 1686 (C = O); ^1^H NMR (400 MHz, DMSO-*d*6) *δ* 12.18 (s, 1H), 10.25 (s, 2H), 8.44 (m, 2H), 7.47 (m, 2H), 7.30 (m, 6H), 4.60 (s, 2H), 3.54 − 3.23 (m, 4H); ^13 ^C NMR (101 MHz, DMSO-d6) *δ* 170.10, 168.60, 165.42, 165.10, 144.63, 143.08, 134.59, 133.60, 130.64, 129.30, 128.43, 128.24, 126.46, 126.13, 125.53, 122.68, 120.20, 111.16, 43.91, 40.10, 35.32. C_23_H_19_N_3_O_4_S (433.48).

##### 4.1.2.3. 2–(2,4-Dioxo-5-((2-oxo-1,2-dihydroquinolin-3-yl)methylene)thiazolidin-3-yl)-N-(2-nitrophenyl)acetamide 8c


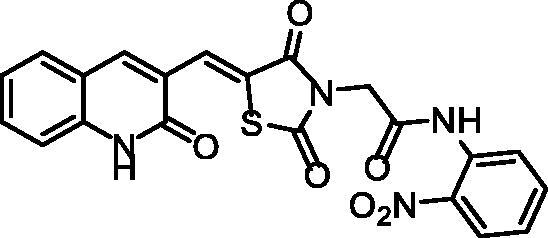


Yellow powder **(**yield, 75%); m. p. = 245–247 °C; IR (KBr, cm^−1^): 3449, 3265 (NH), 2994, 2932(CH aliphatic), 1748, 1688 (C = O); ^1^H NMR (400 MHz, DMSO-*d*6) *δ* 12.18 (s, 1H), 10.25 (s, 1H), 8.46 (s, 1H), 8.25 − 8.05 (m, 1H), 8.03 − 7.84 (m, 2H), 7.79 − 7.61 (m, 2H), 7.53 − 7.39 (m, 2H), 7.39 − 7.20 (m, 2H), 4.82 (s, 2H); ^13 ^C NMR (101 MHz, DMSO-*d*_6_) *δ* 165.10, 161.53, 160.54, 154.98, 143.11, 142.65, 142.27, 136.52, 134.41, 131.08, 130.73, 129.20, 126.32, 124.16, 120.11, 119.16, 117.31, 111.56, 56.04; C_21_H_14_N_4_O_6_S (450.43).

#### 4.1.3. Preparation of the target compounds 12a,b

A mixture of potassium 2,4-dioxo-5–(2-oxoindolin-3-ylidene)thiazolidin-3-ide, **10**, (0.001 mol) and the appropriate 2-chloro-*N*-substitutedacetamide derivatives **7a**, **c** (0.001 mol), K_2_CO_3_ (0.001 mol) and KI(0.001 mol) in DMF (10 mL) was refluxed on a water bath for 6 h. The reaction mixture was then poured on a crushed ice. The precipitate was filtered, dried, and crystallised from ethanol to give target compounds **12a**, **b.**

##### 4.1.3.1. (Z)-2–(2,4-dioxo-5–(2-oxoindolin-3-ylidene)thiazolidin-3-yl)-N-(p-tolyl)acetamide 12a


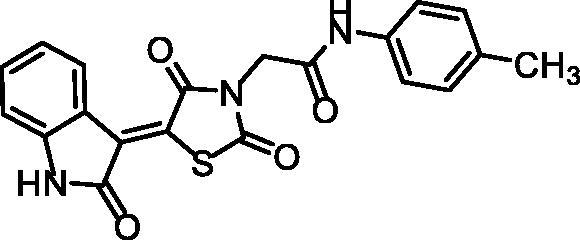


Reddish White powder **(**yield, 79%); m. p. = 271–272 °C; IR (KBr, cm^−1^): 3185, 3142 (NH), 3062 (CH aromatic) 2992, 2936 (CH aliphatic), 1745, 1690 (C = O); ^1^H NMR (400 MHz, DMSO-d6) *δ* 11.31 (s, 1H), 10.34 (s, 1H), 8.77 (d, *J* = 7.9 Hz, 1H), 7.45 (t, *J* = 6.2 Hz, 3H), 7.14 (d, *J* = 8.1 Hz, 2H), 7.09 (t, *J* = 7.8 Hz, 1H), 6.98 (d, *J* = 7.8 Hz, 1H), 4.56 (s, 2H), 2.26 (s, 3H); ^13 ^C NMR (101 MHz, DMSO-d6) *δ* 170.26, 168.72, 165.70, 163.97, 144.60, 136.31, 133.56, 133.24, 129.74 (2 C), 129.40, 128.42, 128.17, 122.67, 120.21, 119.71 (2 C), 111.15, 44.06, 20.91; C_20_H_15_N_3_O_4_S (393.42).

##### 4.1.3.2. (Z)-2–(2,4-dioxo-5–(2-oxoindolin-3-ylidene)thiazolidin-3-yl)-N-(2-nitrophenyl) acetamide 12b


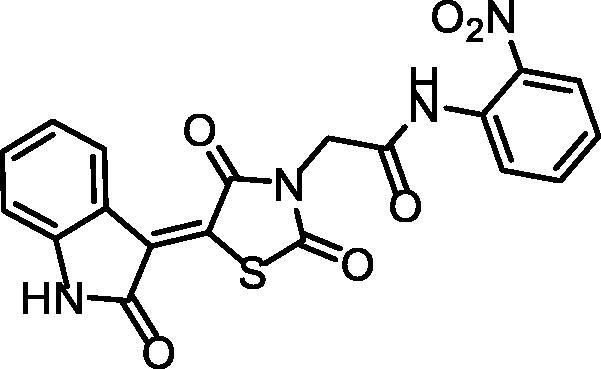


Yellow powder **(**yield, 76%); m. p. = 263–265 °C; IR (KBr, cm^−1^): 3175, 3142 (NH), 3062 (CH aromatic) 2996, 2953 (CH aliphatic), 1744, 1692 (C = O); ^1^H NMR (400 MHz, DMSO-d6) *δ* 11.32 (s, 1H), 10.74 (s, 1H), 8.78 (d, *J* = 7.9 Hz, 1H), 7.99 (dd, *J* = 8.2, 1.5 Hz, 1H), 7.77 − 7.72 (m, 1H), 7.67 (dd, *J* = 8.2, 1.4 Hz, 1H), 7.48 − 7.40 (m, 2H), 7.10 (t, *J* = 7.7 Hz, 1H), 6.99 (d, *J* = 7.8 Hz, 1H), 4.61 (s, 2H); ^13 ^C NMR (101 MHz, DMSO-d6) *δ* 170.12, 168.70, 165.52, 165.01, 144.63, 143.08, 134.59, 133.60, 130.64, 129.30, 128.43, 128.24, 126.46, 126.13, 125.53, 122.68, 120.20, 111.16, 43.91; C_19_H_12_N_4_O_6_S (424.39).

### 4.2. Biological testing

#### 4.2.1. *In vitro* anti-proliferative activities

Anti-proliferative activities of the experimented compounds were assessed against Caco-2, HepG-2, and MDA-MB-231 cell lines using MTT assay protocol^54^^,^^74^ as described in Supplementary data.

#### 4.2.2. *In vitro* VEGFR-2 kinase assay

VEGFR-2 inhibitory activity of the experimented compounds was preceded using a VEGFR-2 ELISA kit as reported and described in Supplementary data ^75^.

#### 4.2.3. Safety assay

The safety profiles of the tested compounds were checked on one non-cancerous cell line (Vero) to determine the treatment concentrations that do not depict toxic effects against the tested cells as described in Supplementary data ^76^.

#### 4.2.4. Selectivity index (SI)

The selectivity index values of the tested compounds on cancer cells were calculated as described (Supplementary data)^77^.

#### 4.2.5. Cell migration assay

Cell migration assay was fulfilled according to the reported protocol as described^78^ in Supplementary data.

#### 4.2.6. Gene expression pattern

The molecular anticancer mode of action of **12a** was investigated by screening their ability to control the gene expression levels of Bcl2, Bcl-xl, TGF and Survivin genes as reported^79^ in Supplementary data.

### 4.3. In silico *studies*

#### 4.3.1. Docking studies

Docking studies were fulfilled using MOE software against the crystal structure of VEGFR-2 [PDB ID: 4ASD] and the results were visualised using Discovery studio 4.0^80–84^ as described in Supplementary data.

#### 4.3.2. Molecular dynamics simulation

The system preparation used the web-based CHARMM-GUI^85–87^ interface utilising the CHARMM36 force field^88^ and NAMD 2.13^89^ package. The TIP3P explicit solvation model was used (Supplementary data).

#### 4.3.3. MM-Pbsa studies

The G_**MM-PBSA** package of GROMACS was utilised to calculate the MM/PBSA (Supplementary data).

#### 4.3.4. Admet studies

ADMET description was fulfilled using Discovery studio 4.0 according to the reported method^84^^, ^^90^^,^^91^ (Supplementary data).

#### 4.3.5. Toxicity studies

The toxicity of the investigated compounds was fulfilled using Discovery studio 4.0 as described^92–94^ in Supplementary data.

## Supplementary Material

Supplemental Material
