# Supplementary material for: Design and synthesis of thiazolidine-2,4-diones hybrids with 1,2-dihydroquinolones and 2-oxindoles as potential VEGFR-2 inhibitors: in-vitro anticancer evaluation and in-silico studies
Source: J Enzyme Inhib Med Chem. 2022 Jul 8;37(1):1903–17. doi: 10.1080/14756366.2022.2085693 (PMC9272924; doi:10.1080/14756366.2022.2085693)
Supplement: Supplemental Material [file IENZ_A_2085693_SM7975.pdf]

Design and synthesis of thiazolidine-2,4-diones hybrids with 1,2-dihydroquinolones and 2-oxindoles as potential VEGFR-2 inhibitors: *In-vitro* anticancer evaluation and *in-silico* studies

Mohammed S. Taghour [a], Hazem Elkady[a], Wagdy M. Eldehna[b, c], Nehal El-Deeb [d], Ahmed M. Kenawy[e], Eslam B. Elkaeed[f], Aisha A. Alsfouk[g], Mohamed S. Alesawy[a], Ahmed M. Metwaly[d,h]* Ibrahim. H. Eissa[a]*,

[a] Pharmaceutical Medicinal Chemistry & Drug Design Department, Faculty of Pharmacy (Boys), Al-Azhar University, Cairo11884, Egypt.

[b] School of Biotechnology, Badr University in Cairo, Badr City, Cairo 11829, Egypt

[c]Department of Pharmaceutical Chemistry, Faculty of Pharmacy, Kafrelsheikh University, P.O. Box 33516, Kafrelsheikh, Egypt.

[d] Biopharmaceutical Products Research Department, Genetic Engineering and Biotechnology Research Institute, City of Scientific Research and Technological Applications (SRTA-City), Alexandria 21934, Egypt.

[e] Nucleic Acids Research Department, Genetic Engineering and Biotechnology Research Institute. City of Scientific Research and Technological Applications (SRTA-City). Alexandria 21934, Egypt

[f] Department of Pharmaceutical Sciences, College of Pharmacy, AlMaarefa University, Riyadh 13713, Saudi Arabia.

[g] Department of Pharmaceutical Sciences, College of Pharmacy, Princess Nourah bint Abdulrahman University, P.O. Box 84428, Riyadh 11671, Saudi Arabia.

[h] Pharmacognosy and Medicinal Plants Department, Faculty of Pharmacy (Boys), Al-Azhar University, Cairo 11884, Egypt.

***Corresponding authors:**
Ibrahim H. Eissa

Medicinal Chemistry Department, Faculty of Pharmacy (Boys), Al-Azhar University, Cairo 11884, Egypt. **Email:** Ibrahimeissa@azhar.edu.eg

Ahmed M. Metwaly
Pharmacognosy and Medicinal Plants Department, Faculty of Pharmacy (Boys), Al-Azhar University, Cairo 11884, Egypt. **Email:** ametwaly@azhar.edu.eg

Content

1	**Biological testing**<br>✓ Safety assay<br>✓ *In vitro* anti-proliferative activity.<br>✓ *In vitro* VEGFR-2 kinase assay.<br>✓ Selectivity index (SI)<br>✓ Wound healing assay (Migration assay).<br>✓ Gene expression pattern alternation of cancer cell after **12a** treatment.
2	***In silico* studies**<br>✓ Docking studies<br>✓ ADMET studies<br>✓ Toxicity studies<br>✓ MD simulation<br>✓ MMPBSA
3	**Chemistry and materials**
4	**Spectral data**
5	**Raw data for VEGFR-2 assay of the tested compounds**

1- Biological testing

a- Mammalian cell lines culture

WISH, CaCo-2, and A549 cell lines were cultured on DMEM media, meanwhile MDA-MB-231 and hepG-2 cell line were cultured on RBMI media. The cultured media were supplemented with 200 mM L-glutamine, 10.0% fetal bovine serum (Lonza), and 1.0% penicillin/streptomycin. Cells were seeded into 25.0 cm tissue culture flasks and incubated at 37°C in a 5.0% CO_2 incubator for 24 h or till confluency.

b- Safety assay

The safety profiles of the tested compounds were checked on one non-cancerous cell line (Vero) to determine the treatments concentrations that do not depict toxic effects against the tested cells. A portion of 100.0 µl of 6×10^4 cell/ml cells was seeded into each well of a 96-well plate and then the plates were incubated at 37°C in a humidified 5.0% CO_2 incubator for 24 h. At the end of incubation period, the exhausted medium was replaced with 100.0 µl of different concentrations of the designated treatment (prepared in RPMI medium starting from 1.0 mM). The inoculated plates were incubated at the same growth conditions for another 24 h. At the end of incubation, cellular viability was assessed using MTS assay kit (Promega) according to the manual instruction.

c- *In-vitro* anticancer activity

Anticancer activities of the tested compounds against CaCo-2, MDA-MB-231, and hepG-2 cell lines were quantified using MTS assay kit (Promega) as described by the Manufacturer.

d- Selectivity index (SI)

The selectivity index values of the tested compounds on cancer cells were calculated as described by Koch et al. [57], with slight modifications; $SI=IC_{50}nc/IC_{50}cc$, where $IC_{50}nc$: the IC_{50} value of the tested compound on normal cells and $IC_{50}cc$: IC_{50} of the tested compound on cancer cell line.

e- Wound healing assay (Migration assay)

CaCo-2 cells were grown to 95.0% confluency in a complete DMEM medium and then the wounds were formed using a plastic tip. After washing with pre-warmed PBS, the cells were incubated in the specific medium or the **12a** treatment. After incubation at 37°C and 5.0% CO_2 for 24h, the cells were washed with PBS and the wounds distance was determined as the scratch width of the treated and untreated groups using ImageJ software.

f- **Gene expression pattern alternation of cancer cell after 12a treatment**

The molecular anticancer mode of action of **12a** was investigated by screening their ability to affect the gene expression levels of Bcl2, Bcl-xl, TGF and Survivin genes using specific forward and reverse primers and RTq-PCR technique (Table 1) in CaCo-2 cells (chosen as the most sensitive cancer cell line). After cellular treatment, CaCo-2 cell line was cultured into 12 well plates (6×10^3 cell/ml) for 24 h. with the sub-IC50 concentration of **12a**. After treatment, total RNA extraction was performed using RNA extraction kit (Qiagen, Germany). Then, 1 ug of the obtained RNA was used to synthesiz cDNA using cDNA synthesis kit (Promega Corp., Madison, WI) as recommended by the manufacturer. Simultaneously, GAPDH forward and reverse primers (Table 1) were used to amplify the house keeping gene as internal control for standardization of PCR products. The RTq-PCR was done using SYBR Green dye (QuantiTect SYBR Green PCR Kits) and Light Cycler fluorimeter (Bio-RAD S1000 Tm thermal cycler). The PCR cycling program was as follows: 95°C for 2 min, followed by 40 cycles of 95°C for 30s, 55°C for 30 s, and 60°C for 45s, and finally 60°C for 5 min.

-Sequence of the primers

Primer ID	Sequence
Bcl-F	5′-TATAAGCTGTCGCAGAGGGGCTA-3′
Bcl-R	5′-GTACTCAGTCATCCACAGGGCGAT-3′
Bcl-Xlf	5’CAGAGCTTTGAACAGGTAG-3′
Bcl-XlR	5’GCTCTCGGGTGCTGTATTG-3′
Surv-F	5′-TGCCCCGACGTTGCC-3′
Surv-R	5′-CAGTTCTTGAATGTAGAGATGCGGT-3′
TGF-F	5′CAAGGGCTACCATGCCAACT3′
TGF-R	5′AGGGCCAGGACCTTGCTG3′
β-actin-F	5′-GTGGGGCGCCCCAGGCACCA-3′
β-actin-R	5′-CTCCTTAATGTCACGCACGATTTC-3′

2- ***In silico* studies**

a- Docking studies

The docking studies were performed utilizing MOE.14 software to explore the binding mode of the synthesized compounds towards VEGFR-2. The 3D crystal structures of the target macromolecules VEGFR-2 were downloaded from the protein databank, http://www.pdb.org (PDB ID; 2OH4 and 4ASD). Sorafenib was used as reference ligand. To prepare the target protein, water molecules were removed, and the valances of atoms were corrected through protonation of the whole molecule. Then energy minimization was carried out by applying CHARMM and MMFF94 force fields. After that, the active binding site was defined and prepared for docking. The validation process was performed by redocking the co-crystallized ligand. The designed compounds together with sorafenib were drawn using ChemBioDraw Ultra 14.0 and saved as MDL-SD format. The sketched compounds were constructed from fragment libraries in MOE program, protonated, followed by energy minimization then prepared for docking. Docking process was carried through Triangle matcher placement inserted in compute window, and the scoring function was London dG. Ten conformers (poses) for each molecule were generated using genetic algorithm searches. The free energies and binding modes of the designed molecules against VEGFR-2 were determined. The most ideal pose was selected according to its binding free energy as well as its binding mode with target molecule. This method is based on the chemistry of the co-crystallized ligand and building three-dimensional template to represent characteristic distributions of hydrogen bonds, hydrophobicity, and other chemical parameters.

b- ADMET studies

ADMET descriptors (absorption, distribution, metabolism, excretion and toxicity) of the synthesized compounds were determined using Discovery studio 4.0. At first, the CHARMM force field was applied then the compounds were prepared and minimized according to the preparation of small molecule protocol. Then ADMET descriptors protocol was applied to carry out these studies.

c- Toxicity studies

The toxicity parameters of the synthesized compounds were calculated using Discovery studio 4.0. Sorafenib was used as a reference drug. At first, the CHARMM force field was applied then the compounds were prepared and minimized according to the preparation of small molecule protocol. Then different parameters were calculated from toxicity prediction (extensible) protocol.

d- **Molecular dynamics simulation**

Molecular dynamics simulation of the protein-ligand complexes was performed using GROMACS 2021 and Linux 5.4 package. The GROMOS96 54a7 forcefield was selected as the force field for proteins and the ligand topologies were generated from the PRODRG server. All the complexes were solvated using simple point charge (SPC) water molecules in a rectangular box. To make the simulation system electrically neutral, required number of Na^+ and Cl^- ions were added while 0.15 mol/L salt concentrations were set in all the systems. Using the steepest descent method, all the solvated systems were subjected to energy minimization for 5000 steps. Afterwards, NVT (constant number of particles, volume, and temperature) series, NPT (constant number of particles, pressure, and temperature) series, and the production run were conducted in the MD simulation. The NVT and the NPT series were conducted at a 300 K temperature and 1 atm pressure for the duration of 300 ps. V-rescale thermostat and Parrinello-Rahman barostat were selected of the performed simulation. Finally, the production run was performed at 300 K for a duration of 100 ns (nanoseconds). Thereafter, a comparative analysis was performed measuring root mean square deviation (RMSD), root mean square fluctuation (RMSF), radius of gyration (Rg), solvent accessible surface area (SASA) and hydrogen bonds to analyze their stability. The Xmgrace program was used to represent the analyses in the form of plots.

e- **MM/PBSA**

The g_mmpbsa package of GROMACS was utilized to calculate the MM/PBSA (Molecular Mechanics/Poisson Boltzmann Surface Area) binding free energies followed by final MD production run to get a detailed overview of the molecular interactions between the protein and ligand. The free solvation energy (polar and nonpolar solvation energies) and potential energy (electrostatic and Van der Waals interactions) of each protein-ligand complex were analyzed to determine the total ΔG_{bind} of the complex. The binding energies were calculated using the following equation in this method:

$$\Delta G_{binding} = G_{complex} - (G_{protein} + G_{ligand})$$

Here, the $\Delta G_{binding}$ = the total binding energy of the protein-ligand complex, $G_{protein}$ = the binding energy of free protein, and G_{ligand} = the binding energy of unbounded ligand.

3- Chemistry and material

All melting points were carried out by open capillary method on a Gallen kamp Melting point apparatus. The infrared spectra were recorded on pye Unicam SP 1000 IR spectrophotometer using potassium bromide disc technique. Proton magnetic resonance 1HNMR spectra were recorded on a Bruker 400 Megahertz-nuclear magnetic resonance (400 MHZ-NMR) spectrophotometer. Carbon-13 (C13) nuclear magnetic resonance (13CNMR) spectra were recorded on a Bruker 100 Megahertz-nuclear magnetic resonance (100 MHZ-NMR) spectrophotometer. Tetramethylsilane (TMS) was used as internal standard and chemical shifts were measured in δ scale one part per million (ppm). All compounds were within ±0.4 of the theoretical values. The reactions were monitored by thin-layer chromatography (TLC) using TLC sheets precoated with UV fluorescent silica gel Merck 60 F254 plates and were visualized using ultraviolet (UV) lamp and different solvents as mobile phases.

IR of compound 8a

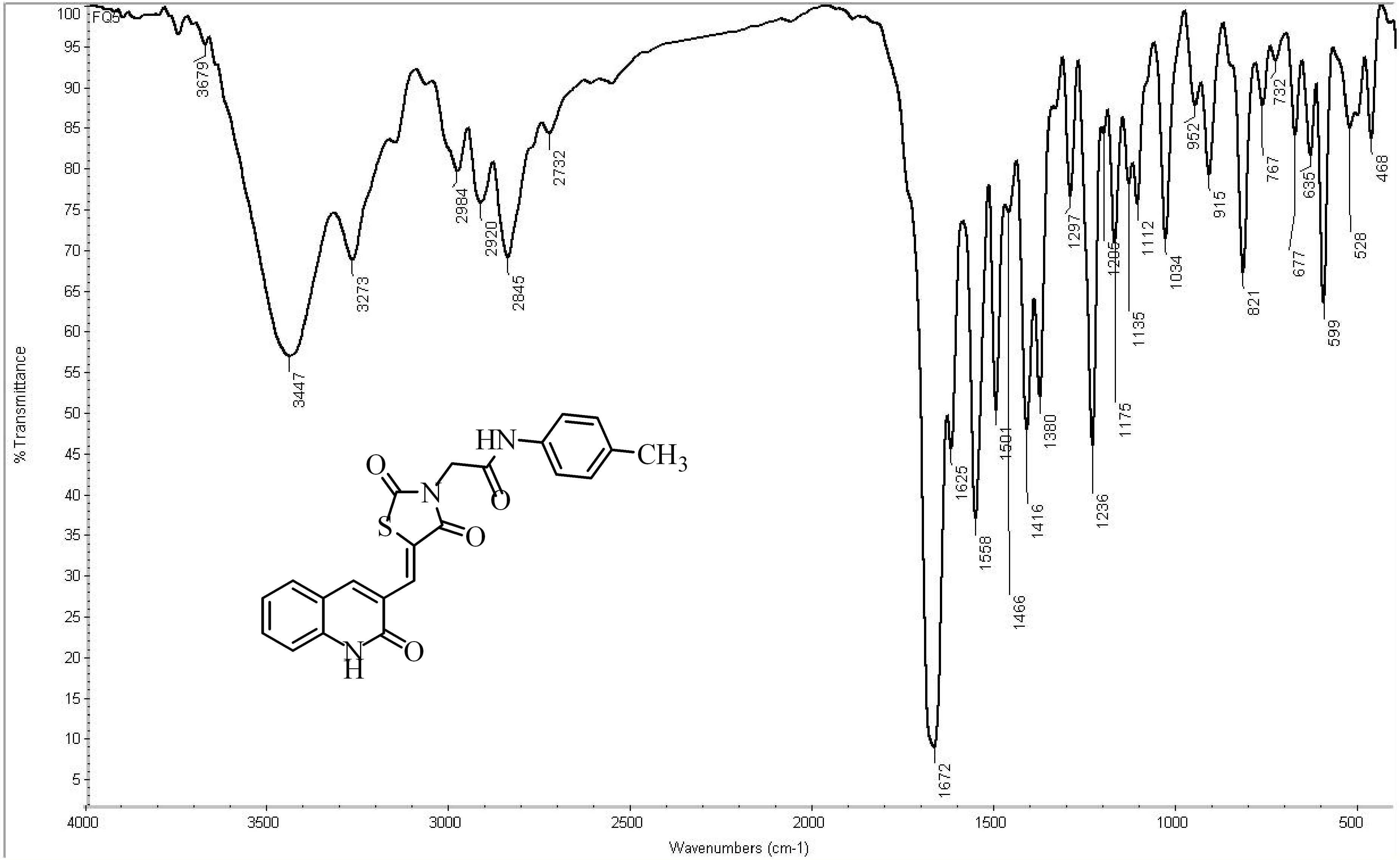

IR of compound 8b

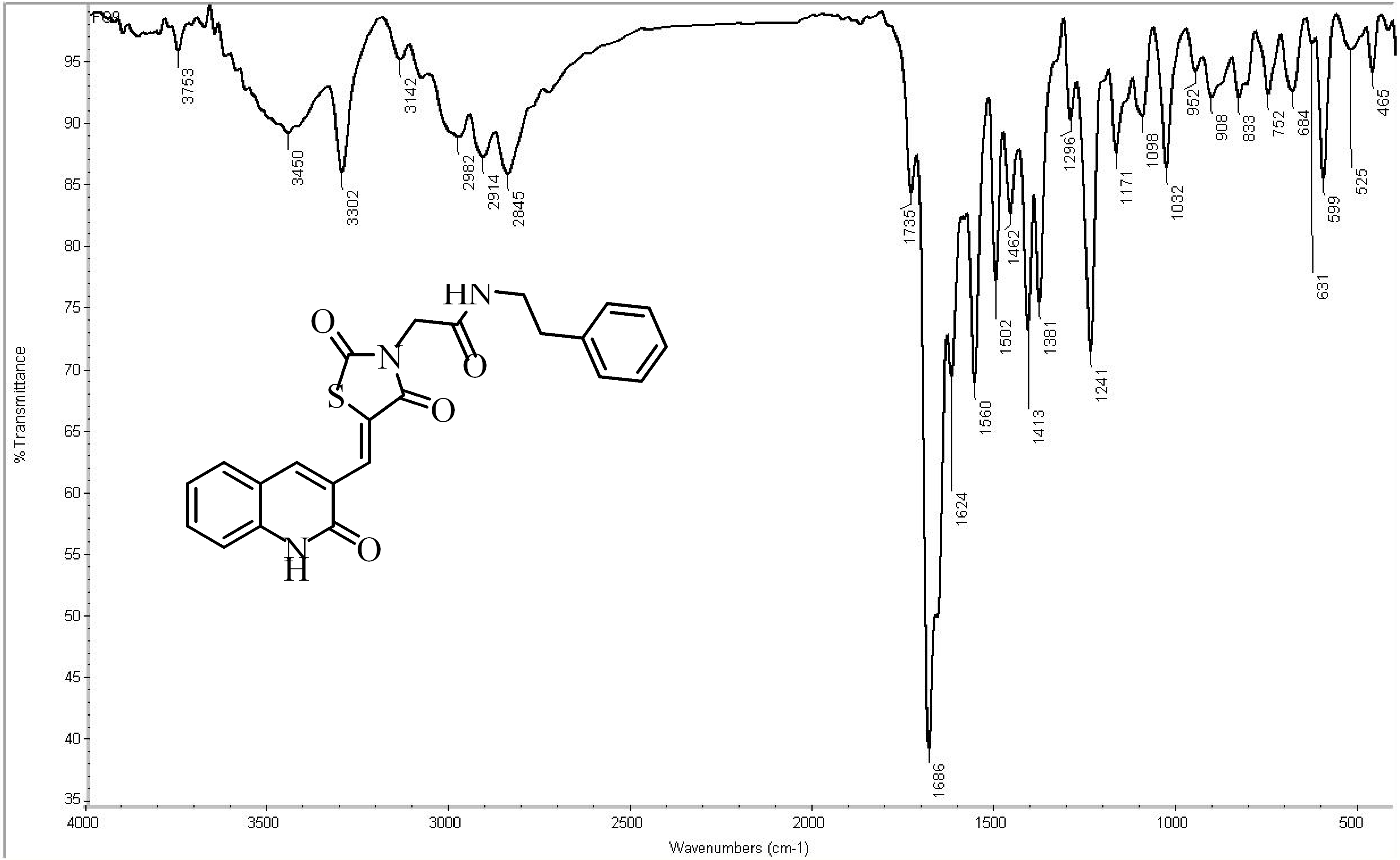

IR of compound 8c

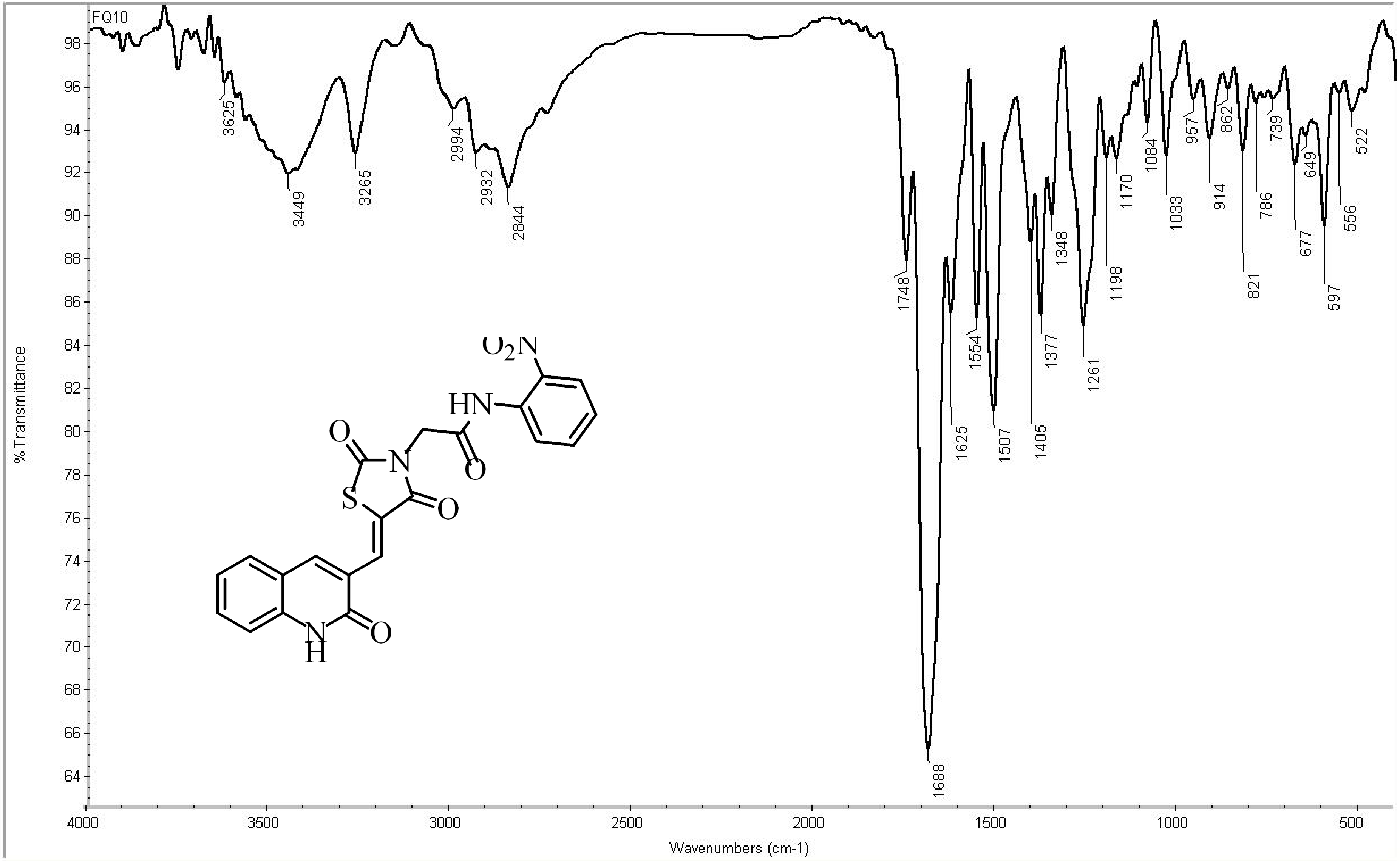

IR of compound 12a

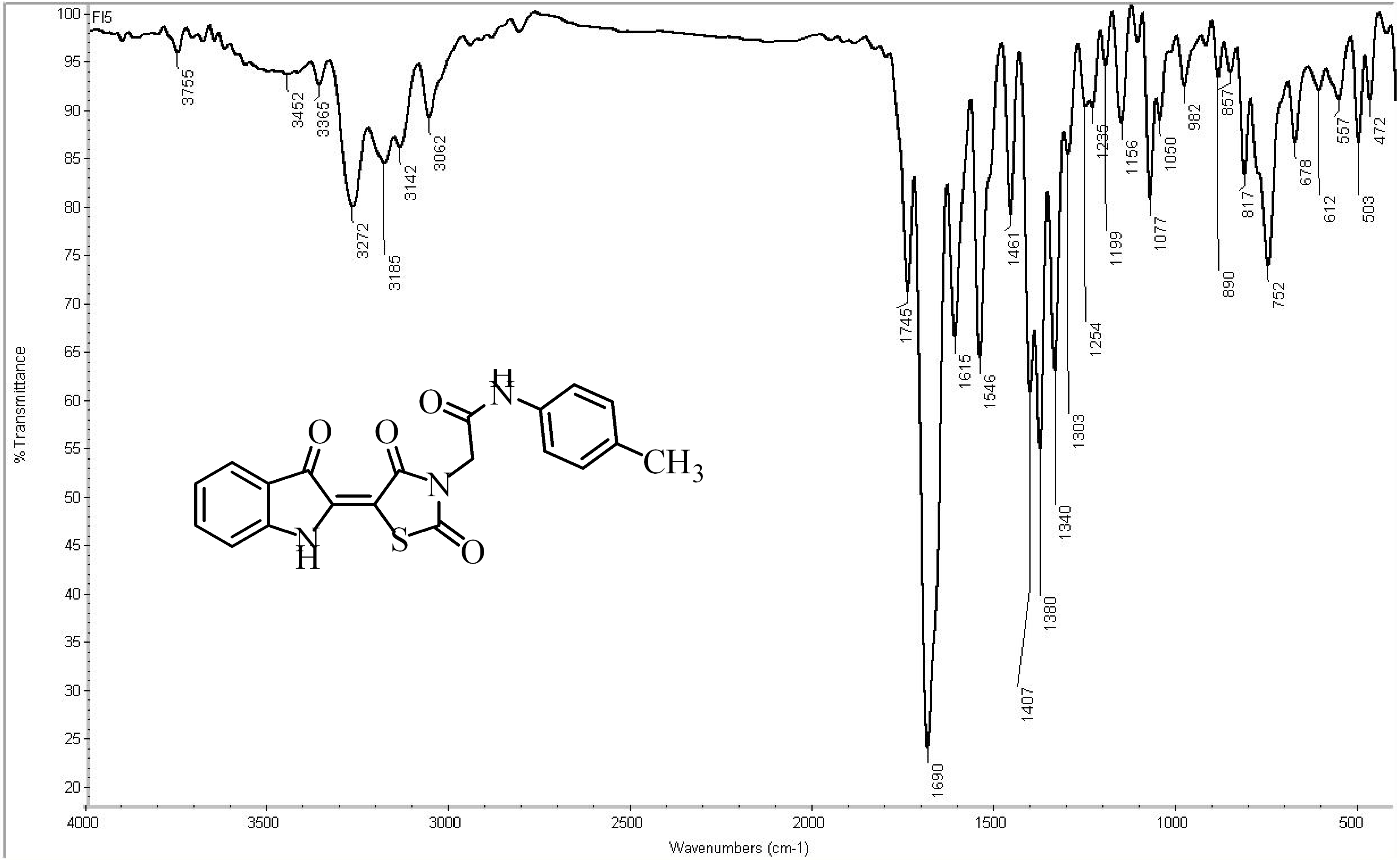

IR of compound 12b

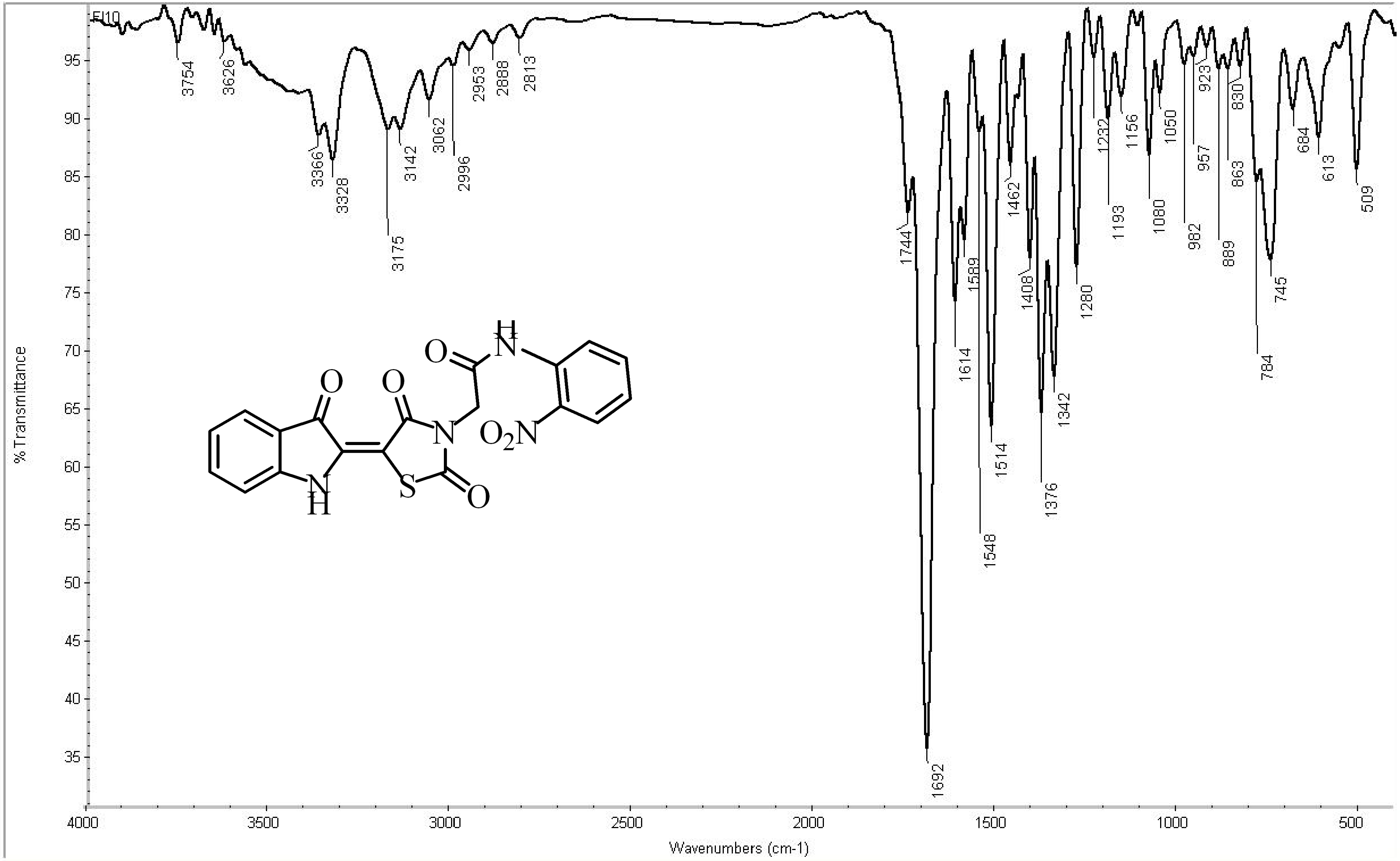

1H NMR 12a

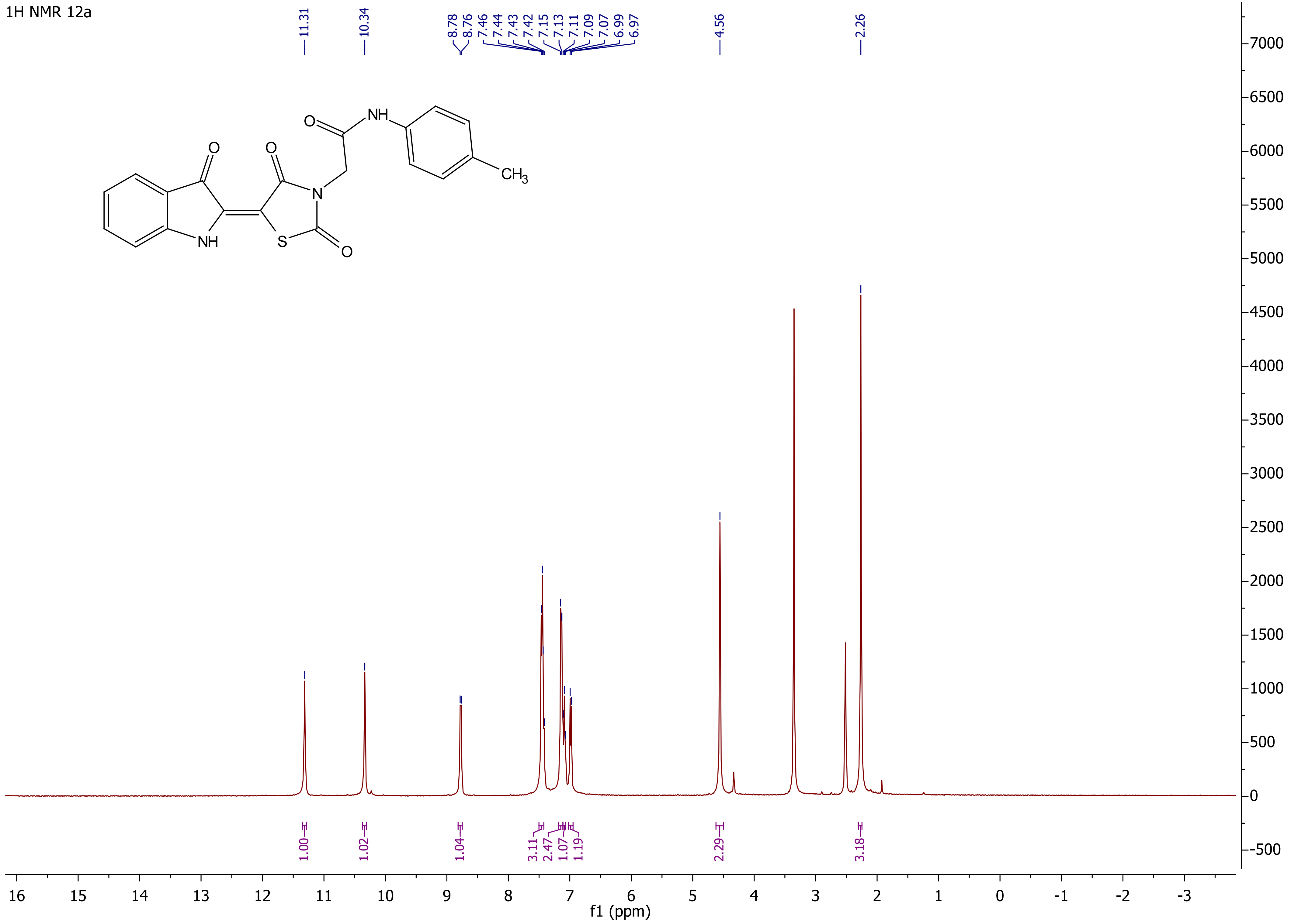

11.31
10.34
8.78
8.76
7.46
7.44
7.43
7.42
7.15
7.13
7.11
7.09
7.07
6.99
6.97
4.56
2.26
1.00
1.02
1.04
3.11
2.47
1.07
1.19
2.29
3.18
f1 (ppm)
16
15
14
13
12
11
10
9
8
7
6
5
4
3
2
1
0
-1
-2
-3
7000
6500
6000
5500
5000
4500
4000
3500
3000
2500
2000
1500
1000
500
0
-500
O
NH
O
O
N
CH3
NH
S
O

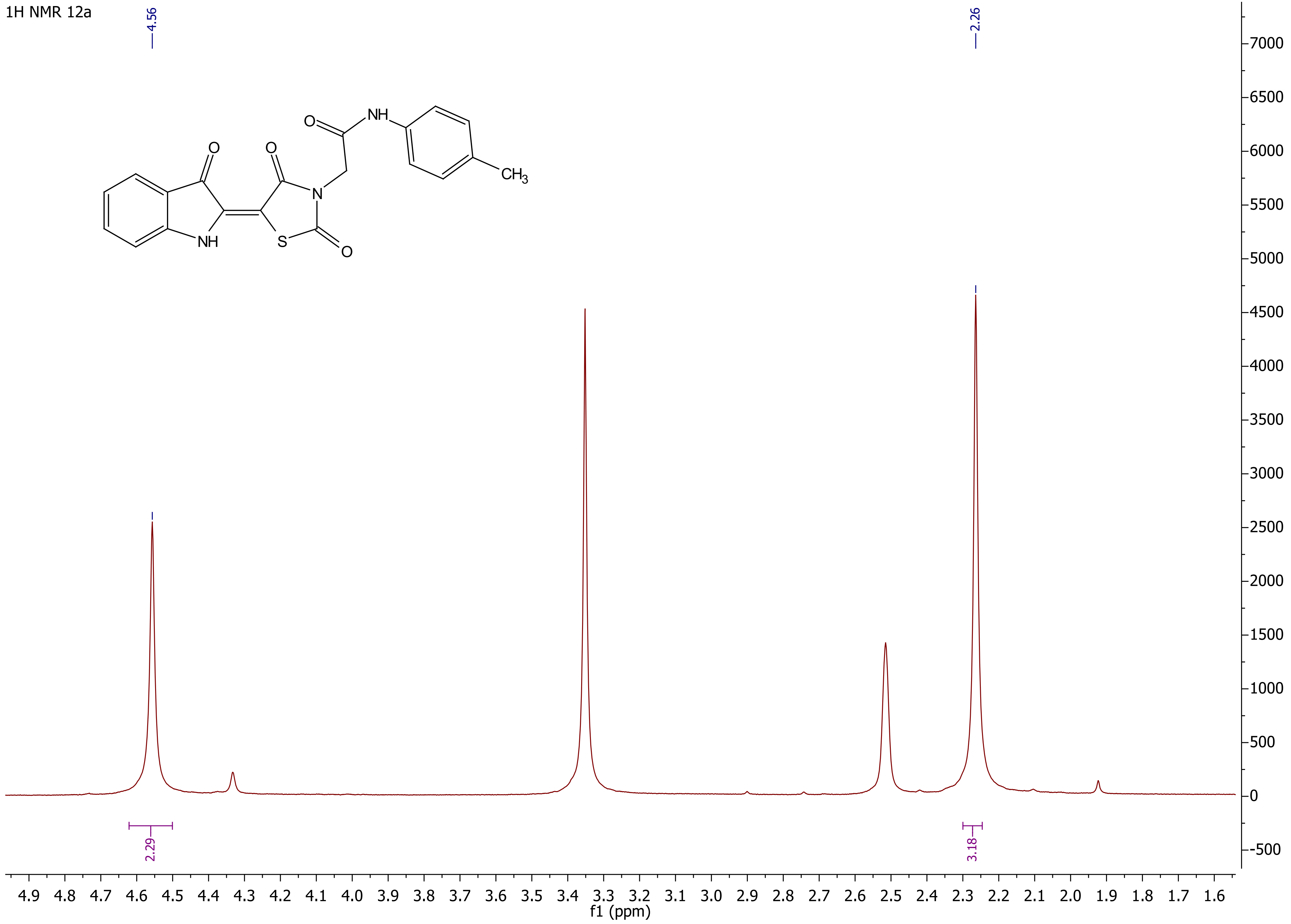
1H NMR 12a
4.56
2.26
O
NH
O
O
CH3
N
NH
S
O
2.29
3.18
7000
6500
6000
5500
5000
4500
4000
3500
3000
2500
2000
1500
1000
500
0
-500
4.9 4.8 4.7 4.6 4.5 4.4 4.3 4.2 4.1 4.0 3.9 3.8 3.7 3.6 3.5 3.4 3.3 3.2 3.1 3.0 2.9 2.8 2.7 2.6 2.5 2.4 2.3 2.2 2.1 2.0 1.9 1.8 1.7 1.6
f1 (ppm)

1H NMR 12a

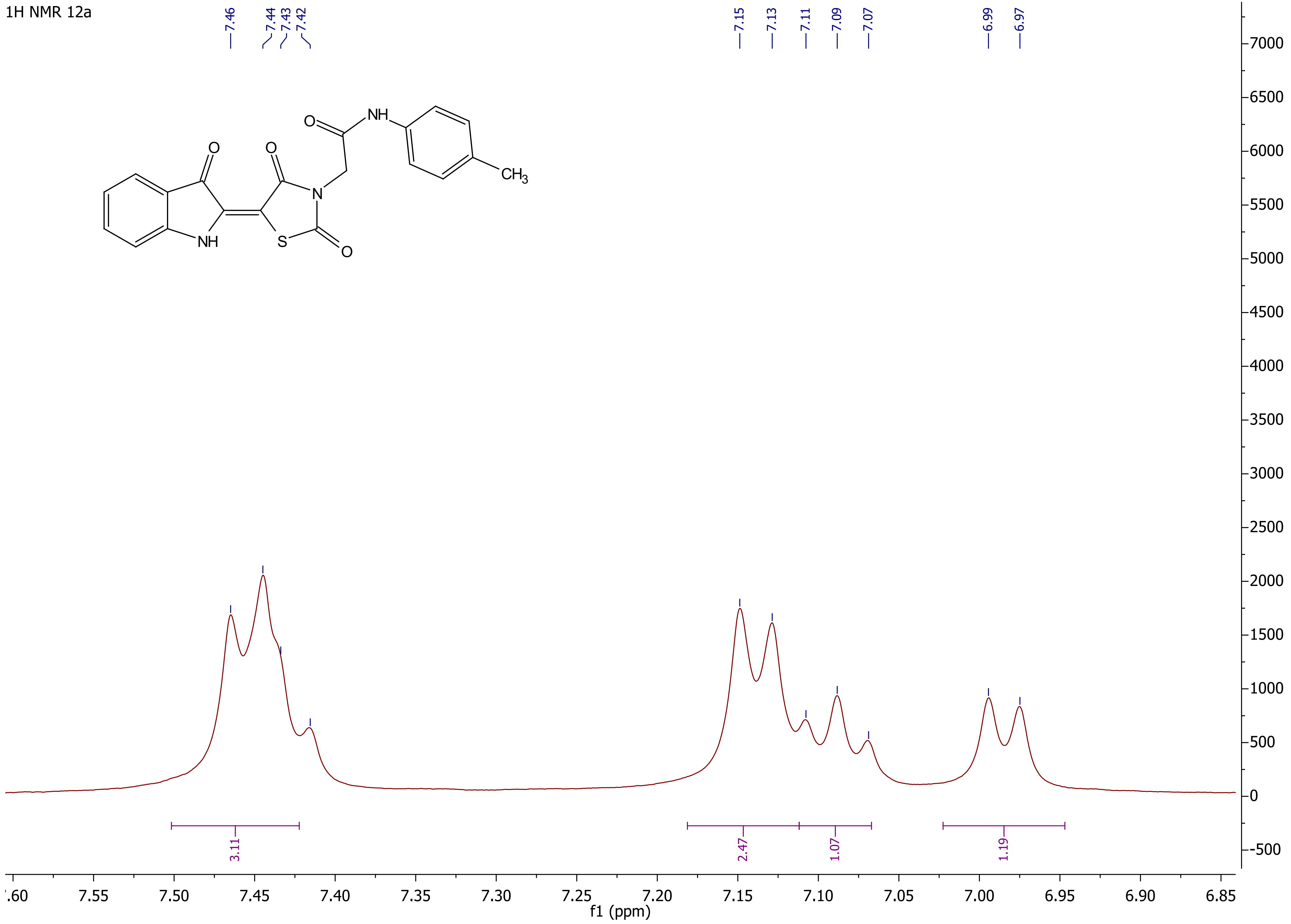

7.46
7.44
7.43
7.42
7.15
7.13
7.11
7.09
7.07
6.99
6.97
O
O
NH
O
N
CH3
NH
S
O
3.11
2.47
1.07
1.19
f1 (ppm)

13C NMR 12a

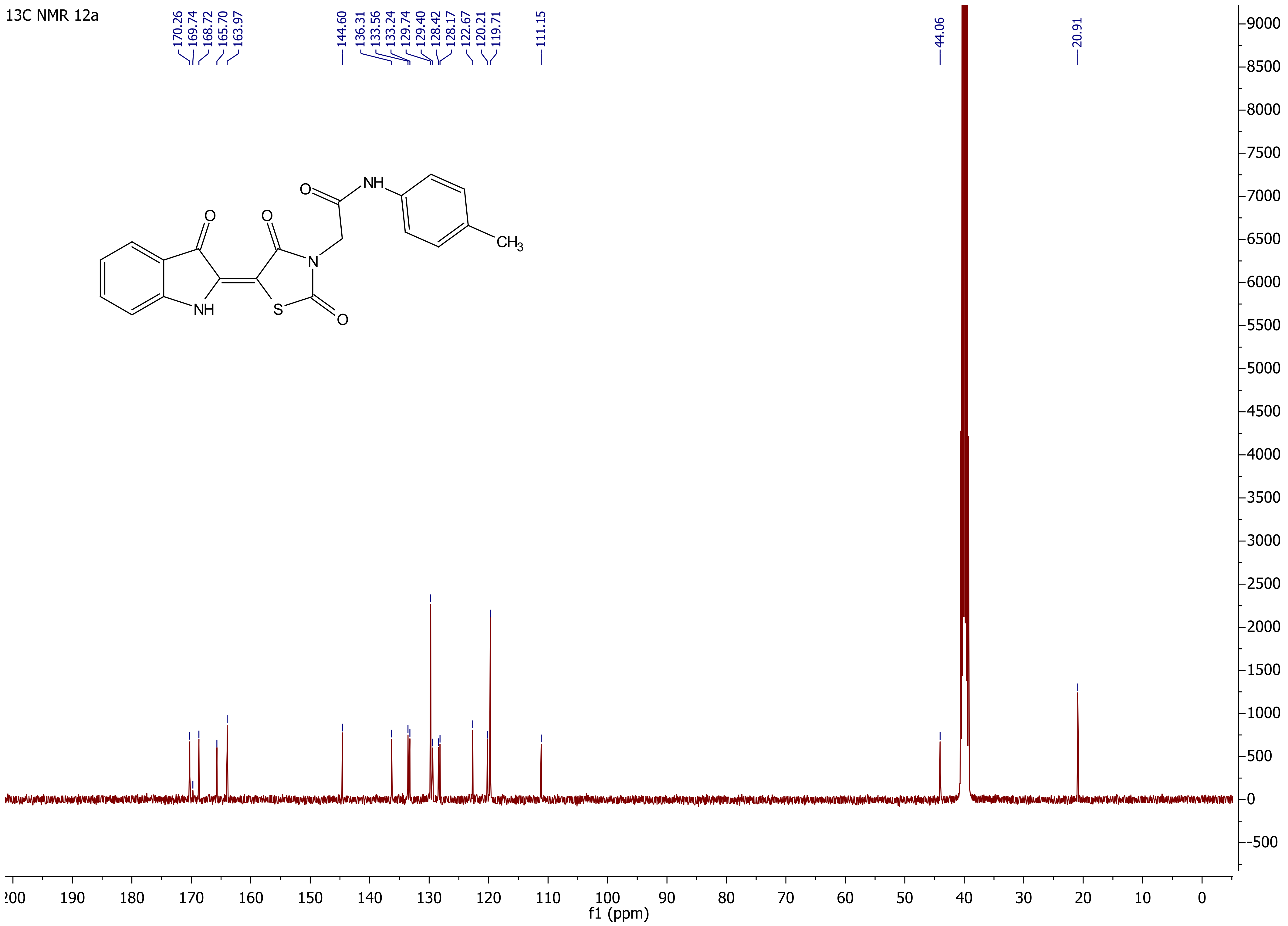

170.26
169.74
168.72
165.70
163.97
144.60
136.31
133.56
133.24
129.74
129.40
128.42
128.17
122.67
120.21
119.71
111.15
44.06
20.91
O
NH
O
O
N
NH
S
O
CH3
f1 (ppm)

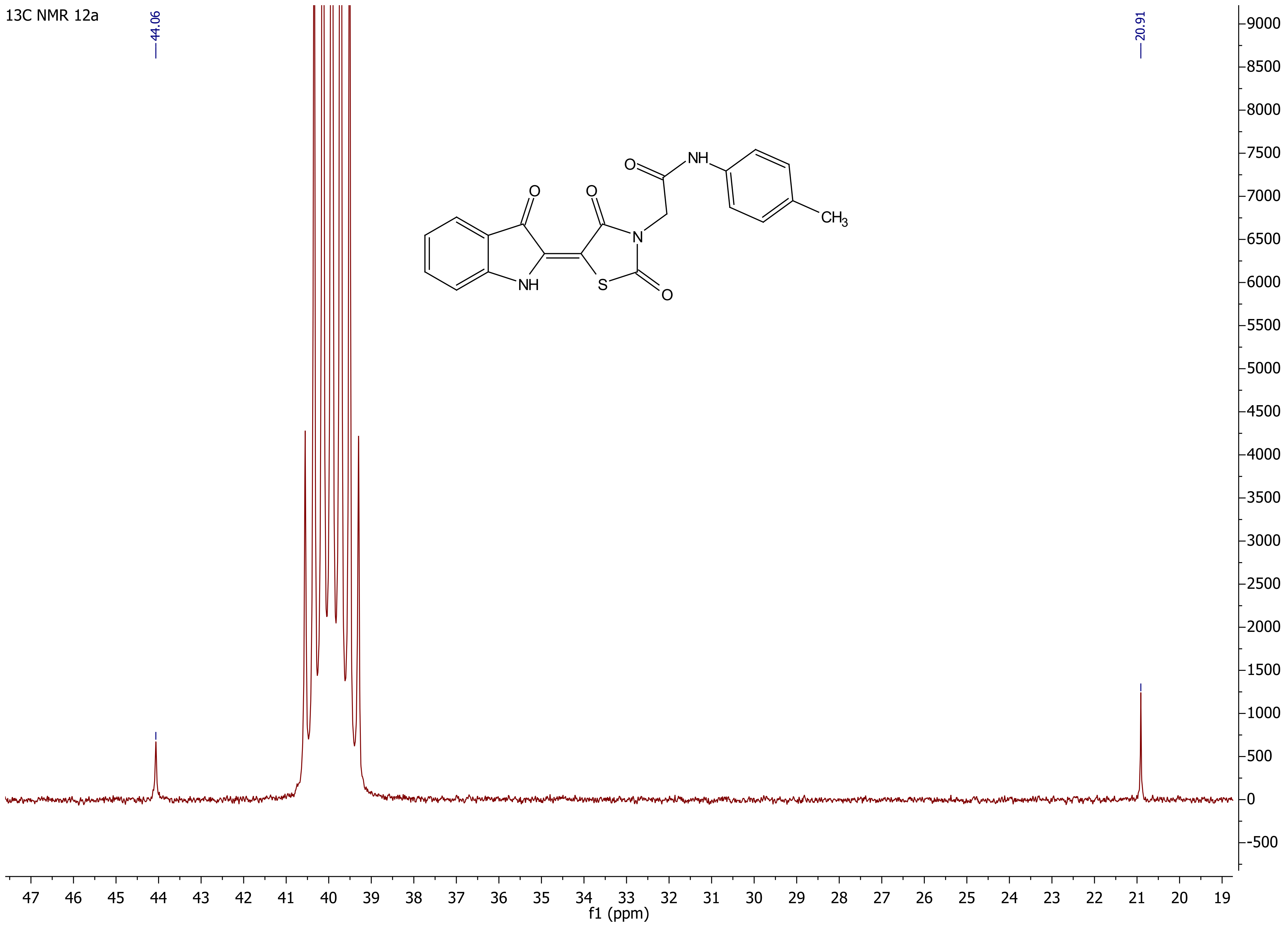

13C NMR 12a
44.06
20.91
O
NH
O
O
N
NH
S
O
CH3
9000
8500
8000
7500
7000
6500
6000
5500
5000
4500
4000
3500
3000
2500
2000
1500
1000
500
0
-500
47
46
45
44
43
42
41
40
39
38
37
36
35
34
33
32
31
30
29
28
27
26
25
24
23
22
21
20
19
f1 (ppm)

13C NMR 12a

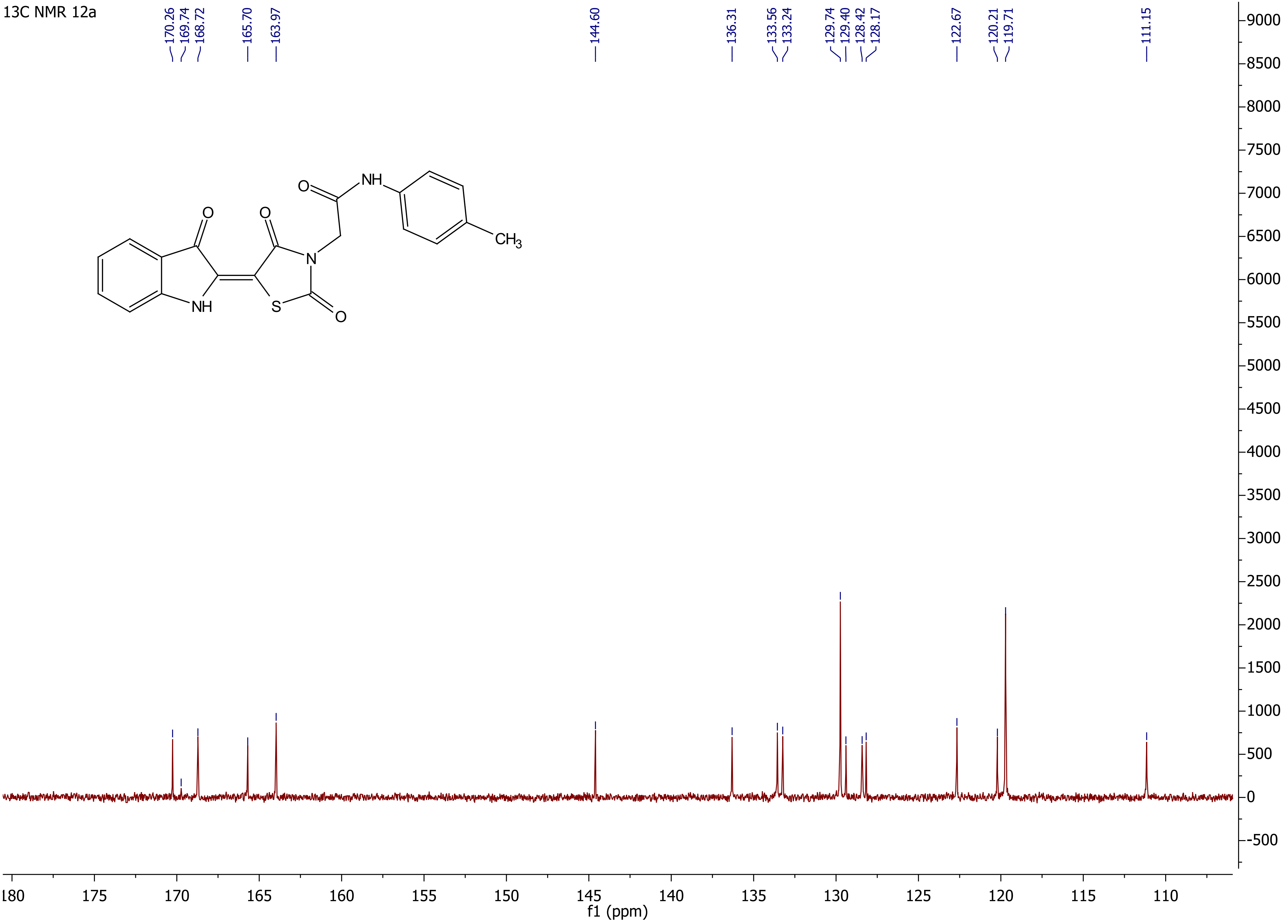
170.26
169.74
168.72
165.70
163.97
144.60
136.31
133.56
133.24
129.74
129.40
128.42
128.17
122.67
120.21
119.71
111.15
O
NH
O
O
N
CH3
NH
S
O
9000
8500
8000
7500
7000
6500
6000
5500
5000
4500
4000
3500
3000
2500
2000
1500
1000
500
0
-500
180
175
170
165
160
155
150
145
140
135
130
125
120
115
110
f1 (ppm)

1H NMR 12b

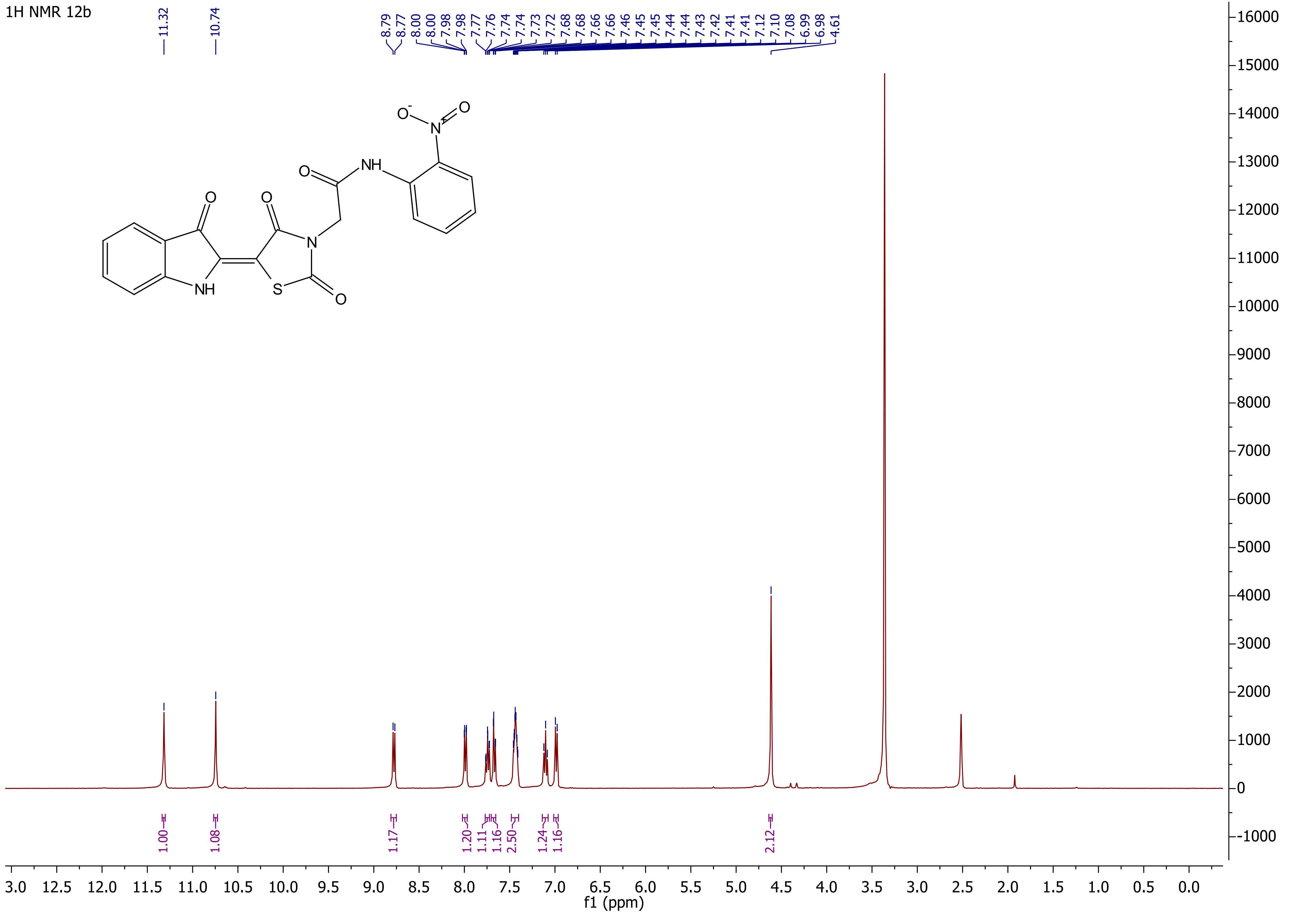

11.32
10.74
8.79
8.77
8.00
8.00
7.98
7.98
7.77
7.76
7.74
7.74
7.73
7.72
7.68
7.68
7.66
7.66
7.46
7.45
7.45
7.44
7.44
7.43
7.42
7.41
7.41
7.12
7.10
7.08
6.99
6.98
4.61
O
NH
O
O
N
S
O
NH
O
N
O
1.00
1.08
1.17
1.20
1.11
1.16
2.50
1.24
1.16
2.12
f1 (ppm)

1H NMR 12b

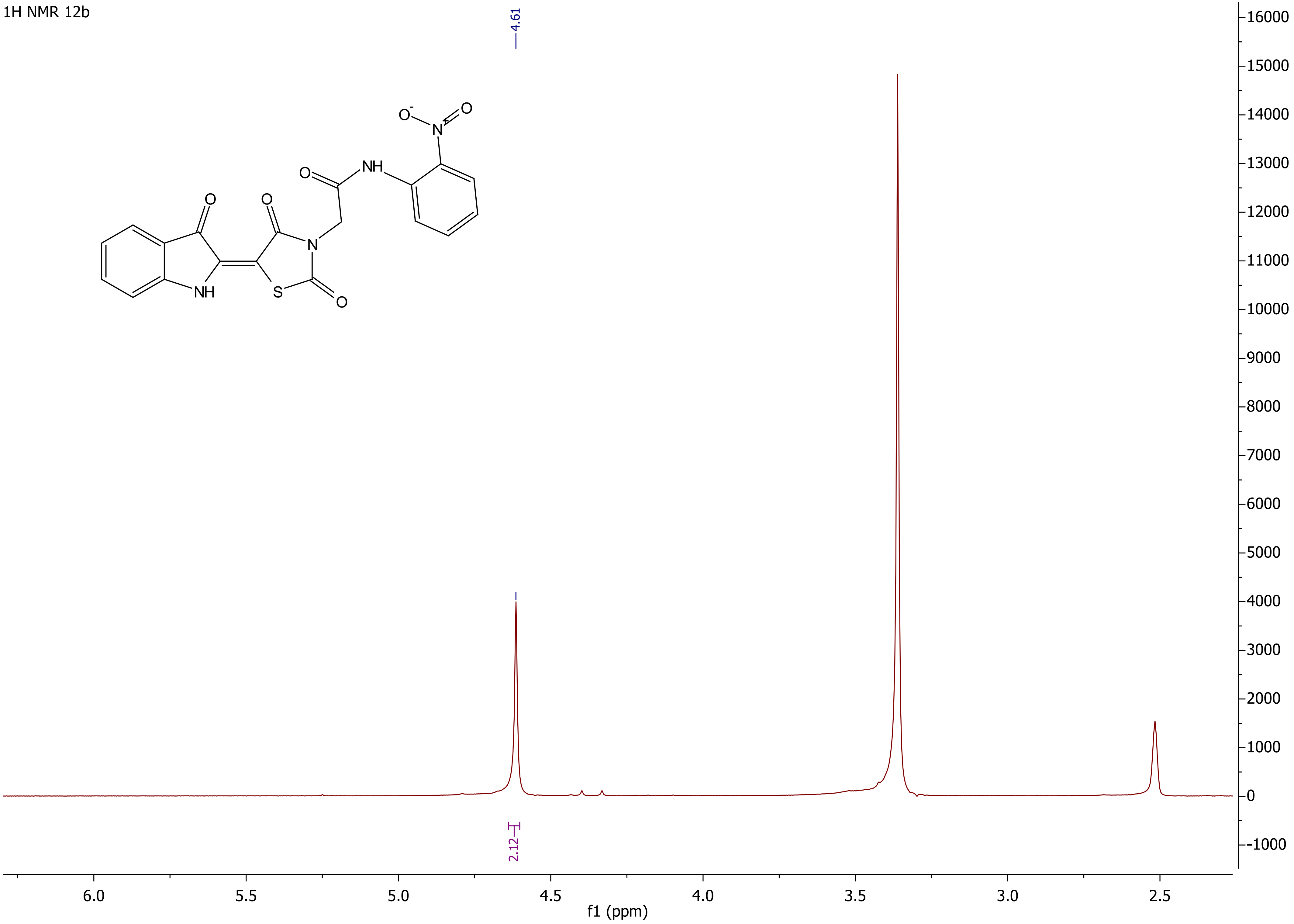
4.61
2.12
f1 (ppm)
6.0
5.5
5.0
4.5
4.0
3.5
3.0
2.5
16000
15000
14000
13000
12000
11000
10000
9000
8000
7000
6000
5000
4000
3000
2000
1000
0
-1000

1H NMR 12b

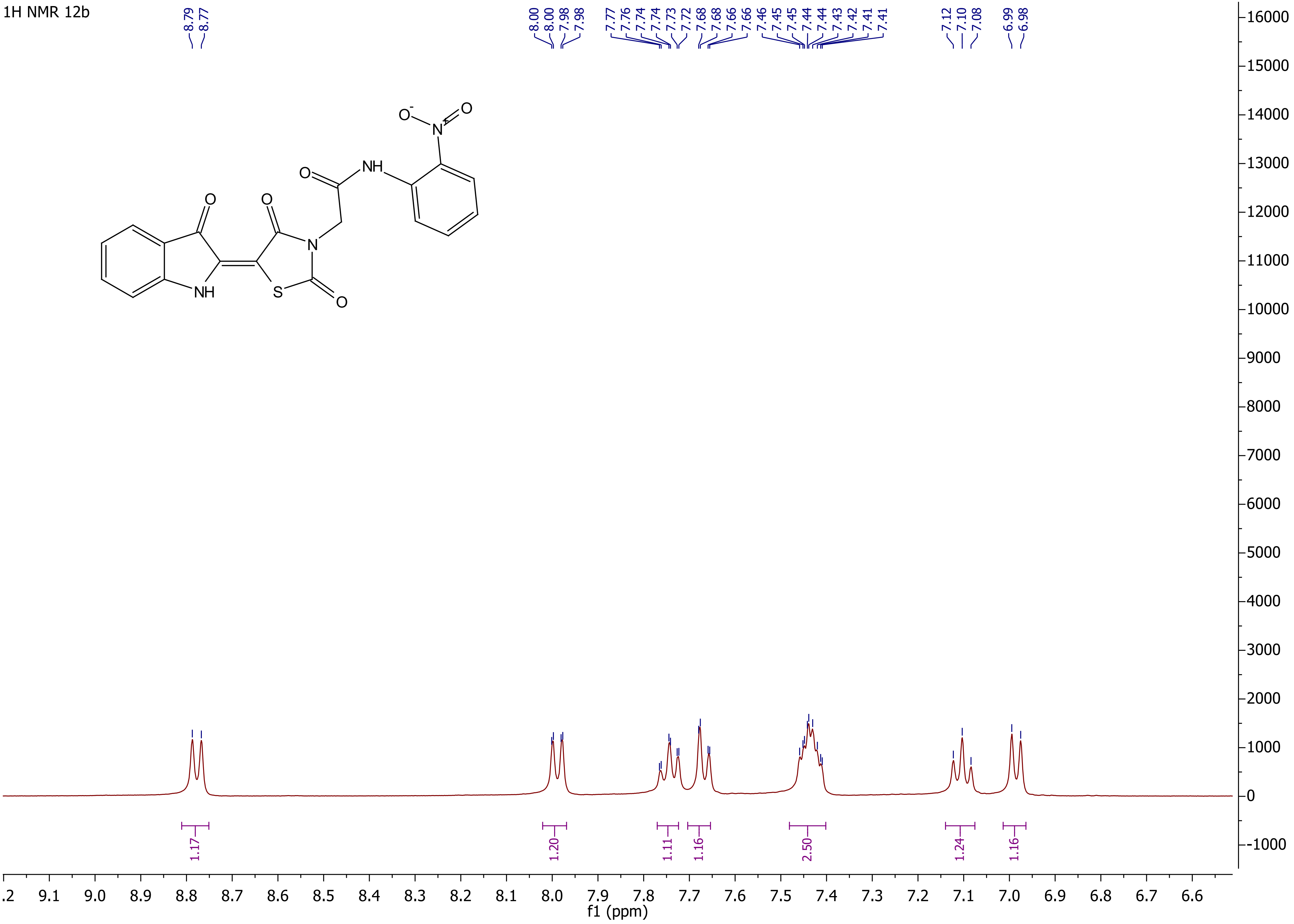

8.79
8.77
8.00
8.00
7.98
7.98
7.77
7.76
7.74
7.74
7.73
7.72
7.68
7.68
7.66
7.66
7.46
7.45
7.45
7.44
7.44
7.43
7.42
7.41
7.41
7.12
7.10
7.08
6.99
6.98
1.17
1.20
1.11
1.16
2.50
1.24
1.16
f1 (ppm)

1H NMR 12b

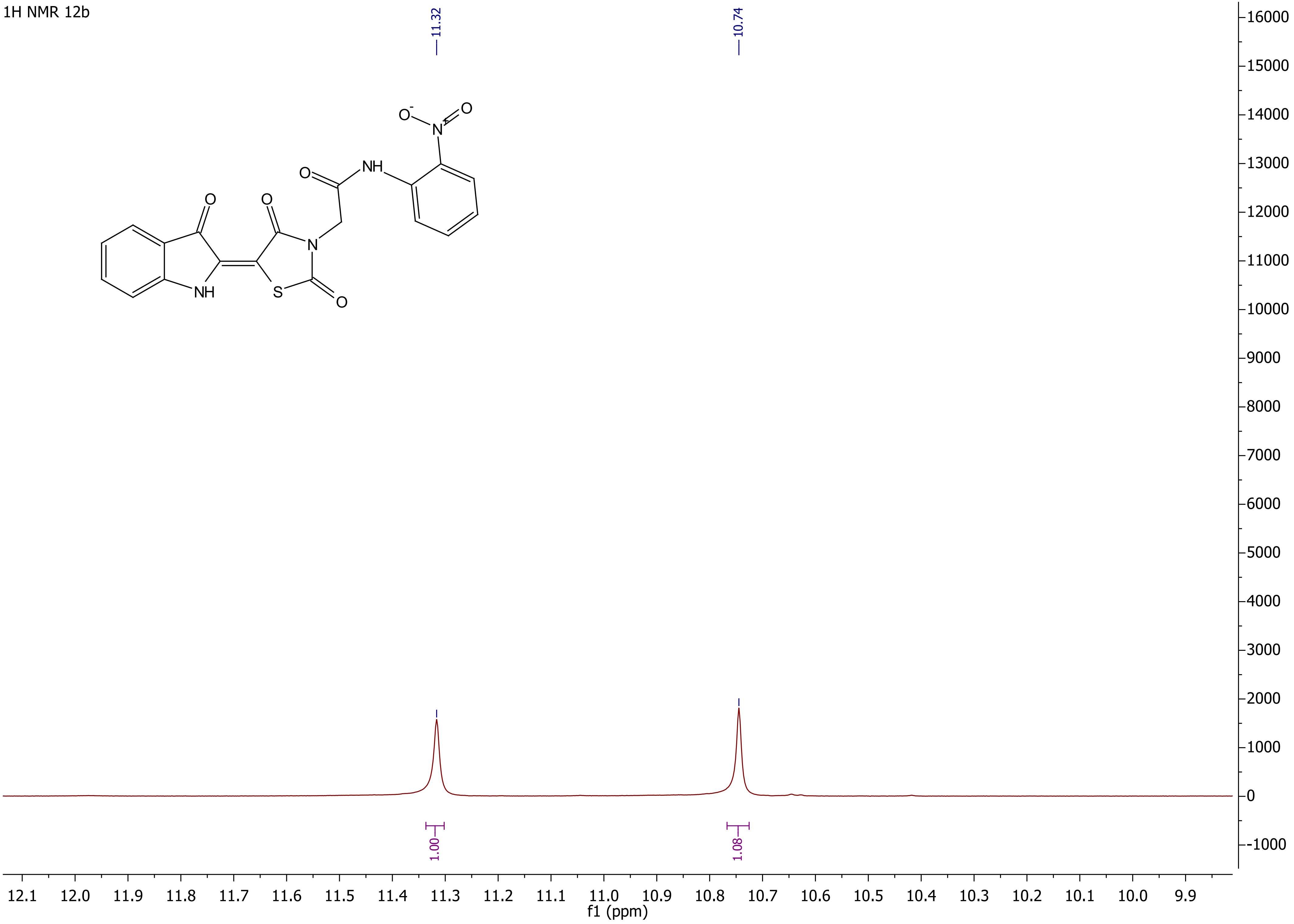

11.32
10.74
1.00
1.08
16000
15000
14000
13000
12000
11000
10000
9000
8000
7000
6000
5000
4000
3000
2000
1000
0
-1000
12.1 12.0 11.9 11.8 11.7 11.6 11.5 11.4 11.3 11.2 11.1 11.0 10.9 10.8 10.7 10.6 10.5 10.4 10.3 10.2 10.1 10.0 9.9
f1 (ppm)

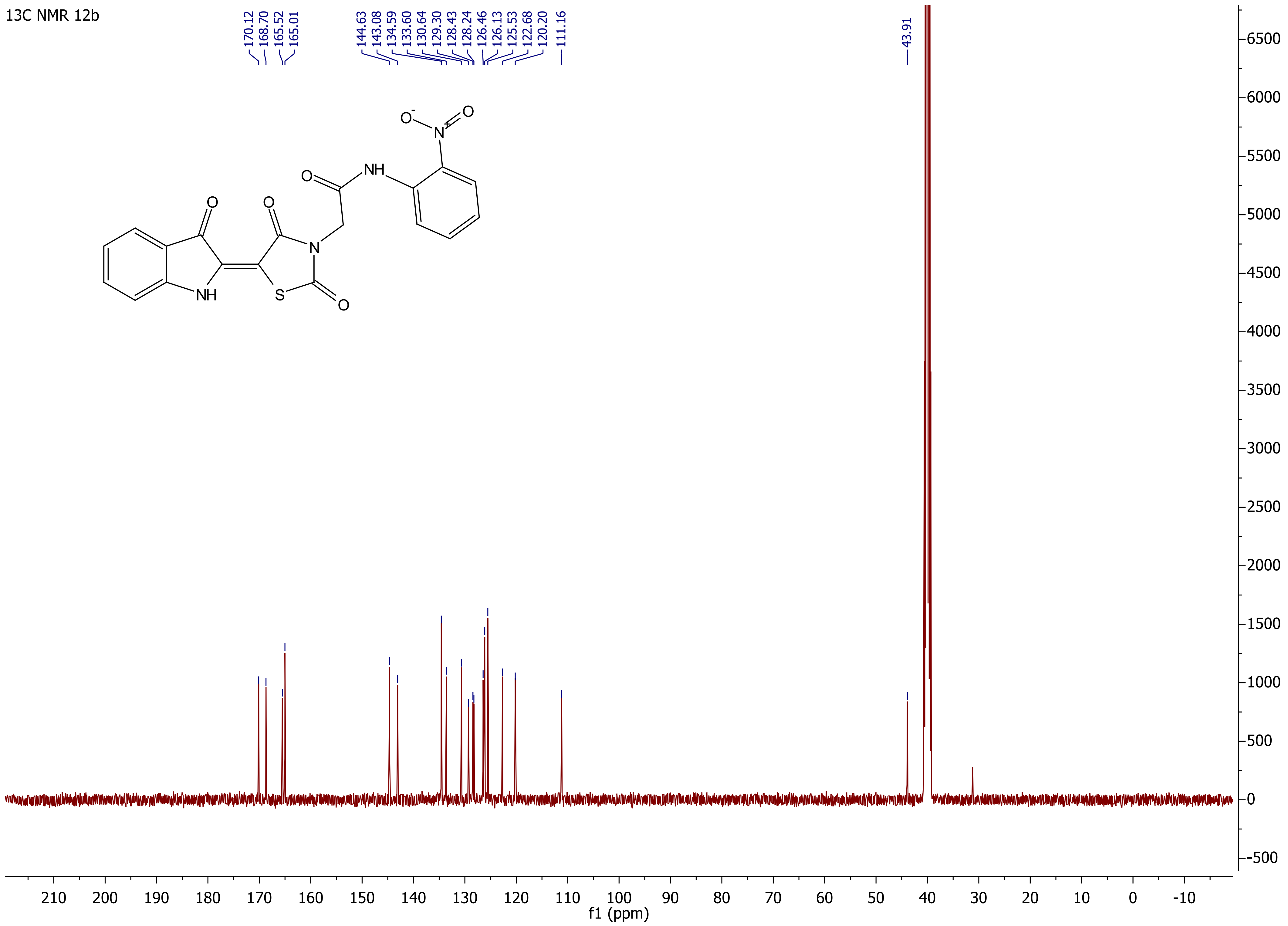
13C NMR 12b
170.12
168.70
165.52
165.01
144.63
143.08
134.59
133.60
130.64
129.30
128.43
128.24
126.46
126.13
125.53
122.68
120.20
111.16
43.91
f1 (ppm)

13C NMR 12b

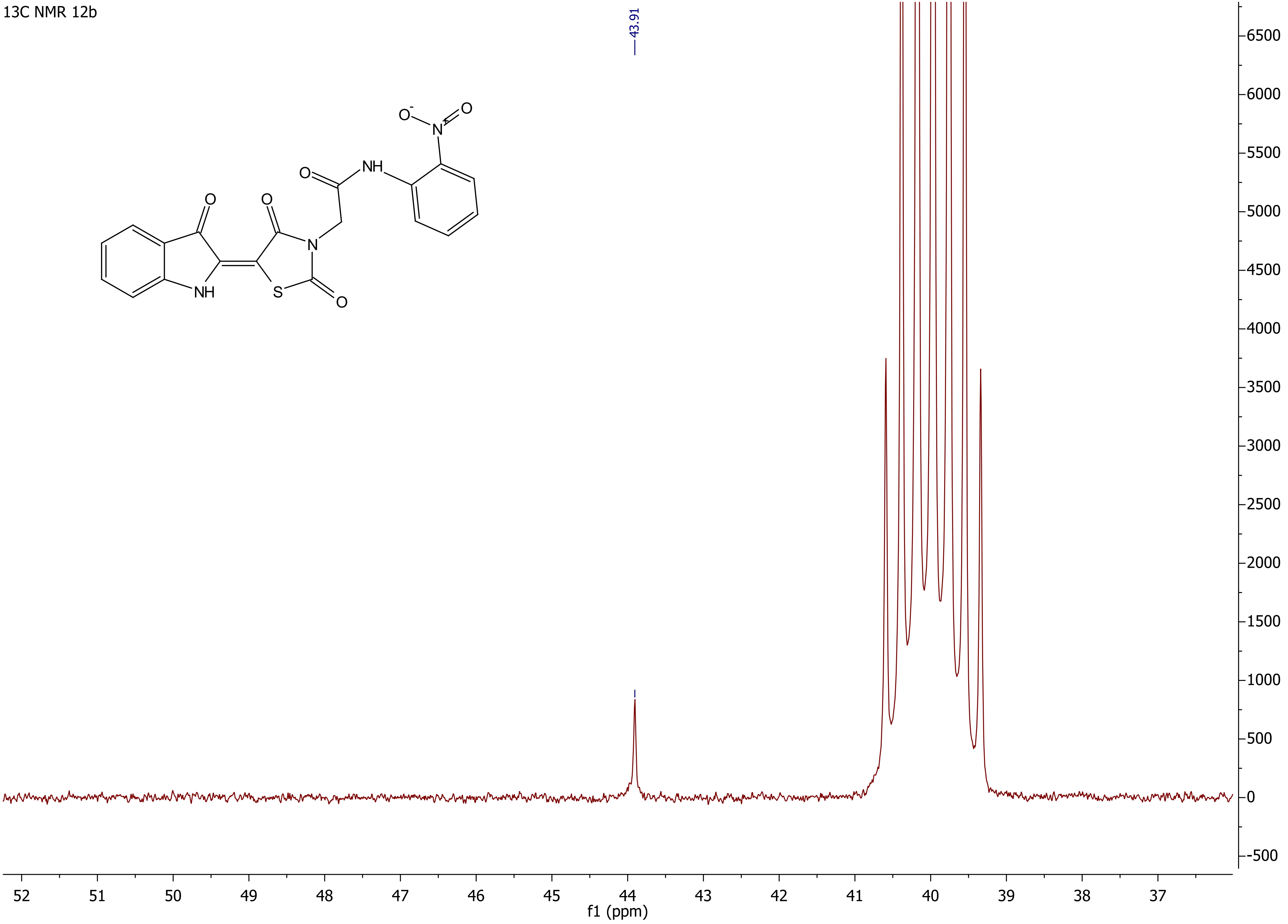

43.91
O
O
O
NH
N
S
O
NH
O
N
O
O
f1 (ppm)
52
51
50
49
48
47
46
45
44
43
42
41
40
39
38
37
6500
6000
5500
5000
4500
4000
3500
3000
2500
2000
1500
1000
500
0
-500

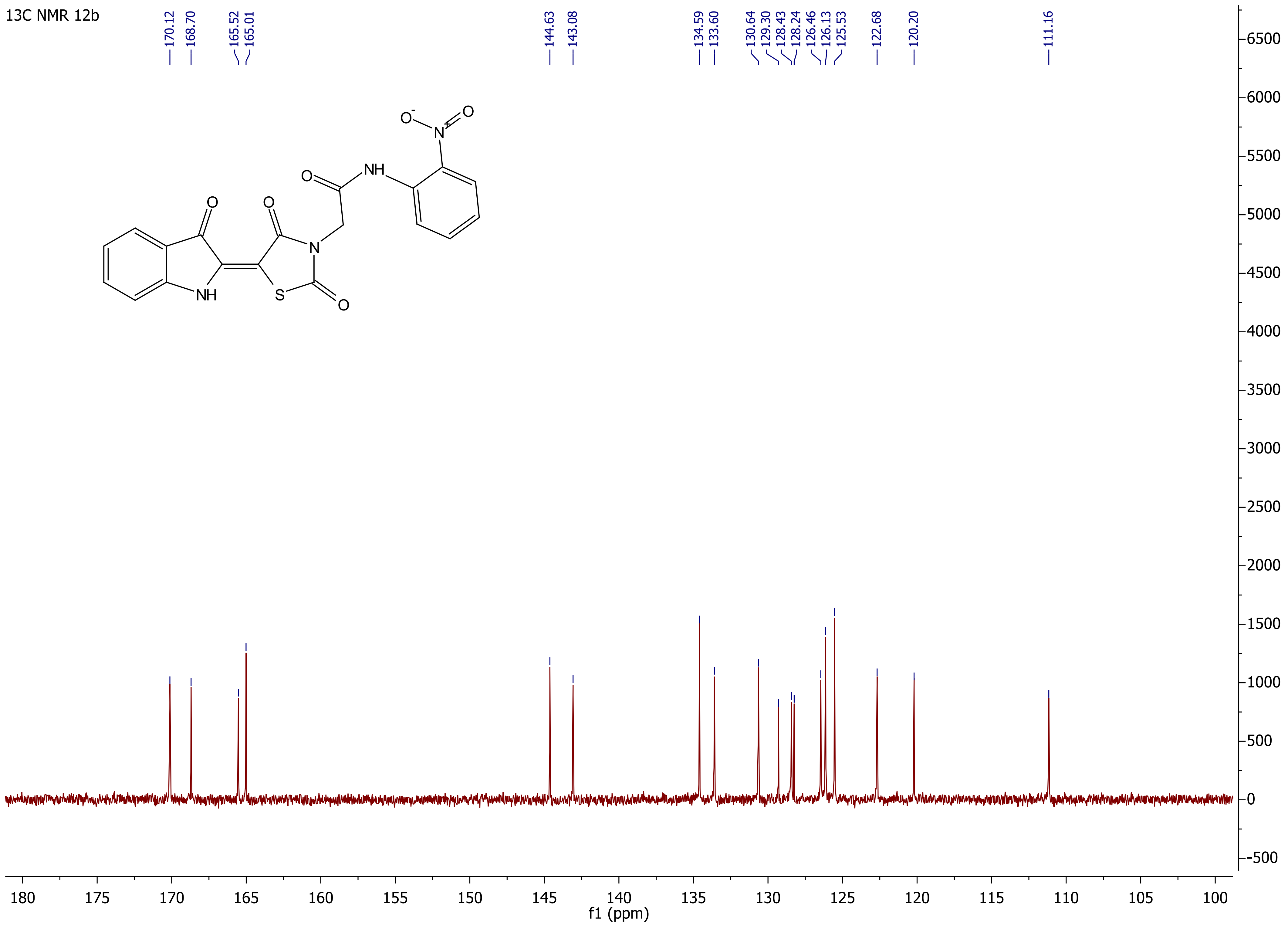
13C NMR 12b
170.12
168.70
165.52
165.01
144.63
143.08
134.59
133.60
130.64
129.30
128.43
128.24
126.46
126.13
125.53
122.68
120.20
111.16
O
NH
S
O
N
O
O
NH
O-
N+
O
f1 (ppm)

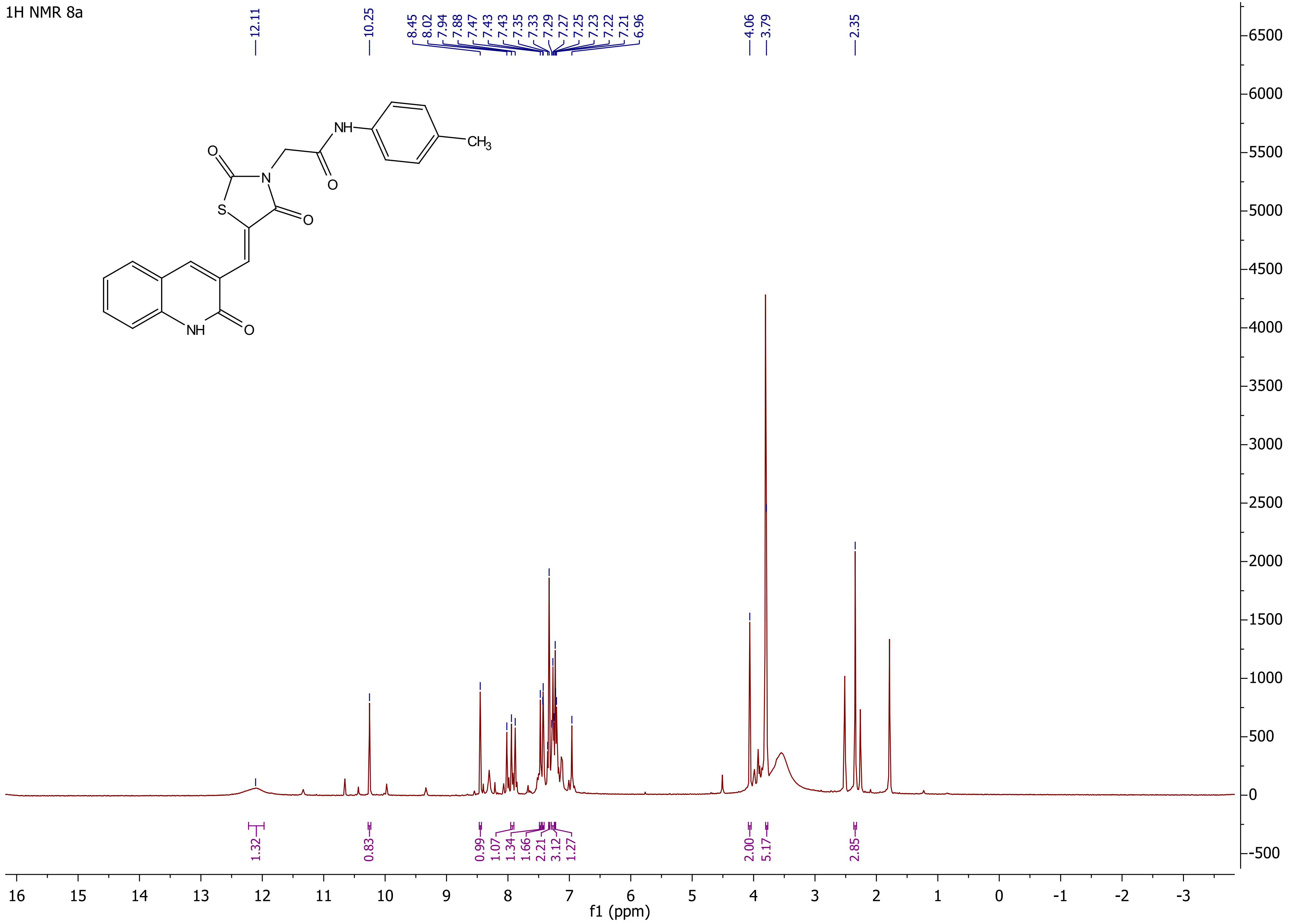

1H NMR 8a
12.11
10.25
8.45
8.02
7.94
7.88
7.47
7.43
7.43
7.35
7.33
7.29
7.27
7.25
7.23
7.22
7.21
6.96
4.06
3.79
2.35
NH
CH3
O
N
O
S
O
NH
O
1.32
0.83
0.99
1.07
1.34
1.66
2.21
3.12
1.27
2.00
5.17
2.85
f1 (ppm)

13C NMR 8a

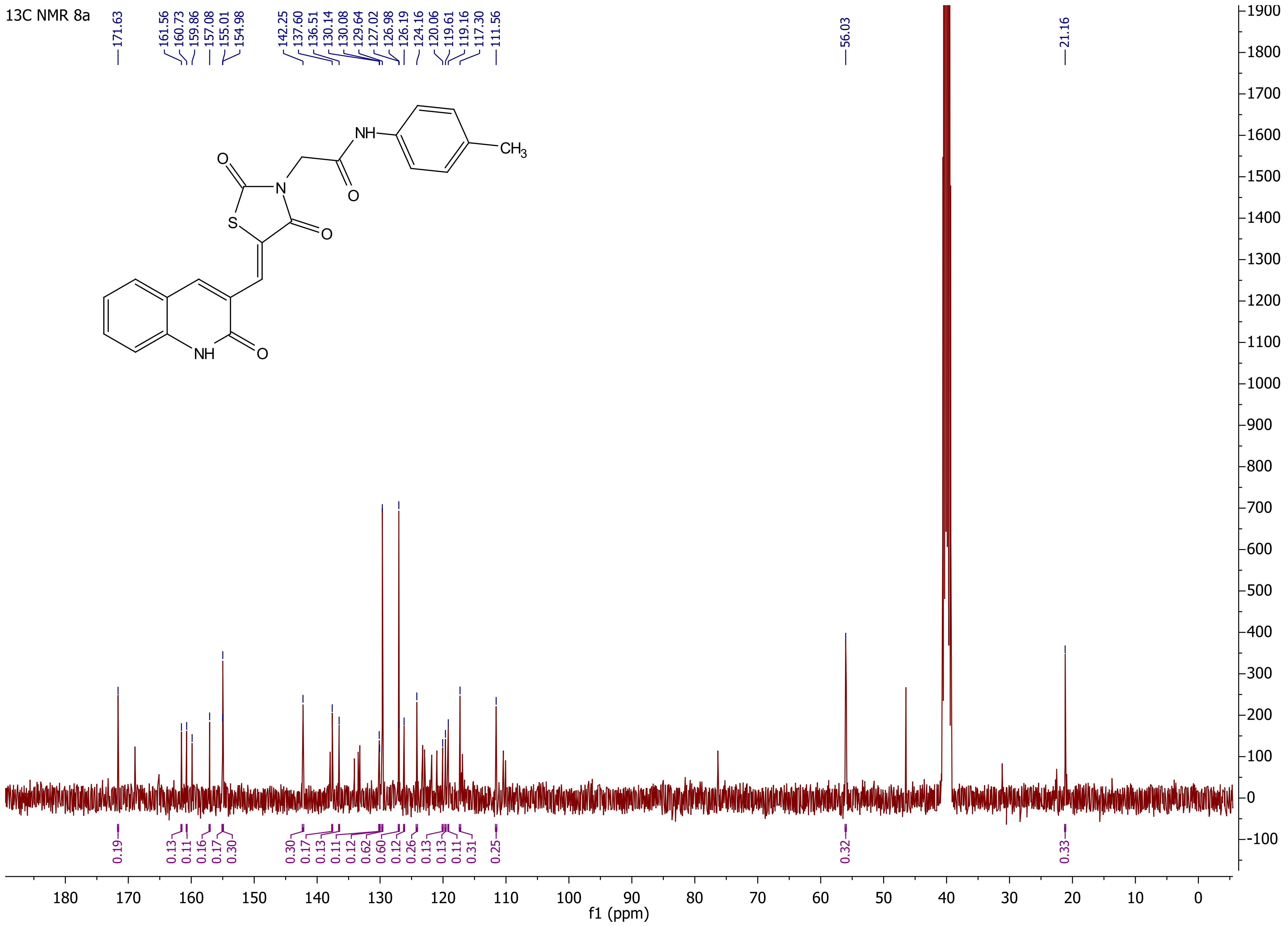
171.63
161.56
160.73
159.86
157.08
155.01
154.98
142.25
137.60
136.51
130.14
130.08
129.64
127.02
126.98
126.19
124.16
120.06
119.61
119.16
117.30
111.56
56.03
21.16
NH
CH3
O
N
S
O
O
NH
O
f1 (ppm)

1H NMR 8b

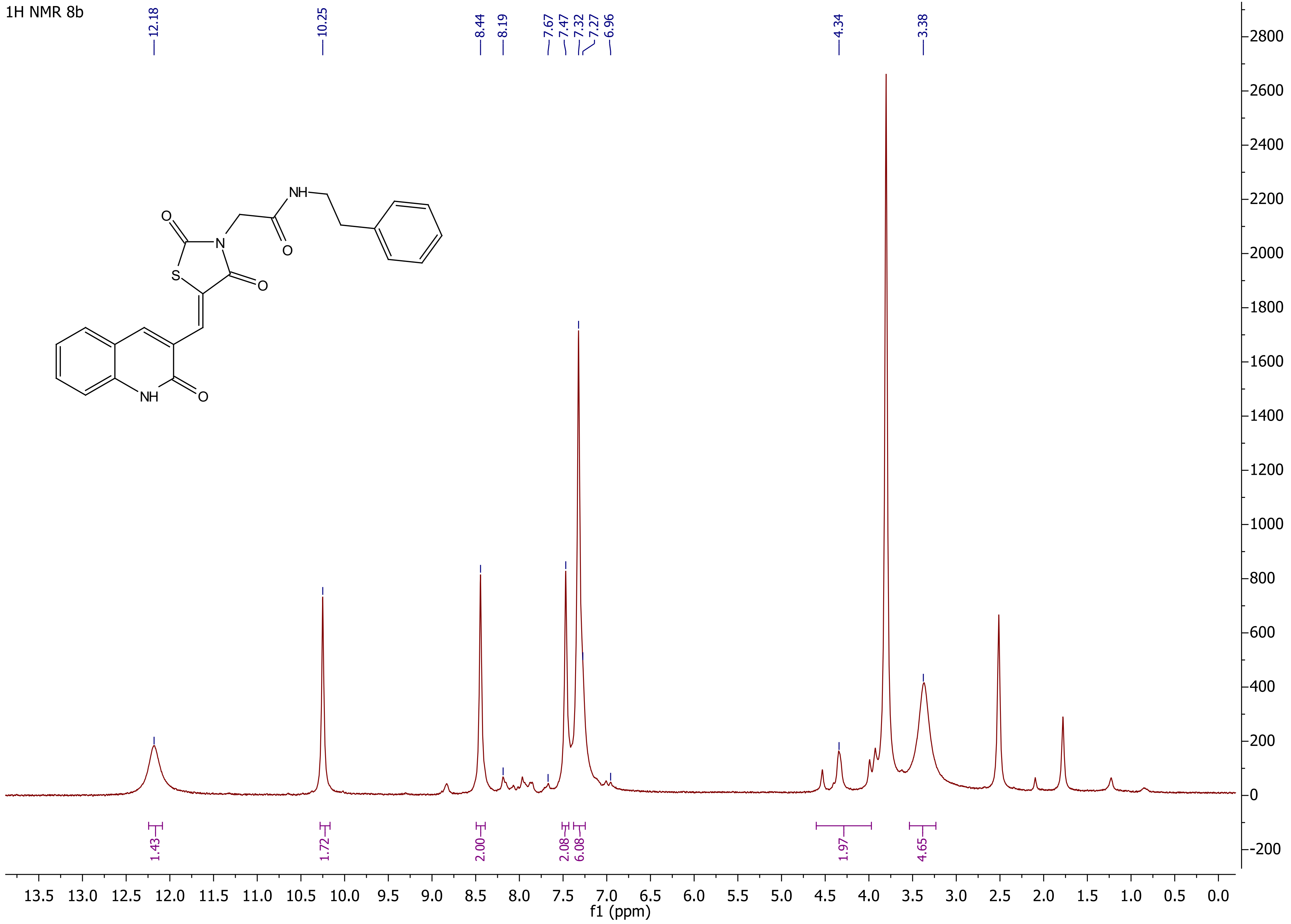
12.18
10.25
8.44
8.19
7.67
7.47
7.32
7.27
6.96
4.34
3.38
NH
O
N
O
S
O
NH
O
1.43
1.72
2.00
2.08
6.08
1.97
4.65
f1 (ppm)

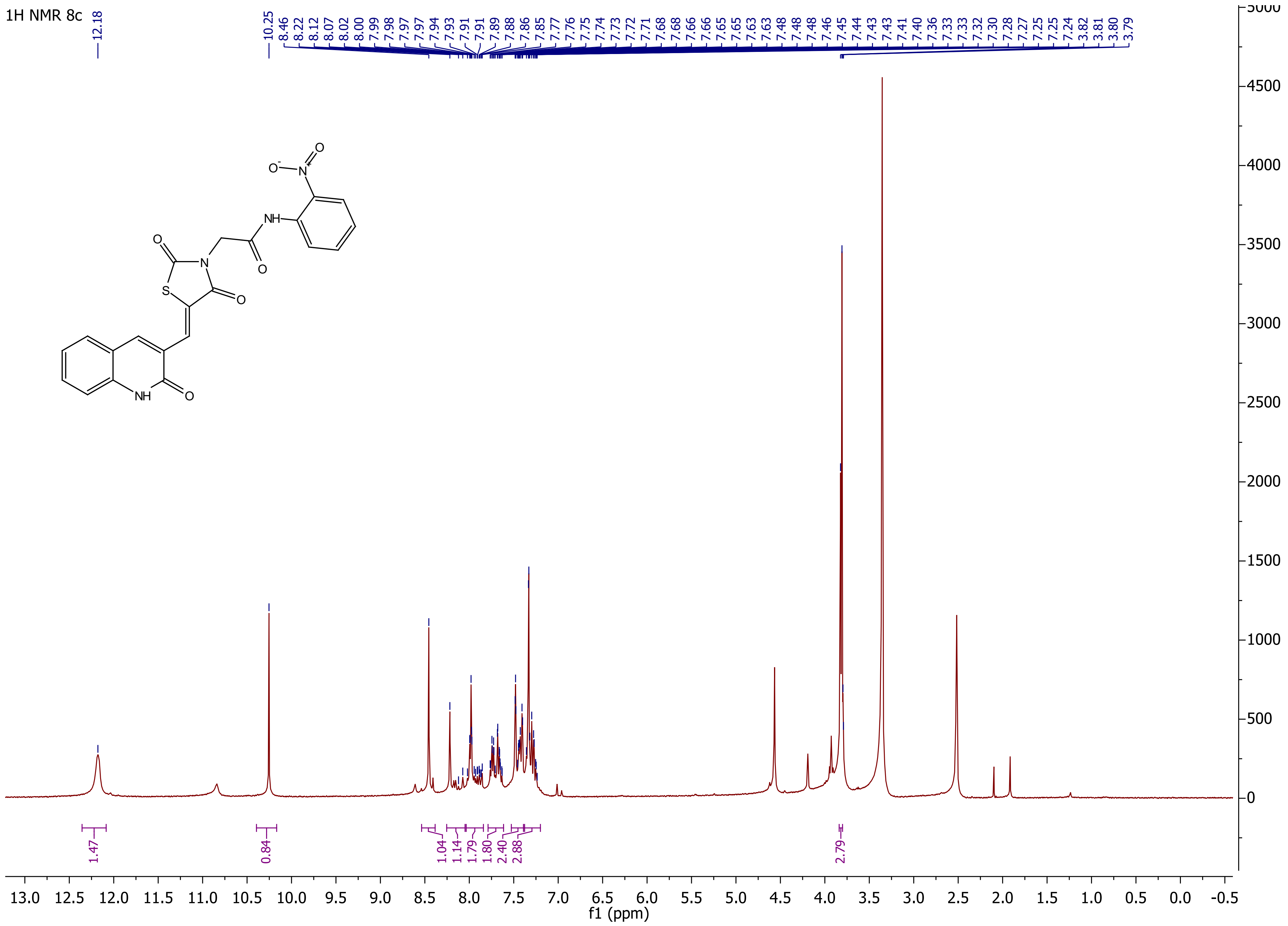
1H NMR 8c
12.18
10.25
8.46
8.22
8.12
8.07
8.02
8.00
7.99
7.98
7.97
7.97
7.94
7.93
7.91
7.91
7.89
7.88
7.86
7.85
7.77
7.76
7.75
7.74
7.73
7.72
7.71
7.68
7.68
7.66
7.66
7.65
7.65
7.63
7.63
7.48
7.48
7.48
7.46
7.45
7.44
7.43
7.43
7.41
7.40
7.36
7.33
7.33
7.32
7.30
7.28
7.27
7.25
7.25
7.24
3.82
3.81
3.80
3.79
O-
N+
O
NH
O
N
O
S
O
NH
O
1.47
0.84
1.04
1.14
1.79
1.80
2.40
2.88
2.79
f1 (ppm)

VEGFR-2 assay of compound 8a

Best-fit values	
LogIC50	1.985
HillSlope	-0.4030
IC50	96.64

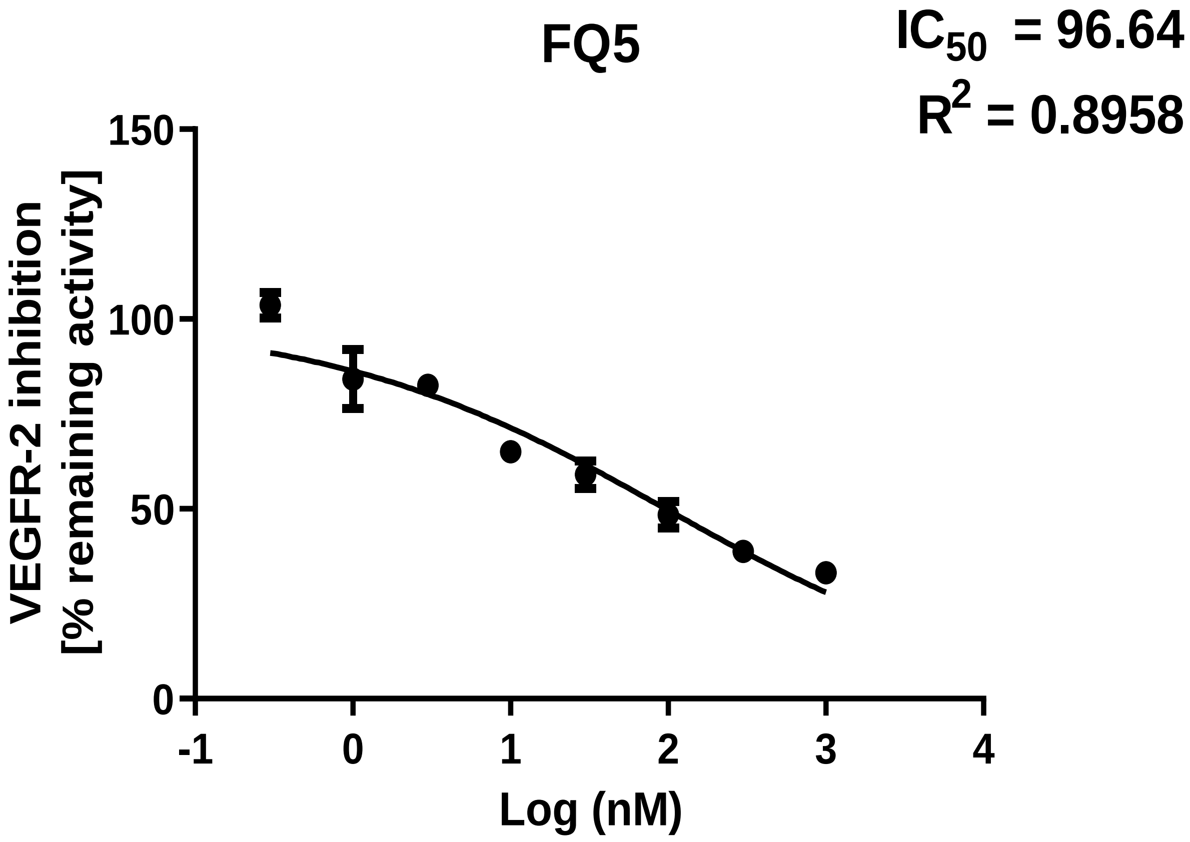

VEGFR-2 assay of compound 8b

Best-fit values	
LogIC50	1.941
HillSlope	-0.3502
IC50	87.37

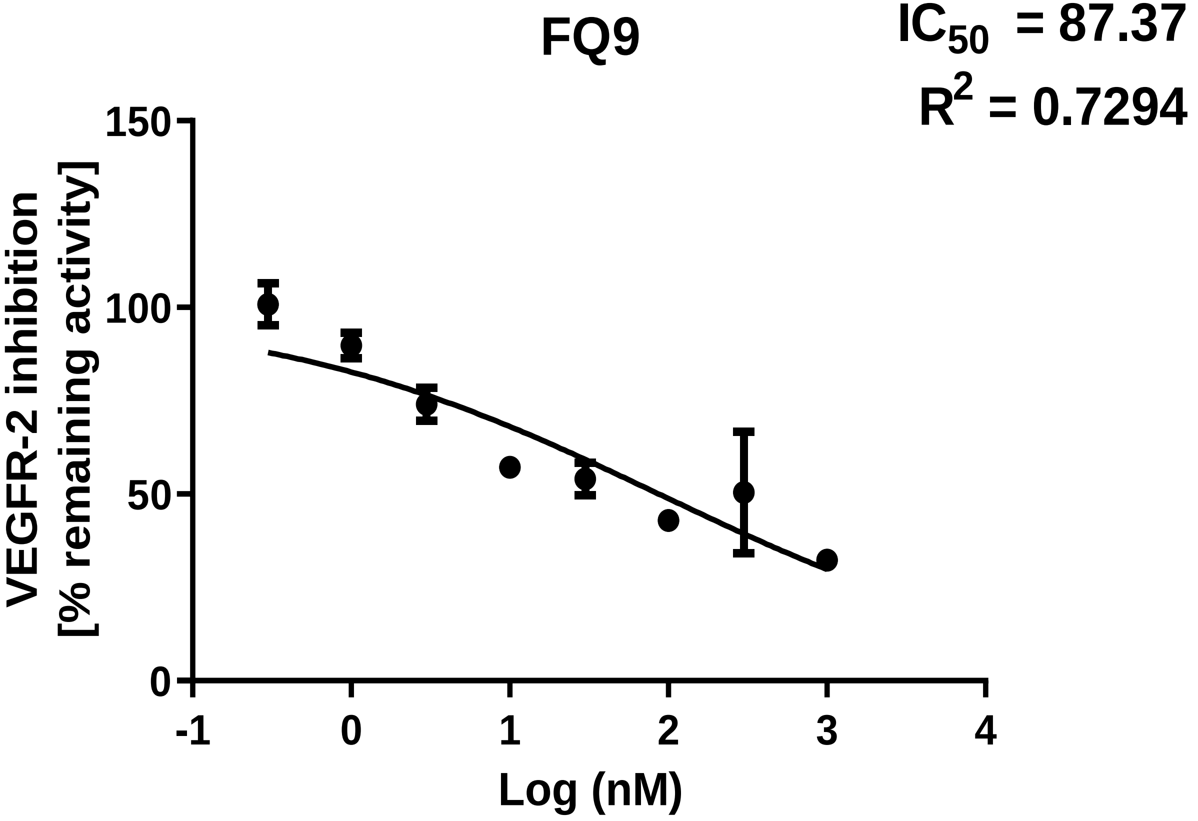

VEGFR-2 assay of compound 8c

Best-fit values	
LogIC50	2.502
HillSlope	-0.3460
IC50	317.7

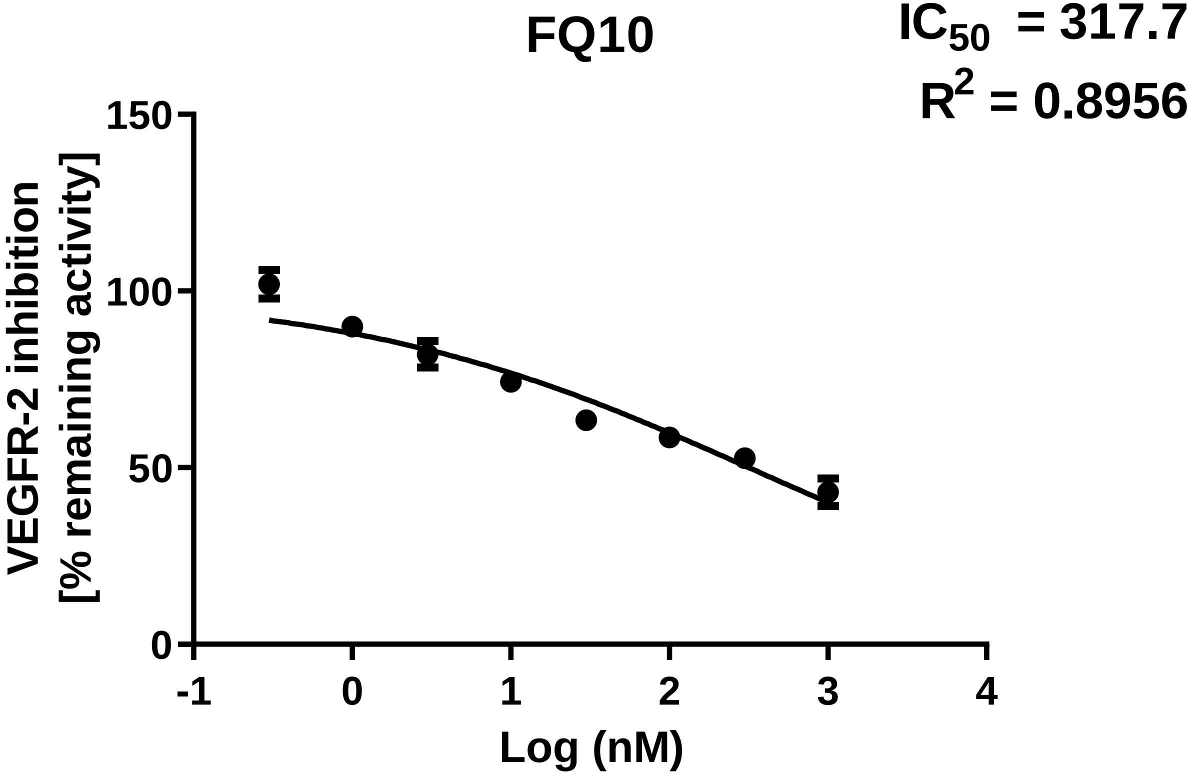

VEGFR-2 assay of compound12a

Best-fit values	
LogIC50	2.066
HillSlope	-0.4584
IC50	116.3

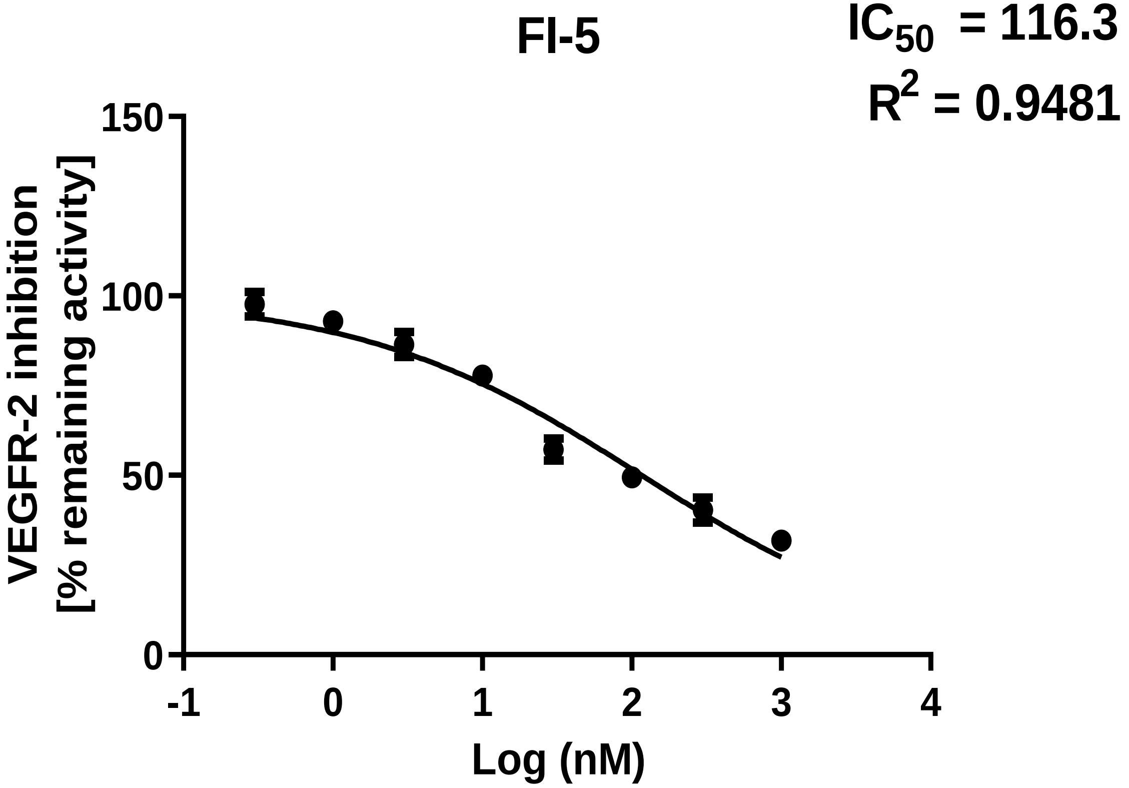

VEGFR-2 assay of compound 12b

Best-fit values	
LogIC50	1.925
HillSlope	-0.2985
IC50	84.05

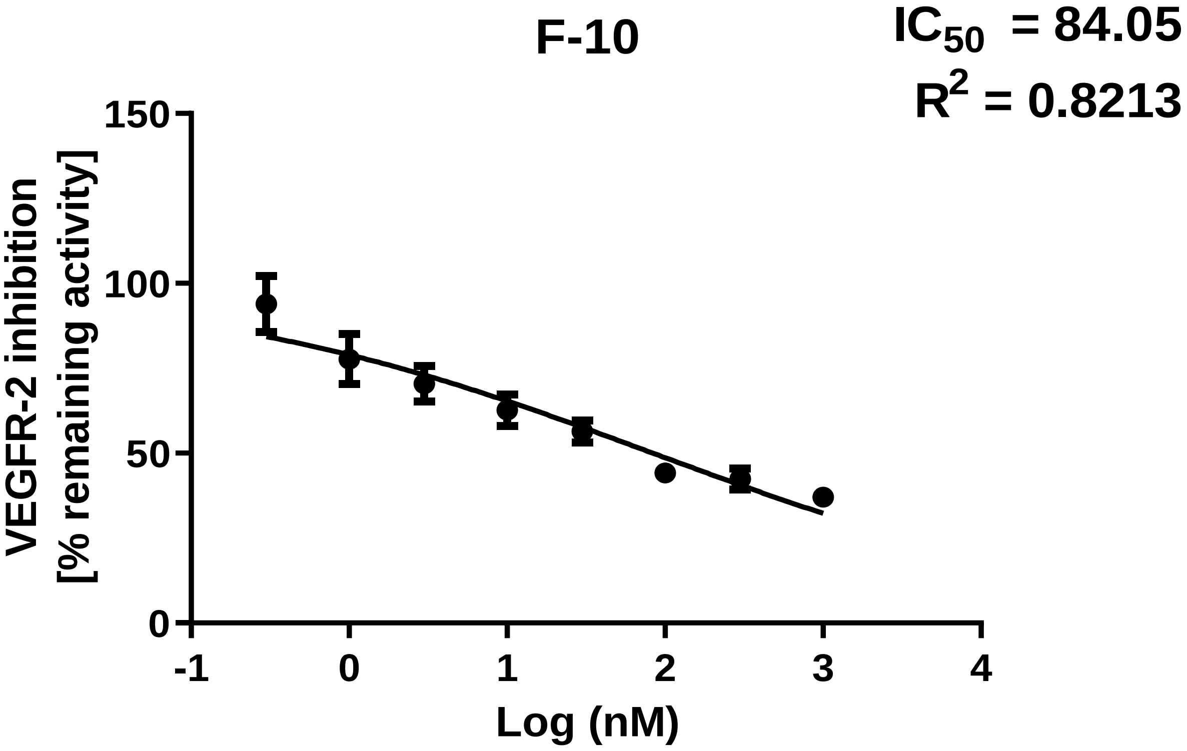

VEGFR-2 assay of Sorafenib

Best-fit values	
LogIC50	1.730
HillSlope	-0.4752
IC50	53.65

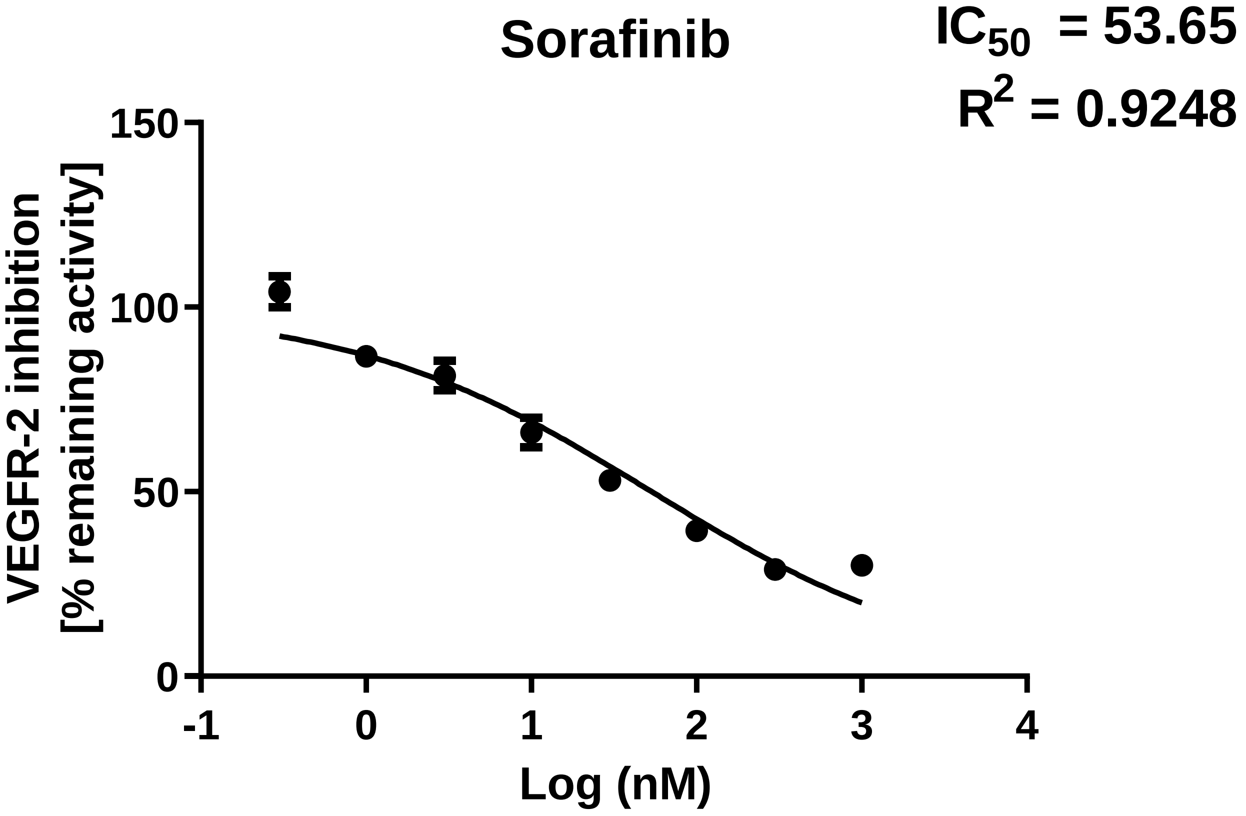

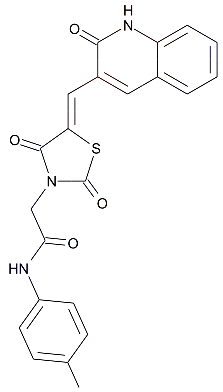

$C_{22}H_{17}N_3O_4S$
Molecular Weight: 419.45307
ALogP: 2.653
Rotatable Bonds: 4
Acceptors: 5
Donors: 2

Model Prediction

Prediction: Toxic
Probability: 0.548
Enrichment: 1.04
Bayesian Score: 0.0192
Mahalanobis Distance: 10.3
Mahalanobis Distance p-value: 0.00972

Prediction: Positive if the Bayesian score is above the estimated best cutoff value from minimizing the false positive and false negative rate.
Probability: The esimated probability that the sample is in the positive category. This assumes that the Bayesian score follows a normal distribution and is different from the prediction using a cutoff.
Enrichment: An estimate of enrichment, that is, the increased likelihood (versus random) of this sample being in the category.
Bayesian Score: The standard Laplacian-modified Bayesian score.
Mahalanobis Distance: The Mahalanobis distance (MD) is the distance to the center of the training data. The larger the MD, the less trustworthy the prediction.
Mahalanobis Distance p-value: The p-value gives the fraction of training data with an MD greater than or equal to the one for the given sample, assuming normally distributed data. The smaller the p-value, the less trustworthy the prediciton. For highly non-normal X properties (e.g., fingerprints), the MD p-value is wildly inaccurate.

Structural Similar Compounds

Name	Sulfonylurea Gliclazide	Amsacrine	D&C Yellow 8
Structure	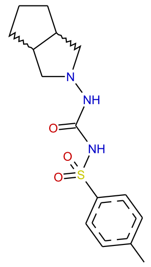	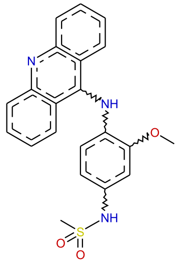	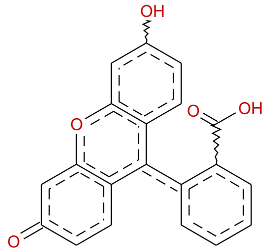
Actual Endpoint	Toxic	Toxic	Non-Toxic
Predicted Endpoint	Toxic	Toxic	Non-Toxic
Distance	0.611	0.619	0.620
Reference	Yakuri to Chiryo 9:3551-3571; 1981	Fundam Appl Toxicol 7(2):214-20; 1986	Food Chem Toxicol 24:819-823; 1986

Model Applicability

Unknown features are fingerprint features in the query molecule, but not found or appearing too infrequently in the training set.

1. All properties and OPS components are within expected ranges.

Feature Contribution

Top features for positive contribution				
**Fingerprint**	**Bit/Smiles**	**Feature Structure**	**Score**	**Toxic in training set**
SCFP_6	-1971137145	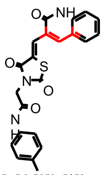 [*]C(=C[c](:[*]):[*])[*]	0.431	7 out of 8

SCFP_6	-1971196727	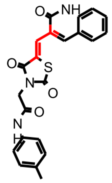 [*]C(=CC(=[*])[*])[*]	0.293	13 out of 18
SCFP_6	853077039	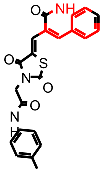 [*]C1=C[c]2:[cH]:[cH]:[*]:[cH]:[c]:2N[*]1	0.271	1 out of 1

Top Features for negative contribution

**Fingerprint**	**Bit/Smiles**	**Feature Structure**	**Score**	**Toxic in training set**
SCFP_6	399659969	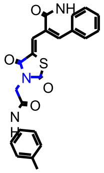 [*]CN1C(=[*])[*][*]C1=[*]	-0.526	3 out of 11
SCFP_6	2097618059	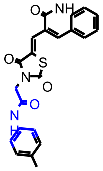 [*]CC(=O)N[c](:[cH]:[*]):[cH]:[*]	-0.422	0 out of 1
SCFP_6	1420330831	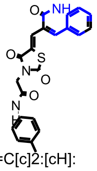 [*]=C1[*]=C[c]2:[cH]:[*]:[cH]:[cH]:[c]:2N1	-0.422	0 out of 1

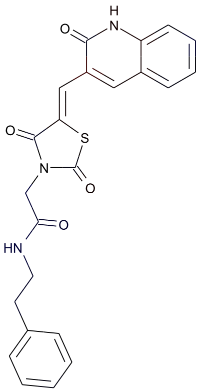

$C_{23}H_{19}N_3O_4S$
Molecular Weight: 433.47965
ALogP: 2.495
Rotatable Bonds: 6
Acceptors: 5
Donors: 2

Model Prediction

Prediction: Non-Toxic
Probability: 0.535
Enrichment: 1.02
Bayesian Score: -0.342
Mahalanobis Distance: 13.1
Mahalanobis Distance p-value: 3.11e-007

Prediction: Positive if the Bayesian score is above the estimated best cutoff value from minimizing the false positive and false negative rate.
Probability: The esimated probability that the sample is in the positive category. This assumes that the Bayesian score follows a normal distribution and is different from the prediction using a cutoff.
Enrichment: An estimate of enrichment, that is, the increased likelihood (versus random) of this sample being in the category.
Bayesian Score: The standard Laplacian-modified Bayesian score.
Mahalanobis Distance: The Mahalanobis distance (MD) is the distance to the center of the training data. The larger the MD, the less trustworthy the prediction.
Mahalanobis Distance p-value: The p-value gives the fraction of training data with an MD greater than or equal to the one for the given sample, assuming normally distributed data. The smaller the p-value, the less trustworthy the prediciton. For highly non-normal X properties (e.g., fingerprints), the MD p-value is wildly inaccurate.

Structural Similar Compounds

Name	Ochratoxin a	Amsacrine	Clebopride Malate
Structure	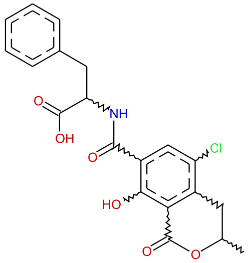	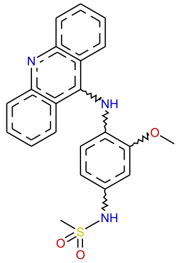	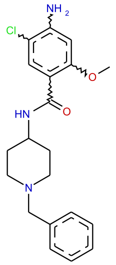
Actual Endpoint	Toxic	Toxic	Non-Toxic
Predicted Endpoint	Toxic	Toxic	Non-Toxic
Distance	0.603	0.628	0.635
Reference	Toxicol Appl Pharmacol 37(2):331-8; 1976	Fundam Appl Toxicol 7(2):214-20; 1986	Kiso to Rinsho 16:5649-5660; 1982

Model Applicability

Unknown features are fingerprint features in the query molecule, but not found or appearing too infrequently in the training set.

1. All properties and OPS components are within expected ranges.

Feature Contribution

Top features for positive contribution				
Fingerprint	Bit/Smiles	Feature Structure	Score	Toxic in training set
SCFP_6	-1971137145	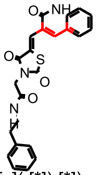 [*]C(=C[c](:[*]):[*])[*]	0.431	7 out of 8

SCFP_6	-1971196727	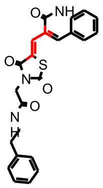<br>[*]C(=CC(=[*])[*])[*]	0.293	13 out of 18
SCFP_6	199205675	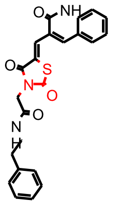<br>[*]N1[*][*]SC1=O	0.271	1 out of 1

Top Features for negative contribution

Fingerprint	Bit/Smiles	Feature Structure	Score	Toxic in training set
SCFP_6	2005026407	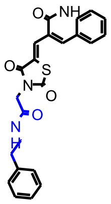<br>[*]CCNC(=O)C[*]	-0.718	0 out of 2
SCFP_6	399659969	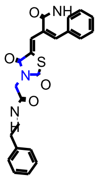<br>[*]CN1C(=[*])[*][*]C1<br>=[*]	-0.526	3 out of 11
SCFP_6	1420330831	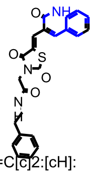<br>[*]=C1[*]=C[c]2:[cH]:<br>[*]:[cH]:[cH]:[c]:2N<br>1	-0.422	0 out of 1

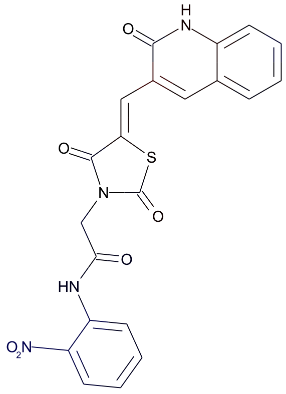

$C_{21}H_{14}N_4O_6S$
Molecular Weight: 450.42405
ALogP: 2.061
Rotatable Bonds: 5
Acceptors: 7
Donors: 2

Model Prediction

Prediction: Non-Toxic
Probability: 0.508
Enrichment: 0.967
Bayesian Score: -1.06
Mahalanobis Distance: 9.97
Mahalanobis Distance p-value: 0.0227

Prediction: Positive if the Bayesian score is above the estimated best cutoff value from minimizing the false positive and false negative rate.
Probability: The esimated probability that the sample is in the positive category. This assumes that the Bayesian score follows a normal distribution and is different from the prediction using a cutoff.
Enrichment: An estimate of enrichment, that is, the increased likelihood (versus random) of this sample being in the category.
Bayesian Score: The standard Laplacian-modified Bayesian score.
Mahalanobis Distance: The Mahalanobis distance (MD) is the distance to the center of the training data. The larger the MD, the less trustworthy the prediction.
Mahalanobis Distance p-value: The p-value gives the fraction of training data with an MD greater than or equal to the one for the given sample, assuming normally distributed data. The smaller the p-value, the less trustworthy the prediciton. For highly non-normal X properties (e.g., fingerprints), the MD p-value is wildly inaccurate.

Structural Similar Compounds

Name	Lenampicillin .HCl (Free base form)	Azosemide	Ochratoxin a
Structure	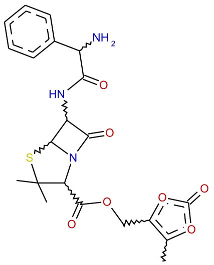	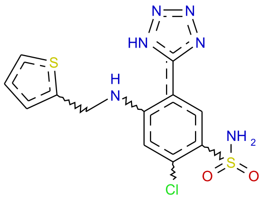	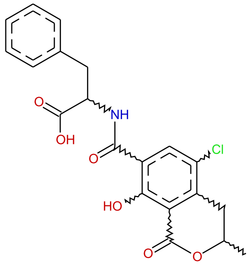
Actual Endpoint	Non-Toxic	Non-Toxic	Toxic
Predicted Endpoint	Non-Toxic	Non-Toxic	Toxic
Distance	0.680	0.695	0.702
Reference	Chemotherapy 32:130-145; 1984	Kiso to Rinsho 18:5187-5195; 1984	Toxicol Appl Pharmacol 37(2):331-8; 1976

Model Applicability

Unknown features are fingerprint features in the query molecule, but not found or appearing too infrequently in the training set.

1. All properties and OPS components are within expected ranges.

Feature Contribution

Top features for positive contribution				
**Fingerprint**	**Bit/Smiles**	**Feature Structure**	**Score**	**Toxic in training set**
SCFP_6	-1971137145	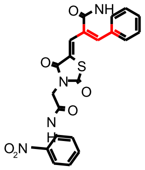 [*]C(=C[c](:[*]):[*])[*]	0.431	7 out of 8

SCFP_6	-1971196727	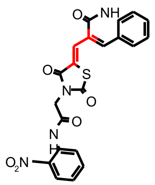 [*]C(=CC(=[*])[*])[*]	0.293	13 out of 18
SCFP_6	1730407098	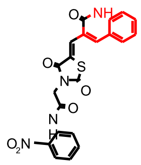 [*]C1=C[c]2:[cH]:[cH]:[cH]:[cH]:[c]:2N[*]1	0.271	1 out of 1
**Top Features for negative contribution**				
**Fingerprint**	**Bit/Smiles**	**Feature Structure**	**Score**	**Toxic in training set**
SCFP_6	399659969	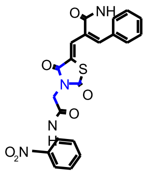 [*]CN1C(=[*])[*][*]C1=[*]	-0.526	3 out of 11
SCFP_6	-1380909229	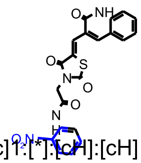 [*][c]1:[*]:[cH]:[cH]:[cH]:[c]:1[N+](=[*])[*]	-0.449	6 out of 19
SCFP_6	1311339974	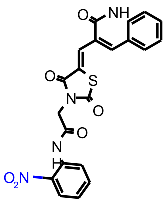 [*][N+](=O)[*]	-0.446	3 out of 10

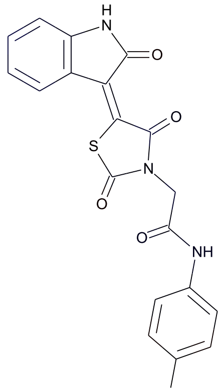

$C_{20}H_{15}N_3O_4S$
Molecular Weight: 393.4158
ALogP: 2.185
Rotatable Bonds: 3
Acceptors: 5
Donors: 2

Structural Similar Compounds

Name	Sulfonylurea Gliclazide	D&C Yellow 8	Piroxicam
Structure	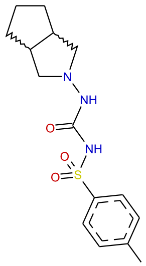	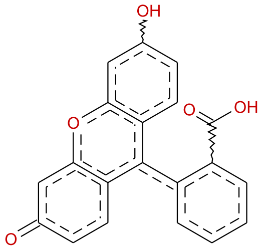	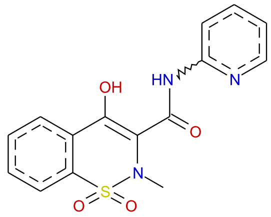
Actual Endpoint	Toxic	Non-Toxic	Toxic
Predicted Endpoint	Toxic	Non-Toxic	Toxic
Distance	0.568	0.602	0.623
Reference	Yakuri to Chiryo 9:3551-3571; 1981	Food Chem Toxicol 24:819-823; 1986	Yakuri to Chiryo 8:4655-4671; 1980

Model Prediction

Prediction: Non-Toxic
Probability: 0.503
Enrichment: 0.956
Bayesian Score: -1.21
Mahalanobis Distance: 8.48
Mahalanobis Distance p-value: 0.375

Prediction: Positive if the Bayesian score is above the estimated best cutoff value from minimizing the false positive and false negative rate.
Probability: The esimated probability that the sample is in the positive category. This assumes that the Bayesian score follows a normal distribution and is different from the prediction using a cutoff.
Enrichment: An estimate of enrichment, that is, the increased likelihood (versus random) of this sample being in the category.
Bayesian Score: The standard Laplacian-modified Bayesian score.
Mahalanobis Distance: The Mahalanobis distance (MD) is the distance to the center of the training data. The larger the MD, the less trustworthy the prediction.
Mahalanobis Distance p-value: The p-value gives the fraction of training data with an MD greater than or equal to the one for the given sample, assuming normally distributed data. The smaller the p-value, the less trustworthy the prediciton. For highly non-normal X properties (e.g., fingerprints), the MD p-value is wildly inaccurate.

Model Applicability

Unknown features are fingerprint features in the query molecule, but not found or appearing too infrequently in the training set.

1. OPS PC14 out of range. Value: 4.1216. Training min, max, SD, explained variance: -3.5766, 3.955, 1.214, 0.0216.

Feature Contribution

Top features for positive contribution

Fingerprint	Bit/Smiles	Feature Structure	Score	Toxic in training set
SCFP_6	-587539325	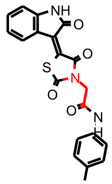 [*]N([*])CC(=[*])[*]	0.271	1 out of 1

SCFP_6	795925860	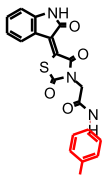 [*][c]1:[cH]:[cH]:[c](C):[cH]:[cH]:1	0.271	1 out of 1
SCFP_6	199205675	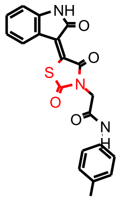 [*]N1[*][*]SC1=O	0.271	1 out of 1

Top Features for negative contribution

Fingerprint	Bit/Smiles	Feature Structure	Score	Toxic in training set
SCFP_6	399659969	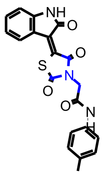 [*]CN1C(=[*])[*][*]C1=[*]	-0.526	3 out of 11
SCFP_6	2097618059	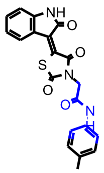 [*]CC(=O)N[c](:[cH]:[*]):[cH]:[*]	-0.422	0 out of 1
SCFP_6	1420330831	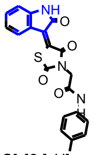 [*]=C1[*]=C[c]2:[cH]:[*]:[cH]:[cH]:[c]:2N1	-0.422	0 out of 1

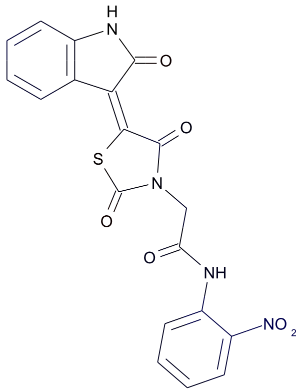

$C_{19}H_{12}N_4O_6S$
Molecular Weight: 424.38678
ALogP: 1.593
Rotatable Bonds: 4
Acceptors: 7
Donors: 2

Model Prediction

Prediction: Non-Toxic
Probability: 0.468
Enrichment: 0.891
Bayesian Score: -2.21
Mahalanobis Distance: 8.11
Mahalanobis Distance p-value: 0.554

Prediction: Positive if the Bayesian score is above the estimated best cutoff value from minimizing the false positive and false negative rate.
Probability: The esimated probability that the sample is in the positive category. This assumes that the Bayesian score follows a normal distribution and is different from the prediction using a cutoff.
Enrichment: An estimate of enrichment, that is, the increased likelihood (versus random) of this sample being in the category.
Bayesian Score: The standard Laplacian-modified Bayesian score.
Mahalanobis Distance: The Mahalanobis distance (MD) is the distance to the center of the training data. The larger the MD, the less trustworthy the prediction.
Mahalanobis Distance p-value: The p-value gives the fraction of training data with an MD greater than or equal to the one for the given sample, assuming normally distributed data. The smaller the p-value, the less trustworthy the prediciton. For highly non-normal X properties (e.g., fingerprints), the MD p-value is wildly inaccurate.

Structural Similar Compounds

Name	Azosemide	Piroxicam	Lenampicillin .HCl (Free base form)
Structure	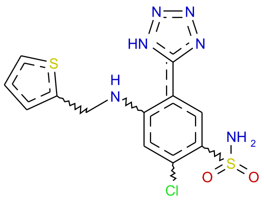	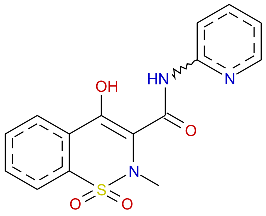	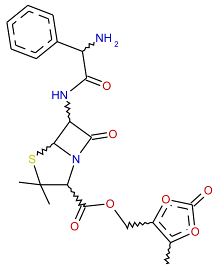
Actual Endpoint	Non-Toxic	Toxic	Non-Toxic
Predicted Endpoint	Non-Toxic	Toxic	Non-Toxic
Distance	0.657	0.703	0.714
Reference	Kiso to Rinsho 18:5187-5195; 1984	Yakuri to Chiryo 8:4655-4671; 1980	Chemotherapy 32:130-145; 1984

Model Applicability

Unknown features are fingerprint features in the query molecule, but not found or appearing too infrequently in the training set.

1. All properties and OPS components are within expected ranges.

Feature Contribution

Top features for positive contribution

Fingerprint	Bit/Smiles	Feature Structure	Score	Toxic in training set
SCFP_6	-587539325	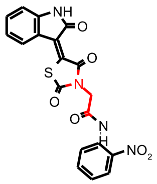 [*]N([*])CC(=[*])[*]	0.271	1 out of 1

SCFP_6	2102703671	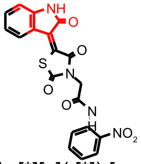 [*]C1=[*][c](:[*]):[c](NC1=O):[cH]:[*]	0.271	1 out of 1
SCFP_6	199205675	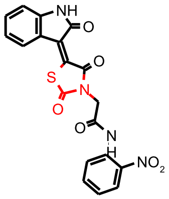 [*]N1[*][*]SC1=O	0.271	1 out of 1

Top Features for negative contribution

Fingerprint	Bit/Smiles	Feature Structure	Score	Toxic in training set
SCFP_6	399659969	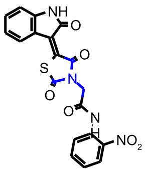 [*]CN1C(=[*])[*][*]C1 =[*]	-0.526	3 out of 11
SCFP_6	-1380909229	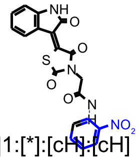 [*][c]1:[*]:[cH]:[cH] :[cH]:[c]:1[N+](=[*])[*]	-0.449	6 out of 19
SCFP_6	1311339974	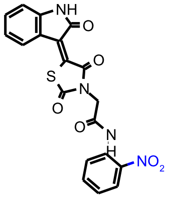 [*][N+](=O)[*]	-0.446	3 out of 10

Sorafenib

TOPKAT_Developmental_Toxicity_Potential

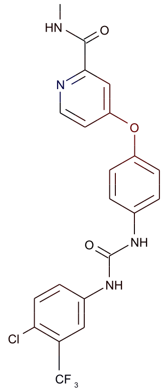

$C_{21}H_{16}ClF_3N_4O_3$
Molecular Weight: 464.82494
ALogP: 4.175
Rotatable Bonds: 6
Acceptors: 4
Donors: 3

Model Prediction

Prediction: Toxic

Probability: 0.592

Enrichment: 1.13

Bayesian Score: 1.15

Mahalanobis Distance: 12.6

Mahalanobis Distance p-value: 2.07e-006

Prediction: Positive if the Bayesian score is above the estimated best cutoff value from minimizing the false positive and false negative rate.
Probability: The esimated probability that the sample is in the positive category. This assumes that the Bayesian score follows a normal distribution and is different from the prediction using a cutoff.
Enrichment: An estimate of enrichment, that is, the increased likelihood (versus random) of this sample being in the category.
Bayesian Score: The standard Laplacian-modified Bayesian score.
Mahalanobis Distance: The Mahalanobis distance (MD) is the distance to the center of the training data. The larger the MD, the less trustworthy the prediction.
Mahalanobis Distance p-value: The p-value gives the fraction of training data with an MD greater than or equal to the one for the given sample, assuming normally distributed data. The smaller the p-value, the less trustworthy the prediciton. For highly non-normal X properties (e.g., fingerprints), the MD p-value is wildly inaccurate.

Structural Similar Compounds

Name	Chenodiol	Amsacrine	Ochratoxin a
Structure	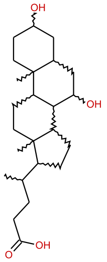	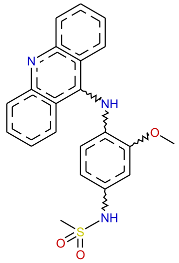	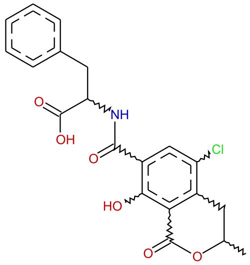
Actual Endpoint	Toxic	Toxic	Toxic
Predicted Endpoint	Toxic	Toxic	Toxic
Distance	0.631	0.637	0.644
Reference	Arch Int Pharm 246:149-158; 1980	Fundam Appl Toxicol 7(2):214-20; 1986	Toxicol Appl Pharmacol 37(2):331-8; 1976

Model Applicability

Unknown features are fingerprint features in the query molecule, but not found or appearing too infrequently in the training set.

1. All properties and OPS components are within expected ranges.

Feature Contribution

Top features for positive contribution

Fingerprint	Bit/Smiles	Feature Structure	Score	Toxic in training set
SCFP_6	1559190850	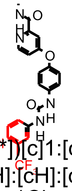 [*]C([*])([*])[c]1:[cH]:[*]:[cH]:[cH]:[c]:1Cl	0.441	3 out of 3

SCFP_6	-488587948	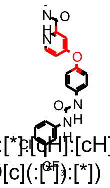 [*][c]1:[*]:[cH]:[cH]:[c](O[c](:[*]):[*]):[cH]:1	0.381	2 out of 2
SCFP_6	-975241316	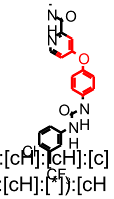 [*][c]1:[cH]:[cH]:[c](O[c](:[cH]:[*]):[cH]:[*]):[cH]:[cH]:1	0.381	2 out of 2
**Top Features for negative contribution**				
**Fingerprint**	**Bit/Smiles**	**Feature Structure**	**Score**	**Toxic in training set**
SCFP_6	-1794974220	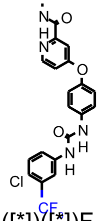 [*]C([*])([*])F	-0.55	2 out of 8
SCFP_6	-937094999	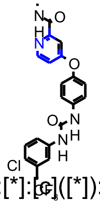 [*][c]1:[*]:[c]([*]):n:[cH]:[cH]:1	-0.358	3 out of 9
SCFP_6	-496201075	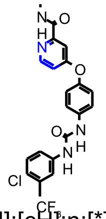 [*]:[cH]:[cH]:n:[*]	-0.289	8 out of 21

Sunitinib

TOPKAT_Developmental_Toxicity_Potential

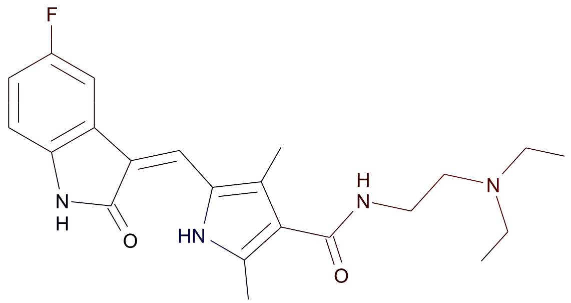

$C_{22}H_{27}FN_4O_2$
Molecular Weight: 398.47378
ALogP: 2.997
Rotatable Bonds: 7
Acceptors: 3
Donors: 3

Model Prediction

Prediction: Toxic
Probability: 0.635
Enrichment: 1.21
Bayesian Score: 2.26
Mahalanobis Distance: 13.3
Mahalanobis Distance p-value: 1.35e-007

Prediction: Positive if the Bayesian score is above the estimated best cutoff value from minimizing the false positive and false negative rate.
Probability: The esimated probability that the sample is in the positive category. This assumes that the Bayesian score follows a normal distribution and is different from the prediction using a cutoff.
Enrichment: An estimate of enrichment, that is, the increased likelihood (versus random) of this sample being in the category.
Bayesian Score: The standard Laplacian-modified Bayesian score.
Mahalanobis Distance: The Mahalanobis distance (MD) is the distance to the center of the training data. The larger the MD, the less trustworthy the prediction.
Mahalanobis Distance p-value: The p-value gives the fraction of training data with an MD greater than or equal to the one for the given sample, assuming normally distributed data. The smaller the p-value, the less trustworthy the prediciton. For highly non-normal X properties (e.g., fingerprints), the MD p-value is wildly inaccurate.

Structural Similar Compounds

Name	Domperidone	Clebopride Malate	Dobutamine .HCl (Free base form)
Structure	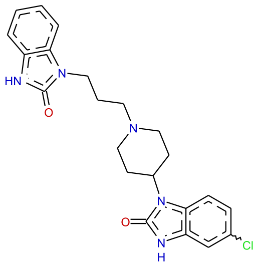	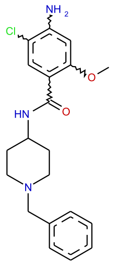	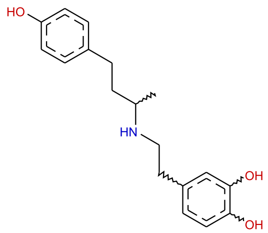
Actual Endpoint	Toxic	Non-Toxic	Toxic
Predicted Endpoint	Toxic	Non-Toxic	Toxic
Distance	0.619	0.627	0.678
Reference	Yakuri to Chiryo 8:4125-4136; 1980	Kiso to Rinsho 16:5649-5660; 1982	Yakuri to Chiryo 7:1707-1730; 1979

Model Applicability

Unknown features are fingerprint features in the query molecule, but not found or appearing too infrequently in the training set.

1. All properties and OPS components are within expected ranges.

Feature Contribution

Top features for positive contribution

Fingerprint	Bit/Smiles	Feature Structure	Score	Toxic in training set
SCFP_6	-1971137145	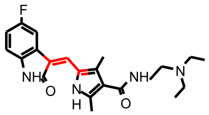 [*]C(=C[c](:[*]):[*])[*]	0.431	7 out of 8

SCFP_6	-182283812	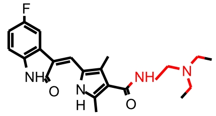[*]CN(C[*])CCN[*]	0.381	2 out of 2
SCFP_6	1725890097	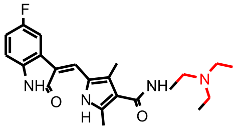[*]CN(C[*])CC	0.381	2 out of 2

Top Features for negative contribution

**Fingerprint**	**Bit/Smiles**	**Feature Structure**	**Score**	**Toxic in training set**
SCFP_6	2109374332	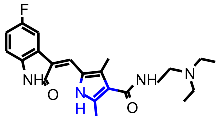[*][c]1:[*]:[*]:[nH]:[c]:1C	-0.446	3 out of 10
SCFP_6	1420330831	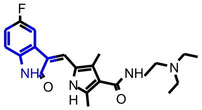[*]=C1[*]=C[c]2:[cH]:[*]:[cH]:[cH]:[c]:2N1	-0.422	0 out of 1
SCFP_6	136686699	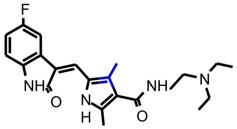[*]:[c](:[*])C	-0.316	7 out of 19

TOPKAT_Mouse_Female_FDA_None_vs_Carcinogen

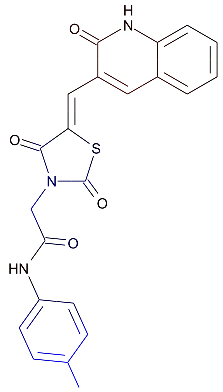

$C_{22}H_{17}N_3O_4S$
Molecular Weight: 419.45307
ALogP: 2.653
Rotatable Bonds: 4
Acceptors: 5
Donors: 2

Model Prediction

Prediction: Non-Carcinogen

Probability: 0.205

Enrichment: 0.639

Bayesian Score: -6.44

Mahalanobis Distance: 10.9

Mahalanobis Distance p-value: 0.124

Prediction: Positive if the Bayesian score is above the estimated best cutoff value from minimizing the false positive and false negative rate.
Probability: The esimated probability that the sample is in the positive category. This assumes that the Bayesian score follows a normal distribution and is different from the prediction using a cutoff.
Enrichment: An estimate of enrichment, that is, the increased likelihood (versus random) of this sample being in the category.
Bayesian Score: The standard Laplacian-modified Bayesian score.
Mahalanobis Distance: The Mahalanobis distance (MD) is the distance to the center of the training data. The larger the MD, the less trustworthy the prediction.
Mahalanobis Distance p-value: The p-value gives the fraction of training data with an MD greater than or equal to the one for the given sample, assuming normally distributed data. The smaller the p-value, the less trustworthy the prediciton. For highly non-normal X properties (e.g., fingerprints), the MD p-value is wildly inaccurate.

Structural Similar Compounds

Name	Bicalutamide	Indapamide	Metolazone
Structure	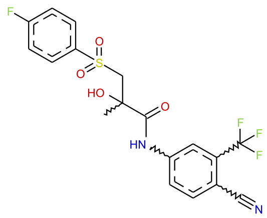	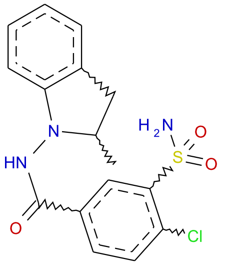	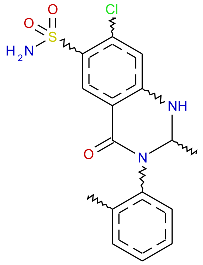
Actual Endpoint	Non-Carcinogen	Non-Carcinogen	Non-Carcinogen
Predicted Endpoint	Non-Carcinogen	Non-Carcinogen	Non-Carcinogen
Distance	0.583	0.597	0.624
Reference	US FDA (Centre for Drug Eval.& Res./Off. Testing & Res.) Sept. 1997	US FDA (Centre for Drug Eval.& Res./Off. Testing & Res.) Sept. 1997	US FDA (Centre for Drug Eval.& Res./Off. Testing & Res.) Sept. 1997

Model Applicability

Unknown features are fingerprint features in the query molecule, but not found or appearing too infrequently in the training set.

1. All properties and OPS components are within expected ranges.
2. Unknown ECFP_2 feature: 2131425032: [*]\C=C(\C=[*])/C(=[*])[*]
3. Unknown ECFP_2 feature: 1182722866: [*]C(=CC(=[*])[*])[*]
4. Unknown ECFP_2 feature: 1000552169: [*]\C=C\1/S[*][*]C1=[*]
5. Unknown ECFP_2 feature: 190445529: [*]N1[*][*]SC1=O

Feature Contribution

Top features for positive contribution				
**Fingerprint**	**Bit/Smiles**	**Feature Structure**	**Score**	**Carcinogen in training set**
ECFP_6	738938915	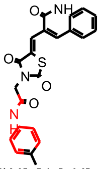 [*]C(=[*])N[c]1:[cH]:[cH]:[*]:[cH]:[cH]:1	0.617	2 out of 2

ECFP_6	464808839	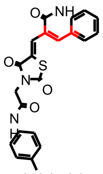 [*]C(=C[c](:[*]):[*])[*]	0.524	8 out of 14
ECFP_6	-1925046727	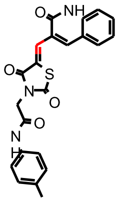 [*]C=[*]	0.391	11 out of 23

Top Features for negative contribution

Fingerprint	Bit/Smiles	Feature Structure	Score	Carcinogen in training set
ECFP_6	-661097313	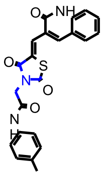 [*]CN1C(=[*])[*][*]C1=[*]	-1.55	0 out of 12
ECFP_6	-179515162	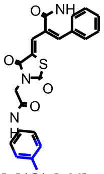 [*]:[cH]:[c](C):[cH]:[*]	-1.41	0 out of 10
ECFP_6	-210573707	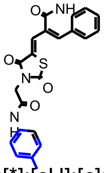 [*][c]1:[*]:[cH]:[c](C):[cH]:[cH]:1	-1.25	0 out of 8

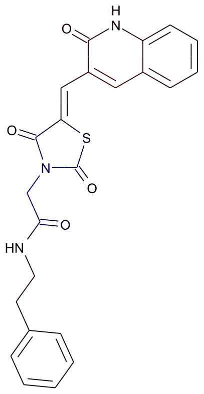

$C_{23}H_{19}N_3O_4S$

Molecular Weight: 433.47965

ALogP: 2.495

Rotatable Bonds: 6

Acceptors: 5

Donors: 2

Model Prediction

Prediction: Non-Carcinogen

Probability: 0.232

Enrichment: 0.725

Bayesian Score: -1.86

Mahalanobis Distance: 12.2

Mahalanobis Distance p-value: 0.00573

Prediction: Positive if the Bayesian score is above the estimated best cutoff value from minimizing the false positive and false negative rate.
Probability: The esimated probability that the sample is in the positive category. This assumes that the Bayesian score follows a normal distribution and is different from the prediction using a cutoff.
Enrichment: An estimate of enrichment, that is, the increased likelihood (versus random) of this sample being in the category.
Bayesian Score: The standard Laplacian-modified Bayesian score.
Mahalanobis Distance: The Mahalanobis distance (MD) is the distance to the center of the training data. The larger the MD, the less trustworthy the prediction.
Mahalanobis Distance p-value: The p-value gives the fraction of training data with an MD greater than or equal to the one for the given sample, assuming normally distributed data. The smaller the p-value, the less trustworthy the prediciton. For highly non-normal X properties (e.g., fingerprints), the MD p-value is wildly inaccurate.

Structural Similar Compounds

Name	Bicalutamide	Glipizide	Moricizine
Structure	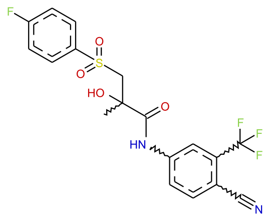	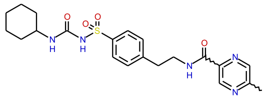	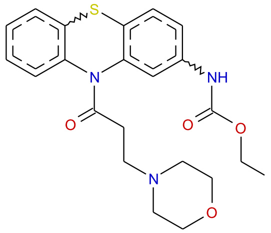
Actual Endpoint	Non-Carcinogen	Non-Carcinogen	Carcinogen
Predicted Endpoint	Non-Carcinogen	Non-Carcinogen	Carcinogen
Distance	0.512	0.628	0.648
Reference	US FDA (Centre for Drug Eval.& Res./Off. Testing & Res.) Sept. 1997	US FDA (Centre for Drug Eval.& Res./Off. Testing & Res.) Sept. 1997	US FDA (Centre for Drug Eval.& Res./Off. Testing & Res.) Sept. 1997

Model Applicability

Unknown features are fingerprint features in the query molecule, but not found or appearing too infrequently in the training set.

1. All properties and OPS components are within expected ranges.
2. Unknown ECFP_2 feature: 2131425032: [*]\C=C(\C=[*])/C(=[*])[*]
3. Unknown ECFP_2 feature: 1182722866: [*]C(=CC(=[*])[*])[*]
4. Unknown ECFP_2 feature: 1000552169: [*]\C=C\1/S[*][*]C1=[*]
5. Unknown ECFP_2 feature: 190445529: [*]N1[*][*]SC1=O

Feature Contribution

Top features for positive contribution				
**Fingerprint**	**Bit/Smiles**	**Feature Structure**	**Score**	**Carcinogen in training set**
ECFP_6	464808839	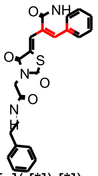 [*]C(=C[c](:[*]):[*]) [*]	0.524	8 out of 14

ECFP_6	-1925046727	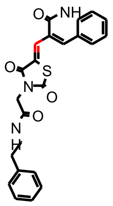 [*]C=[*]	0.391	11 out of 23
ECFP_6	-1699286547	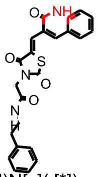 [*]C(=[*])N[c](:[*]):[*]	0.297	12 out of 28
**Top Features for negative contribution**				
**Fingerprint**	**Bit/Smiles**	**Feature Structure**	**Score**	**Carcinogen in training set**
ECFP_6	-661097313	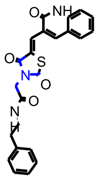 [*]CN1C(=[*])[*][*]C1=[*]	-1.55	0 out of 12
ECFP_6	1731843802	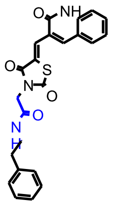 [*]CC(=O)N[*]	-0.657	0 out of 3
ECFP_6	912478223	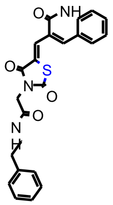 [*]S[*]	-0.638	1 out of 9

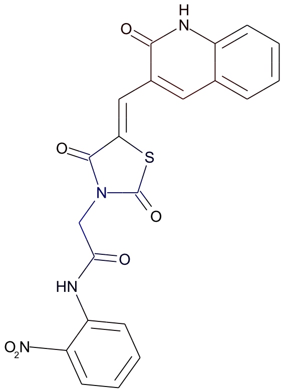

$C_{21}H_{14}N_4O_6S$
Molecular Weight: 450.42405
ALogP: 2.061
Rotatable Bonds: 5
Acceptors: 7
Donors: 2

Model Prediction

Prediction: Non-Carcinogen
Probability: 0.234
Enrichment: 0.731
Bayesian Score: -1.73
Mahalanobis Distance: 11.9
Mahalanobis Distance p-value: 0.0128

Prediction: Positive if the Bayesian score is above the estimated best cutoff value from minimizing the false positive and false negative rate.
Probability: The esimated probability that the sample is in the positive category. This assumes that the Bayesian score follows a normal distribution and is different from the prediction using a cutoff.
Enrichment: An estimate of enrichment, that is, the increased likelihood (versus random) of this sample being in the category.
Bayesian Score: The standard Laplacian-modified Bayesian score.
Mahalanobis Distance: The Mahalanobis distance (MD) is the distance to the center of the training data. The larger the MD, the less trustworthy the prediction.
Mahalanobis Distance p-value: The p-value gives the fraction of training data with an MD greater than or equal to the one for the given sample, assuming normally distributed data. The smaller the p-value, the less trustworthy the prediciton. For highly non-normal X properties (e.g., fingerprints), the MD p-value is wildly inaccurate.

Structural Similar Compounds

Name	Nedocromil	Penicillin	Sulfasalazine
Structure	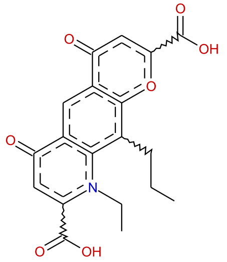	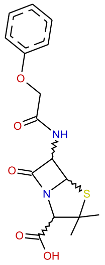	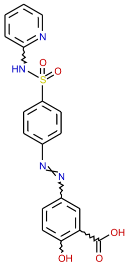
Actual Endpoint	Non-Carcinogen	Non-Carcinogen	Carcinogen
Predicted Endpoint	Non-Carcinogen	Non-Carcinogen	Carcinogen
Distance	0.649	0.675	0.702
Reference	US FDA (Centre for Drug Eval.& Res./Off. Testing & Res.) Sept. 1997	US FDA (Centre for Drug Eval.& Res./Off. Testing & Res.) Sept. 1997	US FDA (Centre for Drug Eval.& Res./Off. Testing & Res.) Sept. 1997

Model Applicability

Unknown features are fingerprint features in the query molecule, but not found or appearing too infrequently in the training set.

1. All properties and OPS components are within expected ranges.
2. Unknown ECFP_2 feature: 1043790491: [*][N+](=[*])[*]
3. Unknown ECFP_2 feature: 781519895: [*][O-]
4. Unknown ECFP_2 feature: 2131425032: [*]\C=C(\C=[*])/C(=[*])[*]
5. Unknown ECFP_2 feature: 1182722866: [*]C(=CC(=[*])[*])[*]
6. Unknown ECFP_2 feature: 1000552169: [*]\C=C\1/S[*][*]C1=[*]
7. Unknown ECFP_2 feature: 190445529: [*]N1[*][*]SC1=O
8. Unknown ECFP_2 feature: -1956535100: [*][c](:[*]):[c](:[cH]:[*])[N+](=[*])[*]
9. Unknown ECFP_2 feature: -215026467: [*]:[c](:[*])[N+](=O)[O-]
10. Unknown ECFP_2 feature: 2104376220: [*][N+](=O)[*]
11. Unknown ECFP_2 feature: -659271057: [*][N+](=[*])[O-]

Feature Contribution

Top features for positive contribution				
**Fingerprint**	**Bit/Smiles**	**Feature Structure**	**Score**	**Carcinogen in training set**

ECFP_6	464808839	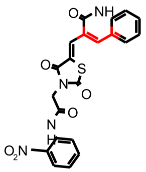 [*]C(=C[c](:[*]):[*]) [*]	0.524	8 out of 14
ECFP_6	-1925046727	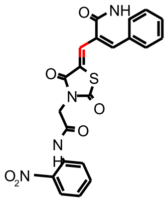 [*]C=[*]	0.391	11 out of 23
ECFP_6	-1699286547	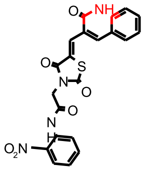 [*]C(=[*])N[c](:[*]): [*]	0.297	12 out of 28

Top Features for negative contribution

**Fingerprint**	**Bit/Smiles**	**Feature Structure**	**Score**	**Carcinogen in training set**
ECFP_6	-661097313	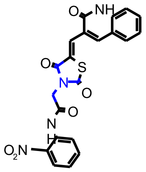 [*]CN1C(=[*])[*][*]C1 =[*]	-1.55	0 out of 12
ECFP_6	1731843802	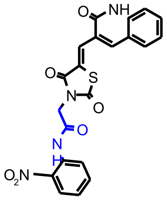 [*]CC(=O)N[*]	-0.657	0 out of 3

ECFP_6	912478223	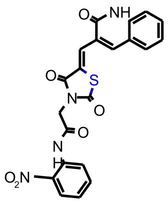 [*]S[*]	-0.638	1 out of 9

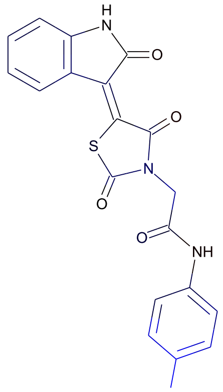

$C_{20}H_{15}N_{3}O_{4}S$
Molecular Weight: 393.4158
ALogP: 2.185
Rotatable Bonds: 3
Acceptors: 5
Donors: 2

Model Prediction

Prediction: Non-Carcinogen
Probability: 0.216
Enrichment: 0.673
Bayesian Score: -9.15
Mahalanobis Distance: 10.3
Mahalanobis Distance p-value: 0.308

Prediction: Positive if the Bayesian score is above the estimated best cutoff value from minimizing the false positive and false negative rate.
Probability: The esimated probability that the sample is in the positive category. This assumes that the Bayesian score follows a normal distribution and is different from the prediction using a cutoff.
Enrichment: An estimate of enrichment, that is, the increased likelihood (versus random) of this sample being in the category.
Bayesian Score: The standard Laplacian-modified Bayesian score.
Mahalanobis Distance: The Mahalanobis distance (MD) is the distance to the center of the training data. The larger the MD, the less trustworthy the prediction.
Mahalanobis Distance p-value: The p-value gives the fraction of training data with an MD greater than or equal to the one for the given sample, assuming normally distributed data. The smaller the p-value, the less trustworthy the prediciton. For highly non-normal X properties (e.g., fingerprints), the MD p-value is wildly inaccurate.

Structural Similar Compounds

Name	Metolazone	Indapamide	Tolazamide
Structure	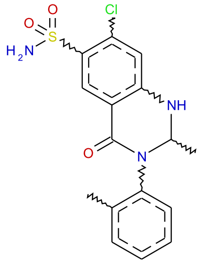	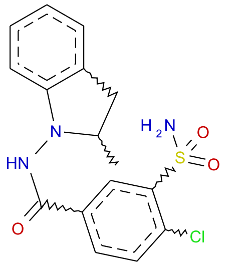	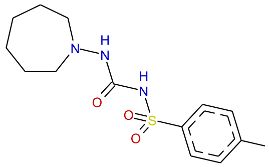
Actual Endpoint	Non-Carcinogen	Non-Carcinogen	Non-Carcinogen
Predicted Endpoint	Non-Carcinogen	Non-Carcinogen	Non-Carcinogen
Distance	0.565	0.567	0.608
Reference	US FDA (Centre for Drug Eval.& Res./Off. Testing & Res.) Sept. 1997	US FDA (Centre for Drug Eval.& Res./Off. Testing & Res.) Sept. 1997	US FDA (Centre for Drug Eval.& Res./Off. Testing & Res.) Sept. 1997

Model Applicability

Unknown features are fingerprint features in the query molecule, but not found or appearing too infrequently in the training set.

1. All properties and OPS components are within expected ranges.
2. Unknown ECFP_2 feature: -631778390: [*]C(=C1S[*][*]C1=[*])[*]
3. Unknown ECFP_2 feature: 190445529: [*]N1[*][*]SC1=O

Feature Contribution

Top features for positive contribution

Fingerprint	Bit/Smiles	Feature Structure	Score	Carcinogen in training set
ECFP_6	738938915	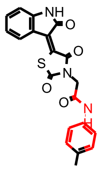 [*]C(=[*])N[c]1:[cH]:[cH]:[*]:[cH]:[cH]:1	0.617	2 out of 2

ECFP_6	-1699286547	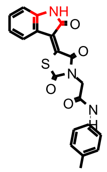 [*]C(=[*])N[c](:[*]):[*]	0.297	12 out of 28
ECFP_6	1298725959	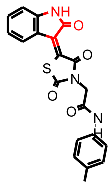 [*]NC(=O)C(=[*])[*]	0.279	4 out of 9
**Top Features for negative contribution**				
**Fingerprint**	**Bit/Smiles**	**Feature Structure**	**Score**	**Carcinogen in training set**
ECFP_6	-661097313	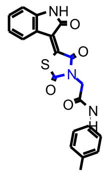 [*]CN1C(=[*])[*][*]C1=[*]	-1.55	0 out of 12
ECFP_6	-179515162	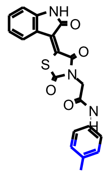 [*]:[cH]:[c](C):[cH]:[*]	-1.41	0 out of 10
ECFP_6	-210573707	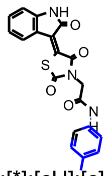 [*][c]1:[*]:[cH]:[c](C):[cH]:[cH]:1	-1.25	0 out of 8

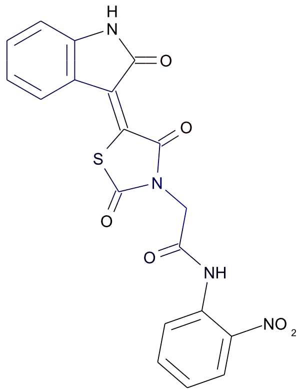

$C_{19}H_{12}N_4O_6S$
Molecular Weight: 424.38678
ALogP: 1.593
Rotatable Bonds: 4
Acceptors: 7
Donors: 2

Model Prediction

Prediction: Non-Carcinogen
Probability: 0.207
Enrichment: 0.647
Bayesian Score: -4.93
Mahalanobis Distance: 11.5
Mahalanobis Distance p-value: 0.0384

Prediction: Positive if the Bayesian score is above the estimated best cutoff value from minimizing the false positive and false negative rate.
Probability: The esimated probability that the sample is in the positive category. This assumes that the Bayesian score follows a normal distribution and is different from the prediction using a cutoff.
Enrichment: An estimate of enrichment, that is, the increased likelihood (versus random) of this sample being in the category.
Bayesian Score: The standard Laplacian-modified Bayesian score.
Mahalanobis Distance: The Mahalanobis distance (MD) is the distance to the center of the training data. The larger the MD, the less trustworthy the prediction.
Mahalanobis Distance p-value: The p-value gives the fraction of training data with an MD greater than or equal to the one for the given sample, assuming normally distributed data. The smaller the p-value, the less trustworthy the prediciton. For highly non-normal X properties (e.g., fingerprints), the MD p-value is wildly inaccurate.

Structural Similar Compounds

Name	Penicillin	Nedocromil	Acetohexamide
Structure	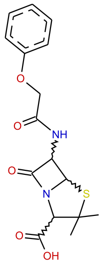	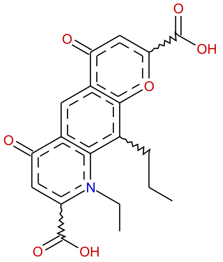	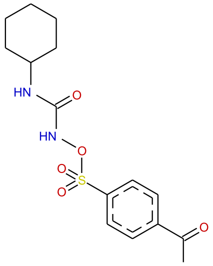
Actual Endpoint	Non-Carcinogen	Non-Carcinogen	Non-Carcinogen
Predicted Endpoint	Non-Carcinogen	Non-Carcinogen	Non-Carcinogen
Distance	0.649	0.668	0.737
Reference	US FDA (Centre for Drug Eval.& Res./Off. Testing & Res.) Sept. 1997	US FDA (Centre for Drug Eval.& Res./Off. Testing & Res.) Sept. 1997	US FDA (Centre for Drug Eval.& Res./Off. Testing & Res.) Sept. 1997

Model Applicability

Unknown features are fingerprint features in the query molecule, but not found or appearing too infrequently in the training set.

1. All properties and OPS components are within expected ranges.
2. Unknown ECFP_2 feature: 1043790491: [*][N+](=[*])[*]
3. Unknown ECFP_2 feature: 781519895: [*][O-]
4. Unknown ECFP_2 feature: -631778390: [*]C(=C1S[*][*]C1=[*])[*]
5. Unknown ECFP_2 feature: 190445529: [*]N1[*][*]SC1=O
6. Unknown ECFP_2 feature: -1956535100: [*][c](:[*]):[c](:[cH]:[*])[N+](=[*])[*]
7. Unknown ECFP_2 feature: -215026467: [*]:[c](:[*])[N+](=O)[O-]
8. Unknown ECFP_2 feature: 2104376220: [*][N+](=O)[*]
9. Unknown ECFP_2 feature: -659271057: [*][N+](=[*])[O-]

Feature Contribution

Top features for positive contribution				
**Fingerprint**	**Bit/Smiles**	**Feature Structure**	**Score**	**Carcinogen in training set**

ECFP_6	-1699286547	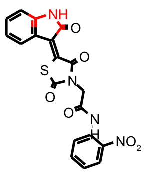 [*]C(=[*])N[c](:[*]):[*]	0.297	12 out of 28
ECFP_6	1298725959	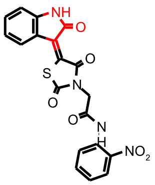 [*]NC(=O)C(=[*])[*]	0.279	4 out of 9
ECFP_6	2106656448	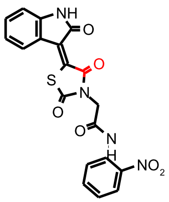 [*]C(=O)[*]	0.254	31 out of 77
**Top Features for negative contribution**				
**Fingerprint**	**Bit/Smiles**	**Feature Structure**	**Score**	**Carcinogen in training set**
ECFP_6	-661097313	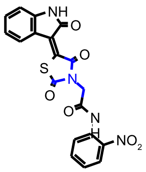 [*]CN1C(=[*])[*][*]C1=[*]	-1.55	0 out of 12
ECFP_6	1731843802	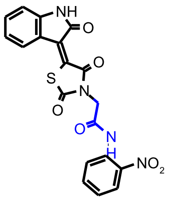 [*]CC(=O)N[*]	-0.657	0 out of 3

ECFP_6	912478223	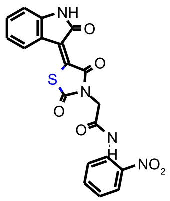 [*]S[*]	-0.638	1 out of 9

Sorafenib

TOPKAT_Mouse_Female_FDA_None_vs_Carcinogen

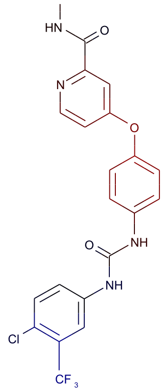

$C_{21}H_{16}ClF_3N_4O_3$
Molecular Weight: 464.82494
ALogP: 4.175
Rotatable Bonds: 6
Acceptors: 4
Donors: 3

Model Prediction

Prediction: Carcinogen
Probability: 0.257
Enrichment: 0.801
Bayesian Score: -0.321
Mahalanobis Distance: 14.9
Mahalanobis Distance p-value: 4.21e-007

Prediction: Positive if the Bayesian score is above the estimated best cutoff value from minimizing the false positive and false negative rate.
Probability: The esimated probability that the sample is in the positive category. This assumes that the Bayesian score follows a normal distribution and is different from the prediction using a cutoff.
Enrichment: An estimate of enrichment, that is, the increased likelihood (versus random) of this sample being in the category.
Bayesian Score: The standard Laplacian-modified Bayesian score.
Mahalanobis Distance: The Mahalanobis distance (MD) is the distance to the center of the training data. The larger the MD, the less trustworthy the prediction.
Mahalanobis Distance p-value: The p-value gives the fraction of training data with an MD greater than or equal to the one for the given sample, assuming normally distributed data. The smaller the p-value, the less trustworthy the prediciton. For highly non-normal X properties (e.g., fingerprints), the MD p-value is wildly inaccurate.

Structural Similar Compounds

Name	Glimepride	Glyburide	Fluvastatin
Structure	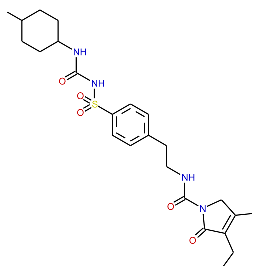	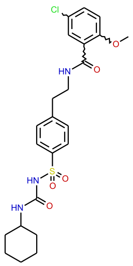	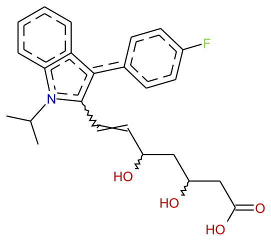
Actual Endpoint	Carcinogen	Non-Carcinogen	Non-Carcinogen
Predicted Endpoint	Carcinogen	Non-Carcinogen	Non-Carcinogen
Distance	0.605	0.615	0.625
Reference	US FDA (Centre for Drug Eval.& Res./Off. Testing & Res.) Sept. 1997	US FDA (Centre for Drug Eval.& Res./Off. Testing & Res.) Sept. 1997	US FDA (Centre for Drug Eval.& Res./Off. Testing & Res.) Sept. 1997

Model Applicability

Unknown features are fingerprint features in the query molecule, but not found or appearing too infrequently in the training set.

1. OPS PC20 out of range. Value: -3.3309. Training min, max, SD, explained variance: -3.1862, 4.4571, 1.28, 0.0167.

Feature Contribution

Top features for positive contribution

Fingerprint	Bit/Smiles	Feature Structure	Score	Carcinogen in training set
ECFP_6	738938915	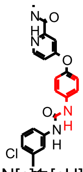 [*]C(=[*])N[c]:[cH]:[cH]:[*]:[cH]:[cH]:1	0.617	2 out of 2

ECFP_6	1338334141	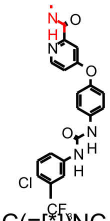 [*]C(=[*])NC	0.442	2 out of 3
ECFP_6	-335167981	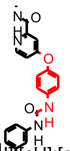 [*]O[c]1:[cH]:[cH]:[c](NC(=[*])[*]):[cH]: [cH]:1	0.424	1 out of 1

Top Features for negative contribution

Fingerprint	Bit/Smiles	Feature Structure	Score	Carcinogen in training set
ECFP_6	1335691903	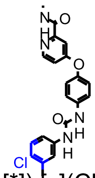 [*][c](:[*]):[c](Cl): [cH]:[*]	-0.669	3 out of 22
ECFP_6	-1952889961	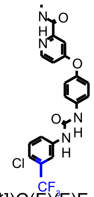 [*]:[c](:[*])C(F)(F)F	-0.657	0 out of 3
ECFP_6	1336678434	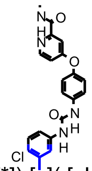 [*][c](:[*]):[c](:[cH]:[*])C([*])([*])[*]	-0.657	0 out of 3

Sunitinib

TOPKAT_Mouse_Female_FDA_None_vs_Carcinogen

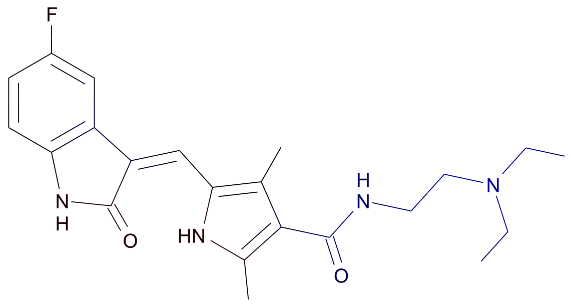

$C_{22}H_{27}FN_4O_2$
Molecular Weight: 398.47378
ALogP: 2.997
Rotatable Bonds: 7
Acceptors: 3
Donors: 3

Model Prediction

Prediction: Non-Carcinogen
Probability: 0.218
Enrichment: 0.679
Bayesian Score: -3.25
Mahalanobis Distance: 13.8
Mahalanobis Distance p-value: 3.24e-005

Prediction: Positive if the Bayesian score is above the estimated best cutoff value from minimizing the false positive and false negative rate.
Probability: The esimated probability that the sample is in the positive category. This assumes that the Bayesian score follows a normal distribution and is different from the prediction using a cutoff.
Enrichment: An estimate of enrichment, that is, the increased likelihood (versus random) of this sample being in the category.
Bayesian Score: The standard Laplacian-modified Bayesian score.
Mahalanobis Distance: The Mahalanobis distance (MD) is the distance to the center of the training data. The larger the MD, the less trustworthy the prediction.
Mahalanobis Distance p-value: The p-value gives the fraction of training data with an MD greater than or equal to the one for the given sample, assuming normally distributed data. The smaller the p-value, the less trustworthy the prediciton. For highly non-normal X properties (e.g., fingerprints), the MD p-value is wildly inaccurate.

Structural Similar Compounds

Name	Fluvastatin	Metoclopramide	Flecainide
Structure	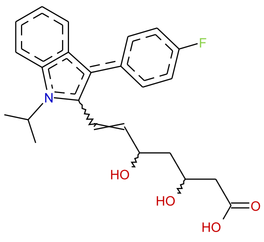	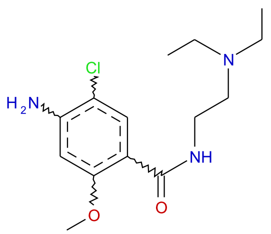	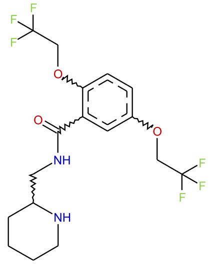
Actual Endpoint	Non-Carcinogen	Non-Carcinogen	Non-Carcinogen
Predicted Endpoint	Non-Carcinogen	Non-Carcinogen	Non-Carcinogen
Distance	0.610	0.643	0.666
Reference	US FDA (Centre for Drug Eval.& Res./Off. Testing & Res.) Sept. 1997	US FDA (Centre for Drug Eval.& Res./Off. Testing & Res.) Sept. 1997	US FDA (Centre for Drug Eval.& Res./Off. Testing & Res.) Sept. 1997

Model Applicability

Unknown features are fingerprint features in the query molecule, but not found or appearing too infrequently in the training set.

1. All properties and OPS components are within expected ranges.
2. Unknown ECFP_2 feature: -1658273810: [*]C(=[*])[c]1:[c]([*]):[*]:[*]:[c]:1[*]
3. Unknown ECFP_2 feature: 980271847: [*][c]1:[*]:[*]:[nH]:[c]:1C=[*]
4. Unknown ECFP_2 feature: 1182722866: [*]C(=CC(=[*])[*])[*]

Feature Contribution

Top features for positive contribution

Fingerprint	Bit/Smiles	Feature Structure	Score	Carcinogen in training set
ECFP_6	1791989338	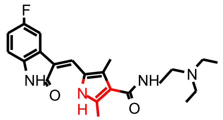 [*][c]1:[*]:[*]:[nH]:[c]:1C	0.424	1 out of 1

ECFP_6	-1925046727	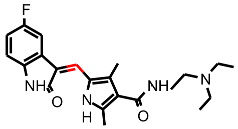 [*]C=[*]	0.391	11 out of 23
ECFP_6	-1699286547	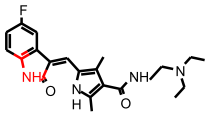 [*]C(=[*])N[c](:[*]):[*]	0.297	12 out of 28

Top Features for negative contribution

Fingerprint	Bit/Smiles	Feature Structure	Score	Carcinogen in training set
ECFP_6	-628327667	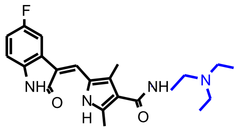 [*]CCN(CC)CC	-0.657	0 out of 3
ECFP_6	1795893449	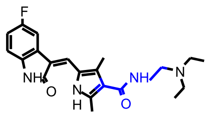 [*]CCNC(=O)[c](:[*]):[*]	-0.657	0 out of 3
ECFP_6	-659402940	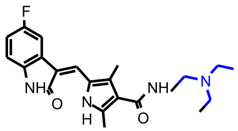 [*]CN(C[*])CC	-0.657	0 out of 3

Sorafenib

TOPKAT_Mouse_Female_FDA_Single_vs_Multiple

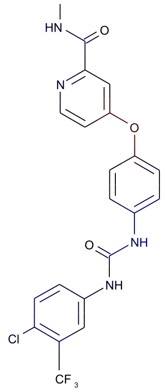

$C_{21}H_{16}ClF_3N_4O_3$
Molecular Weight: 464.82494
ALogP: 4.175
Rotatable Bonds: 6
Acceptors: 4
Donors: 3

Model Prediction

Prediction: Single-Carcinogen
Probability: 0.283
Enrichment: 0.691
Bayesian Score: -3.89
Mahalanobis Distance: 11.1
Mahalanobis Distance p-value: 0.00221

Prediction: Positive if the Bayesian score is above the estimated best cutoff value from minimizing the false positive and false negative rate.
Probability: The esimated probability that the sample is in the positive category. This assumes that the Bayesian score follows a normal distribution and is different from the prediction using a cutoff.
Enrichment: An estimate of enrichment, that is, the increased likelihood (versus random) of this sample being in the category.
Bayesian Score: The standard Laplacian-modified Bayesian score.
Mahalanobis Distance: The Mahalanobis distance (MD) is the distance to the center of the training data. The larger the MD, the less trustworthy the prediction.
Mahalanobis Distance p-value: The p-value gives the fraction of training data with an MD greater than or equal to the one for the given sample, assuming normally distributed data. The smaller the p-value, the less trustworthy the prediciton. For highly non-normal X properties (e.g., fingerprints), the MD p-value is wildly inaccurate.

Structural Similar Compounds

Name	Glimepride	Labetalol	Lansoprazole
Structure	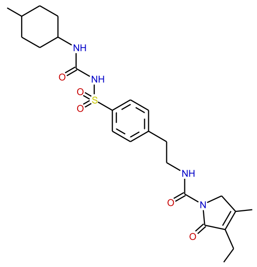	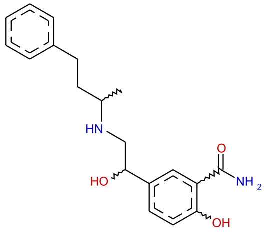	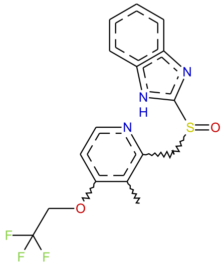
Actual Endpoint	Single-Carcinogen	Single-Carcinogen	Single-Carcinogen
Predicted Endpoint	Single-Carcinogen	Single-Carcinogen	Single-Carcinogen
Distance	0.599	0.808	0.820
Reference	US FDA (Centre for Drug Eval.& Res./Off. Testing & Res.) Sept. 1997	US FDA (Centre for Drug Eval.& Res./Off. Testing & Res.) Sept. 1997	US FDA (Centre for Drug Eval.& Res./Off. Testing & Res.) Sept. 1997

Model Applicability

Unknown features are fingerprint features in the query molecule, but not found or appearing too infrequently in the training set.

1. All properties and OPS components are within expected ranges.
2. Unknown ECFP_2 feature: 1336678434: [*][c](:[*]):[c](:[cH]:[*])C([*])([*])[*]
3. Unknown ECFP_2 feature: -1952889961: [*]:[c](:[*])C(F)(F)F

Feature Contribution

Top features for positive contribution

Fingerprint	Bit/Smiles	Feature Structure	Score	Multiple-Carcinogen in training set
ECFP_4	-834094296	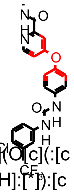 [*]:[cH]:[c](O[c](:[cH]:[*]):[cH]:[*]):[cH]:[*]	0.351	1 out of 1

ECFP_4	143734695	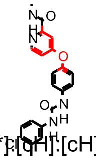 [*][c]1:[*]:[cH]:[cH]:[c](O[c](:[*]):[*]):[cH]:1	0.351	1 out of 1
ECFP_4	1407472008	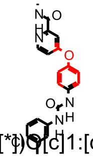 [*]:[c](:[*])O[c]1:[cH]:[cH]:[*]:[cH]:[cH]:1	0.351	1 out of 1

Top Features for negative contribution

**Fingerprint**	**Bit/Smiles**	**Feature Structure**	**Score**	**Multiple-Carcinogen in training set**
ECFP_4	1335691903	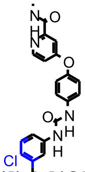 [*][c](:[*]):[c](Cl):[cH]:[*]	-0.8	0 out of 3
ECFP_4	888054369	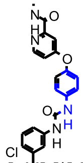 [*]N[c]1:[cH]:[*]:[c]([*]):[cH]:[cH]:1	-0.8	0 out of 3
ECFP_4	738938915	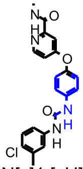 [*]C(=[*])N[c]1:[cH]:[cH]:[*]:[cH]:[cH]:1	-0.597	0 out of 2

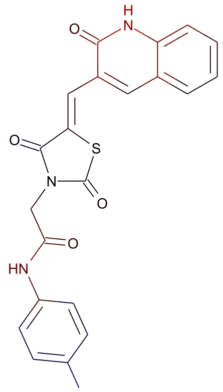

$C_{22}H_{17}N_3O_4S$
Molecular Weight: 419.45307
ALogP: 2.653
Rotatable Bonds: 4
Acceptors: 5
Donors: 2

Model Prediction

Prediction: Carcinogen
Probability: 0.38
Enrichment: 1.29
Bayesian Score: 2.42
Mahalanobis Distance: 10.9
Mahalanobis Distance p-value: 0.0852

Prediction: Positive if the Bayesian score is above the estimated best cutoff value from minimizing the false positive and false negative rate.
Probability: The esimated probability that the sample is in the positive category. This assumes that the Bayesian score follows a normal distribution and is different from the prediction using a cutoff.
Enrichment: An estimate of enrichment, that is, the increased likelihood (versus random) of this sample being in the category.
Bayesian Score: The standard Laplacian-modified Bayesian score.
Mahalanobis Distance: The Mahalanobis distance (MD) is the distance to the center of the training data. The larger the MD, the less trustworthy the prediction.
Mahalanobis Distance p-value: The p-value gives the fraction of training data with an MD greater than or equal to the one for the given sample, assuming normally distributed data. The smaller the p-value, the less trustworthy the prediciton. For highly non-normal X properties (e.g., fingerprints), the MD p-value is wildly inaccurate.

Structural Similar Compounds

Name	Bicalutamide	Indapamide	Metolazone
Structure	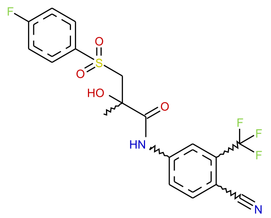	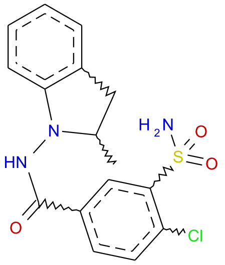	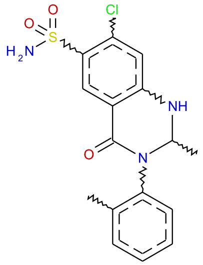
Actual Endpoint	Carcinogen	Non-Carcinogen	Non-Carcinogen
Predicted Endpoint	Carcinogen	Non-Carcinogen	Non-Carcinogen
Distance	0.554	0.591	0.605
Reference	US FDA (Centre for Drug Eval.& Res./Off. Testing & Res.) Sept. 1997	US FDA (Centre for Drug Eval.& Res./Off. Testing & Res.) Sept. 1997	US FDA (Centre for Drug Eval.& Res./Off. Testing & Res.) Sept. 1997

Model Applicability

Unknown features are fingerprint features in the query molecule, but not found or appearing too infrequently in the training set.

1. OPS PC9 out of range. Value: 5.6675. Training min, max, SD, explained variance: -5.0113, 5.5609, 1.7, 0.0303.

Feature Contribution

Top features for positive contribution				
Fingerprint	Bit/Smiles	Feature Structure	Score	Carcinogen in training set
FCFP_6	-451043714	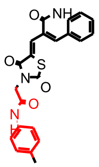 [*]CC(=O)N[c]1:[cH]:[cH]:[c]([*]):[cH]:[cH]:1	0.676	2 out of 2

FCFP_6	1175665944	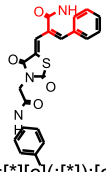 [*]C1=[*][c](:[*]):[c](NC1=O):[cH]:[*]	0.655	7 out of 12
FCFP_6	-1838187238	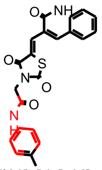 [*]C(=[*])N[c]1:[cH]: [cH]:[*]:[cH]:[cH]:1	0.565	4 out of 7
**Top Features for negative contribution**				
**Fingerprint**	**Bit/Smiles**	**Feature Structure**	**Score**	**Carcinogen in training set**
FCFP_6	-1773728142	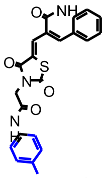 C[c]1:[cH]:[cH]:[*]:[cH]:[cH]:1	-1.29	0 out of 10
FCFP_6	2109043264	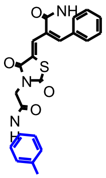 [*][c]1:[cH]:[cH]:[c] (C):[cH]:[cH]:1	-0.947	0 out of 6
FCFP_6	-1553874037	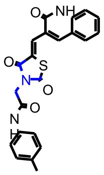 [*]CN1C(=[*])[*][*]C1 =[*]	-0.45	5 out of 32

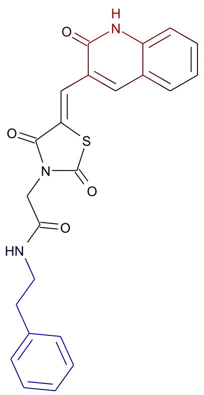

$C_{23}H_{19}N_3O_4S$

Molecular Weight: 433.47965

ALogP: 2.495

Rotatable Bonds: 6

Acceptors: 5

Donors: 2

Model Prediction

Prediction: Carcinogen

Probability: 0.284

Enrichment: 0.964

Bayesian Score: -0.845

Mahalanobis Distance: 11.6

Mahalanobis Distance p-value: 0.0177

Prediction: Positive if the Bayesian score is above the estimated best cutoff value from minimizing the false positive and false negative rate.
Probability: The esimated probability that the sample is in the positive category. This assumes that the Bayesian score follows a normal distribution and is different from the prediction using a cutoff.
Enrichment: An estimate of enrichment, that is, the increased likelihood (versus random) of this sample being in the category.
Bayesian Score: The standard Laplacian-modified Bayesian score.
Mahalanobis Distance: The Mahalanobis distance (MD) is the distance to the center of the training data. The larger the MD, the less trustworthy the prediction.
Mahalanobis Distance p-value: The p-value gives the fraction of training data with an MD greater than or equal to the one for the given sample, assuming normally distributed data. The smaller the p-value, the less trustworthy the prediciton. For highly non-normal X properties (e.g., fingerprints), the MD p-value is wildly inaccurate.

Structural Similar Compounds

Name	Bicalutamide	Glipizide	Glimepride
Structure	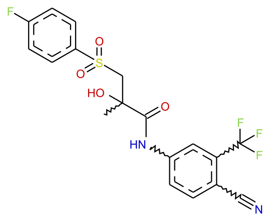	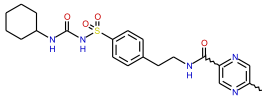	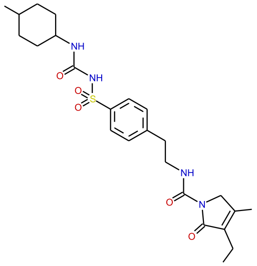
Actual Endpoint	Carcinogen	Non-Carcinogen	Carcinogen
Predicted Endpoint	Carcinogen	Non-Carcinogen	Carcinogen
Distance	0.481	0.613	0.629
Reference	US FDA (Centre for Drug Eval.& Res./Off. Testing & Res.) Sept. 1997	US FDA (Centre for Drug Eval.& Res./Off. Testing & Res.) Sept. 1997	US FDA (Centre for Drug Eval.& Res./Off. Testing & Res.) Sept. 1997

Model Applicability

Unknown features are fingerprint features in the query molecule, but not found or appearing too infrequently in the training set.

1. All properties and OPS components are within expected ranges.

Feature Contribution

Top features for positive contribution

Fingerprint	Bit/Smiles	Feature Structure	Score	Carcinogen in training set
FCFP_6	1175665944	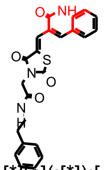 [*]C1=[*][c](:[*]):[c](NC1=O):[cH]:[*]	0.655	7 out of 12

FCFP_6	451847724	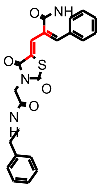 [*]C(=CC(=[*])[*])[*]	0.479	21 out of 48
FCFP_6	2036120522	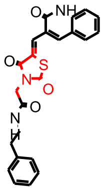 [*]CN1C(=O)SC(=[*])C1=[*]	0.46	1 out of 1
**Top Features for negative contribution**				
**Fingerprint**	**Bit/Smiles**	**Feature Structure**	**Score**	**Carcinogen in training set**
FCFP_6	1981711554	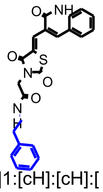 [*]CC[c]1:[cH]:[cH]:[cH]:[cH]:[cH]:1	-1.42	0 out of 12
FCFP_6	-497728148	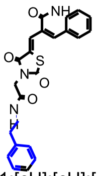 [*]CC[c]1:[cH]:[cH]:[*]:[cH]:[cH]:1	-0.96	2 out of 26
FCFP_6	-1553874037	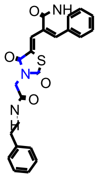 [*]CN1C(=[*])[*][*]C1=[*]	-0.45	5 out of 32

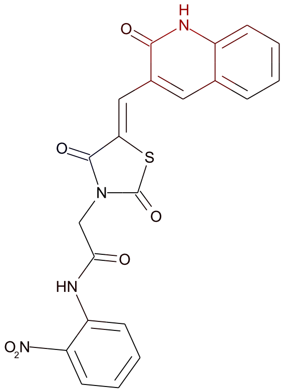

$C_{21}H_{14}N_4O_6S$
Molecular Weight: 450.42405
ALogP: 2.061
Rotatable Bonds: 5
Acceptors: 7
Donors: 2

Model Prediction

Prediction: Carcinogen
Probability: 0.4
Enrichment: 1.36
Bayesian Score: 3
Mahalanobis Distance: 10.8
Mahalanobis Distance p-value: 0.104

Prediction: Positive if the Bayesian score is above the estimated best cutoff value from minimizing the false positive and false negative rate.
Probability: The esimated probability that the sample is in the positive category. This assumes that the Bayesian score follows a normal distribution and is different from the prediction using a cutoff.
Enrichment: An estimate of enrichment, that is, the increased likelihood (versus random) of this sample being in the category.
Bayesian Score: The standard Laplacian-modified Bayesian score.
Mahalanobis Distance: The Mahalanobis distance (MD) is the distance to the center of the training data. The larger the MD, the less trustworthy the prediction.
Mahalanobis Distance p-value: The p-value gives the fraction of training data with an MD greater than or equal to the one for the given sample, assuming normally distributed data. The smaller the p-value, the less trustworthy the prediciton. For highly non-normal X properties (e.g., fingerprints), the MD p-value is wildly inaccurate.

Structural Similar Compounds

Name	Nedocromil	Penicillin	Bicalutamide
Structure	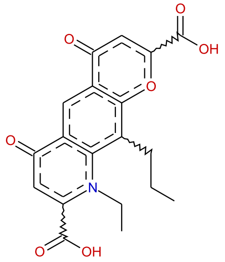	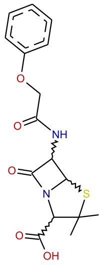	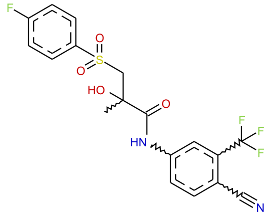
Actual Endpoint	Non-Carcinogen	Non-Carcinogen	Carcinogen
Predicted Endpoint	Non-Carcinogen	Non-Carcinogen	Carcinogen
Distance	0.646	0.673	0.691
Reference	US FDA (Centre for Drug Eval.& Res./Off. Testing & Res.) Sept. 1997	US FDA (Centre for Drug Eval.& Res./Off. Testing & Res.) Sept. 1997	US FDA (Centre for Drug Eval.& Res./Off. Testing & Res.) Sept. 1997

Model Applicability

Unknown features are fingerprint features in the query molecule, but not found or appearing too infrequently in the training set.

1. All properties and OPS components are within expected ranges.
2. Unknown FCFP_2 feature: 5: [*][O-]
3. Unknown FCFP_2 feature: -828984032: [*][c](:[*]):[c](:[cH]:[*])[N+](=[*])[*]
4. Unknown FCFP_2 feature: -1338588315: [*]:[c](:[*])[N+](=O)[O-]
5. Unknown FCFP_2 feature: 1872392852: [*][N+](=O)[*]
6. Unknown FCFP_2 feature: 260476081: [*][N+](=[*])[O-]

Feature Contribution

Top features for positive contribution

Fingerprint	Bit/Smiles	Feature Structure	Score	Carcinogen in training set

FCFP_6	1175665944	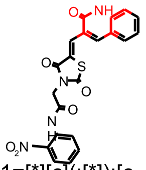 [*]C1=[*][c](:[*]):[c](NC1=O):[cH]:[*]	0.655	7 out of 12
FCFP_6	451847724	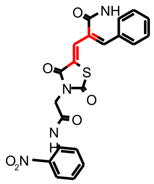 [*]C(=CC(=[*])[*])[*]	0.479	21 out of 48
FCFP_6	2036120522	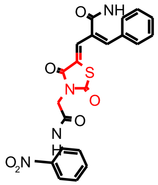 [*]CN1C(=O)SC(=[*])C1 =[*]	0.46	1 out of 1

Top Features for negative contribution

Fingerprint	Bit/Smiles	Feature Structure	Score	Carcinogen in training set
FCFP_6	-1553874037	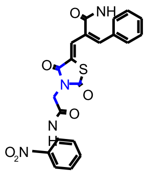 [*]CN1C(=[*])[*][*]C1 =[*]	-0.45	5 out of 32
FCFP_6	-1943081219	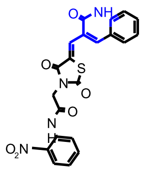 [*]=CC1=C[*]:[c](:[*])NC1=O	-0.233	0 out of 1

FCFP_6	1764344789	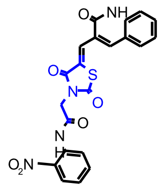<br>[*]C(=[*])CN1C(=O)SC(=[*])C1=O	-0.233	0 out of 1

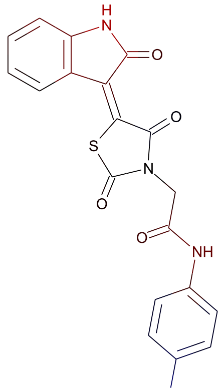

$C_{20}H_{15}N_3O_4S$
Molecular Weight: 393.4158
ALogP: 2.185
Rotatable Bonds: 3
Acceptors: 5
Donors: 2

Model Prediction

Prediction: Carcinogen
Probability: 0.364
Enrichment: 1.24
Bayesian Score: 1.95
Mahalanobis Distance: 10.4
Mahalanobis Distance p-value: 0.219

Prediction: Positive if the Bayesian score is above the estimated best cutoff value from minimizing the false positive and false negative rate.
Probability: The esimated probability that the sample is in the positive category. This assumes that the Bayesian score follows a normal distribution and is different from the prediction using a cutoff.
Enrichment: An estimate of enrichment, that is, the increased likelihood (versus random) of this sample being in the category.
Bayesian Score: The standard Laplacian-modified Bayesian score.
Mahalanobis Distance: The Mahalanobis distance (MD) is the distance to the center of the training data. The larger the MD, the less trustworthy the prediction.
Mahalanobis Distance p-value: The p-value gives the fraction of training data with an MD greater than or equal to the one for the given sample, assuming normally distributed data. The smaller the p-value, the less trustworthy the prediciton. For highly non-normal X properties (e.g., fingerprints), the MD p-value is wildly inaccurate.

Structural Similar Compounds

Name	Metolazone	Indapamide	Tolazamide
Structure	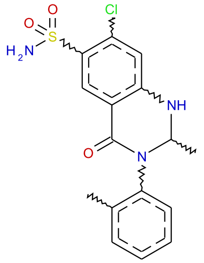	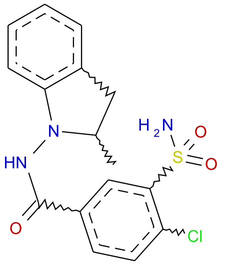	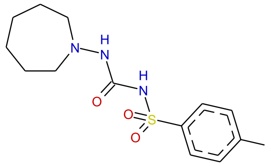
Actual Endpoint	Non-Carcinogen	Non-Carcinogen	Non-Carcinogen
Predicted Endpoint	Non-Carcinogen	Non-Carcinogen	Non-Carcinogen
Distance	0.549	0.561	0.603
Reference	US FDA (Centre for Drug Eval.& Res./Off. Testing & Res.) Sept. 1997	US FDA (Centre for Drug Eval.& Res./Off. Testing & Res.) Sept. 1997	US FDA (Centre for Drug Eval.& Res./Off. Testing & Res.) Sept. 1997

Model Applicability

Unknown features are fingerprint features in the query molecule, but not found or appearing too infrequently in the training set.

1. All properties and OPS components are within expected ranges.

Feature Contribution

Top features for positive contribution

Fingerprint	Bit/Smiles	Feature Structure	Score	Carcinogen in training set
FCFP_6	-451043714	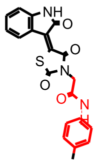 [*]CC(=O)N[c]1:[cH]:[cH]:[c]([*]):[cH]:[cH]:1	0.676	2 out of 2

FCFP_6	1175665944	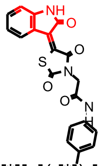 [*]C1=[*][c](:[*]):[c](NC1=O):[cH]:[*]	0.655	7 out of 12
FCFP_6	-1838187238	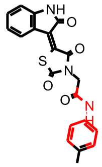 [*]C(=[*])N[c]1:[cH]: [cH]:[*]:[cH]:[cH]:1	0.565	4 out of 7

Top Features for negative contribution

Fingerprint	Bit/Smiles	Feature Structure	Score	Carcinogen in training set
FCFP_6	-1773728142	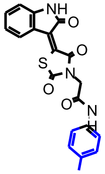 C[c]1:[cH]:[cH]:[*]:[cH]:[cH]:1	-1.29	0 out of 10
FCFP_6	2109043264	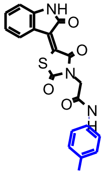 [*][c]1:[cH]:[cH]:[c] (C):[cH]:[cH]:1	-0.947	0 out of 6
FCFP_6	-1553874037	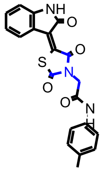 [*]CN1C(=[*])[*][*]C1 =[*]	-0.45	5 out of 32

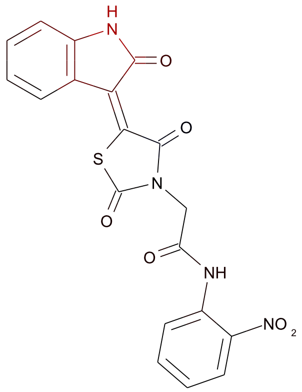

$C_{19}H_{12}N_4O_6S$
Molecular Weight: 424.38678
ALogP: 1.593
Rotatable Bonds: 4
Acceptors: 7
Donors: 2

Model Prediction

Prediction: Carcinogen
Probability: 0.373
Enrichment: 1.27
Bayesian Score: 2.22
Mahalanobis Distance: 10
Mahalanobis Distance p-value: 0.367

Prediction: Positive if the Bayesian score is above the estimated best cutoff value from minimizing the false positive and false negative rate.
Probability: The esimated probability that the sample is in the positive category. This assumes that the Bayesian score follows a normal distribution and is different from the prediction using a cutoff.
Enrichment: An estimate of enrichment, that is, the increased likelihood (versus random) of this sample being in the category.
Bayesian Score: The standard Laplacian-modified Bayesian score.
Mahalanobis Distance: The Mahalanobis distance (MD) is the distance to the center of the training data. The larger the MD, the less trustworthy the prediction.
Mahalanobis Distance p-value: The p-value gives the fraction of training data with an MD greater than or equal to the one for the given sample, assuming normally distributed data. The smaller the p-value, the less trustworthy the prediciton. For highly non-normal X properties (e.g., fingerprints), the MD p-value is wildly inaccurate.

Structural Similar Compounds

Name	Penicillin	Nedocromil	Acetohexamide
Structure	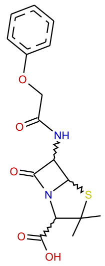	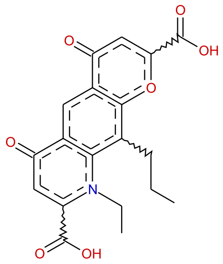	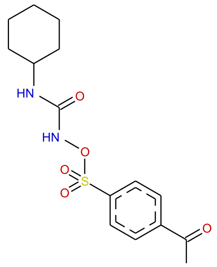
Actual Endpoint	Non-Carcinogen	Non-Carcinogen	Non-Carcinogen
Predicted Endpoint	Non-Carcinogen	Non-Carcinogen	Non-Carcinogen
Distance	0.648	0.669	0.725
Reference	US FDA (Centre for Drug Eval.& Res./Off. Testing & Res.) Sept. 1997	US FDA (Centre for Drug Eval.& Res./Off. Testing & Res.) Sept. 1997	US FDA (Centre for Drug Eval.& Res./Off. Testing & Res.) Sept. 1997

Model Applicability

Unknown features are fingerprint features in the query molecule, but not found or appearing too infrequently in the training set.

1. All properties and OPS components are within expected ranges.
2. Unknown FCFP_2 feature: 5: [*][O-]
3. Unknown FCFP_2 feature: -828984032: [*][c](:[*]):[c](:[cH]:[*])[N+](=[*])[*]
4. Unknown FCFP_2 feature: -1338588315: [*]:[c](:[*])[N+](=O)[O-]
5. Unknown FCFP_2 feature: 1872392852: [*][N+](=O)[*]
6. Unknown FCFP_2 feature: 260476081: [*][N+](=[*])[O-]

Feature Contribution

Top features for positive contribution				
Fingerprint	Bit/Smiles	Feature Structure	Score	Carcinogen in training set

FCFP_6	1175665944	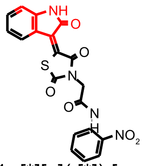 [*]C1=[*][c](:[*]):[c](NC1=O):[cH]:[*]	0.655	7 out of 12
FCFP_6	2036120522	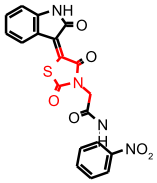 [*]CN1C(=O)SC(=[*])C1 =[*]	0.46	1 out of 1
FCFP_6	-1947166985	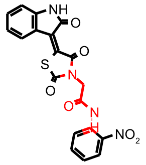 [*]N([*])CC(=O)N[c](: [*]):[*]	0.46	1 out of 1

Top Features for negative contribution

Fingerprint	Bit/Smiles	Feature Structure	Score	Carcinogen in training set
FCFP_6	-1553874037	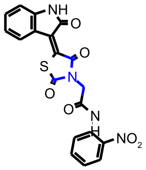 [*]CN1C(=[*])[*][*]C1 =[*]	-0.45	5 out of 32
FCFP_6	1764344789	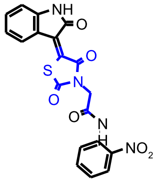 [*]C(=[*])CN1C(=O)SC(=[*])C1=O	-0.233	0 out of 1

FCFP_6	-1698724694	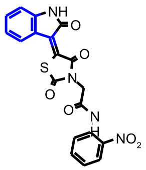<br>[*][c]1:[cH]:[cH]:[cH]:[cH]:[c]:1C=[*]	-0.22	15 out of 72

Sorafenib

TOPKAT_Mouse_Male_FDA_None_vs_Carcinogen

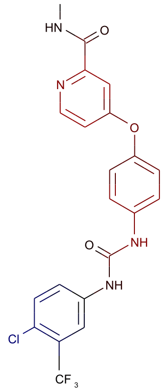

$C_{21}H_{16}ClF_3N_4O_3$
Molecular Weight: 464.82494
ALogP: 4.175
Rotatable Bonds: 6
Acceptors: 4
Donors: 3

Model Prediction

Prediction: Carcinogen
Probability: 0.444
Enrichment: 1.51
Bayesian Score: 4.21
Mahalanobis Distance: 20.3
Mahalanobis Distance p-value: 1.28e-019

Prediction: Positive if the Bayesian score is above the estimated best cutoff value from minimizing the false positive and false negative rate.
Probability: The esimated probability that the sample is in the positive category. This assumes that the Bayesian score follows a normal distribution and is different from the prediction using a cutoff.
Enrichment: An estimate of enrichment, that is, the increased likelihood (versus random) of this sample being in the category.
Bayesian Score: The standard Laplacian-modified Bayesian score.
Mahalanobis Distance: The Mahalanobis distance (MD) is the distance to the center of the training data. The larger the MD, the less trustworthy the prediction.
Mahalanobis Distance p-value: The p-value gives the fraction of training data with an MD greater than or equal to the one for the given sample, assuming normally distributed data. The smaller the p-value, the less trustworthy the prediciton. For highly non-normal X properties (e.g., fingerprints), the MD p-value is wildly inaccurate.

Structural Similar Compounds

Name	Glyburide	Glimepride	Fluvastatin
Structure	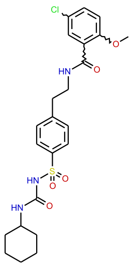	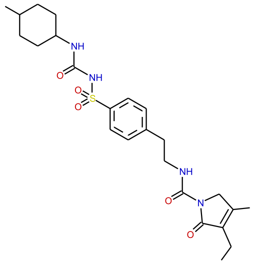	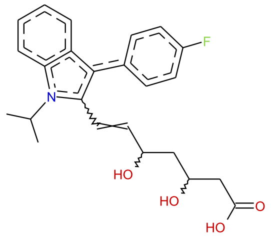
Actual Endpoint	Non-Carcinogen	Carcinogen	Non-Carcinogen
Predicted Endpoint	Non-Carcinogen	Carcinogen	Non-Carcinogen
Distance	0.594	0.599	0.603
Reference	US FDA (Centre for Drug Eval.& Res./Off. Testing & Res.) Sept. 1997	US FDA (Centre for Drug Eval.& Res./Off. Testing & Res.) Sept. 1997	US FDA (Centre for Drug Eval.& Res./Off. Testing & Res.) Sept. 1997

Model Applicability

Unknown features are fingerprint features in the query molecule, but not found or appearing too infrequently in the training set.

1. All properties and OPS components are within expected ranges.

Feature Contribution

Top features for positive contribution

Fingerprint	Bit/Smiles	Feature Structure	Score	Carcinogen in training set
FCFP_6	71953198	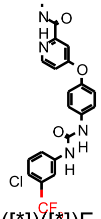 [*]C([*])([*])F	0.612	12 out of 23

FCFP_6	-1838187238	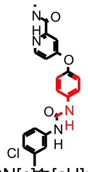 [*]C(=[*])N[c]1:[cH]:[cH]:[*]:[cH]:[cH]:1	0.565	4 out of 7
FCFP_6	-215363676	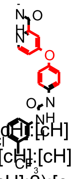 [*][c]1:[*]:[cH]:[cH]:[c](O[c]2:[cH]:[cH]:[*]:[cH]:[cH]:2):[cH]:1	0.46	1 out of 1

Top Features for negative contribution

**Fingerprint**	**Bit/Smiles**	**Feature Structure**	**Score**	**Carcinogen in training set**
FCFP_6	2104062943	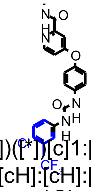 [*]C([*])([*])[c]1:[cH]:[*]:[cH]:[cH]:[c]:1Cl	-1.01	1 out of 17
FCFP_6	551850122	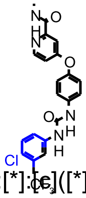 [*][c]1:[*]:[c]([*]):[c](Cl):[cH]:[cH]:1	-0.433	8 out of 49
FCFP_6	71476542	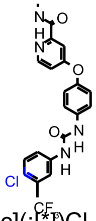 [*]:[c](:[*])Cl	-0.406	10 out of 59

Sunitinib

TOPKAT_Mouse_Male_FDA_None_vs_Carcinogen

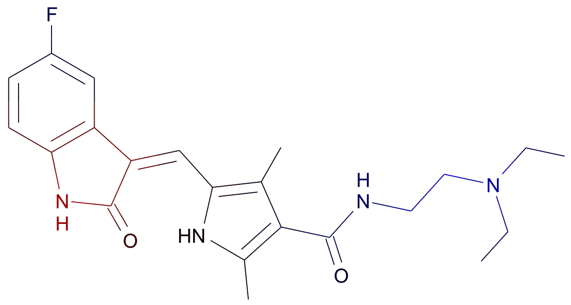

$C_{22}H_{27}FN_4O_2$
Molecular Weight: 398.47378
ALogP: 2.997
Rotatable Bonds: 7
Acceptors: 3
Donors: 3

Model Prediction

Prediction: Non-Carcinogen

Probability: 0.247

Enrichment: 0.839

Bayesian Score: -2.43

Mahalanobis Distance: 14.2

Mahalanobis Distance p-value: 2.87e-006

Prediction: Positive if the Bayesian score is above the estimated best cutoff value from minimizing the false positive and false negative rate.
Probability: The esimated probability that the sample is in the positive category. This assumes that the Bayesian score follows a normal distribution and is different from the prediction using a cutoff.
Enrichment: An estimate of enrichment, that is, the increased likelihood (versus random) of this sample being in the category.
Bayesian Score: The standard Laplacian-modified Bayesian score.
Mahalanobis Distance: The Mahalanobis distance (MD) is the distance to the center of the training data. The larger the MD, the less trustworthy the prediction.
Mahalanobis Distance p-value: The p-value gives the fraction of training data with an MD greater than or equal to the one for the given sample, assuming normally distributed data. The smaller the p-value, the less trustworthy the prediciton. For highly non-normal X properties (e.g., fingerprints), the MD p-value is wildly inaccurate.

Structural Similar Compounds

Name	Metoclopramide	Fluvastatin	Torsemide
Structure	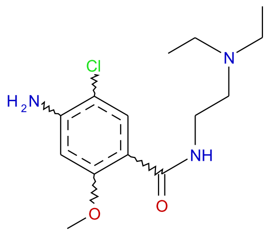	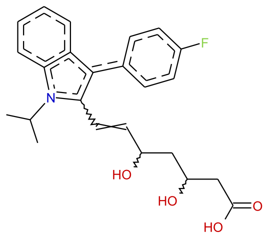	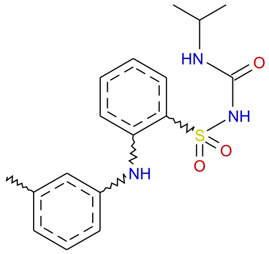
Actual Endpoint	Non-Carcinogen	Non-Carcinogen	Non-Carcinogen
Predicted Endpoint	Non-Carcinogen	Non-Carcinogen	Non-Carcinogen
Distance	0.590	0.603	0.650
Reference	US FDA (Centre for Drug Eval.& Res./Off. Testing & Res.) Sept. 1997	US FDA (Centre for Drug Eval.& Res./Off. Testing & Res.) Sept. 1997	US FDA (Centre for Drug Eval.& Res./Off. Testing & Res.) Sept. 1997

Model Applicability

Unknown features are fingerprint features in the query molecule, but not found or appearing too infrequently in the training set.

1. All properties and OPS components are within expected ranges.

Feature Contribution

Top features for positive contribution				
**Fingerprint**	**Bit/Smiles**	**Feature Structure**	**Score**	**Carcinogen in training set**
FCFP_6	1175665944	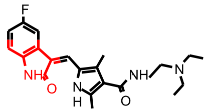 [*]C1=[*][c](:[*]):[c](NC1=O):[cH]:[*]	0.655	7 out of 12

FCFP_6	566058135	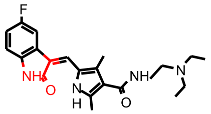 [*]NC(=O)C(=[*])[*]	0.447	17 out of 40
FCFP_6	1294255210	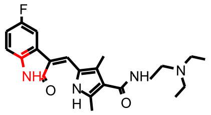 [*]C(=[*])N[c](:[*]):[*]	0.441	12 out of 28
**Top Features for negative contribution**				
**Fingerprint**	**Bit/Smiles**	**Feature Structure**	**Score**	**Carcinogen in training set**
FCFP_6	172450560	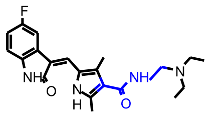 [*]CCNC(=O)[c](:[*]):[*]	-0.839	0 out of 5
FCFP_6	1851332093	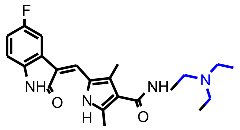 [*]CN(C[*])CC	-0.582	0 out of 3
FCFP_6	-587569116	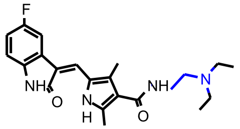 [*]CCN([*])[*]	-0.551	7 out of 49

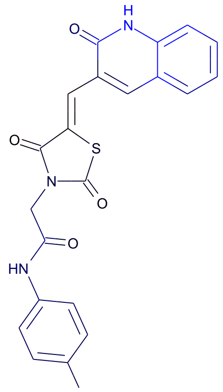

$C_{22}H_{17}N_3O_4S$
Molecular Weight: 419.45307
ALogP: 2.653
Rotatable Bonds: 4
Acceptors: 5
Donors: 2

Model Prediction

Prediction: Single-Carcinogen
Probability: 0.136
Enrichment: 0.451
Bayesian Score: -15.1
Mahalanobis Distance: 12.4
Mahalanobis Distance p-value: 0.000311

Prediction: Positive if the Bayesian score is above the estimated best cutoff value from minimizing the false positive and false negative rate.
Probability: The esimated probability that the sample is in the positive category. This assumes that the Bayesian score follows a normal distribution and is different from the prediction using a cutoff.
Enrichment: An estimate of enrichment, that is, the increased likelihood (versus random) of this sample being in the category.
Bayesian Score: The standard Laplacian-modified Bayesian score.
Mahalanobis Distance: The Mahalanobis distance (MD) is the distance to the center of the training data. The larger the MD, the less trustworthy the prediction.
Mahalanobis Distance p-value: The p-value gives the fraction of training data with an MD greater than or equal to the one for the given sample, assuming normally distributed data. The smaller the p-value, the less trustworthy the prediciton. For highly non-normal X properties (e.g., fingerprints), the MD p-value is wildly inaccurate.

Structural Similar Compounds

Name	Bicalutamide	Flunisolide	Phenolphthalein
Structure	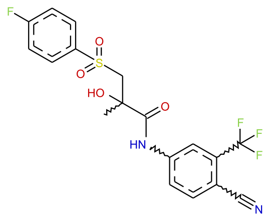	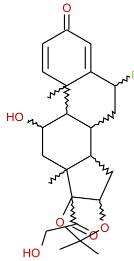	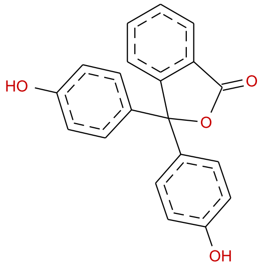
Actual Endpoint	Single-Carcinogen	Single-Carcinogen	Multiple-Carcinogen
Predicted Endpoint	Single-Carcinogen	Single-Carcinogen	Multiple-Carcinogen
Distance	0.592	0.729	0.749
Reference	US FDA (Centre for Drug Eval.& Res./Off. Testing & Res.) Sept. 1997	US FDA (Centre for Drug Eval.& Res./Off. Testing & Res.) Sept. 1997	US FDA (Centre for Drug Eval.& Res./Off. Testing & Res.) Sept. 1997

Model Applicability

Unknown features are fingerprint features in the query molecule, but not found or appearing too infrequently in the training set.

1. OPS PC12 out of range. Value: 3.8399. Training min, max, SD, explained variance: -3.4599, 2.3291, 1.246, 0.0290.

Feature Contribution

Top features for positive contribution				
**Fingerprint**	**Bit/Smiles**	**Feature Structure**	**Score**	**Multiple-Carcinogen in training set**
FCFP_12	451847724	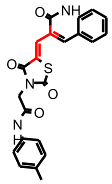 [*]C(=CC(=[*])[*])[*]	0.3	10 out of 21

FCFP_12	436886043	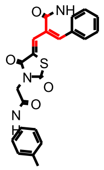 [*]\C=C(\C=[*])/C(=[*])[*]	0.27	7 out of 15
FCFP_12	565998553	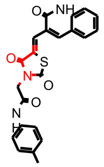 [*]N1[*][*]C(=[*])C1= O	0.194	6 out of 14

Top Features for negative contribution

**Fingerprint**	**Bit/Smiles**	**Feature Structure**	**Score**	**Multiple-Carcinogen in training set**
FCFP_12	1294255210	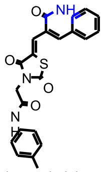 [*]C(=[*])N[c](:[*]): [*]	-1.63	0 out of 12
FCFP_12	1175665944	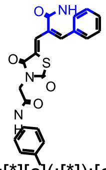 [*]C1=[*][c](:[*]):[c](NC1=O):[cH]:[*]	-1.22	0 out of 7
FCFP_12	590925877	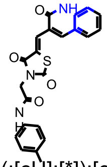 [*]N[c](:[cH]:[*]):[c]([*]):[*]	-0.998	1 out of 13

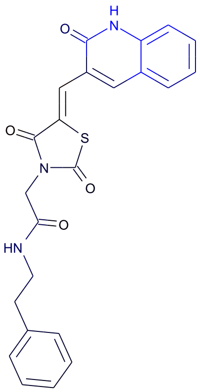

$C_{23}H_{19}N_3O_4S$

Molecular Weight: 433.47965

ALogP: 2.495

Rotatable Bonds: 6

Acceptors: 5

Donors: 2

Model Prediction

Prediction: Single-Carcinogen

Probability: 0.152

Enrichment: 0.506

Bayesian Score: -13.2

Mahalanobis Distance: 13.2

Mahalanobis Distance p-value: 6.44e-005

Prediction: Positive if the Bayesian score is above the estimated best cutoff value from minimizing the false positive and false negative rate.
Probability: The esimated probability that the sample is in the positive category. This assumes that the Bayesian score follows a normal distribution and is different from the prediction using a cutoff.
Enrichment: An estimate of enrichment, that is, the increased likelihood (versus random) of this sample being in the category.
Bayesian Score: The standard Laplacian-modified Bayesian score.
Mahalanobis Distance: The Mahalanobis distance (MD) is the distance to the center of the training data. The larger the MD, the less trustworthy the prediction.
Mahalanobis Distance p-value: The p-value gives the fraction of training data with an MD greater than or equal to the one for the given sample, assuming normally distributed data. The smaller the p-value, the less trustworthy the prediciton. For highly non-normal X properties (e.g., fingerprints), the MD p-value is wildly inaccurate.

Structural Similar Compounds

Name	Bicalutamide	Glimepride	Lansoprazole
Structure	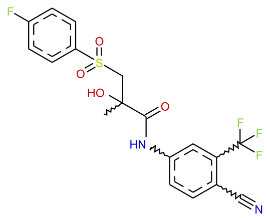	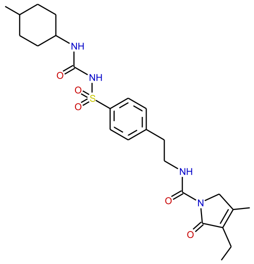	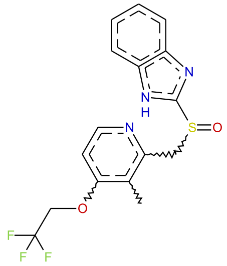
Actual Endpoint	Single-Carcinogen	Single-Carcinogen	Single-Carcinogen
Predicted Endpoint	Single-Carcinogen	Single-Carcinogen	Single-Carcinogen
Distance	0.501	0.698	0.736
Reference	US FDA (Centre for Drug Eval.& Res./Off. Testing & Res.) Sept. 1997	US FDA (Centre for Drug Eval.& Res./Off. Testing & Res.) Sept. 1997	US FDA (Centre for Drug Eval.& Res./Off. Testing & Res.) Sept. 1997

Model Applicability

Unknown features are fingerprint features in the query molecule, but not found or appearing too infrequently in the training set.

1. OPS PC12 out of range. Value: 3.3119. Training min, max, SD, explained variance: -3.4599, 2.3291, 1.246, 0.0290.

Feature Contribution

Top features for positive contribution

Fingerprint	Bit/Smiles	Feature Structure	Score	Multiple-Carcinogen in training set
FCFP_12	451847724	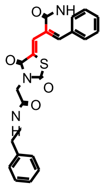 [*]C(=CC(=[*])[*])[*]	0.3	10 out of 21

FCFP_12	436886043	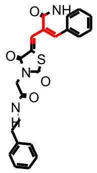 [*]\C=C(\C=[*])/C(=[*])[*]	0.27	7 out of 15
FCFP_12	907007053	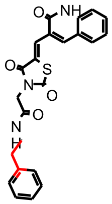 [*]CC[c](:[*]):[*]	0.235	5 out of 11
**Top Features for negative contribution**				
**Fingerprint**	**Bit/Smiles**	**Feature Structure**	**Score**	**Multiple-Carcinogen in training set**
FCFP_12	1294255210	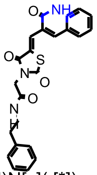 [*]C(=[*])N[c](:[*]):[*]	-1.63	0 out of 12
FCFP_12	1175665944	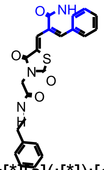 [*]C1=[*][c](:[*]):[c](NC1=O):[cH]:[*]	-1.22	0 out of 7
FCFP_12	590925877	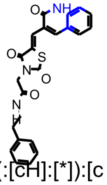 [*]N[c](:[cH]:[*]):[c]([*]):[*]	-0.998	1 out of 13

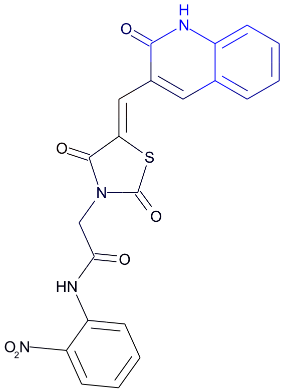

$C_{21}H_{14}N_4O_6S$
Molecular Weight: 450.42405
ALogP: 2.061
Rotatable Bonds: 5
Acceptors: 7
Donors: 2

Structural Similar Compounds

Name	Bicalutamide	Sulfasalazine	Dihydroxymethylfuratrizine
Structure	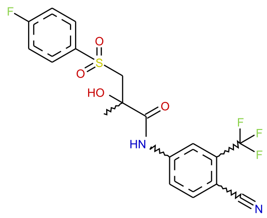	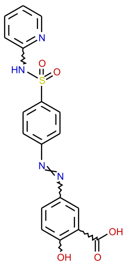	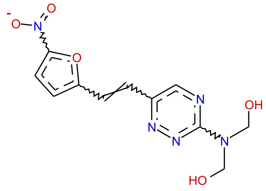
Actual Endpoint	Single-Carcinogen	Single-Carcinogen	Multiple-Carcinogen
Predicted Endpoint	Single-Carcinogen	Single-Carcinogen	Multiple-Carcinogen
Distance	0.732	0.741	0.809
Reference	US FDA (Centre for Drug Eval.& Res./Off. Testing & Res.) Sept. 1997	US FDA (Centre for Drug Eval.& Res./Off. Testing & Res.) Sept. 1997	US FDA (Centre for Drug Eval.& Res./Off. Testing & Res.) Sept. 1997

Model Prediction

Prediction: Single-Carcinogen
Probability: 0.146
Enrichment: 0.485
Bayesian Score: -10.2
Mahalanobis Distance: 11.5
Mahalanobis Distance p-value: 0.00141

Prediction: Positive if the Bayesian score is above the estimated best cutoff value from minimizing the false positive and false negative rate.
Probability: The esimated probability that the sample is in the positive category. This assumes that the Bayesian score follows a normal distribution and is different from the prediction using a cutoff.
Enrichment: An estimate of enrichment, that is, the increased likelihood (versus random) of this sample being in the category.
Bayesian Score: The standard Laplacian-modified Bayesian score.
Mahalanobis Distance: The Mahalanobis distance (MD) is the distance to the center of the training data. The larger the MD, the less trustworthy the prediction.
Mahalanobis Distance p-value: The p-value gives the fraction of training data with an MD greater than or equal to the one for the given sample, assuming normally distributed data. The smaller the p-value, the less trustworthy the prediciton. For highly non-normal X properties (e.g., fingerprints), the MD p-value is wildly inaccurate.

Model Applicability

Unknown features are fingerprint features in the query molecule, but not found or appearing too infrequently in the training set.

1. OPS PC12 out of range. Value: 3.253. Training min, max, SD, explained variance: -3.4599, 2.3291, 1.246, 0.0290.
2. Unknown FCFP_2 feature: 5: [*][O-]
3. Unknown FCFP_2 feature: -828984032: [*][c](:[*]):[c](:[cH]:[*])[N+](=[*])[*]
4. Unknown FCFP_2 feature: -1338588315: [*]:[c](:[*])[N+](=O)[O-]
5. Unknown FCFP_2 feature: 1872392852: [*][N+](=O)[*]
6. Unknown FCFP_2 feature: 260476081: [*][N+](=[*])[O-]

Feature Contribution

Top features for positive contribution				
Fingerprint	Bit/Smiles	Feature Structure	Score	Multiple-Carcinogen in training set

FCFP_12	451847724	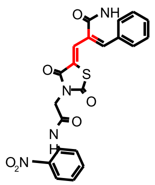<br>[*]C(=CC(=[*])[*])[*]	0.3	10 out of 21
FCFP_12	436886043	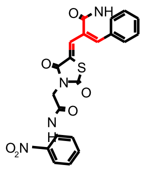<br>[*]\C=C(\C=[*])/C(=[*]<br>])[*]	0.27	7 out of 15
FCFP_12	565998553	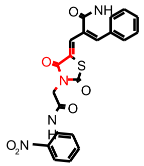<br>[*]N1[*][*]C(=[*])C1=<br>O	0.194	6 out of 14

Top Features for negative contribution

Fingerprint	Bit/Smiles	Feature Structure	Score	Multiple-Carcinogen in training set
FCFP_12	1294255210	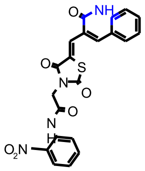<br>[*]C(=[*])N[c](:[*]):<br>[*]	-1.63	0 out of 12
FCFP_12	1175665944	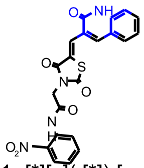<br>[*]C1=[*][c](:[*]):[c<br>](NC1=O):[cH]:[*]	-1.22	0 out of 7

FCFP_12	590925877	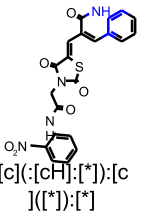 [*]N[c](:[cH]:[*]):[c]([*]):[*]	-0.998	1 out of 13

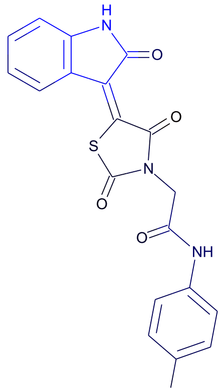

$C_{20}H_{15}N_3O_4S$

Molecular Weight: 393.4158

ALogP: 2.185

Rotatable Bonds: 3

Acceptors: 5

Donors: 2

Model Prediction

Prediction: Single-Carcinogen

Probability: 0.134

Enrichment: 0.444

Bayesian Score: -15.3

Mahalanobis Distance: 15.6

Mahalanobis Distance p-value: 7.9e-007

Prediction: Positive if the Bayesian score is above the estimated best cutoff value from minimizing the false positive and false negative rate.
Probability: The esimated probability that the sample is in the positive category. This assumes that the Bayesian score follows a normal distribution and is different from the prediction using a cutoff.
Enrichment: An estimate of enrichment, that is, the increased likelihood (versus random) of this sample being in the category.
Bayesian Score: The standard Laplacian-modified Bayesian score.
Mahalanobis Distance: The Mahalanobis distance (MD) is the distance to the center of the training data. The larger the MD, the less trustworthy the prediction.
Mahalanobis Distance p-value: The p-value gives the fraction of training data with an MD greater than or equal to the one for the given sample, assuming normally distributed data. The smaller the p-value, the less trustworthy the prediciton. For highly non-normal X properties (e.g., fingerprints), the MD p-value is wildly inaccurate.

Structural Similar Compounds

Name	Bicalutamide	Sulfamethazine	Flunisolide
Structure	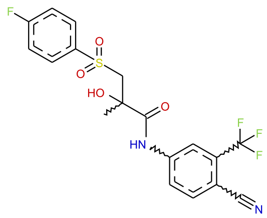	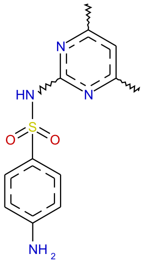	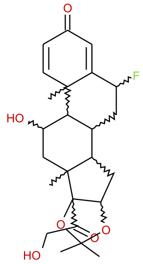
Actual Endpoint	Single-Carcinogen	Single-Carcinogen	Single-Carcinogen
Predicted Endpoint	Single-Carcinogen	Single-Carcinogen	Single-Carcinogen
Distance	0.675	0.691	0.710
Reference	US FDA (Centre for Drug Eval.& Res./Off. Testing & Res.) Sept. 1997	US FDA (Centre for Drug Eval.& Res./Off. Testing & Res.) Sept. 1997	US FDA (Centre for Drug Eval.& Res./Off. Testing & Res.) Sept. 1997

Model Applicability

Unknown features are fingerprint features in the query molecule, but not found or appearing too infrequently in the training set.

1. OPS PC12 out of range. Value: 3.6573. Training min, max, SD, explained variance: -3.4599, 2.3291, 1.246, 0.0290.

Feature Contribution

Top features for positive contribution

Fingerprint	Bit/Smiles	Feature Structure	Score	Multiple-Carcinogen in training set
FCFP_12	565998553	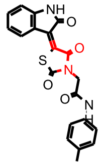 [*]N1[*][*]C(=[*])C1=O	0.194	6 out of 14

Top Features for negative contribution

Fingerprint	Bit/Smiles	Feature Structure	Score	Multiple-Carcinogen in training set
FCFP_12	1294255210	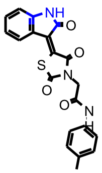 [*]C(=[*])N[c](:[*]):[*]	-1.63	0 out of 12
FCFP_12	1175665944	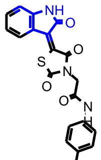 [*]C1=[*][c](:[*]):[c](NC1=O):[cH]:[*]	-1.22	0 out of 7
FCFP_12	590925877	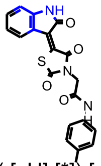 [*]N[c](:[cH]:[*]):[c]([*]):[*]	-0.998	1 out of 13

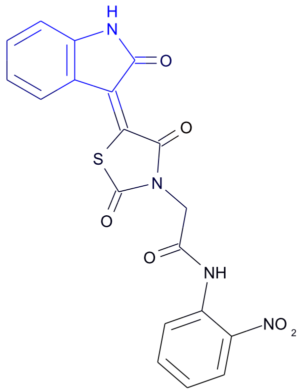

$C_{19}H_{12}N_4O_6S$
Molecular Weight: 424.38678
ALogP: 1.593
Rotatable Bonds: 4
Acceptors: 7
Donors: 2

Structural Similar Compounds

Name	Sulfasalazine	Dihydroxymethylfuratrizine	Bicalutamide
Structure	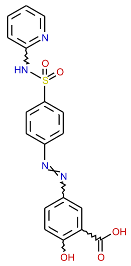	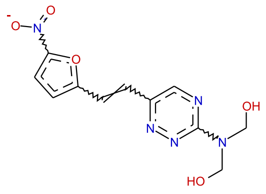	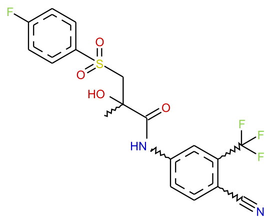
Actual Endpoint	Single-Carcinogen	Multiple-Carcinogen	Single-Carcinogen
Predicted Endpoint	Single-Carcinogen	Multiple-Carcinogen	Single-Carcinogen
Distance	0.771	0.776	0.782
Reference	US FDA (Centre for Drug Eval.& Res./Off. Testing & Res.) Sept. 1997	US FDA (Centre for Drug Eval.& Res./Off. Testing & Res.) Sept. 1997	US FDA (Centre for Drug Eval.& Res./Off. Testing & Res.) Sept. 1997

Model Prediction

Prediction: Single-Carcinogen
Probability: 0.15
Enrichment: 0.498
Bayesian Score: -11.5
Mahalanobis Distance: 13.3
Mahalanobis Distance p-value: 5.19e-005

Prediction: Positive if the Bayesian score is above the estimated best cutoff value from minimizing the false positive and false negative rate.
Probability: The esimated probability that the sample is in the positive category. This assumes that the Bayesian score follows a normal distribution and is different from the prediction using a cutoff.
Enrichment: An estimate of enrichment, that is, the increased likelihood (versus random) of this sample being in the category.
Bayesian Score: The standard Laplacian-modified Bayesian score.
Mahalanobis Distance: The Mahalanobis distance (MD) is the distance to the center of the training data. The larger the MD, the less trustworthy the prediction.
Mahalanobis Distance p-value: The p-value gives the fraction of training data with an MD greater than or equal to the one for the given sample, assuming normally distributed data. The smaller the p-value, the less trustworthy the prediciton. For highly non-normal X properties (e.g., fingerprints), the MD p-value is wildly inaccurate.

Model Applicability

Unknown features are fingerprint features in the query molecule, but not found or appearing too infrequently in the training set.

1. OPS PC12 out of range. Value: 3.0698. Training min, max, SD, explained variance: -3.4599, 2.3291, 1.246, 0.0290.
2. Unknown FCFP_2 feature: 5: [*][O-]
3. Unknown FCFP_2 feature: -828984032: [*][c](:[*]):[c](:[cH]:[*])[N+](=[*])[*]
4. Unknown FCFP_2 feature: -1338588315: [*]:[c](:[*])[N+](=O)[O-]
5. Unknown FCFP_2 feature: 1872392852: [*][N+](=O)[*]
6. Unknown FCFP_2 feature: 260476081: [*][N+](=[*])[O-]

Feature Contribution

Top features for positive contribution

Fingerprint	Bit/Smiles	Feature Structure	Score	Multiple-Carcinogen in training set

FCFP_12	565998553	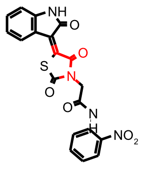 [*]N1[*][*]C(=[*])C1=O	0.194	6 out of 14
**Top Features for negative contribution**				
**Fingerprint**	**Bit/Smiles**	**Feature Structure**	**Score**	**Multiple-Carcinogen in training set**
FCFP_12	1294255210	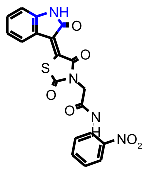 [*]C(=[*])N[c](:[*]):[*]	-1.63	0 out of 12
FCFP_12	1175665944	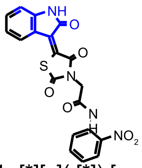 [*]C1=[*][c](:[*]):[c](NC1=O):[cH]:[*]	-1.22	0 out of 7
FCFP_12	590925877	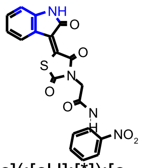 [*]N[c](:[cH]:[*]):[c]([*]):[*]	-0.998	1 out of 13

Sorafenib

TOPKAT_Mouse_Male_FDA_Single_vs_Multiple

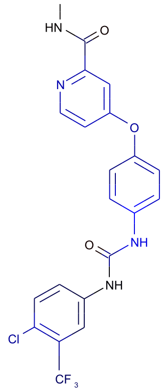

$C_{21}H_{16}ClF_3N_4O_3$
Molecular Weight: 464.82494
ALogP: 4.175
Rotatable Bonds: 6
Acceptors: 4
Donors: 3

Model Prediction

Prediction: Single-Carcinogen
Probability: 0.139
Enrichment: 0.461
Bayesian Score: -14.7
Mahalanobis Distance: 21.3
Mahalanobis Distance p-value: 4.93e-011

Prediction: Positive if the Bayesian score is above the estimated best cutoff value from minimizing the false positive and false negative rate.
Probability: The esimated probability that the sample is in the positive category. This assumes that the Bayesian score follows a normal distribution and is different from the prediction using a cutoff.
Enrichment: An estimate of enrichment, that is, the increased likelihood (versus random) of this sample being in the category.
Bayesian Score: The standard Laplacian-modified Bayesian score.
Mahalanobis Distance: The Mahalanobis distance (MD) is the distance to the center of the training data. The larger the MD, the less trustworthy the prediction.
Mahalanobis Distance p-value: The p-value gives the fraction of training data with an MD greater than or equal to the one for the given sample, assuming normally distributed data. The smaller the p-value, the less trustworthy the prediciton. For highly non-normal X properties (e.g., fingerprints), the MD p-value is wildly inaccurate.

Structural Similar Compounds

Name	Glimepride	Bicalutamide	Lansoprazole
Structure	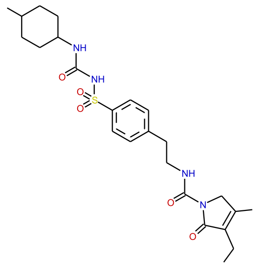	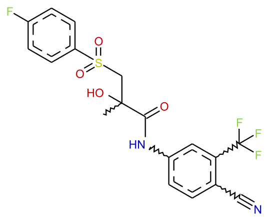	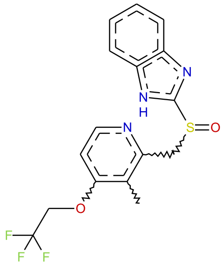
Actual Endpoint	Single-Carcinogen	Single-Carcinogen	Single-Carcinogen
Predicted Endpoint	Single-Carcinogen	Single-Carcinogen	Single-Carcinogen
Distance	0.626	0.700	0.866
Reference	US FDA (Centre for Drug Eval.& Res./Off. Testing & Res.) Sept. 1997	US FDA (Centre for Drug Eval.& Res./Off. Testing & Res.) Sept. 1997	US FDA (Centre for Drug Eval.& Res./Off. Testing & Res.) Sept. 1997

Model Applicability

Unknown features are fingerprint features in the query molecule, but not found or appearing too infrequently in the training set.

1. All properties and OPS components are within expected ranges.

Feature Contribution

Top features for positive contribution

Fingerprint	Bit/Smiles	Feature Structure	Score	Multiple-Carcinogen in training set
FCFP_12	1499521844	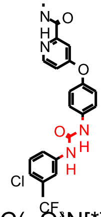 [*]NC(=O)N[*]	0.39	5 out of 9

FCFP_12	-904785030	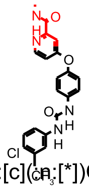 [*]:[cH]:[c](:n:[*])C (=O)NC	0.174	1 out of 2
FCFP_12	-1549103449	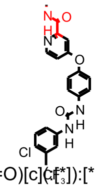 [*]NC(=O)[c](:[*]):[*]	0.168	3 out of 7

Top Features for negative contribution

Fingerprint	Bit/Smiles	Feature Structure	Score	Multiple-Carcinogen in training set
FCFP_12	1294255210	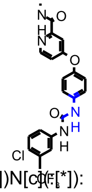 [*]C(=[*])N[c](:[*]): [*]	-1.63	0 out of 12
FCFP_12	590925877	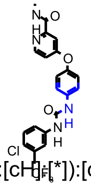 [*]N[c](:[cH]:[*]):[c]([*]):[*]	-0.998	1 out of 13
FCFP_12	-1462709112	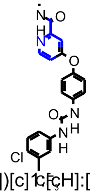 [*]C(=[*])[c]1:[cH]:[*]:[cH]:[cH]:n:1	-0.994	0 out of 5

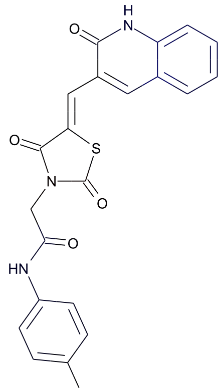

$C_{22}H_{17}N_3O_4S$
Molecular Weight: 419.45307
ALogP: 2.653
Rotatable Bonds: 4
Acceptors: 5
Donors: 2

Structural Similar Compounds

Name	1-AMINO-4-BENZOYLAMINO-ANTHRAQUINONE	5-NORBORNENE-2;3-DICARBOXYLIC ACID; 1;4;5;6;7;7-HEXACHLORO-	ANTHRAQUINONE; 1-AMINO-4-HYDROXY-2-PHENOXY-
Structure	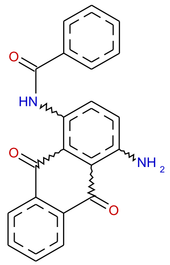	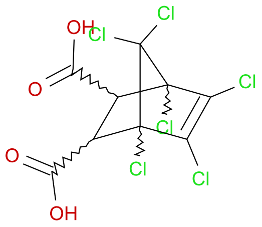	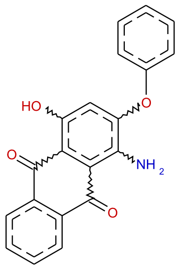
Actual Endpoint	Mild	Moderate_Severe	Mild
Predicted Endpoint	Mild	Moderate_Severe	Mild
Distance	0.624	0.626	0.641
Reference	28ZPAK-;124;72	28ZPAK-;92;72	28ZPAK 239;72

Model Prediction

Prediction: Mild
Probability: 0.761
Enrichment: 1.1
Bayesian Score: -2.23
Mahalanobis Distance: 11.1
Mahalanobis Distance p-value: 0.00352

Prediction: Positive if the Bayesian score is above the estimated best cutoff value from minimizing the false positive and false negative rate.
Probability: The esimated probability that the sample is in the positive category. This assumes that the Bayesian score follows a normal distribution and is different from the prediction using a cutoff.
Enrichment: An estimate of enrichment, that is, the increased likelihood (versus random) of this sample being in the category.
Bayesian Score: The standard Laplacian-modified Bayesian score.
Mahalanobis Distance: The Mahalanobis distance (MD) is the distance to the center of the training data. The larger the MD, the less trustworthy the prediction.
Mahalanobis Distance p-value: The p-value gives the fraction of training data with an MD greater than or equal to the one for the given sample, assuming normally distributed data. The smaller the p-value, the less trustworthy the prediciton. For highly non-normal X properties (e.g., fingerprints), the MD p-value is wildly inaccurate.

Model Applicability

Unknown features are fingerprint features in the query molecule, but not found or appearing too infrequently in the training set.

1. All properties and OPS components are within expected ranges.

Feature Contribution

Top features for positive contribution

Fingerprint	Bit/Smiles	Feature Structure	Score	Moderate_Severe in training set
FCFP_10	136120670	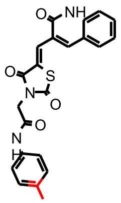 [*]:[c](:[*])C	0.206	53 out of 65

FCFP_10	3	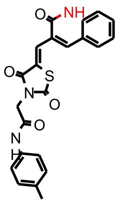 [*]N[*]	0.165	383 out of 491
FCFP_10	436886043	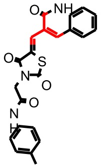 [*]\C=C(\C=[*])/C(=[*])[*]	0.16	25 out of 32
**Top Features for negative contribution**				
**Fingerprint**	**Bit/Smiles**	**Feature Structure**	**Score**	**Moderate_Severe in training set**
FCFP_10	-790336137	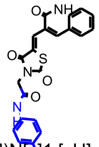 [*]C(=[*])N[c]1:[cH]:[cH]:[c](C):[cH]:[cH]:1	-0.507	0 out of 1
FCFP_10	1011367537	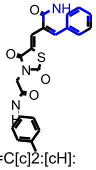 [*]=C1[*]=C[c]2:[cH]:[*]:[cH]:[cH]:[c]:2N1	-0.329	4 out of 9
FCFP_10	-773983804	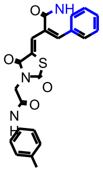 [*]N[c]1:[cH]:[cH]:[cH]:[*]:[c]:1[*]	-0.294	50 out of 102

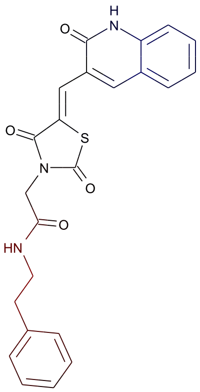

$C_{23}H_{19}N_3O_4S$
Molecular Weight: 433.47965
ALogP: 2.495
Rotatable Bonds: 6
Acceptors: 5
Donors: 2

Structural Similar Compounds

Name	5-NORBORNENE-2;3-DICARBOXYLIC ACID; 1;4;5;6;7;7-HEXACHLORO-	1-AMINO-4-BENZOYLAMINO-ANTHRAQUINONE	ANTHRAQUINONE; 1-AMINO-4-HYDROXY-2-PHENOXY-
Structure	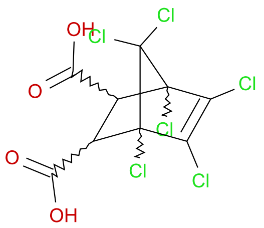	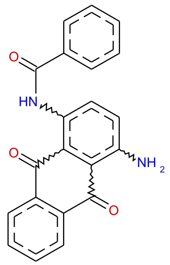	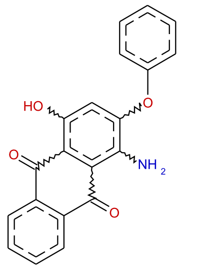
Actual Endpoint	Moderate_Severe	Mild	Mild
Predicted Endpoint	Moderate_Severe	Mild	Mild
Distance	0.662	0.665	0.689
Reference	28ZPAK-;92;72	28ZPAK-;124;72	28ZPAK 239;72

Model Prediction

Prediction: Moderate_Severe
Probability: 0.829
Enrichment: 1.2
Bayesian Score: 0.458
Mahalanobis Distance: 11.8
Mahalanobis Distance p-value: 0.000174

Prediction: Positive if the Bayesian score is above the estimated best cutoff value from minimizing the false positive and false negative rate.
Probability: The esimated probability that the sample is in the positive category. This assumes that the Bayesian score follows a normal distribution and is different from the prediction using a cutoff.
Enrichment: An estimate of enrichment, that is, the increased likelihood (versus random) of this sample being in the category.
Bayesian Score: The standard Laplacian-modified Bayesian score.
Mahalanobis Distance: The Mahalanobis distance (MD) is the distance to the center of the training data. The larger the MD, the less trustworthy the prediction.
Mahalanobis Distance p-value: The p-value gives the fraction of training data with an MD greater than or equal to the one for the given sample, assuming normally distributed data. The smaller the p-value, the less trustworthy the prediciton. For highly non-normal X properties (e.g., fingerprints), the MD p-value is wildly inaccurate.

Model Applicability

Unknown features are fingerprint features in the query molecule, but not found or appearing too infrequently in the training set.

1. All properties and OPS components are within expected ranges.

Feature Contribution

Top features for positive contribution

Fingerprint	Bit/Smiles	Feature Structure	Score	Moderate_Severe in training set
FCFP_10	-497728148	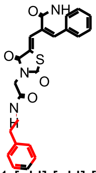 [*]CC[c]1:[cH]:[cH]:[*]:[cH]:[cH]:1	0.356	24 out of 25

FCFP_10	-547731249	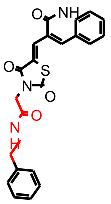<br>[*]CCNC(=O)C[*]	0.294	3 out of 3
FCFP_10	-1272709286	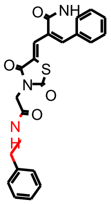<br>[*]CCN[*]	0.285	234 out of 266
**Top Features for negative contribution**				
**Fingerprint**	**Bit/Smiles**	**Feature Structure**	**Score**	**Moderate_Severe in training set**
FCFP_10	1011367537	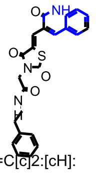<br>[*]=C1[*]=C[c]2:[cH]:[*]:[cH]:[cH]:[c]:2N1	-0.329	4 out of 9
FCFP_10	-773983804	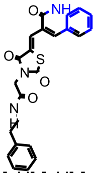<br>[*]N[c]1:[cH]:[cH]:[cH]:[*]:[c]:1[*]	-0.294	50 out of 102
FCFP_10	-1698724694	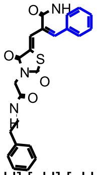<br>[*][c]1:[cH]:[cH]:[cH]:[cH]:[c]:1C=[*]	-0.284	53 out of 107

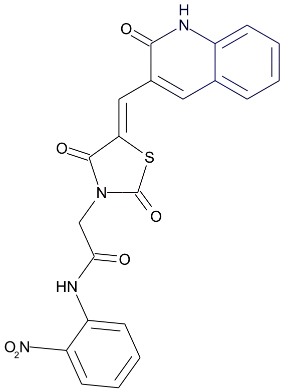

$C_{21}H_{14}N_4O_6S$
Molecular Weight: 450.42405
ALogP: 2.061
Rotatable Bonds: 5
Acceptors: 7
Donors: 2

Structural Similar Compounds

Name	4;4'-DIAMINO-1;1'-DIANTHRIMIDE	ANTHRAQUINONE; 1-AMINO-4-HYDROXY-2-PHENOXY-	2-Anthracenesulfonic acid; 9;10-dihydro-1-amino-4-bromo-9;10-dioxo-; sodium
Structure	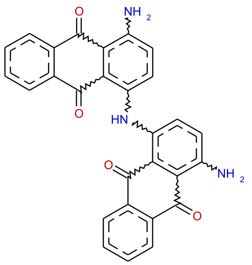	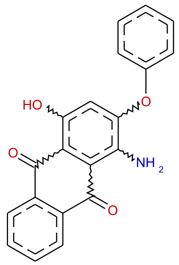	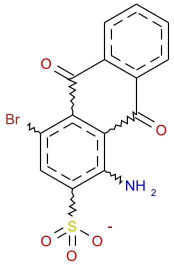
Actual Endpoint	Mild	Mild	Mild
Predicted Endpoint	Mild	Mild	Mild
Distance	0.780	0.817	0.827
Reference	28ZPAK-;125;72	28ZPAK 239;72	Prehled Prumyslove Toxikologie; Organicke Latky; Marhold; J. pp 1062;86

Model Prediction

Prediction: Mild
Probability: 0.771
Enrichment: 1.12
Bayesian Score: -1.96
Mahalanobis Distance: 11.2
Mahalanobis Distance p-value: 0.00242

Prediction: Positive if the Bayesian score is above the estimated best cutoff value from minimizing the false positive and false negative rate.
Probability: The esimated probability that the sample is in the positive category. This assumes that the Bayesian score follows a normal distribution and is different from the prediction using a cutoff.
Enrichment: An estimate of enrichment, that is, the increased likelihood (versus random) of this sample being in the category.
Bayesian Score: The standard Laplacian-modified Bayesian score.
Mahalanobis Distance: The Mahalanobis distance (MD) is the distance to the center of the training data. The larger the MD, the less trustworthy the prediction.
Mahalanobis Distance p-value: The p-value gives the fraction of training data with an MD greater than or equal to the one for the given sample, assuming normally distributed data. The smaller the p-value, the less trustworthy the prediciton. For highly non-normal X properties (e.g., fingerprints), the MD p-value is wildly inaccurate.

Model Applicability

Unknown features are fingerprint features in the query molecule, but not found or appearing too infrequently in the training set.

1. All properties and OPS components are within expected ranges.
2. Unknown FCFP_2 feature: -828984032: [*][c](:[*]):[c](:[cH]:[*])[N+](=[*])[*]
3. Unknown FCFP_2 feature: -1338588315: [*]:[c](:[*])[N+](=O)[O-]
4. Unknown FCFP_2 feature: 1872392852: [*][N+](=O)[*]
5. Unknown FCFP_2 feature: 260476081: [*][N+](=[*])[O-]

Feature Contribution

Top features for positive contribution

Fingerprint	Bit/Smiles	Feature Structure	Score	Moderate_Severe in training set

FCFP_10	3	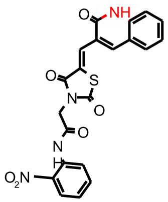 [*]N[*]	0.165	383 out of 491
FCFP_10	436886043	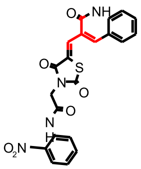 [*]\C=C(\C=[*])/C(=[*])[*]	0.16	25 out of 32
FCFP_10	-1553874037	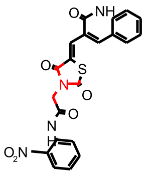 [*]CN1C(=[*])[*][*]C1 =[*]	0.107	31 out of 42

Top Features for negative contribution

**Fingerprint**	**Bit/Smiles**	**Feature Structure**	**Score**	**Moderate_Severe in training set**
FCFP_10	1011367537	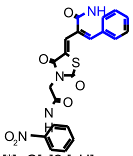 [*]=C1[*]=C[c]2:[cH]: [*]:[cH]:[cH]:[c]:2N 1	-0.329	4 out of 9
FCFP_10	-773983804	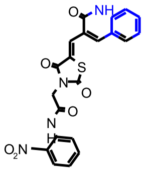 [*]N[c]1:[cH]:[cH]:[c H]:[*]:[c]:1[*]	-0.294	50 out of 102

FCFP_10	-1698724694	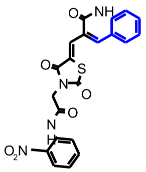 [*][c]1:[cH]:[cH]:[cH]:[cH]:[c]:1C=[*]	-0.284	53 out of 107

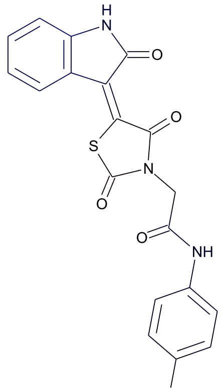

$C_{20}H_{15}N_3O_4S$
Molecular Weight: 393.4158
ALogP: 2.185
Rotatable Bonds: 3
Acceptors: 5
Donors: 2

Structural Similar Compounds

Name	1-AMINO-4-BENZOYLAMINO-ANTHRAQUINONE	5-NORBORNENE-2;3-DICARBOXYLIC ACID; 1;4;5;6;7;7-HEXACHLORO-	ANTHRAQUINONE; 1-AMINO-4-HYDROXY-2-PHENOXY-
Structure	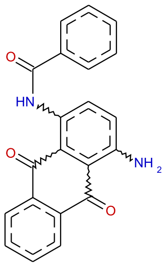	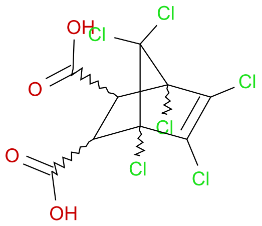	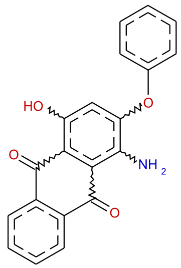
Actual Endpoint	Mild	Moderate_Severe	Mild
Predicted Endpoint	Mild	Moderate_Severe	Mild
Distance	0.606	0.630	0.631
Reference	28ZPAK-;124;72	28ZPAK-;92;72	28ZPAK 239;72

Model Prediction

Prediction: Mild
Probability: 0.753
Enrichment: 1.09
Bayesian Score: -2.44
Mahalanobis Distance: 10.4
Mahalanobis Distance p-value: 0.0373

Prediction: Positive if the Bayesian score is above the estimated best cutoff value from minimizing the false positive and false negative rate.
Probability: The esimated probability that the sample is in the positive category. This assumes that the Bayesian score follows a normal distribution and is different from the prediction using a cutoff.
Enrichment: An estimate of enrichment, that is, the increased likelihood (versus random) of this sample being in the category.
Bayesian Score: The standard Laplacian-modified Bayesian score.
Mahalanobis Distance: The Mahalanobis distance (MD) is the distance to the center of the training data. The larger the MD, the less trustworthy the prediction.
Mahalanobis Distance p-value: The p-value gives the fraction of training data with an MD greater than or equal to the one for the given sample, assuming normally distributed data. The smaller the p-value, the less trustworthy the prediciton. For highly non-normal X properties (e.g., fingerprints), the MD p-value is wildly inaccurate.

Model Applicability

Unknown features are fingerprint features in the query molecule, but not found or appearing too infrequently in the training set.

1. All properties and OPS components are within expected ranges.

Feature Contribution

Top features for positive contribution

Fingerprint	Bit/Smiles	Feature Structure	Score	Moderate_Severe in training set
FCFP_10	136120670	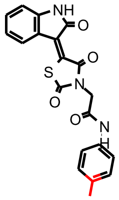 [*]:[c](:[*])C	0.206	53 out of 65

FCFP_10	3	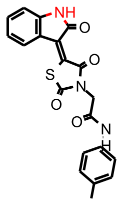 [*]N[*]	0.165	383 out of 491
FCFP_10	-1773728142	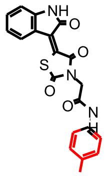 C[c]1:[cH]:[cH]:[*]:[cH]:[cH]:1	0.142	23 out of 30

Top Features for negative contribution

Fingerprint	Bit/Smiles	Feature Structure	Score	Moderate_Severe in training set
FCFP_10	-790336137	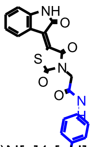 [*]C(=[*])N[c]1:[cH]:[cH]:[c](C):[cH]:[cH]:1	-0.507	0 out of 1
FCFP_10	1011367537	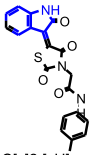 [*]=C1[*]=C[c]2:[cH]:[*]:[cH]:[cH]:[c]:2N1	-0.329	4 out of 9
FCFP_10	-773983804	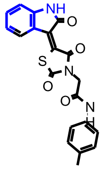 [*]N[c]1:[cH]:[cH]:[cH]:[*]:[c]:1[*]	-0.294	50 out of 102

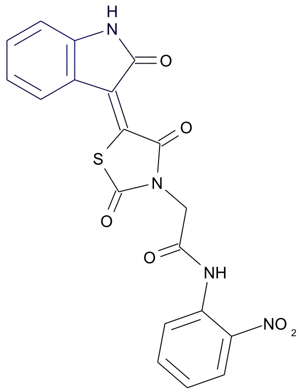

$C_{19}H_{12}N_4O_6S$
Molecular Weight: 424.38678
ALogP: 1.593
Rotatable Bonds: 4
Acceptors: 7
Donors: 2

Structural Similar Compounds

Name	2-Anthracenesulfonic acid; 9;10-dihydro-1-amino-4-bromo-9;10-dioxo-; sodium	2-NAPHTHALENESULFONIC ACID;5-AMINO-6-ETHOXY-	2;2'-Stilbenedisulfonic acid; 4-amino-4'-nitro-
Structure	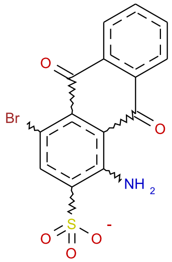	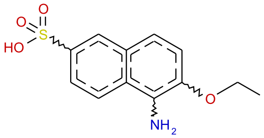	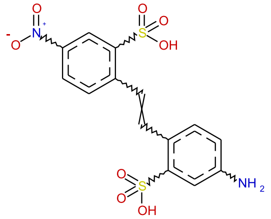
Actual Endpoint	Mild	Moderate_Severe	Mild
Predicted Endpoint	Mild	Mild	Mild
Distance	0.773	0.797	0.800
Reference	Prehled Prumyslove Toxikologie; Organicke Latky; Marhold; J. pp 1062;86	28ZPAK-;191;72	Prehled Prumyslove Toxikologie; Organicke Latky; Marhold; J. pp 1062;86

Model Prediction

Prediction: Mild
Probability: 0.775
Enrichment: 1.12
Bayesian Score: -1.85
Mahalanobis Distance: 10.5
Mahalanobis Distance p-value: 0.024

Prediction: Positive if the Bayesian score is above the estimated best cutoff value from minimizing the false positive and false negative rate.
Probability: The esimated probability that the sample is in the positive category. This assumes that the Bayesian score follows a normal distribution and is different from the prediction using a cutoff.
Enrichment: An estimate of enrichment, that is, the increased likelihood (versus random) of this sample being in the category.
Bayesian Score: The standard Laplacian-modified Bayesian score.
Mahalanobis Distance: The Mahalanobis distance (MD) is the distance to the center of the training data. The larger the MD, the less trustworthy the prediction.
Mahalanobis Distance p-value: The p-value gives the fraction of training data with an MD greater than or equal to the one for the given sample, assuming normally distributed data. The smaller the p-value, the less trustworthy the prediciton. For highly non-normal X properties (e.g., fingerprints), the MD p-value is wildly inaccurate.

Model Applicability

Unknown features are fingerprint features in the query molecule, but not found or appearing too infrequently in the training set.

1. All properties and OPS components are within expected ranges.
2. Unknown FCFP_2 feature: -828984032: [*][c](:[*]):[c](:[cH]:[*])[N+](=[*])[*]
3. Unknown FCFP_2 feature: -1338588315: [*]:[c](:[*])[N+](=O)[O-]
4. Unknown FCFP_2 feature: 1872392852: [*][N+](=O)[*]
5. Unknown FCFP_2 feature: 260476081: [*][N+](=[*])[O-]

Feature Contribution

Top features for positive contribution

Fingerprint	Bit/Smiles	Feature Structure	Score	Moderate_Severe in training set

FCFP_10	3	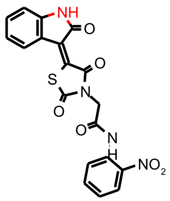 [*]N[*]	0.165	383 out of 491
FCFP_10	-1553874037	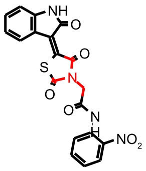 [*]CN1C(=[*])[*][*]C1=[*]	0.107	31 out of 42
FCFP_10	-568981285	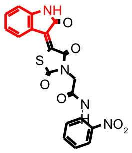 [*]=C1[*]=C[c]2:[cH]:[cH]:[cH]:[cH]:[c]:2N1	0.0934	3 out of 4

Top Features for negative contribution

Fingerprint	Bit/Smiles	Feature Structure	Score	Moderate_Severe in training set
FCFP_10	1011367537	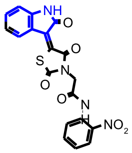 [*]=C1[*]=C[c]2:[cH]:[*]:[cH]:[cH]:[c]:2N1	-0.329	4 out of 9
FCFP_10	-773983804	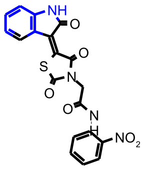 [*]N[c]1:[cH]:[cH]:[cH]:[*]:[c]:1[*]	-0.294	50 out of 102

FCFP_10	-1698724694	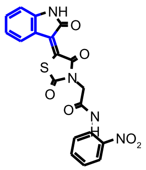 [*][c]1:[cH]:[cH]:[cH]:[cH]:[c]:1C=[*]	-0.284	53 out of 107

Sorafenib

TOPKAT_Ocular_Irritancy_Mild_vs_Moderate_Severe

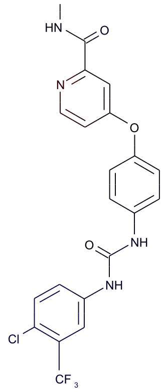

$C_{21}H_{16}ClF_3N_4O_3$
Molecular Weight: 464.82494
ALogP: 4.175
Rotatable Bonds: 6
Acceptors: 4
Donors: 3

Structural Similar Compounds

Name	4;4'-DIAMINO-1;1'-DIANTHRIMIDE	5-NORBORNENE-2;3-DICARBOXYLIC ACID; 1;4;5;6;7;7-HEXACHLORO-	METHANE;TRIS(4-AMINOPHENYL)-
Structure	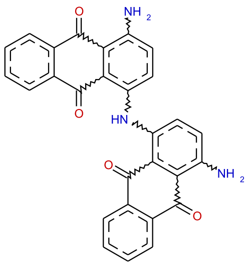	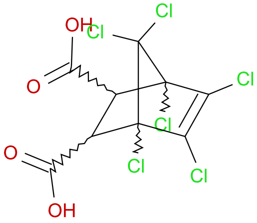	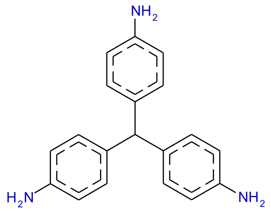
Actual Endpoint	Mild	Moderate_Severe	Moderate_Severe
Predicted Endpoint	Mild	Moderate_Severe	Moderate_Severe
Distance	0.799	0.816	0.827
Reference	28ZPAK-;125;72	28ZPAK-;92;72	28ZPAK-;73;72

Model Prediction

Prediction: Mild
Probability: 0.776
Enrichment: 1.13
Bayesian Score: -1.8
Mahalanobis Distance: 8.95
Mahalanobis Distance p-value: 0.537

Prediction: Positive if the Bayesian score is above the estimated best cutoff value from minimizing the false positive and false negative rate.
Probability: The esimated probability that the sample is in the positive category. This assumes that the Bayesian score follows a normal distribution and is different from the prediction using a cutoff.
Enrichment: An estimate of enrichment, that is, the increased likelihood (versus random) of this sample being in the category.
Bayesian Score: The standard Laplacian-modified Bayesian score.
Mahalanobis Distance: The Mahalanobis distance (MD) is the distance to the center of the training data. The larger the MD, the less trustworthy the prediction.
Mahalanobis Distance p-value: The p-value gives the fraction of training data with an MD greater than or equal to the one for the given sample, assuming normally distributed data. The smaller the p-value, the less trustworthy the prediciton. For highly non-normal X properties (e.g., fingerprints), the MD p-value is wildly inaccurate.

Model Applicability

Unknown features are fingerprint features in the query molecule, but not found or appearing too infrequently in the training set.

1. All properties and OPS components are within expected ranges.

Feature Contribution

Top features for positive contribution

Fingerprint	Bit/Smiles	Feature Structure	Score	Moderate_Severe in training set
FCFP_10	-1695756380	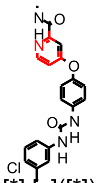 [*][c]1:[*]:[c]([*]):n:[cH]:[cH]:1	0.285	10 out of 11

FCFP_10	-124655670	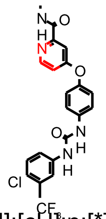 [*]:[cH]:[cH]:n:[*]	0.259	14 out of 16
FCFP_10	-885550502	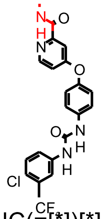 [*]CNC(=[*])[*]	0.239	54 out of 64
**Top Features for negative contribution**				
**Fingerprint**	**Bit/Smiles**	**Feature Structure**	**Score**	**Moderate_Severe in training set**
FCFP_10	2104062943	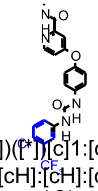 [*]C([*])([*])[c]1:[cH]:[*]:[cH]:[cH]:[c]:1Cl	-0.745	7 out of 24
FCFP_10	-174293376	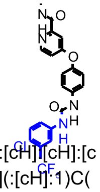 [*]N[c]1:[cH]:[cH]:[c](Cl):[c](:[cH]:1)C([*])([*])[*]	-0.507	0 out of 1
FCFP_10	-1549103449	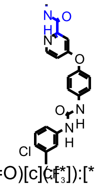 [*]NC(=O)[c](:[*]):[*]	-0.504	2 out of 6

Sunitinib

TOPKAT_Ocular_Irritancy_Mild_vs_Moderate_Severe

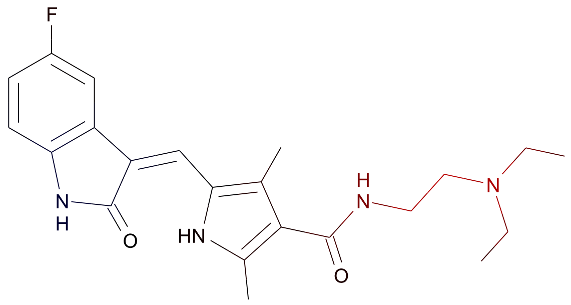

$C_{22}H_{27}FN_4O_2$

Molecular Weight: 398.47378

ALogP: 2.997

Rotatable Bonds: 7

Acceptors: 3

Donors: 3

Model Prediction

Prediction: Moderate_Severe

Probability: 0.893

Enrichment: 1.3

Bayesian Score: 3.24

Mahalanobis Distance: 11.1

Mahalanobis Distance p-value: 0.00304

Prediction: Positive if the Bayesian score is above the estimated best cutoff value from minimizing the false positive and false negative rate.
Probability: The esimated probability that the sample is in the positive category. This assumes that the Bayesian score follows a normal distribution and is different from the prediction using a cutoff.
Enrichment: An estimate of enrichment, that is, the increased likelihood (versus random) of this sample being in the category.
Bayesian Score: The standard Laplacian-modified Bayesian score.
Mahalanobis Distance: The Mahalanobis distance (MD) is the distance to the center of the training data. The larger the MD, the less trustworthy the prediction.
Mahalanobis Distance p-value: The p-value gives the fraction of training data with an MD greater than or equal to the one for the given sample, assuming normally distributed data. The smaller the p-value, the less trustworthy the prediciton. For highly non-normal X properties (e.g., fingerprints), the MD p-value is wildly inaccurate.

Structural Similar Compounds

Name	METHANE;TRIS(4-AMINOPHENYL)-	ANTHRAQUINONE; 1-((2-HYDROXYETHYL)AMINO)-4-(METHYLAMINO)-	1-AMINO-4-BENZOYLAMINO-ANTHRAQUINONE
Structure	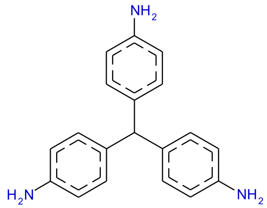	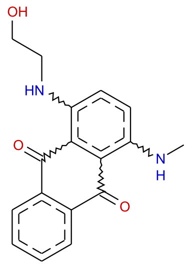	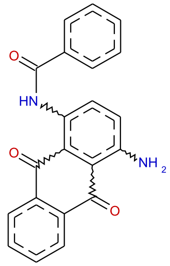
Actual Endpoint	Moderate_Severe	Mild	Mild
Predicted Endpoint	Moderate_Severe	Mild	Mild
Distance	0.713	0.770	0.786
Reference	28ZPAK-;73;72	28ZPAK 245;72	28ZPAK-;124;72

Model Applicability

Unknown features are fingerprint features in the query molecule, but not found or appearing too infrequently in the training set.

1. All properties and OPS components are within expected ranges.
2. Unknown FCFP_2 feature: 203707511: [*][c]1:[*]:[*]:[nH]:[c]:1C

Feature Contribution

Top features for positive contribution

Fingerprint	Bit/Smiles	Feature Structure	Score	Moderate_Severe in training set
FCFP_10	1851332093	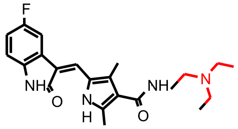 [*]CN(C[*])CC	0.376	12 out of 12

FCFP_10	-371808660	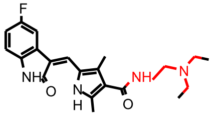 [*]CN(C[*])CCN[*]	0.338	18 out of 19
FCFP_10	-587569116	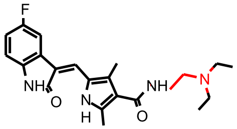 [*]CCN([*])[*]	0.335	66 out of 71
**Top Features for negative contribution**				
**Fingerprint**	**Bit/Smiles**	**Feature Structure**	**Score**	**Moderate_Severe in training set**
FCFP_10	-1549103449	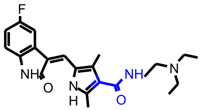 [*]NC(=O)[c](:[*]):[*]	-0.504	2 out of 6
FCFP_10	1011367537	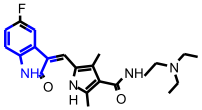 [*]=C1[*]=C[c]2:[cH]:[*]:[cH]:[cH]:[c]:2N1	-0.329	4 out of 9
FCFP_10	-773983804	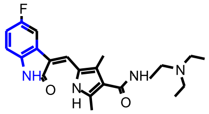 [*]N[c]1:[cH]:[cH]:[cH]:[*]:[c]:1[*]	-0.294	50 out of 102

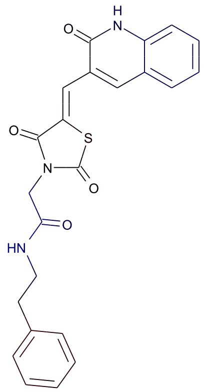

$C_{23}H_{19}N_3O_4S$

Molecular Weight: 433.47965

ALogP: 2.495

Rotatable Bonds: 6

Acceptors: 5

Donors: 2

Model Prediction

Prediction: Moderate

Probability: 0.551

Enrichment: 0.888

Bayesian Score: -3.22

Mahalanobis Distance: 12.1

Mahalanobis Distance p-value: 7.95e-006

Prediction: Positive if the Bayesian score is above the estimated best cutoff value from minimizing the false positive and false negative rate.
Probability: The esimated probability that the sample is in the positive category. This assumes that the Bayesian score follows a normal distribution and is different from the prediction using a cutoff.
Enrichment: An estimate of enrichment, that is, the increased likelihood (versus random) of this sample being in the category.
Bayesian Score: The standard Laplacian-modified Bayesian score.
Mahalanobis Distance: The Mahalanobis distance (MD) is the distance to the center of the training data. The larger the MD, the less trustworthy the prediction.
Mahalanobis Distance p-value: The p-value gives the fraction of training data with an MD greater than or equal to the one for the given sample, assuming normally distributed data. The smaller the p-value, the less trustworthy the prediciton. For highly non-normal X properties (e.g., fingerprints), the MD p-value is wildly inaccurate.

Structural Similar Compounds

Name	5-NORBORNENE-2;3-DICARBOXYLIC ACID; 1;4;5;6;7;7-HEXACHLORO-	COLCHICINE	2-NAPHTHALENESULFONIC ACID;5-AMINO-6-ETHOXY-
Structure	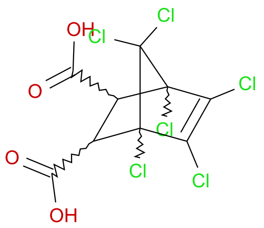	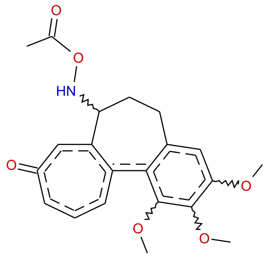	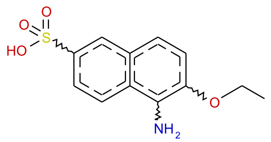
Actual Endpoint	Severe	Severe	Moderate
Predicted Endpoint	Severe	Severe	Moderate
Distance	0.653	0.807	0.823
Reference	28ZPAK-;92;72	AJOPAA 31;837;48	28ZPAK-;191;72

Model Applicability

Unknown features are fingerprint features in the query molecule, but not found or appearing too infrequently in the training set.

1. OPS PC11 out of range. Value: -4.1613. Training min, max, SD, explained variance: -3.5735, 4.44, 1.244, 0.0204.

Feature Contribution

Top features for positive contribution

Fingerprint	Bit/Smiles	Feature Structure	Score	Severe in training set
SCFP_12	-1640858361	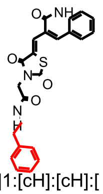 [*]CC[c]1:[cH]:[cH]:[cH]:[cH]:[cH]:1	0.376	4 out of 4

SCFP_12	-1272709286	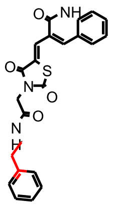 [*]CC[c](:[*]):[*]	0.231	24 out of 31
SCFP_12	2102703671	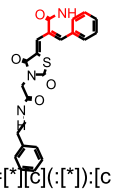 [*]C1=[*][c](:[*]):[c](NC1=O):[cH]:[*]	0.218	1 out of 1

Top Features for negative contribution

**Fingerprint**	**Bit/Smiles**	**Feature Structure**	**Score**	**Severe in training set**
SCFP_12	2005026407	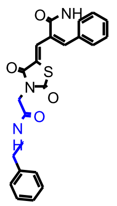 [*]CCNC(=O)C[*]	-0.796	0 out of 2
SCFP_12	1655488245	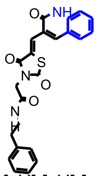 [*]N[c]1:[cH]:[cH]:[c H]:[cH]:[c]:1[*]	-0.796	0 out of 2
SCFP_12	-587569116	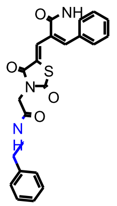 [*]CCN[*]	-0.619	11 out of 35

Sunitinib

TOPKAT_Ocular_Irritancy_Moderate_vs_Severe

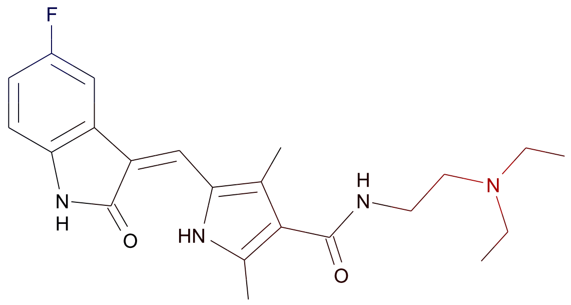

$C_{22}H_{27}FN_4O_2$

Molecular Weight: 398.47378

ALogP: 2.997

Rotatable Bonds: 7

Acceptors: 3

Donors: 3

Structural Similar Compounds

Name	METHANE;TRIS(4-AMINOPHENYL)-	5-NORBORNENE-2;3-DICARBOXYLIC ACID; 1;4;5;6;7;7-HEXACHLORO-	FLUORENE-9;9-(BIS)PROPYLAMINE
Structure	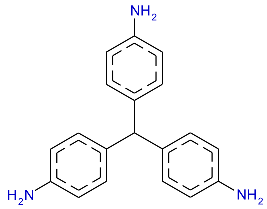	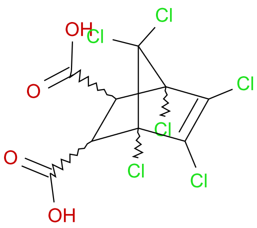	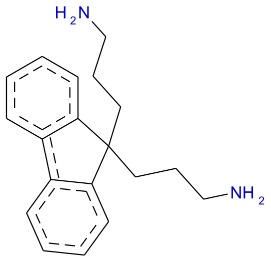
Actual Endpoint	Moderate	Severe	Severe
Predicted Endpoint	Moderate	Severe	Severe
Distance	0.726	0.793	0.804
Reference	28ZPAK-;73;72	28ZPAK-;92;72	IHFCAY 6;1;67

Model Prediction

Prediction: Severe

Probability: 0.714

Enrichment: 1.15

Bayesian Score: 0.743

Mahalanobis Distance: 11.7

Mahalanobis Distance p-value: 7.58e-005

Prediction: Positive if the Bayesian score is above the estimated best cutoff value from minimizing the false positive and false negative rate.
Probability: The esimated probability that the sample is in the positive category. This assumes that the Bayesian score follows a normal distribution and is different from the prediction using a cutoff.
Enrichment: An estimate of enrichment, that is, the increased likelihood (versus random) of this sample being in the category.
Bayesian Score: The standard Laplacian-modified Bayesian score.
Mahalanobis Distance: The Mahalanobis distance (MD) is the distance to the center of the training data. The larger the MD, the less trustworthy the prediction.
Mahalanobis Distance p-value: The p-value gives the fraction of training data with an MD greater than or equal to the one for the given sample, assuming normally distributed data. The smaller the p-value, the less trustworthy the prediciton. For highly non-normal X properties (e.g., fingerprints), the MD p-value is wildly inaccurate.

Model Applicability

Unknown features are fingerprint features in the query molecule, but not found or appearing too infrequently in the training set.

1. All properties and OPS components are within expected ranges.

Feature Contribution

Top features for positive contribution				
Fingerprint	Bit/Smiles	Feature Structure	Score	Severe in training set
SCFP_12	1725890097	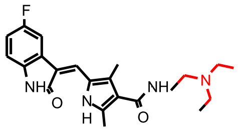 [*]CN(C[*])CC	0.449	12 out of 12

SCFP_12	2088704928	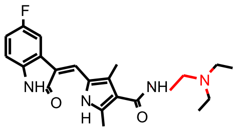 [*]CCN([*])[*]	0.342	110 out of 128
SCFP_12	5	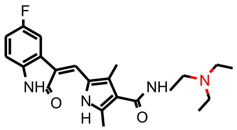 [*]N([*])[*]	0.337	129 out of 151

Top Features for negative contribution

Fingerprint	Bit/Smiles	Feature Structure	Score	Severe in training set
SCFP_12	-587569116	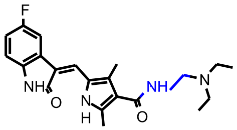 [*]CCN[*]	-0.619	11 out of 35
SCFP_12	-1794884847	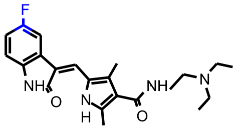 [*]:[c](:[*])F	-0.345	1 out of 3
SCFP_12	-1381307546	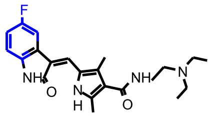 [*][c]1:[*]:[cH]:[c](F):[cH]:[cH]:1	-0.345	1 out of 3

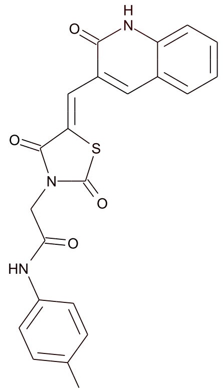

$C_{22}H_{17}N_3O_4S$
Molecular Weight: 419.45307
ALogP: 2.653
Rotatable Bonds: 4
Acceptors: 5
Donors: 2

Model Prediction

Prediction: Irritant
Probability: 1
Enrichment: 1.18
Bayesian Score: 1.88
Mahalanobis Distance: 10.9
Mahalanobis Distance p-value: 0.0056

Prediction: Positive if the Bayesian score is above the estimated best cutoff value from minimizing the false positive and false negative rate.
Probability: The esimated probability that the sample is in the positive category. This assumes that the Bayesian score follows a normal distribution and is different from the prediction using a cutoff.
Enrichment: An estimate of enrichment, that is, the increased likelihood (versus random) of this sample being in the category.
Bayesian Score: The standard Laplacian-modified Bayesian score.
Mahalanobis Distance: The Mahalanobis distance (MD) is the distance to the center of the training data. The larger the MD, the less trustworthy the prediction.
Mahalanobis Distance p-value: The p-value gives the fraction of training data with an MD greater than or equal to the one for the given sample, assuming normally distributed data. The smaller the p-value, the less trustworthy the prediciton. For highly non-normal X properties (e.g., fingerprints), the MD p-value is wildly inaccurate.

Structural Similar Compounds

Name	1-AMINO-4-BENZOYLAMINO-ANTHRAQUINONE	5-NORBORNENE-2;3-DICARBOXYLIC ACID; 1;4;5;6;7;7-HEXACHLORO-	ANTHRAQUINONE; 1-AMINO-4-HYDROXY-2-PHENOXY-
Structure	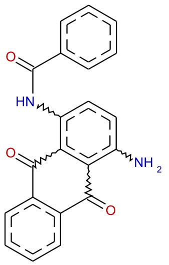	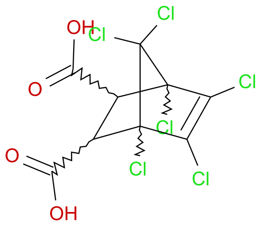	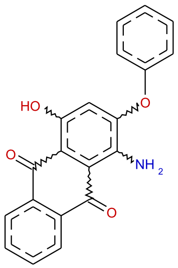
Actual Endpoint	Irritant	Irritant	Irritant
Predicted Endpoint	Irritant	Irritant	Irritant
Distance	0.620	0.622	0.632
Reference	28ZPAK-;124;72	28ZPAK-;92;72	28ZPAK 239;72

Model Applicability

Unknown features are fingerprint features in the query molecule, but not found or appearing too infrequently in the training set.

1. All properties and OPS components are within expected ranges.

Feature Contribution

Top features for positive contribution

Fingerprint	Bit/Smiles	Feature Structure	Score	Irritant in training set
FCFP_12	1175665944	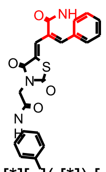 [*]C1=[*][c](:[*]):[c](NC1=O):[cH]:[*]	0.198	14 out of 14

FCFP_12	-568981285	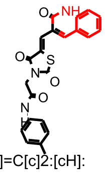 [*]=C1[*]=C[c]2:[cH]:[cH]:[cH]:[cH]:[c]:2N1	0.167	4 out of 4
FCFP_12	436915834	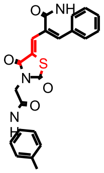 [*]\C=C\1/S[*][*]C1=[*]	0.167	4 out of 4

Top Features for negative contribution

Fingerprint	Bit/Smiles	Feature Structure	Score	Irritant in training set
FCFP_12	451371068	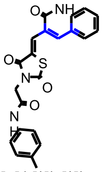 [*]C(=C[c](:[*]):[*])[*]	-0.167	6 out of 9
FCFP_12	-1698724694	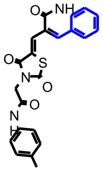 [*][c]1:[cH]:[cH]:[cH]:[cH]:[c]:1C=[*]	-0.0964	107 out of 146
FCFP_12	565998553	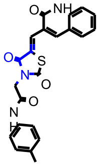 [*]N1[*][*]C(=[*])C1=O	-0.0662	198 out of 262

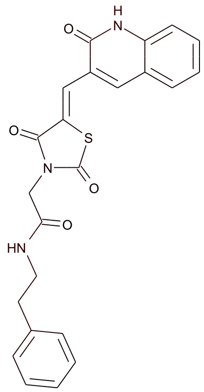

$C_{23}H_{19}N_3O_4S$
Molecular Weight: 433.47965
ALogP: 2.495
Rotatable Bonds: 6
Acceptors: 5
Donors: 2

Model Prediction

Prediction: Irritant
Probability: 1
Enrichment: 1.18
Bayesian Score: 1.93
Mahalanobis Distance: 11.5
Mahalanobis Distance p-value: 0.000737

Prediction: Positive if the Bayesian score is above the estimated best cutoff value from minimizing the false positive and false negative rate.
Probability: The esimated probability that the sample is in the positive category. This assumes that the Bayesian score follows a normal distribution and is different from the prediction using a cutoff.
Enrichment: An estimate of enrichment, that is, the increased likelihood (versus random) of this sample being in the category.
Bayesian Score: The standard Laplacian-modified Bayesian score.
Mahalanobis Distance: The Mahalanobis distance (MD) is the distance to the center of the training data. The larger the MD, the less trustworthy the prediction.
Mahalanobis Distance p-value: The p-value gives the fraction of training data with an MD greater than or equal to the one for the given sample, assuming normally distributed data. The smaller the p-value, the less trustworthy the prediciton. For highly non-normal X properties (e.g., fingerprints), the MD p-value is wildly inaccurate.

Structural Similar Compounds

Name	5-NORBORNENE-2;3-DICARBOXYLIC ACID; 1;4;5;6;7;7-HEXACHLORO-	1-AMINO-4-BENZOYLAMINO-ANTHRAQUINONE	ANTHRAQUINONE; 1-AMINO-4-HYDROXY-2-PHENOXY-
Structure	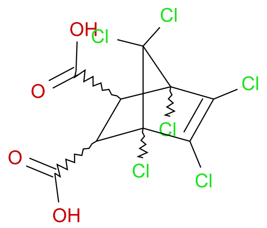	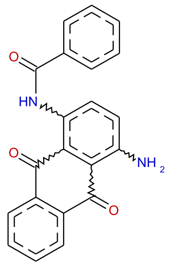	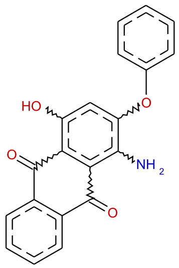
Actual Endpoint	Irritant	Irritant	Irritant
Predicted Endpoint	Irritant	Irritant	Irritant
Distance	0.652	0.657	0.675
Reference	28ZPAK-;92;72	28ZPAK-;124;72	28ZPAK 239;72

Model Applicability

Unknown features are fingerprint features in the query molecule, but not found or appearing too infrequently in the training set.

1. All properties and OPS components are within expected ranges.

Feature Contribution

Top features for positive contribution

Fingerprint	Bit/Smiles	Feature Structure	Score	Irritant in training set
FCFP_12	1175665944	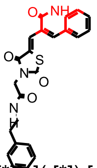 [*]C1=[*][c](:[*]):[c](NC1=O):[cH]:[*]	0.198	14 out of 14

FCFP_12	-885550502	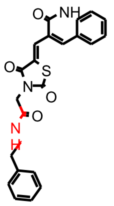 [*]CNC(=[*])[*]	0.18	64 out of 66
FCFP_12	436915834	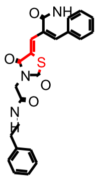 [*]\C=C\1/S[*][*]C1=[*]	0.167	4 out of 4

Top Features for negative contribution

Fingerprint	Bit/Smiles	Feature Structure	Score	Irritant in training set
FCFP_12	451371068	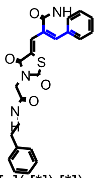 [*]C(=C[c](:[*]):[*]) [*]	-0.167	6 out of 9
FCFP_12	1981711554	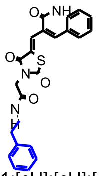 [*]CC[c]1:[cH]:[cH]:[cH]:[cH]:[cH]:1	-0.103	5 out of 7
FCFP_12	-1698724694	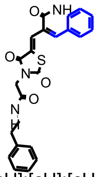 [*][c]1:[cH]:[cH]:[cH]:[cH]:[c]:1C=[*]	-0.0964	107 out of 146

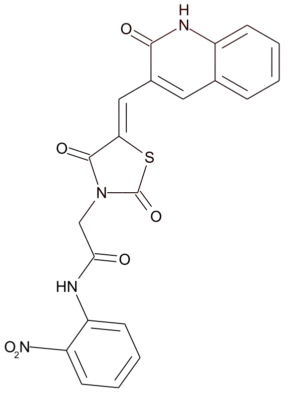

$C_{21}H_{14}N_4O_6S$
Molecular Weight: 450.42405
ALogP: 2.061
Rotatable Bonds: 5
Acceptors: 7
Donors: 2

Structural Similar Compounds

Name	4;4'-DIAMINO-1;1'-DIANTHRIMIDE	ANTHRAQUINONE; 1-AMINO-4-HYDROXY-2-PHENOXY-	2-Anthracenesulfonic acid; 9;10-dihydro-1-amino-4-bromo-9;10-dioxo-; sodium
Structure	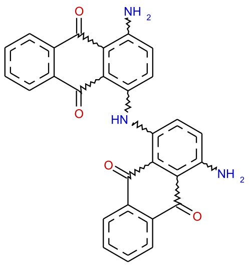	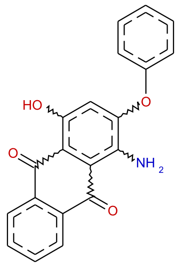	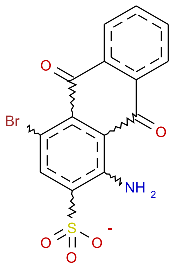
Actual Endpoint	Irritant	Irritant	Irritant
Predicted Endpoint	Irritant	Irritant	Irritant
Distance	0.770	0.803	0.808
Reference	28ZPAK-;125;72	28ZPAK 239;72	Prehled Prumyslove Toxikologie; Organicke Latky; Marhold; J. pp 1062;86

Model Prediction

Prediction: Irritant
Probability: 1
Enrichment: 1.18
Bayesian Score: 1.34
Mahalanobis Distance: 10.8
Mahalanobis Distance p-value: 0.00784

Prediction: Positive if the Bayesian score is above the estimated best cutoff value from minimizing the false positive and false negative rate.
Probability: The esimated probability that the sample is in the positive category. This assumes that the Bayesian score follows a normal distribution and is different from the prediction using a cutoff.
Enrichment: An estimate of enrichment, that is, the increased likelihood (versus random) of this sample being in the category.
Bayesian Score: The standard Laplacian-modified Bayesian score.
Mahalanobis Distance: The Mahalanobis distance (MD) is the distance to the center of the training data. The larger the MD, the less trustworthy the prediction.
Mahalanobis Distance p-value: The p-value gives the fraction of training data with an MD greater than or equal to the one for the given sample, assuming normally distributed data. The smaller the p-value, the less trustworthy the prediciton. For highly non-normal X properties (e.g., fingerprints), the MD p-value is wildly inaccurate.

Model Applicability

Unknown features are fingerprint features in the query molecule, but not found or appearing too infrequently in the training set.

1. All properties and OPS components are within expected ranges.
2. Unknown FCFP_2 feature: -828984032: [*][c](:[*]):[c](:[cH]:[*])[N+](=[*])[*]
3. Unknown FCFP_2 feature: -1338588315: [*]:[c](:[*])[N+](=O)[O-]
4. Unknown FCFP_2 feature: 1872392852: [*][N+](=O)[*]
5. Unknown FCFP_2 feature: 260476081: [*][N+](=[*])[O-]

Feature Contribution

Top features for positive contribution				
**Fingerprint**	**Bit/Smiles**	**Feature Structure**	**Score**	**Irritant in training set**

FCFP_12	1175665944	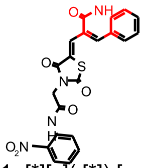<br>[*]C1=[*][c](:[*]):[c<br>](NC1=O):[cH]:[*]	0.198	14 out of 14
FCFP_12	2036120522	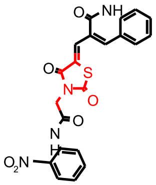<br>[*]CN1C(=O)SC(=[*])C1<br>=[*]	0.167	4 out of 4
FCFP_12	436915834	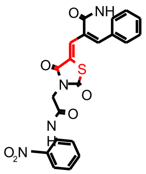<br>[*]\C=C\1/S[*][*]C1=[<br>*]	0.167	4 out of 4
**Top Features for negative contribution**				
**Fingerprint**	**Bit/Smiles**	**Feature Structure**	**Score**	**Irritant in training set**
FCFP_12	451371068	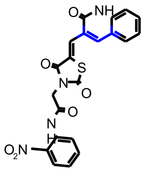<br>[*]C(=C[c](:[*]):[*])<br>[*]	-0.167	6 out of 9
FCFP_12	-1698724694	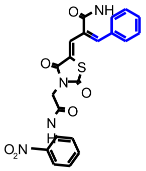<br>[*][c]1:[cH]:[cH]:[cH<br>]:[cH]:[c]:1C=[*]	-0.0964	107 out of 146

FCFP_12	565998553	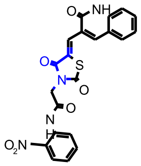<br>[*]N1[*][*]C(=[*])C1=O	-0.0662	198 out of 262

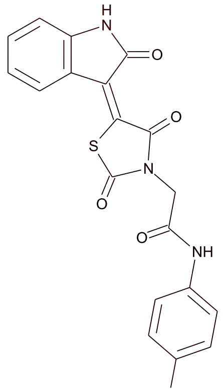

$C_{20}H_{15}N_3O_4S$
Molecular Weight: 393.4158
ALogP: 2.185
Rotatable Bonds: 3
Acceptors: 5
Donors: 2

Model Prediction

Prediction: Irritant
Probability: 1
Enrichment: 1.18
Bayesian Score: 2.09
Mahalanobis Distance: 8.23
Mahalanobis Distance p-value: 0.865

Prediction: Positive if the Bayesian score is above the estimated best cutoff value from minimizing the false positive and false negative rate.
Probability: The esimated probability that the sample is in the positive category. This assumes that the Bayesian score follows a normal distribution and is different from the prediction using a cutoff.
Enrichment: An estimate of enrichment, that is, the increased likelihood (versus random) of this sample being in the category.
Bayesian Score: The standard Laplacian-modified Bayesian score.
Mahalanobis Distance: The Mahalanobis distance (MD) is the distance to the center of the training data. The larger the MD, the less trustworthy the prediction.
Mahalanobis Distance p-value: The p-value gives the fraction of training data with an MD greater than or equal to the one for the given sample, assuming normally distributed data. The smaller the p-value, the less trustworthy the prediciton. For highly non-normal X properties (e.g., fingerprints), the MD p-value is wildly inaccurate.

Structural Similar Compounds

Name	1-AMINO-4-BENZOYLAMINO-ANTHRAQUINONE	ANTHRAQUINONE; 1-AMINO-4-HYDROXY-2-PHENOXY-	5-NORBORNENE-2;3-DICARBOXYLIC ACID; 1;4;5;6;7;7-HEXACHLORO-
Structure	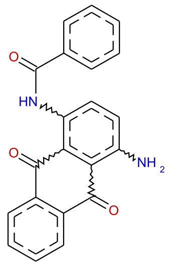	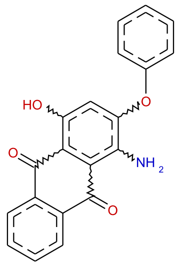	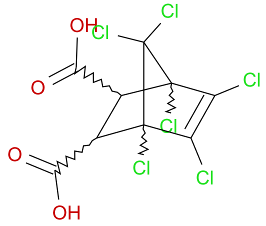
Actual Endpoint	Irritant	Irritant	Irritant
Predicted Endpoint	Irritant	Irritant	Irritant
Distance	0.603	0.620	0.628
Reference	28ZPAK-;124;72	28ZPAK 239;72	28ZPAK-;92;72

Model Applicability

Unknown features are fingerprint features in the query molecule, but not found or appearing too infrequently in the training set.

1. All properties and OPS components are within expected ranges.

Feature Contribution

Top features for positive contribution

Fingerprint	Bit/Smiles	Feature Structure	Score	Irritant in training set
FCFP_12	1175665944	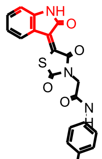 [*]C1=[*][c](:[*]):[c](NC1=O):[cH]:[*]	0.198	14 out of 14

FCFP_12	2036120522	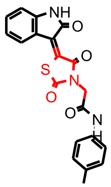 [*]CN1C(=O)SC(=[*])C1=[*]	0.167	4 out of 4
FCFP_12	-568981285	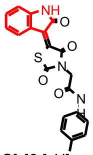 [*]=C1[*]=C[c]2:[cH]:[cH]:[cH]:[cH]:[c]:2N1	0.167	4 out of 4

Top Features for negative contribution

Fingerprint	Bit/Smiles	Feature Structure	Score	Irritant in training set
FCFP_12	-1698724694	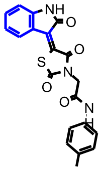 [*][c]1:[cH]:[cH]:[cH]:[cH]:[c]:1C=[*]	-0.0964	107 out of 146
FCFP_12	565998553	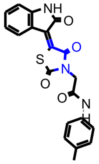 [*]N1[*][*]C(=[*])C1=O	-0.0662	198 out of 262
FCFP_12	-1678275541	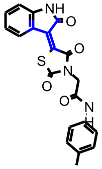 [*]C(=C1C(=[*])[*][*]:[c]1:[*])[*]	-0.0561	3 out of 4

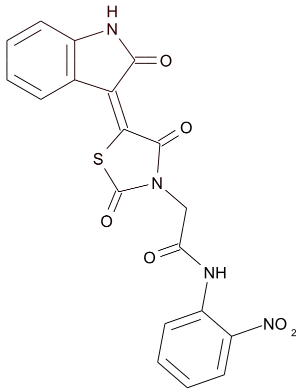

$C_{19}H_{12}N_4O_6S$

Molecular Weight: 424.38678

ALogP: 1.593

Rotatable Bonds: 4

Acceptors: 7

Donors: 2

Structural Similar Compounds

Name	2-Anthracenesulfonic acid; 9;10-dihydro-1-amino-4-bromo-9;10-dioxo-; sodium	2-NAPHTHALENESULFONIC ACID;5-AMINO-6-ETHOXY-	Benzenesulfonic acid; 2-anilino-5-nitro-
Structure	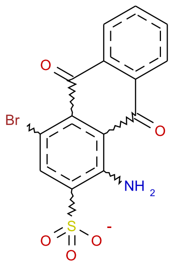	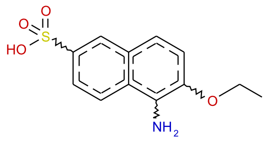	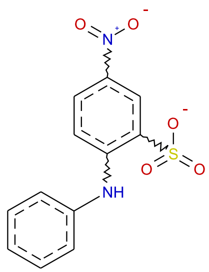
Actual Endpoint	Irritant	Irritant	Irritant
Predicted Endpoint	Irritant	Irritant	Irritant
Distance	0.760	0.784	0.792
Reference	Prehled Prumyslove Toxikologie; Organicke Latky; Marhold; J. pp 1062;86	28ZPAK-;191;72	Prehled Prumyslove Toxikologie; Organicke Latky; Marhold; J. - ;1061;86

Model Prediction

Prediction: Irritant

Probability: 1

Enrichment: 1.18

Bayesian Score: 1.44

Mahalanobis Distance: 8.19

Mahalanobis Distance p-value: 0.878

Prediction: Positive if the Bayesian score is above the estimated best cutoff value from minimizing the false positive and false negative rate.
Probability: The esimated probability that the sample is in the positive category. This assumes that the Bayesian score follows a normal distribution and is different from the prediction using a cutoff.
Enrichment: An estimate of enrichment, that is, the increased likelihood (versus random) of this sample being in the category.
Bayesian Score: The standard Laplacian-modified Bayesian score.
Mahalanobis Distance: The Mahalanobis distance (MD) is the distance to the center of the training data. The larger the MD, the less trustworthy the prediction.
Mahalanobis Distance p-value: The p-value gives the fraction of training data with an MD greater than or equal to the one for the given sample, assuming normally distributed data. The smaller the p-value, the less trustworthy the prediciton. For highly non-normal X properties (e.g., fingerprints), the MD p-value is wildly inaccurate.

Model Applicability

Unknown features are fingerprint features in the query molecule, but not found or appearing too infrequently in the training set.

1. All properties and OPS components are within expected ranges.
2. Unknown FCFP_2 feature: -828984032: [*][c](:[*]):[c](:[cH]:[*])[N+](=[*])[*]
3. Unknown FCFP_2 feature: -1338588315: [*]:[c](:[*])[N+](=O)[O-]
4. Unknown FCFP_2 feature: 1872392852: [*][N+](=O)[*]
5. Unknown FCFP_2 feature: 260476081: [*][N+](=[*])[O-]

Feature Contribution

Top features for positive contribution				
**Fingerprint**	**Bit/Smiles**	**Feature Structure**	**Score**	**Irritant in training set**

FCFP_12	1175665944	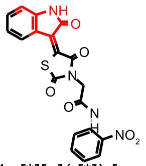 [*]C1=[*][c](:[*]):[c](NC1=O):[cH]:[*]	0.198	14 out of 14
FCFP_12	2036120522	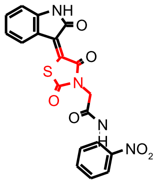 [*]CN1C(=O)SC(=[*])C1 =[*]	0.167	4 out of 4
FCFP_12	-568981285	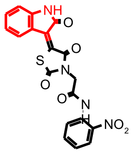 [*]=C1[*]=C[c]2:[cH]: [cH]:[cH]:[cH]:[c]:2 N1	0.167	4 out of 4

Top Features for negative contribution

Fingerprint	Bit/Smiles	Feature Structure	Score	Irritant in training set
FCFP_12	-1698724694	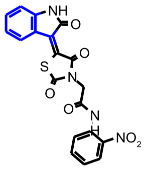 [*][c]1:[cH]:[cH]:[cH]:[cH]:[c]:1C=[*]	-0.0964	107 out of 146
FCFP_12	565998553	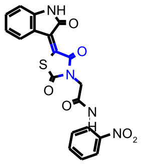 [*]N1[*][*]C(=[*])C1= O	-0.0662	198 out of 262

FCFP_12	8	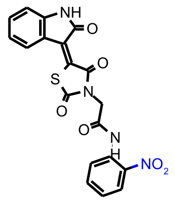<br>[*][N+](=[*])[*]	-0.0561	3 out of 4

Sorafenib

TOPKAT_Ocular_Irritancy_None_vs_Irritant

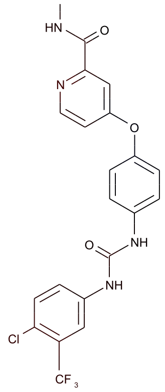

$C_{21}H_{16}ClF_3N_4O_3$
Molecular Weight: 464.82494
ALogP: 4.175
Rotatable Bonds: 6
Acceptors: 4
Donors: 3

Model Prediction

Prediction: Irritant
Probability: 1
Enrichment: 1.18
Bayesian Score: 3.04
Mahalanobis Distance: 6.28
Mahalanobis Distance p-value: 1

Prediction: Positive if the Bayesian score is above the estimated best cutoff value from minimizing the false positive and false negative rate.
Probability: The esimated probability that the sample is in the positive category. This assumes that the Bayesian score follows a normal distribution and is different from the prediction using a cutoff.
Enrichment: An estimate of enrichment, that is, the increased likelihood (versus random) of this sample being in the category.
Bayesian Score: The standard Laplacian-modified Bayesian score.
Mahalanobis Distance: The Mahalanobis distance (MD) is the distance to the center of the training data. The larger the MD, the less trustworthy the prediction.
Mahalanobis Distance p-value: The p-value gives the fraction of training data with an MD greater than or equal to the one for the given sample, assuming normally distributed data. The smaller the p-value, the less trustworthy the prediciton. For highly non-normal X properties (e.g., fingerprints), the MD p-value is wildly inaccurate.

Structural Similar Compounds

Name	BENZANILIDE;2';2'''-DITHIOBIS-	4;4'-DIAMINO-1;1'-DIANTHRIMIDE	5-NORBORNENE-2;3-DICARBOXYLIC ACID; 1;4;5;6;7;7-HEXACHLORO-
Structure	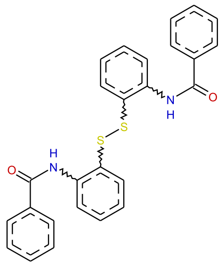	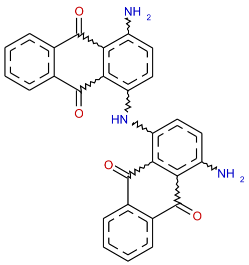	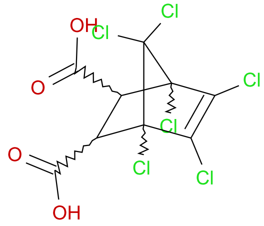
Actual Endpoint	Non-Irritant	Irritant	Irritant
Predicted Endpoint	Non-Irritant	Irritant	Irritant
Distance	0.743	0.791	0.801
Reference	28ZPAK-;173;72	28ZPAK-;125;72	28ZPAK-;92;72

Model Applicability

Unknown features are fingerprint features in the query molecule, but not found or appearing too infrequently in the training set.

1. All properties and OPS components are within expected ranges.

Feature Contribution

Top features for positive contribution				
**Fingerprint**	**Bit/Smiles**	**Feature Structure**	**Score**	**Irritant in training set**
FCFP_12	1747237384	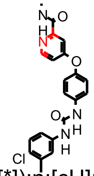 [*][c](:[*]):n:[cH]:[*]	0.208	44 out of 44

FCFP_12	-124655670	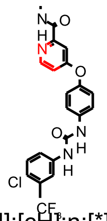 [*]:[cH]:[cH]:n:[*]	0.2	16 out of 16
FCFP_12	-1539132615	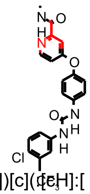 [*]C(=[*])[c](:[cH]:[*]):n:[*]	0.197	13 out of 13

Top Features for negative contribution

Fingerprint	Bit/Smiles	Feature Structure	Score	Irritant in training set
FCFP_12	702861189	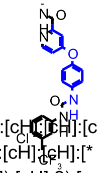 [*]N[c]1:[cH]:[cH]:[c](O[c]2:[cH]:[cH]:[*]:[c]([*]):[cH]:2):[cH]:[cH]:1	-0.268	1 out of 2
FCFP_12	-747629521	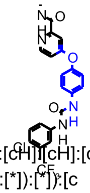 [*]N[c]1:[cH]:[cH]:[c](O[c](:[*]):[*]):[cH]:[cH]:1	-0.268	1 out of 2
FCFP_12	1872154524	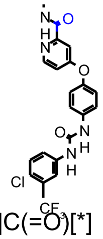 [*]C(=O)[*]	0	563 out of 690

Sunitinib

TOPKAT_Ocular_Irritancy_None_vs_Irritant

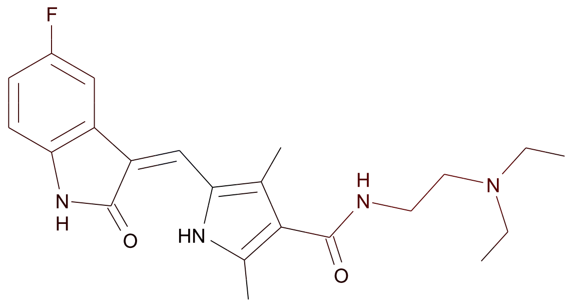

$C_{22}H_{27}FN_4O_2$
Molecular Weight: 398.47378
ALogP: 2.997
Rotatable Bonds: 7
Acceptors: 3
Donors: 3

Model Prediction

Prediction: Irritant
Probability: 1
Enrichment: 1.18
Bayesian Score: 4.17
Mahalanobis Distance: 8.04
Mahalanobis Distance p-value: 0.916

Prediction: Positive if the Bayesian score is above the estimated best cutoff value from minimizing the false positive and false negative rate.
Probability: The esimated probability that the sample is in the positive category. This assumes that the Bayesian score follows a normal distribution and is different from the prediction using a cutoff.
Enrichment: An estimate of enrichment, that is, the increased likelihood (versus random) of this sample being in the category.
Bayesian Score: The standard Laplacian-modified Bayesian score.
Mahalanobis Distance: The Mahalanobis distance (MD) is the distance to the center of the training data. The larger the MD, the less trustworthy the prediction.
Mahalanobis Distance p-value: The p-value gives the fraction of training data with an MD greater than or equal to the one for the given sample, assuming normally distributed data. The smaller the p-value, the less trustworthy the prediciton. For highly non-normal X properties (e.g., fingerprints), the MD p-value is wildly inaccurate.

Structural Similar Compounds

Name	METHANE;TRIS(4-AMINOPHENYL)-	ANTHRAQUINONE; 1-((2-HYDROXYETHYL)AMINO)-4-(METHYLAMINO)-	PHENOL;4-(3-CARBAZOLYLAMINO)-
Structure	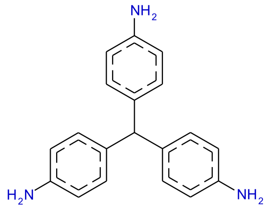	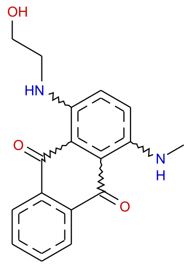	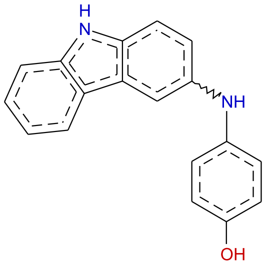
Actual Endpoint	Irritant	Irritant	Irritant
Predicted Endpoint	Irritant	Irritant	Irritant
Distance	0.704	0.757	0.773
Reference	28ZPAK-;73;72	28ZPAK 245;72	28ZPAK-;143;72

Model Applicability

Unknown features are fingerprint features in the query molecule, but not found or appearing too infrequently in the training set.

1. All properties and OPS components are within expected ranges.
2. Unknown FCFP_2 feature: 203707511: [*][c]1:[*]:[*]:[nH]:[c]:1C

Feature Contribution

Top features for positive contribution

Fingerprint	Bit/Smiles	Feature Structure	Score	Irritant in training set
FCFP_12	1175665944	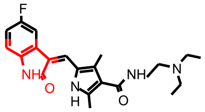 [*]C1=[*][c](:[*]):[c](NC1=O):[cH]:[*]	0.198	14 out of 14

FCFP_12	1851332093	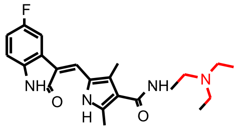 [*]CN(C[*])CC	0.195	12 out of 12
FCFP_12	-885550502	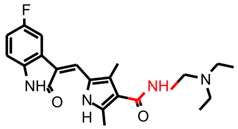 [*]CNC(=[*])[*]	0.18	64 out of 66

Top Features for negative contribution

Fingerprint	Bit/Smiles	Feature Structure	Score	Irritant in training set
FCFP_12	451371068	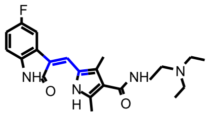 [*]C(=C[c](:[*]):[*]) [*]	-0.167	6 out of 9
FCFP_12	-1678275541	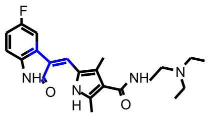 [*]C(=C1C(=[*])[*][*] :[c]1:[*])[*]	-0.0561	3 out of 4
FCFP_12	0	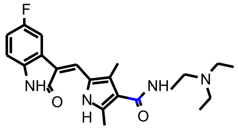 [*]C(=[*])[*]	0	1184 out of 1397

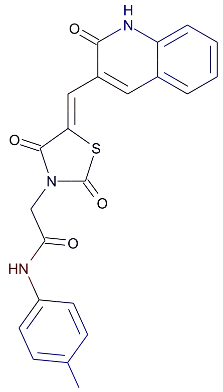

$C_{22}H_{17}N_3O_4S$
Molecular Weight: 419.45307
ALogP: 2.653
Rotatable Bonds: 4
Acceptors: 5
Donors: 2

Model Prediction

Prediction: Non-Carcinogen
Probability: 0.223
Enrichment: 0.693
Bayesian Score: -4.84
Mahalanobis Distance: 9.21
Mahalanobis Distance p-value: 0.739

Prediction: Positive if the Bayesian score is above the estimated best cutoff value from minimizing the false positive and false negative rate.
Probability: The esimated probability that the sample is in the positive category. This assumes that the Bayesian score follows a normal distribution and is different from the prediction using a cutoff.
Enrichment: An estimate of enrichment, that is, the increased likelihood (versus random) of this sample being in the category.
Bayesian Score: The standard Laplacian-modified Bayesian score.
Mahalanobis Distance: The Mahalanobis distance (MD) is the distance to the center of the training data. The larger the MD, the less trustworthy the prediction.
Mahalanobis Distance p-value: The p-value gives the fraction of training data with an MD greater than or equal to the one for the given sample, assuming normally distributed data. The smaller the p-value, the less trustworthy the prediciton. For highly non-normal X properties (e.g., fingerprints), the MD p-value is wildly inaccurate.

Structural Similar Compounds

Name	Bicalutamide	Indapamide	Metolazone
Structure	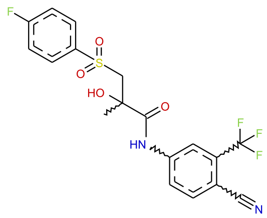	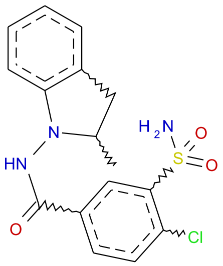	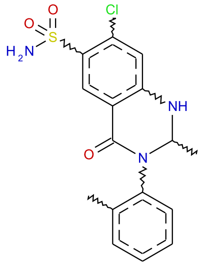
Actual Endpoint	Carcinogen	Non-Carcinogen	Non-Carcinogen
Predicted Endpoint	Carcinogen	Non-Carcinogen	Non-Carcinogen
Distance	0.598	0.613	0.643
Reference	US FDA (Centre for Drug Eval.& Res./Off. Testing & Res.) Sept. 1997	US FDA (Centre for Drug Eval.& Res./Off. Testing & Res.) Sept. 1997	US FDA (Centre for Drug Eval.& Res./Off. Testing & Res.) Sept. 1997

Model Applicability

Unknown features are fingerprint features in the query molecule, but not found or appearing too infrequently in the training set.

1. All properties and OPS components are within expected ranges.
2. Unknown ECFP_2 feature: 2131425032: [*]\C=C(\C=[*])/C(=[*])[*]
3. Unknown ECFP_2 feature: 1182722866: [*]C(=CC(=[*])[*])[*]
4. Unknown ECFP_2 feature: 1000552169: [*]\C=C\1/S[*][*]C1=[*]
5. Unknown ECFP_2 feature: 190445529: [*]N1[*][*]SC1=O

Feature Contribution

Top features for positive contribution

Fingerprint	Bit/Smiles	Feature Structure	Score	Carcinogen in training set
ECFP_12	-177077903	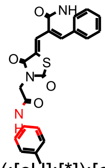 [*]N[c](:[cH]:[*]):[cH]:[*]	0.529	6 out of 10

ECFP_12	-1236483485	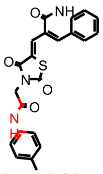 [*]C(=[*])N[c](:[*]):[*]	0.46	9 out of 17
ECFP_12	888054369	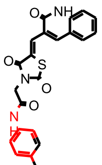 [*]N[c]1:[cH]:[*]:[c]([*]):[cH]:[cH]:1	0.454	5 out of 9

Top Features for negative contribution

Fingerprint	Bit/Smiles	Feature Structure	Score	Carcinogen in training set
ECFP_12	1335833675	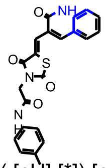 [*]N[c](:[cH]:[*]):[c]([*]):[*]	-1.25	0 out of 8
ECFP_12	-1926229349	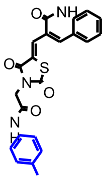 [*][c]1:[cH]:[cH]:[c](C):[cH]:[cH]:1	-1.06	0 out of 6
ECFP_12	-533780882	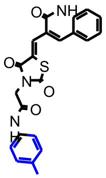 C[c]1:[cH]:[cH]:[*]:[cH]:[cH]:1	-1.06	0 out of 6

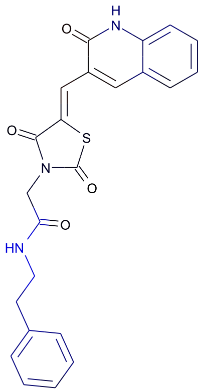

$C_{23}H_{19}N_3O_4S$
Molecular Weight: 433.47965
ALogP: 2.495
Rotatable Bonds: 6
Acceptors: 5
Donors: 2

Model Prediction

Prediction: Non-Carcinogen
Probability: 0.187
Enrichment: 0.582
Bayesian Score: -8.95
Mahalanobis Distance: 11
Mahalanobis Distance p-value: 0.0727

Prediction: Positive if the Bayesian score is above the estimated best cutoff value from minimizing the false positive and false negative rate.
Probability: The esimated probability that the sample is in the positive category. This assumes that the Bayesian score follows a normal distribution and is different from the prediction using a cutoff.
Enrichment: An estimate of enrichment, that is, the increased likelihood (versus random) of this sample being in the category.
Bayesian Score: The standard Laplacian-modified Bayesian score.
Mahalanobis Distance: The Mahalanobis distance (MD) is the distance to the center of the training data. The larger the MD, the less trustworthy the prediction.
Mahalanobis Distance p-value: The p-value gives the fraction of training data with an MD greater than or equal to the one for the given sample, assuming normally distributed data. The smaller the p-value, the less trustworthy the prediciton. For highly non-normal X properties (e.g., fingerprints), the MD p-value is wildly inaccurate.

Structural Similar Compounds

Name	Bicalutamide	Glipizide	Moricizine
Structure	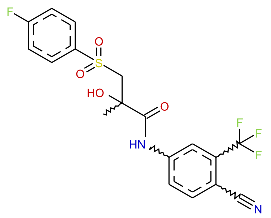	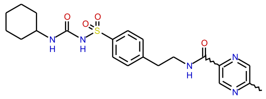	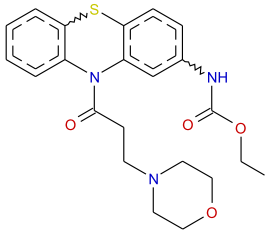
Actual Endpoint	Carcinogen	Non-Carcinogen	Carcinogen
Predicted Endpoint	Carcinogen	Non-Carcinogen	Carcinogen
Distance	0.523	0.652	0.666
Reference	US FDA (Centre for Drug Eval.& Res./Off. Testing & Res.) Sept. 1997	US FDA (Centre for Drug Eval.& Res./Off. Testing & Res.) Sept. 1997	US FDA (Centre for Drug Eval.& Res./Off. Testing & Res.) Sept. 1997

Model Applicability

Unknown features are fingerprint features in the query molecule, but not found or appearing too infrequently in the training set.

1. All properties and OPS components are within expected ranges.
2. Unknown ECFP_2 feature: 2131425032: [*]\C=C(\C=[*])/C(=[*])[*]
3. Unknown ECFP_2 feature: 1182722866: [*]C(=CC(=[*])[*])[*]
4. Unknown ECFP_2 feature: 1000552169: [*]\C=C\1/S[*][*]C1=[*]
5. Unknown ECFP_2 feature: 190445529: [*]N1[*][*]SC1=O

Feature Contribution

Top features for positive contribution				
**Fingerprint**	**Bit/Smiles**	**Feature Structure**	**Score**	**Carcinogen in training set**
ECFP_12	-1925046727	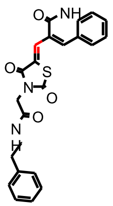 [*]C=[*]	0.407	16 out of 33

ECFP_12	1336666212	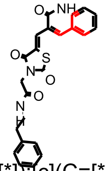 [*][c](:[*]):[c](C=[*]):[cH]:[*]	0.288	2 out of 4
ECFP_12	-1650219925	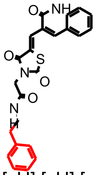 [*]C[c]1:[cH]:[cH]:[c H]:[cH]:[cH]:1	0.208	6 out of 15

Top Features for negative contribution

**Fingerprint**	**Bit/Smiles**	**Feature Structure**	**Score**	**Carcinogen in training set**
ECFP_12	1335833675	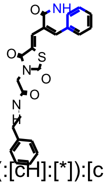 [*]N[c](:[cH]:[*]):[c]([*]):[*]	-1.25	0 out of 8
ECFP_12	497523368	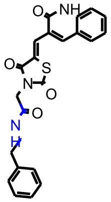 [*]CNC(=[*])[*]	-0.989	1 out of 14
ECFP_12	-2058216030	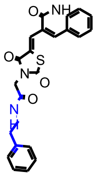 [*]C(=[*])NCC[c](:[*]):[*]	-0.811	0 out of 4

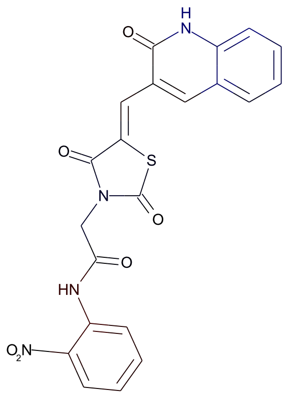

$C_{21}H_{14}N_4O_6S$

Molecular Weight: 450.42405

ALogP: 2.061

Rotatable Bonds: 5

Acceptors: 7

Donors: 2

Model Prediction

Prediction: Non-Carcinogen

Probability: 0.257

Enrichment: 0.798

Bayesian Score: -2.32

Mahalanobis Distance: 9.58

Mahalanobis Distance p-value: 0.568

Prediction: Positive if the Bayesian score is above the estimated best cutoff value from minimizing the false positive and false negative rate.
Probability: The esimated probability that the sample is in the positive category. This assumes that the Bayesian score follows a normal distribution and is different from the prediction using a cutoff.
Enrichment: An estimate of enrichment, that is, the increased likelihood (versus random) of this sample being in the category.
Bayesian Score: The standard Laplacian-modified Bayesian score.
Mahalanobis Distance: The Mahalanobis distance (MD) is the distance to the center of the training data. The larger the MD, the less trustworthy the prediction.
Mahalanobis Distance p-value: The p-value gives the fraction of training data with an MD greater than or equal to the one for the given sample, assuming normally distributed data. The smaller the p-value, the less trustworthy the prediciton. For highly non-normal X properties (e.g., fingerprints), the MD p-value is wildly inaccurate.

Structural Similar Compounds

Name	Polythiazide	Nedocromil	Penicillin
Structure	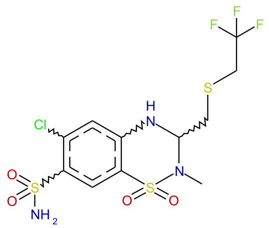	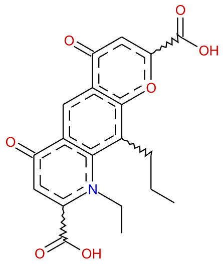	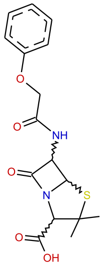
Actual Endpoint	Non-Carcinogen	Non-Carcinogen	Non-Carcinogen
Predicted Endpoint	Non-Carcinogen	Non-Carcinogen	Non-Carcinogen
Distance	0.577	0.665	0.696
Reference	US FDA (Centre for Drug Eval.& Res./Off. Testing & Res.) Sept. 1997	US FDA (Centre for Drug Eval.& Res./Off. Testing & Res.) Sept. 1997	US FDA (Centre for Drug Eval.& Res./Off. Testing & Res.) Sept. 1997

Model Applicability

Unknown features are fingerprint features in the query molecule, but not found or appearing too infrequently in the training set.

1. All properties and OPS components are within expected ranges.
2. Unknown ECFP_2 feature: 1043790491: [*][N+](=[*])[*]
3. Unknown ECFP_2 feature: 781519895: [*][O-]
4. Unknown ECFP_2 feature: 2131425032: [*]\C=C(\C=[*])/C(=[*])[*]
5. Unknown ECFP_2 feature: 1182722866: [*]C(=CC(=[*])[*])[*]
6. Unknown ECFP_2 feature: 1000552169: [*]\C=C\1/S[*][*]C1=[*]
7. Unknown ECFP_2 feature: 190445529: [*]N1[*][*]SC1=O
8. Unknown ECFP_2 feature: -1956535100: [*][c](:[*]):[c](:[cH]:[*])[N+](=[*])[*]
9. Unknown ECFP_2 feature: -215026467: [*]:[c](:[*])[N+](=O)[O-]
10. Unknown ECFP_2 feature: 2104376220: [*][N+](=O)[*]
11. Unknown ECFP_2 feature: -659271057: [*][N+](=[*])[O-]

Feature Contribution

Top features for positive contribution				
**Fingerprint**	**Bit/Smiles**	**Feature Structure**	**Score**	**Carcinogen in training set**

ECFP_12	-1236483485	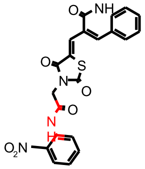 [*]C(=[*])N[c](:[*]):[*]	0.46	9 out of 17
ECFP_12	-1925046727	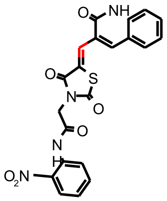 [*]C=[*]	0.407	16 out of 33
ECFP_12	1799533624	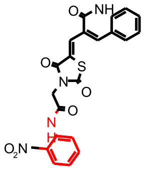 [*]N[c]1:[cH]:[cH]:[cH]:[cH]:[c]:1[*]	0.288	2 out of 4

Top Features for negative contribution

Fingerprint	Bit/Smiles	Feature Structure	Score	Carcinogen in training set
ECFP_12	1335833675	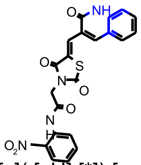 [*]N[c](:[cH]:[*]):[c]([*]):[*]	-1.25	0 out of 8
ECFP_12	1640720160	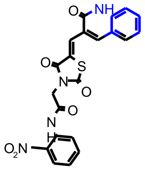 [*]N[c]1:[cH]:[cH]:[cH]:[*]:[c]:1[*]	-0.485	0 out of 2

ECFP_12	912478223	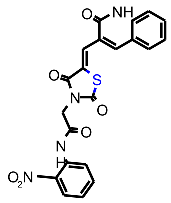 [*]S[*]	-0.318	2 out of 10

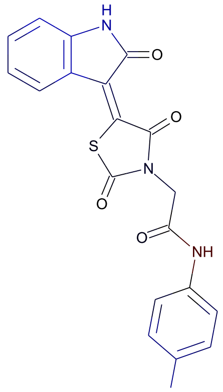

$C_{20}H_{15}N_3O_4S$
Molecular Weight: 393.4158
ALogP: 2.185
Rotatable Bonds: 3
Acceptors: 5
Donors: 2

Model Prediction

Prediction: Non-Carcinogen
Probability: 0.2
Enrichment: 0.621
Bayesian Score: -7.21
Mahalanobis Distance: 7.91
Mahalanobis Distance p-value: 0.991

Prediction: Positive if the Bayesian score is above the estimated best cutoff value from minimizing the false positive and false negative rate.
Probability: The esimated probability that the sample is in the positive category. This assumes that the Bayesian score follows a normal distribution and is different from the prediction using a cutoff.
Enrichment: An estimate of enrichment, that is, the increased likelihood (versus random) of this sample being in the category.
Bayesian Score: The standard Laplacian-modified Bayesian score.
Mahalanobis Distance: The Mahalanobis distance (MD) is the distance to the center of the training data. The larger the MD, the less trustworthy the prediction.
Mahalanobis Distance p-value: The p-value gives the fraction of training data with an MD greater than or equal to the one for the given sample, assuming normally distributed data. The smaller the p-value, the less trustworthy the prediciton. For highly non-normal X properties (e.g., fingerprints), the MD p-value is wildly inaccurate.

Structural Similar Compounds

Name	Indapamide	Metolazone	Acetohexamide
Structure	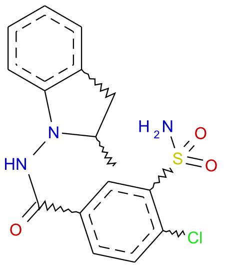	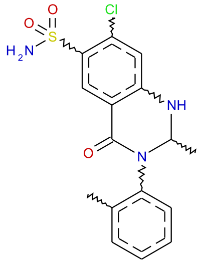	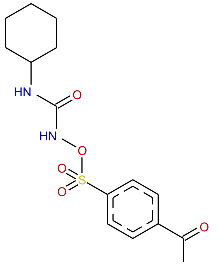
Actual Endpoint	Non-Carcinogen	Non-Carcinogen	Non-Carcinogen
Predicted Endpoint	Non-Carcinogen	Non-Carcinogen	Non-Carcinogen
Distance	0.583	0.585	0.630
Reference	US FDA (Centre for Drug Eval.& Res./Off. Testing & Res.) Sept. 1997	US FDA (Centre for Drug Eval.& Res./Off. Testing & Res.) Sept. 1997	US FDA (Centre for Drug Eval.& Res./Off. Testing & Res.) Sept. 1997

Model Applicability

Unknown features are fingerprint features in the query molecule, but not found or appearing too infrequently in the training set.

1. All properties and OPS components are within expected ranges.
2. Unknown ECFP_2 feature: -631778390: [*]C(=C1S[*][*]C1=[*])[*]
3. Unknown ECFP_2 feature: 190445529: [*]N1[*][*]SC1=O

Feature Contribution

Top features for positive contribution				
Fingerprint	Bit/Smiles	Feature Structure	Score	Carcinogen in training set
ECFP_12	-177077903	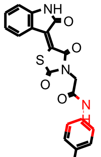 [*]N[c](:[cH]:[*]):[cH]:[*]	0.529	6 out of 10

ECFP_12	-1236483485	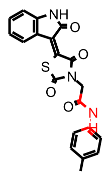 [*]C(=[*])N[c](:[*]):[*]	0.46	9 out of 17
ECFP_12	888054369	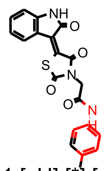 [*]N[c]1:[cH]:[*]:[c]([*]):[cH]:[cH]:1	0.454	5 out of 9

Top Features for negative contribution

Fingerprint	Bit/Smiles	Feature Structure	Score	Carcinogen in training set
ECFP_12	1335833675	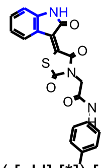 [*]N[c](:[cH]:[*]):[c]([*]):[*]	-1.25	0 out of 8
ECFP_12	-533780882	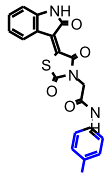 C[c]1:[cH]:[cH]:[*]:[cH]:[cH]:1	-1.06	0 out of 6
ECFP_12	-1926229349	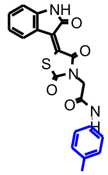 [*][c]1:[cH]:[cH]:[c](C):[cH]:[cH]:1	-1.06	0 out of 6

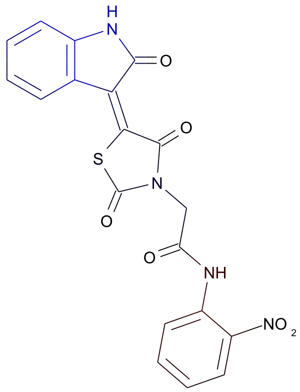

$C_{19}H_{12}N_4O_6S$
Molecular Weight: 424.38678
ALogP: 1.593
Rotatable Bonds: 4
Acceptors: 7
Donors: 2

Structural Similar Compounds

Name	Polythiazide	Penicillin	Nedocromil
Structure	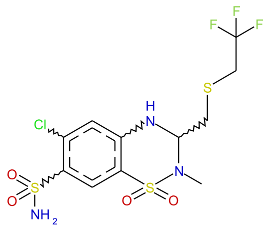	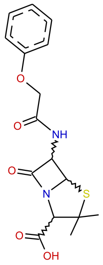	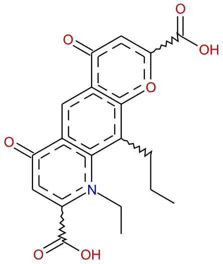
Actual Endpoint	Non-Carcinogen	Non-Carcinogen	Non-Carcinogen
Predicted Endpoint	Non-Carcinogen	Non-Carcinogen	Non-Carcinogen
Distance	0.600	0.668	0.683
Reference	US FDA (Centre for Drug Eval.& Res./Off. Testing & Res.) Sept. 1997	US FDA (Centre for Drug Eval.& Res./Off. Testing & Res.) Sept. 1997	US FDA (Centre for Drug Eval.& Res./Off. Testing & Res.) Sept. 1997

Model Prediction

Prediction: Non-Carcinogen
Probability: 0.229
Enrichment: 0.711
Bayesian Score: -4.33
Mahalanobis Distance: 8.36
Mahalanobis Distance p-value: 0.961

Prediction: Positive if the Bayesian score is above the estimated best cutoff value from minimizing the false positive and false negative rate.
Probability: The esimated probability that the sample is in the positive category. This assumes that the Bayesian score follows a normal distribution and is different from the prediction using a cutoff.
Enrichment: An estimate of enrichment, that is, the increased likelihood (versus random) of this sample being in the category.
Bayesian Score: The standard Laplacian-modified Bayesian score.
Mahalanobis Distance: The Mahalanobis distance (MD) is the distance to the center of the training data. The larger the MD, the less trustworthy the prediction.
Mahalanobis Distance p-value: The p-value gives the fraction of training data with an MD greater than or equal to the one for the given sample, assuming normally distributed data. The smaller the p-value, the less trustworthy the prediciton. For highly non-normal X properties (e.g., fingerprints), the MD p-value is wildly inaccurate.

Model Applicability

Unknown features are fingerprint features in the query molecule, but not found or appearing too infrequently in the training set.

1. All properties and OPS components are within expected ranges.
2. Unknown ECFP_2 feature: 1043790491: [*][N+](=[*])[*]
3. Unknown ECFP_2 feature: 781519895: [*][O-]
4. Unknown ECFP_2 feature: -631778390: [*]C(=C1S[*][*]C1=[*])[*]
5. Unknown ECFP_2 feature: 190445529: [*]N1[*][*]SC1=O
6. Unknown ECFP_2 feature: -1956535100: [*][c](:[*]):[c](:[cH]:[*])[N+](=[*])[*]
7. Unknown ECFP_2 feature: -215026467: [*]:[c](:[*])[N+](=O)[O-]
8. Unknown ECFP_2 feature: 2104376220: [*][N+](=O)[*]
9. Unknown ECFP_2 feature: -659271057: [*][N+](=[*])[O-]

Feature Contribution

Top features for positive contribution				
**Fingerprint**	**Bit/Smiles**	**Feature Structure**	**Score**	**Carcinogen in training set**

ECFP_12	-1236483485	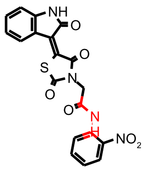 [*]C(=[*])N[c](:[*]):[*]	0.46	9 out of 17
ECFP_12	1635992319	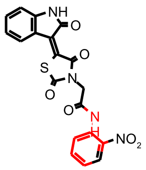 [*]N[c]1:[cH]:[cH]:[cH]:[*]:[c]:1[*]	0.288	2 out of 4
ECFP_12	1799533624	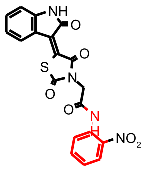 [*]N[c]1:[cH]:[cH]:[cH]:[cH]:[c]:1[*]	0.288	2 out of 4

Top Features for negative contribution

Fingerprint	Bit/Smiles	Feature Structure	Score	Carcinogen in training set
ECFP_12	1335833675	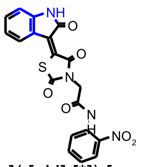 [*]N[c](:[cH]:[*]):[c]([*]):[*]	-1.25	0 out of 8
ECFP_12	-319922023	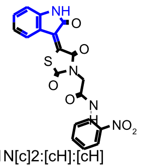 [*]=C1N[c]2:[cH]:[cH]:[*]:[cH]:[c]:2C1=[*]	-0.661	0 out of 3

ECFP_12	1640720160	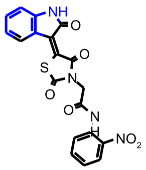 [*]N[c]1:[cH]:[cH]:[cH]:[*]:[c]:1[*]	-0.485	0 out of 2

Sorafenib

TOPKAT_Rat_Female_FDA_None_vs_Carcinogen

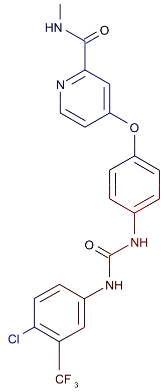

$C_{21}H_{16}ClF_3N_4O_3$
Molecular Weight: 464.82494
ALogP: 4.175
Rotatable Bonds: 6
Acceptors: 4
Donors: 3

Structural Similar Compounds

Name	Glimepride	Glyburide	Fluvastatin
Structure	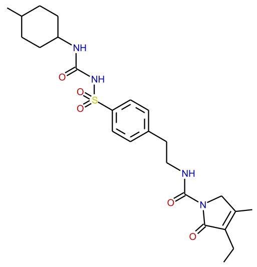	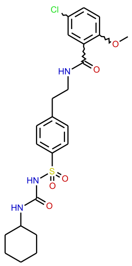	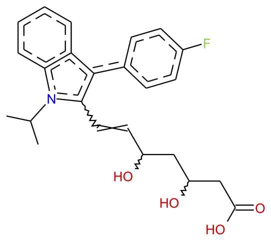
Actual Endpoint	Non-Carcinogen	Non-Carcinogen	Non-Carcinogen
Predicted Endpoint	Non-Carcinogen	Non-Carcinogen	Non-Carcinogen
Distance	0.620	0.635	0.635
Reference	US FDA (Centre for Drug Eval.& Res./Off. Testing & Res.) Sept. 1997	US FDA (Centre for Drug Eval.& Res./Off. Testing & Res.) Sept. 1997	US FDA (Centre for Drug Eval.& Res./Off. Testing & Res.) Sept. 1997

Model Prediction

Prediction: Non-Carcinogen
Probability: 0.236
Enrichment: 0.734
Bayesian Score: -3.76
Mahalanobis Distance: 12.2
Mahalanobis Distance p-value: 0.00229

Prediction: Positive if the Bayesian score is above the estimated best cutoff value from minimizing the false positive and false negative rate.
Probability: The esimated probability that the sample is in the positive category. This assumes that the Bayesian score follows a normal distribution and is different from the prediction using a cutoff.
Enrichment: An estimate of enrichment, that is, the increased likelihood (versus random) of this sample being in the category.
Bayesian Score: The standard Laplacian-modified Bayesian score.
Mahalanobis Distance: The Mahalanobis distance (MD) is the distance to the center of the training data. The larger the MD, the less trustworthy the prediction.
Mahalanobis Distance p-value: The p-value gives the fraction of training data with an MD greater than or equal to the one for the given sample, assuming normally distributed data. The smaller the p-value, the less trustworthy the prediciton. For highly non-normal X properties (e.g., fingerprints), the MD p-value is wildly inaccurate.

Model Applicability

Unknown features are fingerprint features in the query molecule, but not found or appearing too infrequently in the training set.

1. All properties and OPS components are within expected ranges.

Feature Contribution

Top features for positive contribution

Fingerprint	Bit/Smiles	Feature Structure	Score	Carcinogen in training set
ECFP_12	-970385855	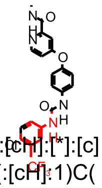 [*]N[c]1:[cH]:[*]:[c]([*]):[c](:[cH]:1)C([*])([*])[*]	0.613	2 out of 2

ECFP_12	-177077903	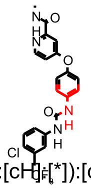 [*]N[c](:[cH]:[*]):[cH]:[*]	0.529	6 out of 10
ECFP_12	-1236483485	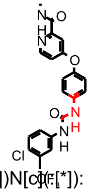 [*]C(=[*])N[c](:[*]):[*]	0.46	9 out of 17
**Top Features for negative contribution**				
**Fingerprint**	**Bit/Smiles**	**Feature Structure**	**Score**	**Carcinogen in training set**
ECFP_12	1335691903	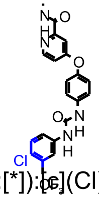 [*][c](:[*]):[c](Cl):[cH]:[*]	-1.11	2 out of 26
ECFP_12	99947387	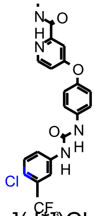 [*]:[c](:[*])Cl	-0.817	8 out of 62
ECFP_12	1413420509	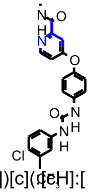 [*]C(=[*])[c](:[cH]:[*]):n:[*]	-0.661	0 out of 3

Sunitinib

TOPKAT_Rat_Female_FDA_None_vs_Carcinogen

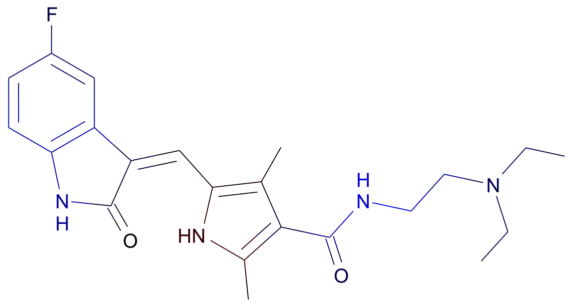

$C_{22}H_{27}FN_4O_2$
Molecular Weight: 398.47378
ALogP: 2.997
Rotatable Bonds: 7
Acceptors: 3
Donors: 3

Structural Similar Compounds

Name	Fluvastatin	Metoclopramide	Torsemide
Structure	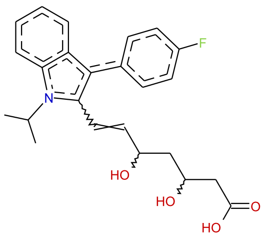	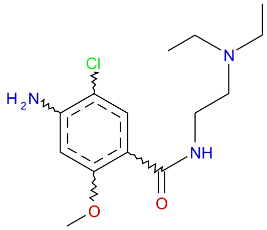	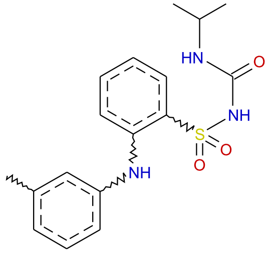
Actual Endpoint	Non-Carcinogen	Non-Carcinogen	Carcinogen
Predicted Endpoint	Non-Carcinogen	Non-Carcinogen	Carcinogen
Distance	0.629	0.681	0.684
Reference	US FDA (Centre for Drug Eval.& Res./Off. Testing & Res.) Sept. 1997	US FDA (Centre for Drug Eval.& Res./Off. Testing & Res.) Sept. 1997	US FDA (Centre for Drug Eval.& Res./Off. Testing & Res.) Sept. 1997

Model Prediction

Prediction: Non-Carcinogen
Probability: 0.188
Enrichment: 0.584
Bayesian Score: -8.84
Mahalanobis Distance: 11.3
Mahalanobis Distance p-value: 0.0371

Prediction: Positive if the Bayesian score is above the estimated best cutoff value from minimizing the false positive and false negative rate.
Probability: The esimated probability that the sample is in the positive category. This assumes that the Bayesian score follows a normal distribution and is different from the prediction using a cutoff.
Enrichment: An estimate of enrichment, that is, the increased likelihood (versus random) of this sample being in the category.
Bayesian Score: The standard Laplacian-modified Bayesian score.
Mahalanobis Distance: The Mahalanobis distance (MD) is the distance to the center of the training data. The larger the MD, the less trustworthy the prediction.
Mahalanobis Distance p-value: The p-value gives the fraction of training data with an MD greater than or equal to the one for the given sample, assuming normally distributed data. The smaller the p-value, the less trustworthy the prediciton. For highly non-normal X properties (e.g., fingerprints), the MD p-value is wildly inaccurate.

Model Applicability

Unknown features are fingerprint features in the query molecule, but not found or appearing too infrequently in the training set.

1. All properties and OPS components are within expected ranges.
2. Unknown ECFP_2 feature: 1791989338: [*][c]1:[*]:[*]:[nH]:[c]:1C
3. Unknown ECFP_2 feature: 980271847: [*][c]1:[*]:[*]:[nH]:[c]:1C=[*]
4. Unknown ECFP_2 feature: 1182722866: [*]C(=CC(=[*])[*])[*]

Feature Contribution

Top features for positive contribution				
**Fingerprint**	**Bit/Smiles**	**Feature Structure**	**Score**	**Carcinogen in training set**
ECFP_12	558201926	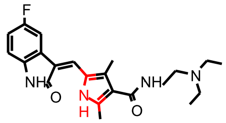 [*][c]1:[*]:[*]:[c]([*]):[nH]:1	0.539	5 out of 8

ECFP_12	-1658273810	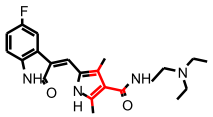<br>[*]C(=[*])[c]1:[c]([*<br>]):[*]:[*]:[c]:1[*]	0.421	1 out of 1
ECFP_12	-152683720	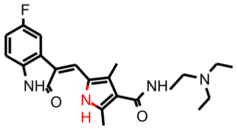<br>[*]:[nH]:[*]	0.412	9 out of 18

Top Features for negative contribution

Fingerprint	Bit/Smiles	Feature Structure	Score	Carcinogen in training set
ECFP_12	1335833675	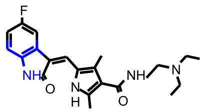<br>[*]N[c](:[cH]:[*]):[c<br>]([*]):[*]	-1.25	0 out of 8
ECFP_12	497523368	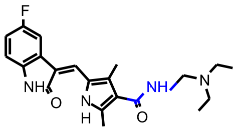<br>[*]CNC(=[*])[*]	-0.989	1 out of 14
ECFP_12	2083628577	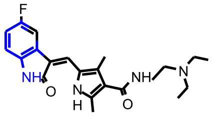<br>[*][c]1:[*]:[c]2[*][*<br>]N[c]:2:[cH]:[cH]:1	-0.811	0 out of 4

1 TOPKAT_Rat_Male_FDA_None_vs_Carcinogen

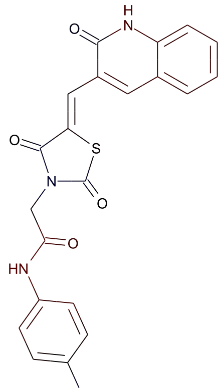

$C_{22}H_{17}N_3O_4S$
Molecular Weight: 419.45307
ALogP: 2.653
Rotatable Bonds: 4
Acceptors: 5
Donors: 2

Model Prediction

Prediction: Carcinogen
Probability: 0.415
Enrichment: 1.24
Bayesian Score: 1.91
Mahalanobis Distance: 11
Mahalanobis Distance p-value: 0.205

Prediction: Positive if the Bayesian score is above the estimated best cutoff value from minimizing the false positive and false negative rate.
Probability: The esimated probability that the sample is in the positive category. This assumes that the Bayesian score follows a normal distribution and is different from the prediction using a cutoff.
Enrichment: An estimate of enrichment, that is, the increased likelihood (versus random) of this sample being in the category.
Bayesian Score: The standard Laplacian-modified Bayesian score.
Mahalanobis Distance: The Mahalanobis distance (MD) is the distance to the center of the training data. The larger the MD, the less trustworthy the prediction.
Mahalanobis Distance p-value: The p-value gives the fraction of training data with an MD greater than or equal to the one for the given sample, assuming normally distributed data. The smaller the p-value, the less trustworthy the prediciton. For highly non-normal X properties (e.g., fingerprints), the MD p-value is wildly inaccurate.

Structural Similar Compounds

Name	Bicalutamide	Indapamide	Metolazone
Structure	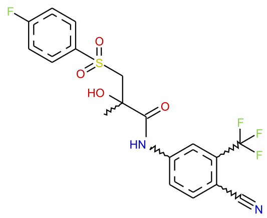	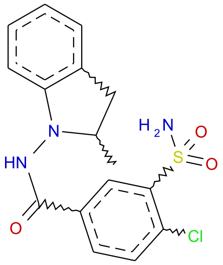	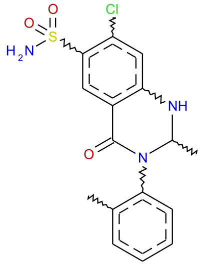
Actual Endpoint	Carcinogen	Non-Carcinogen	Non-Carcinogen
Predicted Endpoint	Carcinogen	Non-Carcinogen	Non-Carcinogen
Distance	0.567	0.590	0.610
Reference	US FDA (Centre for Drug Eval.& Res./Off. Testing & Res.) Sept. 1997	US FDA (Centre for Drug Eval.& Res./Off. Testing & Res.) Sept. 1997	US FDA (Centre for Drug Eval.& Res./Off. Testing & Res.) Sept. 1997

Model Applicability

Unknown features are fingerprint features in the query molecule, but not found or appearing too infrequently in the training set.

1. All properties and OPS components are within expected ranges.

Feature Contribution

Top features for positive contribution

Fingerprint	Bit/Smiles	Feature Structure	Score	Carcinogen in training set
SCFP_6	-347048986	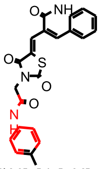 [*]C(=[*])N[c]1:[cH]:[cH]:[*]:[cH]:[cH]:1	0.615	5 out of 7

SCFP_6	814408713	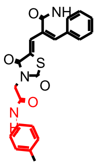 [*]CC(=O)N[c]1:[cH]:[cH]:[c]([*]):[cH]:[cH]:1	0.603	2 out of 2
SCFP_6	2097618059	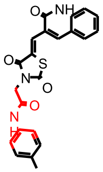 [*]CC(=O)N[c](:[cH]:[*]):[cH]:[*]	0.437	7 out of 13

Top Features for negative contribution

Fingerprint	Bit/Smiles	Feature Structure	Score	Carcinogen in training set
SCFP_6	399659969	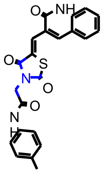 [*]CN1C(=[*])[*][*]C1=[*]	-0.578	1 out of 8
SCFP_6	2102703671	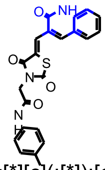 [*]C1=[*][c](:[*]):[c](NC1=O):[cH]:[*]	-0.496	0 out of 2
SCFP_6	795925860	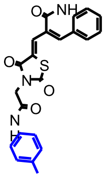 [*][c]1:[cH]:[cH]:[c](C):[cH]:[cH]:1	-0.38	1 out of 6

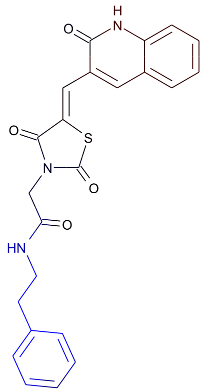

$C_{23}H_{19}N_3O_4S$
Molecular Weight: 433.47965
ALogP: 2.495
Rotatable Bonds: 6
Acceptors: 5
Donors: 2

Model Prediction

Prediction: Non-Carcinogen
Probability: 0.219
Enrichment: 0.654
Bayesian Score: -6.13
Mahalanobis Distance: 14.1
Mahalanobis Distance p-value: 3.13e-005

Prediction: Positive if the Bayesian score is above the estimated best cutoff value from minimizing the false positive and false negative rate.
Probability: The esimated probability that the sample is in the positive category. This assumes that the Bayesian score follows a normal distribution and is different from the prediction using a cutoff.
Enrichment: An estimate of enrichment, that is, the increased likelihood (versus random) of this sample being in the category.
Bayesian Score: The standard Laplacian-modified Bayesian score.
Mahalanobis Distance: The Mahalanobis distance (MD) is the distance to the center of the training data. The larger the MD, the less trustworthy the prediction.
Mahalanobis Distance p-value: The p-value gives the fraction of training data with an MD greater than or equal to the one for the given sample, assuming normally distributed data. The smaller the p-value, the less trustworthy the prediciton. For highly non-normal X properties (e.g., fingerprints), the MD p-value is wildly inaccurate.

Structural Similar Compounds

Name	Bicalutamide	Glipizide	Moricizine
Structure	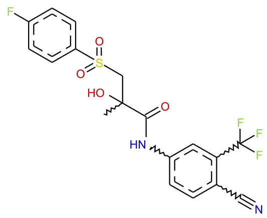	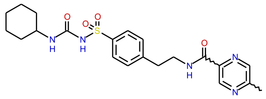	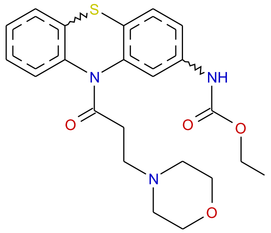
Actual Endpoint	Carcinogen	Non-Carcinogen	Carcinogen
Predicted Endpoint	Carcinogen	Non-Carcinogen	Carcinogen
Distance	0.493	0.617	0.637
Reference	US FDA (Centre for Drug Eval.& Res./Off. Testing & Res.) Sept. 1997	US FDA (Centre for Drug Eval.& Res./Off. Testing & Res.) Sept. 1997	US FDA (Centre for Drug Eval.& Res./Off. Testing & Res.) Sept. 1997

Model Applicability

Unknown features are fingerprint features in the query molecule, but not found or appearing too infrequently in the training set.

1. OPS PC29 out of range. Value: -4.0299. Training min, max, SD, explained variance: -3.1746, 3.7825, 1.007, 0.0095.

Feature Contribution

Top features for positive contribution				
**Fingerprint**	**Bit/Smiles**	**Feature Structure**	**Score**	**Carcinogen in training set**
SCFP_6	-1971137145	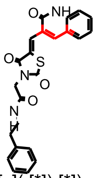 [*]C(=C[c](:[*]):[*])[*]	0.434	5 out of 9

SCFP_6	853077039	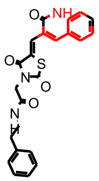<br>[*]C1=C[c]2:[cH]:[cH]:[*]:[cH]:[c]:2N[*]1	0.415	1 out of 1
SCFP_6	1730407098	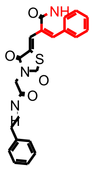<br>[*]C1=C[c]2:[cH]:[cH]:[cH]:[cH]:[c]:2N[*]1	0.415	1 out of 1

Top Features for negative contribution

**Fingerprint**	**Bit/Smiles**	**Feature Structure**	**Score**	**Carcinogen in training set**
SCFP_6	-1211866396	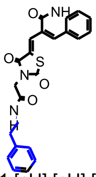<br>[*]CC[c]1:[cH]:[cH]:[*]:[cH]:[cH]:1	-1.1	2 out of 25
SCFP_6	-1849236245	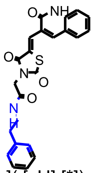<br>[*]NCC[c](:[cH]:[*]):[cH]:[*]	-0.825	0 out of 4
SCFP_6	-1640858361	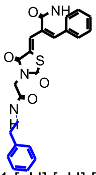<br>[*]CC[c]1:[cH]:[cH]:[cH]:[cH]:[cH]:1	-0.817	1 out of 11

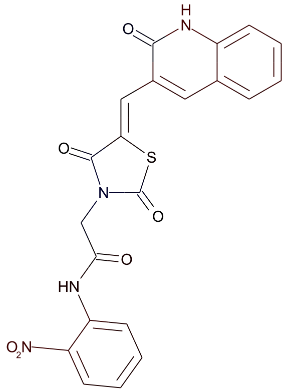

$C_{21}H_{14}N_4O_6S$
Molecular Weight: 450.42405
ALogP: 2.061
Rotatable Bonds: 5
Acceptors: 7
Donors: 2

Model Prediction

Prediction: Carcinogen
Probability: 0.461
Enrichment: 1.38
Bayesian Score: 3.28
Mahalanobis Distance: 16.3
Mahalanobis Distance p-value: 9.61e-010

Prediction: Positive if the Bayesian score is above the estimated best cutoff value from minimizing the false positive and false negative rate.
Probability: The esimated probability that the sample is in the positive category. This assumes that the Bayesian score follows a normal distribution and is different from the prediction using a cutoff.
Enrichment: An estimate of enrichment, that is, the increased likelihood (versus random) of this sample being in the category.
Bayesian Score: The standard Laplacian-modified Bayesian score.
Mahalanobis Distance: The Mahalanobis distance (MD) is the distance to the center of the training data. The larger the MD, the less trustworthy the prediction.
Mahalanobis Distance p-value: The p-value gives the fraction of training data with an MD greater than or equal to the one for the given sample, assuming normally distributed data. The smaller the p-value, the less trustworthy the prediciton. For highly non-normal X properties (e.g., fingerprints), the MD p-value is wildly inaccurate.

Structural Similar Compounds

Name	Polythiazide	Nedocromil	Penicillin
Structure	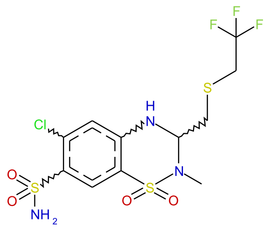	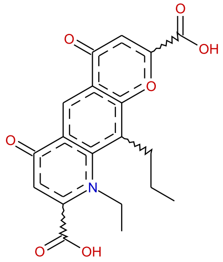	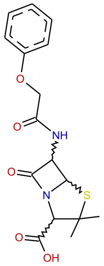
Actual Endpoint	Non-Carcinogen	Non-Carcinogen	Non-Carcinogen
Predicted Endpoint	Non-Carcinogen	Non-Carcinogen	Non-Carcinogen
Distance	0.559	0.649	0.690
Reference	US FDA (Centre for Drug Eval.& Res./Off. Testing & Res.) Sept. 1997	US FDA (Centre for Drug Eval.& Res./Off. Testing & Res.) Sept. 1997	US FDA (Centre for Drug Eval.& Res./Off. Testing & Res.) Sept. 1997

Model Applicability

Unknown features are fingerprint features in the query molecule, but not found or appearing too infrequently in the training set.

1. OPS PC12 out of range. Value: -4.4575. Training min, max, SD, explained variance: -3.9196, 6.4101, 1.581, 0.0233.

Feature Contribution

Top features for positive contribution

Fingerprint	Bit/Smiles	Feature Structure	Score	Carcinogen in training set
SCFP_6	2097618059	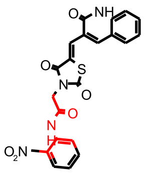 [*]CC(=O)N[c](:[cH]:[*]):[cH]:[*]	0.437	7 out of 13

SCFP_6	-1971137145	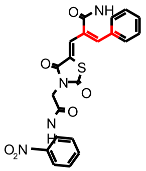 [*]C(=C[c](:[*]):[*])[*]	0.434	5 out of 9
SCFP_6	1730407098	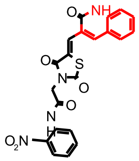 [*]C1=C[c]2:[cH]:[cH]:[cH]:[cH]:[c]:2N[*]1	0.415	1 out of 1

Top Features for negative contribution

Fingerprint	Bit/Smiles	Feature Structure	Score	Carcinogen in training set
SCFP_6	399659969	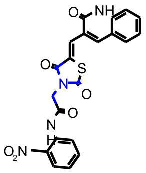 [*]CN1C(=[*])[*][*]C1=[*]	-0.578	1 out of 8
SCFP_6	2102703671	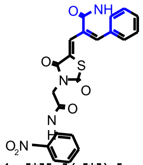 [*]C1=[*][c](:[*]):[c](NC1=O):[cH]:[*]	-0.496	0 out of 2
SCFP_6	-587539325	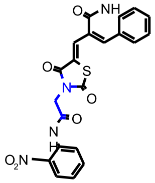 [*]N([*])CC(=[*])[*]	-0.264	1 out of 5

4 TOPKAT_Rat_Male_FDA_None_vs_Carcinogen

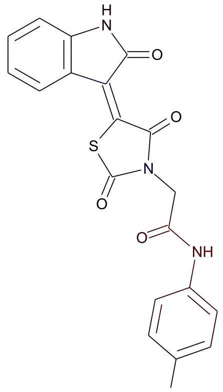

$C_{20}H_{15}N_3O_4S$
Molecular Weight: 393.4158
ALogP: 2.185
Rotatable Bonds: 3
Acceptors: 5
Donors: 2

Model Prediction

Prediction: Non-Carcinogen
Probability: 0.349
Enrichment: 1.04
Bayesian Score: -0.256
Mahalanobis Distance: 10
Mahalanobis Distance p-value: 0.647

Prediction: Positive if the Bayesian score is above the estimated best cutoff value from minimizing the false positive and false negative rate.
Probability: The esimated probability that the sample is in the positive category. This assumes that the Bayesian score follows a normal distribution and is different from the prediction using a cutoff.
Enrichment: An estimate of enrichment, that is, the increased likelihood (versus random) of this sample being in the category.
Bayesian Score: The standard Laplacian-modified Bayesian score.
Mahalanobis Distance: The Mahalanobis distance (MD) is the distance to the center of the training data. The larger the MD, the less trustworthy the prediction.
Mahalanobis Distance p-value: The p-value gives the fraction of training data with an MD greater than or equal to the one for the given sample, assuming normally distributed data. The smaller the p-value, the less trustworthy the prediciton. For highly non-normal X properties (e.g., fingerprints), the MD p-value is wildly inaccurate.

Structural Similar Compounds

Name	Metolazone	Indapamide	Acetohexamide
Structure	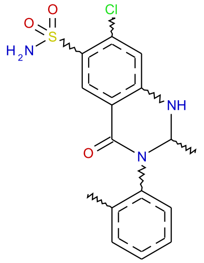	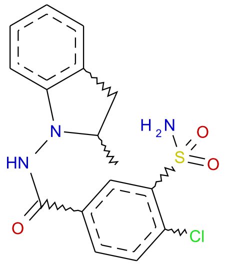	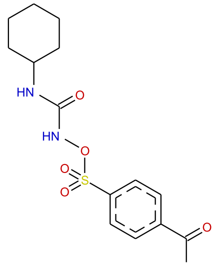
Actual Endpoint	Non-Carcinogen	Non-Carcinogen	Non-Carcinogen
Predicted Endpoint	Non-Carcinogen	Non-Carcinogen	Non-Carcinogen
Distance	0.554	0.559	0.609
Reference	US FDA (Centre for Drug Eval.& Res./Off. Testing & Res.) Sept. 1997	US FDA (Centre for Drug Eval.& Res./Off. Testing & Res.) Sept. 1997	US FDA (Centre for Drug Eval.& Res./Off. Testing & Res.) Sept. 1997

Model Applicability

Unknown features are fingerprint features in the query molecule, but not found or appearing too infrequently in the training set.

1. All properties and OPS components are within expected ranges.

Feature Contribution

Top features for positive contribution				
**Fingerprint**	**Bit/Smiles**	**Feature Structure**	**Score**	**Carcinogen in training set**
SCFP_6	-347048986	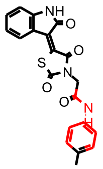 [*]C(=[*])N[c]1:[cH]:[cH]:[*]:[cH]:[cH]:1	0.615	5 out of 7

SCFP_6	814408713	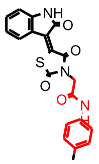 [*]CC(=O)N[c]1:[cH]:[cH]:[c]([*]):[cH]:[cH]:1	0.603	2 out of 2
SCFP_6	2097618059	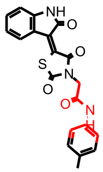 [*]CC(=O)N[c](:[cH]:[*]):[cH]:[*]	0.437	7 out of 13
**Top Features for negative contribution**				
**Fingerprint**	**Bit/Smiles**	**Feature Structure**	**Score**	**Carcinogen in training set**
SCFP_6	1798334293	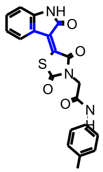 [*]C(=C1C(=[*])[*][*]:[c]1:[*])[*]	-0.674	0 out of 3
SCFP_6	399659969	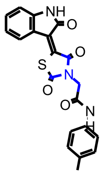 [*]CN1C(=[*])[*][*]C1=[*]	-0.578	1 out of 8
SCFP_6	2102703671	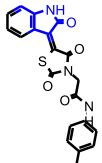 [*]C1=[*][c](:[*]):[c](NC1=O):[cH]:[*]	-0.496	0 out of 2

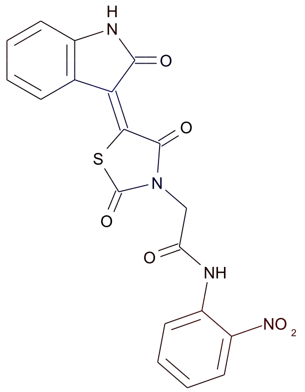

$C_{19}H_{12}N_4O_6S$
Molecular Weight: 424.38678
ALogP: 1.593
Rotatable Bonds: 4
Acceptors: 7
Donors: 2

Structural Similar Compounds

Name	Polythiazide	Penicillin	Nedocromil
Structure	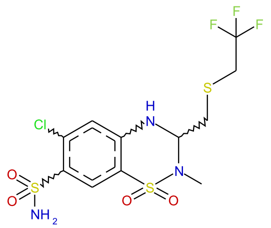	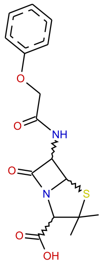	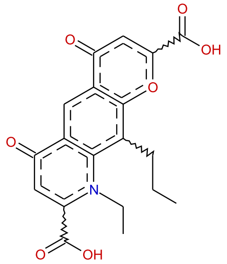
Actual Endpoint	Non-Carcinogen	Non-Carcinogen	Non-Carcinogen
Predicted Endpoint	Non-Carcinogen	Non-Carcinogen	Non-Carcinogen
Distance	0.582	0.662	0.670
Reference	US FDA (Centre for Drug Eval.& Res./Off. Testing & Res.) Sept. 1997	US FDA (Centre for Drug Eval.& Res./Off. Testing & Res.) Sept. 1997	US FDA (Centre for Drug Eval.& Res./Off. Testing & Res.) Sept. 1997

Model Prediction

Prediction: Carcinogen
Probability: 0.392
Enrichment: 1.17
Bayesian Score: 1.2
Mahalanobis Distance: 16
Mahalanobis Distance p-value: 4.9e-009

Prediction: Positive if the Bayesian score is above the estimated best cutoff value from minimizing the false positive and false negative rate.
Probability: The esimated probability that the sample is in the positive category. This assumes that the Bayesian score follows a normal distribution and is different from the prediction using a cutoff.
Enrichment: An estimate of enrichment, that is, the increased likelihood (versus random) of this sample being in the category.
Bayesian Score: The standard Laplacian-modified Bayesian score.
Mahalanobis Distance: The Mahalanobis distance (MD) is the distance to the center of the training data. The larger the MD, the less trustworthy the prediction.
Mahalanobis Distance p-value: The p-value gives the fraction of training data with an MD greater than or equal to the one for the given sample, assuming normally distributed data. The smaller the p-value, the less trustworthy the prediciton. For highly non-normal X properties (e.g., fingerprints), the MD p-value is wildly inaccurate.

Model Applicability

Unknown features are fingerprint features in the query molecule, but not found or appearing too infrequently in the training set.

1. OPS PC12 out of range. Value: -4.5232. Training min, max, SD, explained variance: -3.9196, 6.4101, 1.581, 0.0233.

Feature Contribution

Top features for positive contribution

Fingerprint	Bit/Smiles	Feature Structure	Score	Carcinogen in training set
SCFP_6	2097618059	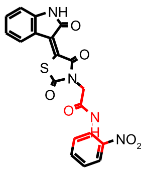 [*]CC(=O)N[c](:[cH]:[*]):[cH]:[*]	0.437	7 out of 13

SCFP_6	1311339974	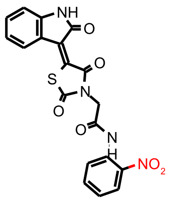 [*][N+](=O)[*]	0.405	13 out of 26
SCFP_6	-1380909229	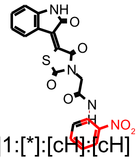 [*][c]1:[*]:[cH]:[cH] :[cH]:[c]:1[N+](=[*])[*]	0.287	17 out of 39
**Top Features for negative contribution**				
**Fingerprint**	**Bit/Smiles**	**Feature Structure**	**Score**	**Carcinogen in training set**
SCFP_6	1798334293	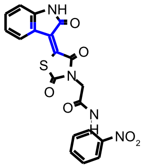 [*]C(=C1C(=[*])[*][*] :[c]1:[*])[*]	-0.674	0 out of 3
SCFP_6	399659969	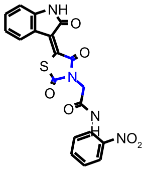 [*]CN1C(=[*])[*][*]C1 =[*]	-0.578	1 out of 8
SCFP_6	2102703671	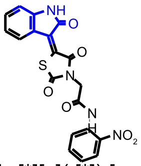 [*]C1=[*][c](:[*]):[c](NC1=O):[cH]:[*]	-0.496	0 out of 2

Sorafenib

TOPKAT_Rat_Male_FDA_None_vs_Carcinogen

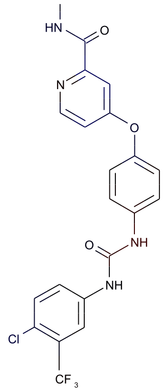

$C_{21}H_{16}ClF_3N_4O_3$
Molecular Weight: 464.82494
ALogP: 4.175
Rotatable Bonds: 6
Acceptors: 4
Donors: 3

Structural Similar Compounds

Name	Glyburide	Glimepride	Fluvastatin
Structure	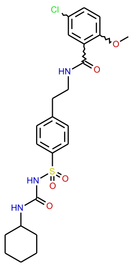	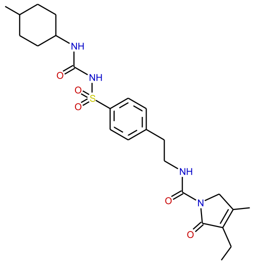	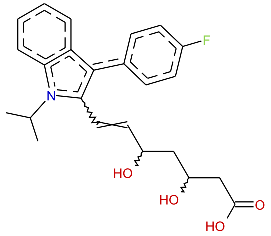
Actual Endpoint	Non-Carcinogen	Non-Carcinogen	Carcinogen
Predicted Endpoint	Non-Carcinogen	Non-Carcinogen	Carcinogen
Distance	0.593	0.600	0.615
Reference	US FDA (Centre for Drug Eval.& Res./Off. Testing & Res.) Sept. 1997	US FDA (Centre for Drug Eval.& Res./Off. Testing & Res.) Sept. 1997	US FDA (Centre for Drug Eval.& Res./Off. Testing & Res.) Sept. 1997

Model Prediction

Prediction: Non-Carcinogen
Probability: 0.293
Enrichment: 0.878
Bayesian Score: -2.4
Mahalanobis Distance: 17.6
Mahalanobis Distance p-value: 1.1e-012

Prediction: Positive if the Bayesian score is above the estimated best cutoff value from minimizing the false positive and false negative rate.
Probability: The esimated probability that the sample is in the positive category. This assumes that the Bayesian score follows a normal distribution and is different from the prediction using a cutoff.
Enrichment: An estimate of enrichment, that is, the increased likelihood (versus random) of this sample being in the category.
Bayesian Score: The standard Laplacian-modified Bayesian score.
Mahalanobis Distance: The Mahalanobis distance (MD) is the distance to the center of the training data. The larger the MD, the less trustworthy the prediction.
Mahalanobis Distance p-value: The p-value gives the fraction of training data with an MD greater than or equal to the one for the given sample, assuming normally distributed data. The smaller the p-value, the less trustworthy the prediciton. For highly non-normal X properties (e.g., fingerprints), the MD p-value is wildly inaccurate.

Model Applicability

Unknown features are fingerprint features in the query molecule, but not found or appearing too infrequently in the training set.

1. All properties and OPS components are within expected ranges.

Feature Contribution

Top features for positive contribution

Fingerprint	Bit/Smiles	Feature Structure	Score	Carcinogen in training set
SCFP_6	-347048986	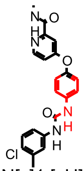 [*]C(=[*])N[c]:[cH]:[cH]:[*]:[cH]:[cH]:1	0.615	5 out of 7

SCFP_6	-754059116	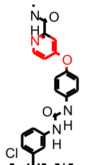<br>[*]O[c]1:[cH]:[*]:n:[cH]:[cH]:1	0.415	1 out of 1
SCFP_6	-347281112	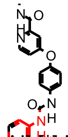<br>[*]N[c]1:[cH]:[*]:[c]([*]):[c](:[cH]:1)C([*])([*])[*]	0.273	2 out of 4
**Top Features for negative contribution**				
**Fingerprint**	**Bit/Smiles**	**Feature Structure**	**Score**	**Carcinogen in training set**
SCFP_6	-827073191	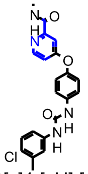<br>[*]C(=[*])[c]1:[cH]:[*]:[cH]:[cH]:n:1	-0.674	0 out of 3
SCFP_6	-975241316	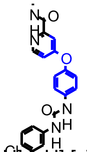<br>[*][c]1:[cH]:[cH]:[c](O[c](:[cH]:[*]):[cH]:[*]):[cH]:[cH]:1	-0.496	0 out of 2
SCFP_6	-488587948	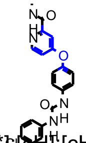<br>[*][c]1:[*]:[cH]:[cH]:[c](O[c](:[*]):[*]):[cH]:1	-0.496	0 out of 2

Sunitinib

TOPKAT_Rat_Male_FDA_None_vs_Carcinogen

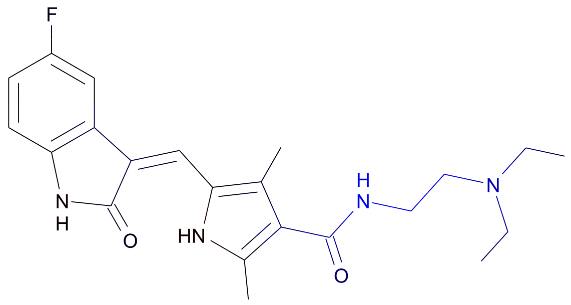

$C_{22}H_{27}FN_4O_2$
Molecular Weight: 398.47378
ALogP: 2.997
Rotatable Bonds: 7
Acceptors: 3
Donors: 3

Structural Similar Compounds

Name	Fluvastatin	Metoclopramide	Flecainide
Structure	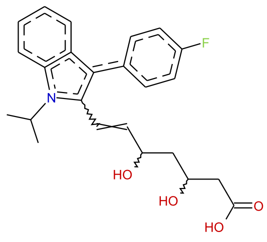	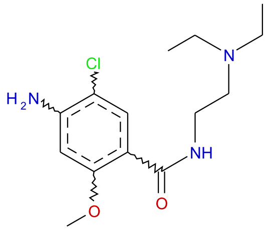	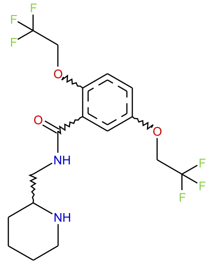
Actual Endpoint	Carcinogen	Non-Carcinogen	Non-Carcinogen
Predicted Endpoint	Carcinogen	Non-Carcinogen	Non-Carcinogen
Distance	0.599	0.634	0.643
Reference	US FDA (Centre for Drug Eval.& Res./Off. Testing & Res.) Sept. 1997	US FDA (Centre for Drug Eval.& Res./Off. Testing & Res.) Sept. 1997	US FDA (Centre for Drug Eval.& Res./Off. Testing & Res.) Sept. 1997

Model Prediction

Prediction: Non-Carcinogen
Probability: 0.217
Enrichment: 0.648
Bayesian Score: -6.26
Mahalanobis Distance: 15.7
Mahalanobis Distance p-value: 2.18e-008

Prediction: Positive if the Bayesian score is above the estimated best cutoff value from minimizing the false positive and false negative rate.
Probability: The esimated probability that the sample is in the positive category. This assumes that the Bayesian score follows a normal distribution and is different from the prediction using a cutoff.
Enrichment: An estimate of enrichment, that is, the increased likelihood (versus random) of this sample being in the category.
Bayesian Score: The standard Laplacian-modified Bayesian score.
Mahalanobis Distance: The Mahalanobis distance (MD) is the distance to the center of the training data. The larger the MD, the less trustworthy the prediction.
Mahalanobis Distance p-value: The p-value gives the fraction of training data with an MD greater than or equal to the one for the given sample, assuming normally distributed data. The smaller the p-value, the less trustworthy the prediciton. For highly non-normal X properties (e.g., fingerprints), the MD p-value is wildly inaccurate.

Model Applicability

Unknown features are fingerprint features in the query molecule, but not found or appearing too infrequently in the training set.

1. OPS PC28 out of range. Value: 3.8517. Training min, max, SD, explained variance: -3.142, 3.4791, 1.048, 0.0102.

Feature Contribution

Top features for positive contribution				
**Fingerprint**	**Bit/Smiles**	**Feature Structure**	**Score**	**Carcinogen in training set**
SCFP_6	-1971137145	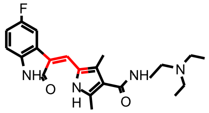 [*]C(=C[c](:[*]):[*])[*]	0.434	5 out of 9

SCFP_6	136686699	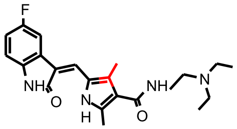 [*]:[c](:[*])C	0.287	17 out of 39
SCFP_6	-1375926917	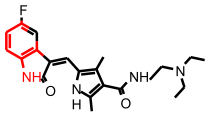 [*]N[c]1:[cH]:[cH]:[cH]:[*]:[c]:1[*]	0.251	11 out of 26
**Top Features for negative contribution**				
**Fingerprint**	**Bit/Smiles**	**Feature Structure**	**Score**	**Carcinogen in training set**
SCFP_6	1323614814	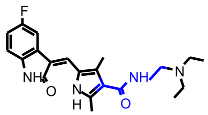 [*]CCNC(=O)[c](:[*]):[*]	-0.825	0 out of 4
SCFP_6	1798334293	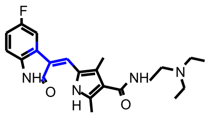 [*]C(=C1C(=[*])[*][*]:[c]1:[*])[*]	-0.674	0 out of 3
SCFP_6	-182283812	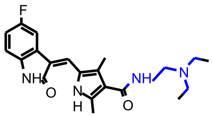 [*]CN(C[*])CCN[*]	-0.674	0 out of 3

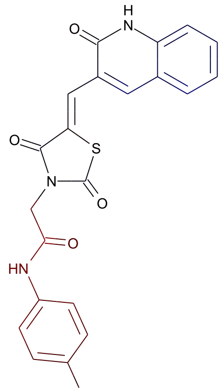

$C_{22}H_{17}N_3O_4S$
Molecular Weight: 419.45307
ALogP: 2.653
Rotatable Bonds: 4
Acceptors: 5
Donors: 2

Model Prediction

Prediction: Multiple-Carcinogen
Probability: 0.569
Enrichment: 1.37
Bayesian Score: 1.39
Mahalanobis Distance: 13.6
Mahalanobis Distance p-value: 0.000184

Prediction: Positive if the Bayesian score is above the estimated best cutoff value from minimizing the false positive and false negative rate.
Probability: The esimated probability that the sample is in the positive category. This assumes that the Bayesian score follows a normal distribution and is different from the prediction using a cutoff.
Enrichment: An estimate of enrichment, that is, the increased likelihood (versus random) of this sample being in the category.
Bayesian Score: The standard Laplacian-modified Bayesian score.
Mahalanobis Distance: The Mahalanobis distance (MD) is the distance to the center of the training data. The larger the MD, the less trustworthy the prediction.
Mahalanobis Distance p-value: The p-value gives the fraction of training data with an MD greater than or equal to the one for the given sample, assuming normally distributed data. The smaller the p-value, the less trustworthy the prediciton. For highly non-normal X properties (e.g., fingerprints), the MD p-value is wildly inaccurate.

Structural Similar Compounds

Name	Bicalutamide	Doxefazepam	Moricizine
Structure	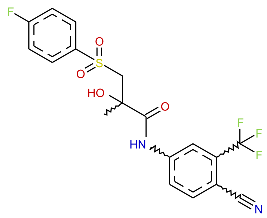	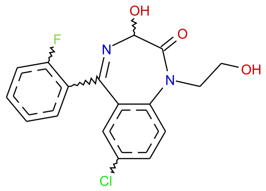	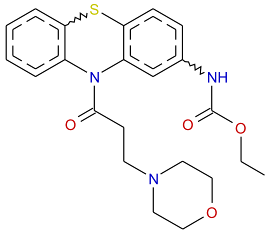
Actual Endpoint	Multiple-Carcinogen	Single-Carcinogen	Single-Carcinogen
Predicted Endpoint	Multiple-Carcinogen	Single-Carcinogen	Single-Carcinogen
Distance	0.588	0.648	0.715
Reference	US FDA (Centre for Drug Eval.& Res./Off. Testing & Res.) Sept. 1997	US FDA (Centre for Drug Eval.& Res./Off. Testing & Res.) Sept. 1997	US FDA (Centre for Drug Eval.& Res./Off. Testing & Res.) Sept. 1997

Model Applicability

Unknown features are fingerprint features in the query molecule, but not found or appearing too infrequently in the training set.

1. OPS PC18 out of range. Value: -3.1183. Training min, max, SD, explained variance: -2.5092, 3.7383, 1.072, 0.0156.

Feature Contribution

Top features for positive contribution

Fingerprint	Bit/Smiles	Feature Structure	Score	Multiple-Carcinogen in training set
SCFP_8	2097618059	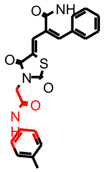 [*]CC(=O)N[c](:[cH]:[*]):[cH]:[*]	0.681	6 out of 7

SCFP_8	-347048986	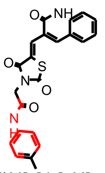 [*]C(=[*])N[c]1:[cH]:[cH]:[*]:[cH]:[cH]:1	0.574	4 out of 5
SCFP_8	814408713	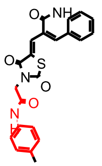 [*]CC(=O)N[c]1:[cH]:[cH]:[c]([*]):[cH]:[cH]:1	0.553	2 out of 2
**Top Features for negative contribution**				
**Fingerprint**	**Bit/Smiles**	**Feature Structure**	**Score**	**Multiple-Carcinogen in training set**
SCFP_8	1798274711	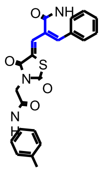 [*]\C=C(\C=[*])/C(=[*])[*]	-0.546	0 out of 2
SCFP_8	399659969	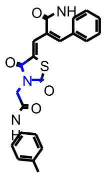 [*]CN1C(=[*])[*][*]C1=[*]	-0.31	0 out of 1
SCFP_8	1420330831	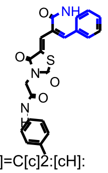 [*]=C1[*]=C[c]2:[cH]:[*]:[cH]:[cH]:[c]:2N1	-0.31	0 out of 1

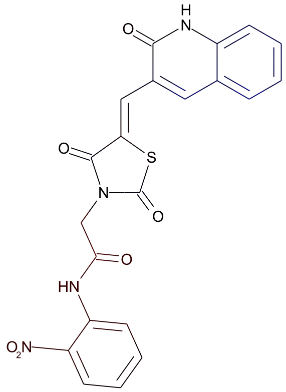

$C_{21}H_{14}N_4O_6S$
Molecular Weight: 450.42405
ALogP: 2.061
Rotatable Bonds: 5
Acceptors: 7
Donors: 2

Model Prediction

Prediction: Multiple-Carcinogen
Probability: 0.568
Enrichment: 1.37
Bayesian Score: 1.98
Mahalanobis Distance: 14
Mahalanobis Distance p-value: 8.67e-005

Prediction: Positive if the Bayesian score is above the estimated best cutoff value from minimizing the false positive and false negative rate.
Probability: The esimated probability that the sample is in the positive category. This assumes that the Bayesian score follows a normal distribution and is different from the prediction using a cutoff.
Enrichment: An estimate of enrichment, that is, the increased likelihood (versus random) of this sample being in the category.
Bayesian Score: The standard Laplacian-modified Bayesian score.
Mahalanobis Distance: The Mahalanobis distance (MD) is the distance to the center of the training data. The larger the MD, the less trustworthy the prediction.
Mahalanobis Distance p-value: The p-value gives the fraction of training data with an MD greater than or equal to the one for the given sample, assuming normally distributed data. The smaller the p-value, the less trustworthy the prediciton. For highly non-normal X properties (e.g., fingerprints), the MD p-value is wildly inaccurate.

Structural Similar Compounds

Name	Bicalutamide	Sulfasalazine	Budesonide
Structure	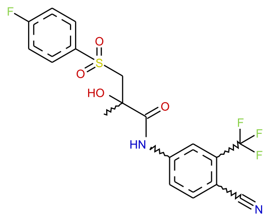	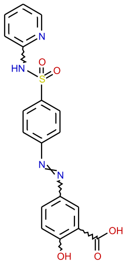	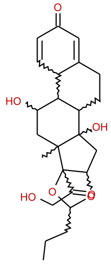
Actual Endpoint	Multiple-Carcinogen	Single-Carcinogen	Multiple-Carcinogen
Predicted Endpoint	Multiple-Carcinogen	Single-Carcinogen	Multiple-Carcinogen
Distance	0.721	0.749	0.766
Reference	US FDA (Centre for Drug Eval.& Res./Off. Testing & Res.) Sept. 1997	US FDA (Centre for Drug Eval.& Res./Off. Testing & Res.) Sept. 1997	US FDA (Centre for Drug Eval.& Res./Off. Testing & Res.) Sept. 1997

Model Applicability

Unknown features are fingerprint features in the query molecule, but not found or appearing too infrequently in the training set.

1. OPS PC14 out of range. Value: 3.313. Training min, max, SD, explained variance: -3.4626, 3.2359, 1.286, 0.0225.
2. OPS PC18 out of range. Value: -2.8129. Training min, max, SD, explained variance: -2.5092, 3.7383, 1.072, 0.0156.

Feature Contribution

Top features for positive contribution

Fingerprint	Bit/Smiles	Feature Structure	Score	Multiple-Carcinogen in training set
SCFP_8	2097618059	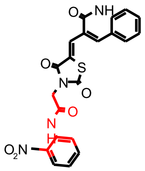 [*]CC(=O)N[c](:[cH]:[*]):[cH]:[*]	0.681	6 out of 7

SCFP_8	1631845520	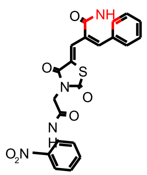 [*]C(=[*])N[c](:[*]):[*]	0.495	6 out of 9
SCFP_8	1311339974	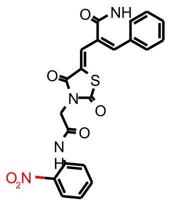 [*][N+](=O)[*]	0.453	8 out of 13
**Top Features for negative contribution**				
**Fingerprint**	**Bit/Smiles**	**Feature Structure**	**Score**	**Multiple-Carcinogen in training set**
SCFP_8	1798274711	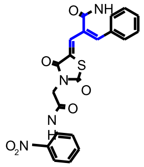 [*]\C=C(\C=[*])/C(=[*])[*]	-0.546	0 out of 2
SCFP_8	853077039	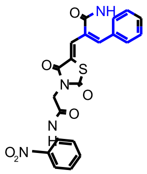 [*]C1=C[c]2:[cH]:[cH]:[*]:[cH]:[c]:2N[*]1	-0.31	0 out of 1
SCFP_8	1420330831	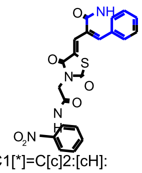 [*]=C1[*]=C[c]2:[cH]:[*]:[cH]:[cH]:[c]:2N1	-0.31	0 out of 1

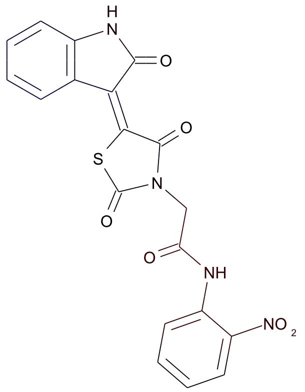

$C_{19}H_{12}N_4O_6S$

Molecular Weight: 424.38678

ALogP: 1.593

Rotatable Bonds: 4

Acceptors: 7

Donors: 2

Model Prediction

Prediction: Multiple-Carcinogen

Probability: 0.569

Enrichment: 1.37

Bayesian Score: 1.49

Mahalanobis Distance: 12.4

Mahalanobis Distance p-value: 0.00212

Prediction: Positive if the Bayesian score is above the estimated best cutoff value from minimizing the false positive and false negative rate.
Probability: The esimated probability that the sample is in the positive category. This assumes that the Bayesian score follows a normal distribution and is different from the prediction using a cutoff.
Enrichment: An estimate of enrichment, that is, the increased likelihood (versus random) of this sample being in the category.
Bayesian Score: The standard Laplacian-modified Bayesian score.
Mahalanobis Distance: The Mahalanobis distance (MD) is the distance to the center of the training data. The larger the MD, the less trustworthy the prediction.
Mahalanobis Distance p-value: The p-value gives the fraction of training data with an MD greater than or equal to the one for the given sample, assuming normally distributed data. The smaller the p-value, the less trustworthy the prediciton. For highly non-normal X properties (e.g., fingerprints), the MD p-value is wildly inaccurate.

Structural Similar Compounds

Name	Budesonide	Dihydroxymethylfuratrizine	Terazosin
Structure	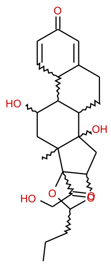	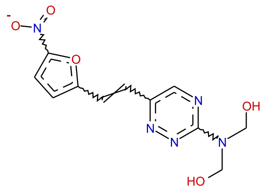	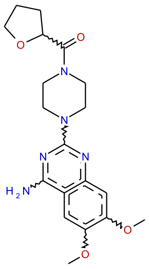
Actual Endpoint	Multiple-Carcinogen	Multiple-Carcinogen	Single-Carcinogen
Predicted Endpoint	Multiple-Carcinogen	Multiple-Carcinogen	Single-Carcinogen
Distance	0.767	0.772	0.777
Reference	US FDA (Centre for Drug Eval.& Res./Off. Testing & Res.) Sept. 1997	US FDA (Centre for Drug Eval.& Res./Off. Testing & Res.) Sept. 1997	US FDA (Centre for Drug Eval.& Res./Off. Testing & Res.) Sept. 1997

Model Applicability

Unknown features are fingerprint features in the query molecule, but not found or appearing too infrequently in the training set.

1. OPS PC18 out of range. Value: -3.1837. Training min, max, SD, explained variance: -2.5092, 3.7383, 1.072, 0.0156.

Feature Contribution

Top features for positive contribution

Fingerprint	Bit/Smiles	Feature Structure	Score	Multiple-Carcinogen in training set
SCFP_8	2097618059	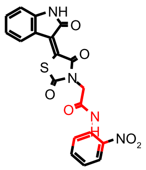 [*]CC(=O)N[c](:[cH]:[*]):[cH]:[*]	0.681	6 out of 7

SCFP_8	1631845520	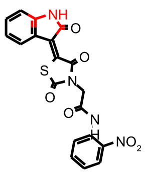 [*]C(=[*])N[c](:[*]):[*]	0.495	6 out of 9
SCFP_8	1311339974	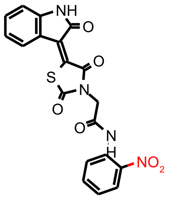 [*][N+](=O)[*]	0.453	8 out of 13

Top Features for negative contribution

Fingerprint	Bit/Smiles	Feature Structure	Score	Multiple-Carcinogen in training set
SCFP_8	1420330831	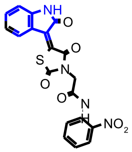 [*]=C1[*]=C[c]2:[cH]:[*]:[cH]:[cH]:[c]:2N1	-0.31	0 out of 1
SCFP_8	399659969	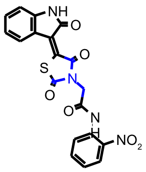 [*]CN1C(=[*])[*][*]C1=[*]	-0.31	0 out of 1
SCFP_8	-2056718782	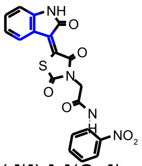 [*][c](:[*]):[c](C=[*]):[cH]:[*]	-0.29	6 out of 23

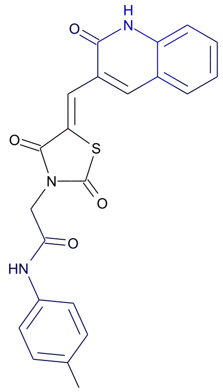

$C_{22}H_{17}N_3O_4S$
Molecular Weight: 419.45307
ALogP: 2.653
Rotatable Bonds: 4
Acceptors: 5
Donors: 2

Model Prediction

Prediction: Non-Irritant
Probability: 0.0864
Enrichment: 0.0938
Bayesian Score: -5.96
Mahalanobis Distance: 13.3
Mahalanobis Distance p-value: 1.08e-008

Prediction: Positive if the Bayesian score is above the estimated best cutoff value from minimizing the false positive and false negative rate.
Probability: The esimated probability that the sample is in the positive category. This assumes that the Bayesian score follows a normal distribution and is different from the prediction using a cutoff.
Enrichment: An estimate of enrichment, that is, the increased likelihood (versus random) of this sample being in the category.
Bayesian Score: The standard Laplacian-modified Bayesian score.
Mahalanobis Distance: The Mahalanobis distance (MD) is the distance to the center of the training data. The larger the MD, the less trustworthy the prediction.
Mahalanobis Distance p-value: The p-value gives the fraction of training data with an MD greater than or equal to the one for the given sample, assuming normally distributed data. The smaller the p-value, the less trustworthy the prediciton. For highly non-normal X properties (e.g., fingerprints), the MD p-value is wildly inaccurate.

Structural Similar Compounds

Name	2-Anthracenesulfonic acid, 1-amino-9,10-dihydro-9,10-dioxo-4-(2,4,6 -trimethylanilino)-, monosodium salt	5-Norbornene-2,3-dicarboxylic acid, 1,4,5,6,7,7-hexachloro-	Benzenesulfonic acid, 2-anilino-5-nitro-
Structure	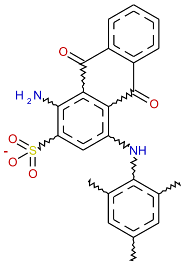	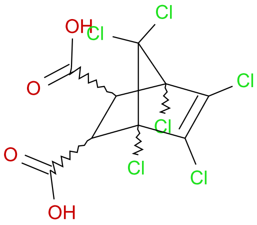	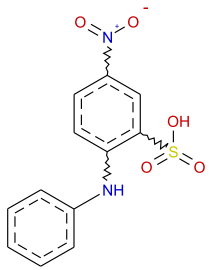
Actual Endpoint	Irritant	Irritant	Irritant
Predicted Endpoint	Non-Irritant	Irritant	Non-Irritant
Distance	0.688	0.781	0.792
Reference	85JCAE "Prehled Prumyslove Toxikologie; Organicke Latky," Marhold, J., Prague , Czechoslovakia, Avicenum, 1986 Volume(issue)/page/year: -,1327,1986	85JCAE "Prehled Prumyslove Toxikologie; Organicke Latky," Marhold, J., Prague , Czechoslovakia, Avicenum, 1986 Volume(issue)/page/year: -,581,1986	85JCAE "Prehled Prumyslove Toxikologie; Organicke Latky," Marhold, J., Prague , Czechoslovakia, Avicenum, 1986 Volume(issue)/page/year: -,1061,1986

Model Applicability

Unknown features are fingerprint features in the query molecule, but not found or appearing too infrequently in the training set.

1. All properties and OPS components are within expected ranges.

Feature Contribution

Top features for positive contribution				
**Fingerprint**	**Bit/Smiles**	**Feature Structure**	**Score**	**Irritant in training set**

FCFP_12	-1986158408	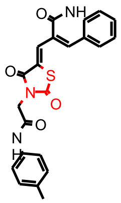 [*]N1[*][*]SC1=O	0.0821	13 out of 13
FCFP_12	436886043	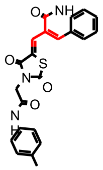 [*]\C=C(\C=[*])/C(=[*])[*]	0.0804	129 out of 130
FCFP_12	1383817444	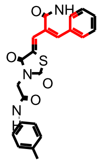 [*]=CC1=C[c](:[cH]:[*]):[c](:[*])[*]C1=[*]	0.0772	7 out of 7
**Top Features for negative contribution**				
**Fingerprint**	**Bit/Smiles**	**Feature Structure**	**Score**	**Irritant in training set**
FCFP_12	1175665944	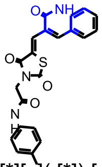 [*]C1=[*][c](:[*]):[c](NC1=O):[cH]:[*]	-1.02	2 out of 8
FCFP_12	-1838187238	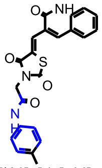 [*]C(=[*])N[c]1:[cH]:[cH]:[*]:[cH]:[cH]:1	-0.692	5 out of 12

FCFP_12	-451043714	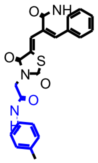 [*]CC(=O)N[c]1:[cH]:[cH]:[c]([*]):[cH]:[cH]:1	-0.65	0 out of 1

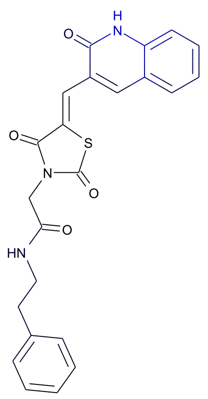

$C_{23}H_{19}N_3O_4S$
Molecular Weight: 433.47965
ALogP: 2.495
Rotatable Bonds: 6
Acceptors: 5
Donors: 2

Structural Similar Compounds

Name	2-Anthracenesulfonic acid, 1-amino-9,10-dihydro-9,10-dioxo-4-(2,4,6 -trimethylanilino)-, monosodium salt	Pregna-1,4-diene-3,20-dione, 21-(acetyloxy)-11-hydroxy-6-methyl-17- (1-oxopropoxy)-, (6-alpha,11-beta)-	5-Norbornene-2,3-dicarboxylic acid, 1,4,5,6,7,7-hexachloro-
Structure	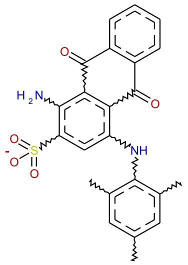	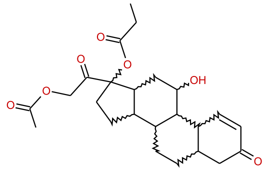	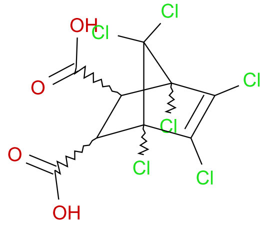
Actual Endpoint	Irritant	Irritant	Irritant
Predicted Endpoint	Non-Irritant	Irritant	Irritant
Distance	0.727	0.780	0.824
Reference	85JCAE "Prehled Prumyslove Toxikologie; Organicke Latky," Marhold, J., Prague , Czechoslovakia, Avicenum, 1986 Volume(issue)/page/year: -,1327,1986	YACHDS Yakuri to Chiryo. Pharmacology and Therapeutics. (Raifu Saiensu Shup pan K.K., 2-5-13, Yaesu, Chuo-ku, Tokyo 104, Japan) V.1-1972- Volume(issue) /page/year: 19,3103,1991	85JCAE "Prehled Prumyslove Toxikologie; Organicke Latky," Marhold, J., Prague , Czechoslovakia, Avicenum, 1986 Volume(issue)/page/year: -,581,1986

Model Prediction

Prediction: Non-Irritant
Probability: 0.259
Enrichment: 0.281
Bayesian Score: -5.22
Mahalanobis Distance: 14
Mahalanobis Distance p-value: 1.07e-010

Prediction: Positive if the Bayesian score is above the estimated best cutoff value from minimizing the false positive and false negative rate.
Probability: The esimated probability that the sample is in the positive category. This assumes that the Bayesian score follows a normal distribution and is different from the prediction using a cutoff.
Enrichment: An estimate of enrichment, that is, the increased likelihood (versus random) of this sample being in the category.
Bayesian Score: The standard Laplacian-modified Bayesian score.
Mahalanobis Distance: The Mahalanobis distance (MD) is the distance to the center of the training data. The larger the MD, the less trustworthy the prediction.
Mahalanobis Distance p-value: The p-value gives the fraction of training data with an MD greater than or equal to the one for the given sample, assuming normally distributed data. The smaller the p-value, the less trustworthy the prediciton. For highly non-normal X properties (e.g., fingerprints), the MD p-value is wildly inaccurate.

Model Applicability

Unknown features are fingerprint features in the query molecule, but not found or appearing too infrequently in the training set.

1. All properties and OPS components are within expected ranges.

Feature Contribution

Top features for positive contribution				
**Fingerprint**	**Bit/Smiles**	**Feature Structure**	**Score**	**Irritant in training set**

FCFP_12	-1986158408	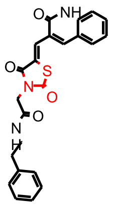 [*]N1[*][*]SC1=O	0.0821	13 out of 13
FCFP_12	436886043	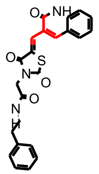 [*]\C=C(\C=[*])/C(=[*])[*]	0.0804	129 out of 130
FCFP_12	1383817444	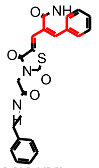 [*]=CC1=C[c](:[cH]:[*]):[c](:[*])[*]C1=[*]	0.0772	7 out of 7

Top Features for negative contribution

Fingerprint	Bit/Smiles	Feature Structure	Score	Irritant in training set
FCFP_12	1175665944	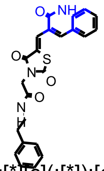 [*]C1=[*][c](:[*]):[c](NC1=O):[cH]:[*]	-1.02	2 out of 8
FCFP_12	-2002900105	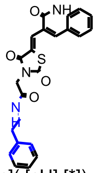 [*]NCC[c](:[cH]:[*]): [cH]:[*]	-0.65	0 out of 1

FCFP_12	1294255210	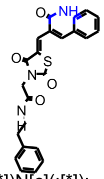 [*]C(=[*])N[c](:[*]):[*]	-0.486	12 out of 22

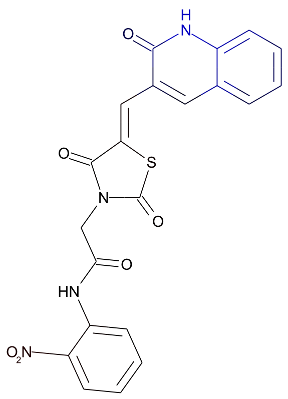

$C_{21}H_{14}N_4O_6S$

Molecular Weight: 450.42405

ALogP: 2.061

Rotatable Bonds: 5

Acceptors: 7

Donors: 2

Model Prediction

Prediction: Non-Irritant

Probability: 0.762

Enrichment: 0.828

Bayesian Score: -3.69

Mahalanobis Distance: 14.1

Mahalanobis Distance p-value: 5.39e-011

Prediction: Positive if the Bayesian score is above the estimated best cutoff value from minimizing the false positive and false negative rate.
Probability: The esimated probability that the sample is in the positive category. This assumes that the Bayesian score follows a normal distribution and is different from the prediction using a cutoff.
Enrichment: An estimate of enrichment, that is, the increased likelihood (versus random) of this sample being in the category.
Bayesian Score: The standard Laplacian-modified Bayesian score.
Mahalanobis Distance: The Mahalanobis distance (MD) is the distance to the center of the training data. The larger the MD, the less trustworthy the prediction.
Mahalanobis Distance p-value: The p-value gives the fraction of training data with an MD greater than or equal to the one for the given sample, assuming normally distributed data. The smaller the p-value, the less trustworthy the prediciton. For highly non-normal X properties (e.g., fingerprints), the MD p-value is wildly inaccurate.

Structural Similar Compounds

Name	2-Anthracenesulfonic acid, 1-amino-9,10-dihydro-9,10-dioxo-4-(2,4,6 -trimethylanilino)-, monosodium salt	2,2'-Stilbenedisulfonic acid, 4,4'-dinitro-	Urea, 1,3-bis(2-benzothiazolylthiomethyl)-
Structure	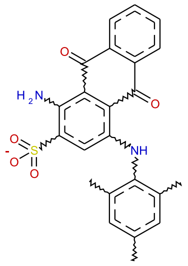	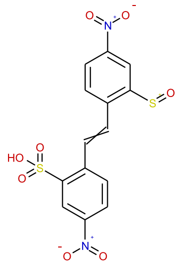	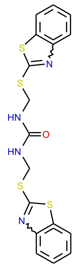
Actual Endpoint	Irritant	Irritant	Irritant
Predicted Endpoint	Non-Irritant	Irritant	Irritant
Distance	0.667	0.715	0.825
Reference	85JCAE "Prehled Prumyslove Toxikologie; Organicke Latky," Marhold, J., Prague , Czechoslovakia, Avicenum, 1986 Volume(issue)/page/year: -,1327,1986	85JCAE "Prehled Prumyslove Toxikologie; Organicke Latky," Marhold, J., Prague , Czechoslovakia, Avicenum, 1986 Volume(issue)/page/year: -,1062,1986	AMIHBC AMA Archives of Industrial Hygiene and Occupational Medicine. (Chicago , IL) V.2-10, 1950-54. For publisher information, see AEHLAU. Volume(issue)/pag e/year: 5,311,1952

Model Applicability

Unknown features are fingerprint features in the query molecule, but not found or appearing too infrequently in the training set.

1. All properties and OPS components are within expected ranges.

Feature Contribution

Top features for positive contribution				
Fingerprint	Bit/Smiles	Feature Structure	Score	Irritant in training set

FCFP_12	5	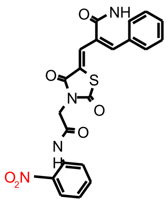 [*][O-]	0.0854	27 out of 27
FCFP_12	8	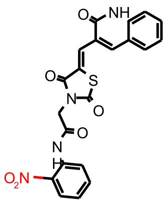 [*][N+](=[*])[*]	0.0843	20 out of 20
FCFP_12	-1986158408	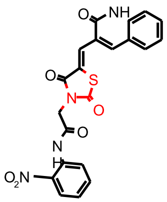 [*]N1[*][*]SC1=O	0.0821	13 out of 13

Top Features for negative contribution

Fingerprint	Bit/Smiles	Feature Structure	Score	Irritant in training set
FCFP_12	1175665944	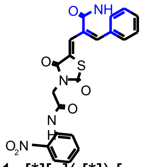 [*]C1=[*][c](:[*]):[c](NC1=O):[cH]:[*]	-1.02	2 out of 8
FCFP_12	1294255210	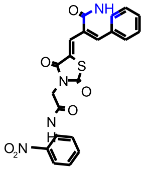 [*]C(=[*])N[c](:[*]): [*]	-0.486	12 out of 22

FCFP_12	-1724769936	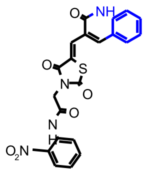 [*]N[c]1:[cH]:[cH]:[cH]:[cH]:[c]:1[*]	-0.475	11 out of 20

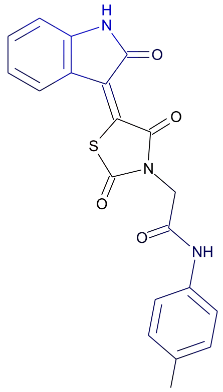

$C_{20}H_{15}N_3O_4S$
Molecular Weight: 393.4158
ALogP: 2.185
Rotatable Bonds: 3
Acceptors: 5
Donors: 2

Model Prediction

Prediction: Non-Irritant
Probability: 0.0516
Enrichment: 0.056
Bayesian Score: -6.25
Mahalanobis Distance: 11.2
Mahalanobis Distance p-value: 0.00128

Prediction: Positive if the Bayesian score is above the estimated best cutoff value from minimizing the false positive and false negative rate.
Probability: The esimated probability that the sample is in the positive category. This assumes that the Bayesian score follows a normal distribution and is different from the prediction using a cutoff.
Enrichment: An estimate of enrichment, that is, the increased likelihood (versus random) of this sample being in the category.
Bayesian Score: The standard Laplacian-modified Bayesian score.
Mahalanobis Distance: The Mahalanobis distance (MD) is the distance to the center of the training data. The larger the MD, the less trustworthy the prediction.
Mahalanobis Distance p-value: The p-value gives the fraction of training data with an MD greater than or equal to the one for the given sample, assuming normally distributed data. The smaller the p-value, the less trustworthy the prediciton. For highly non-normal X properties (e.g., fingerprints), the MD p-value is wildly inaccurate.

Structural Similar Compounds

Name	2-Anthracenesulfonic acid, 1-amino-9,10-dihydro-9,10-dioxo-4-(2,4,6 -trimethylanilino)-, monosodium salt	Benzenesulfonic acid, 2-anilino-5-nitro-	1-Amino-2-bromo-4-hydroxyanthraquinone
Structure	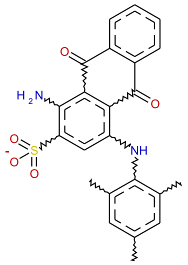	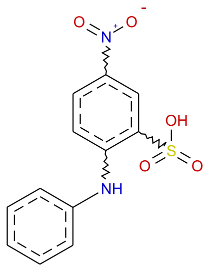	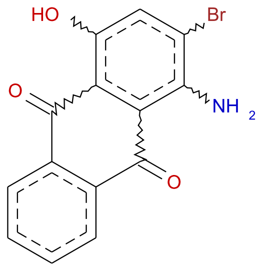
Actual Endpoint	Irritant	Irritant	Non-Irritant
Predicted Endpoint	Non-Irritant	Non-Irritant	Non-Irritant
Distance	0.687	0.732	0.770
Reference	85JCAE "Prehled Prumyslove Toxikologie; Organicke Latky," Marhold, J., Prague , Czechoslovakia, Avicenum, 1986 Volume(issue)/page/year: -,1327,1986	85JCAE "Prehled Prumyslove Toxikologie; Organicke Latky," Marhold, J., Prague , Czechoslovakia, Avicenum, 1986 Volume(issue)/page/year: -,1061,1986	28ZPAK -,83,72

Model Applicability

Unknown features are fingerprint features in the query molecule, but not found or appearing too infrequently in the training set.

1. All properties and OPS components are within expected ranges.

Feature Contribution

Top features for positive contribution				
**Fingerprint**	**Bit/Smiles**	**Feature Structure**	**Score**	**Irritant in training set**

FCFP_12	-1986158408	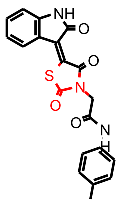<br>[*]N1[*][*]SC1=O	0.0821	13 out of 13
FCFP_12	436915834	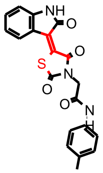<br>[*]\C=C\1/S[*][*]C1=[<br>*]	0.0756	6 out of 6
FCFP_12	-1143715940	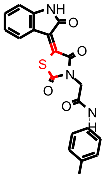<br>[*]=C1[*][*]C(=[*])S1	0.0575	475 out of 490

Top Features for negative contribution

Fingerprint	Bit/Smiles	Feature Structure	Score	Irritant in training set
FCFP_12	1175665944	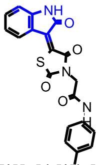<br>[*]C1=[*][c](:[*]):[c<br>](NC1=O):[cH]:[*]	-1.02	2 out of 8
FCFP_12	-1838187238	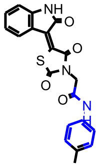<br>[*]C(=[*])N[c]1:[cH]:<br>[cH]:[*]:[cH]:[cH]:1	-0.692	5 out of 12

FCFP_12	-451043714	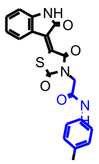 [*]CC(=O)N[c]1:[cH]:[cH]:[c]([*]):[cH]:[cH]:1	-0.65	0 out of 1

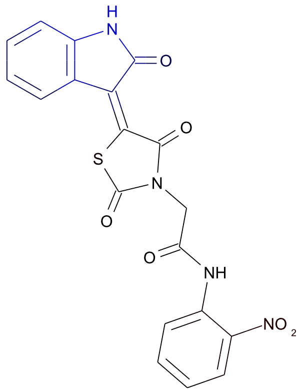

$C_{19}H_{12}N_4O_6S$
Molecular Weight: 424.38678
ALogP: 1.593
Rotatable Bonds: 4
Acceptors: 7
Donors: 2

Model Prediction

Prediction: Non-Irritant
Probability: 0.705
Enrichment: 0.766
Bayesian Score: -3.92
Mahalanobis Distance: 12
Mahalanobis Distance p-value: 3.14e-005

Prediction: Positive if the Bayesian score is above the estimated best cutoff value from minimizing the false positive and false negative rate.
Probability: The esimated probability that the sample is in the positive category. This assumes that the Bayesian score follows a normal distribution and is different from the prediction using a cutoff.
Enrichment: An estimate of enrichment, that is, the increased likelihood (versus random) of this sample being in the category.
Bayesian Score: The standard Laplacian-modified Bayesian score.
Mahalanobis Distance: The Mahalanobis distance (MD) is the distance to the center of the training data. The larger the MD, the less trustworthy the prediction.
Mahalanobis Distance p-value: The p-value gives the fraction of training data with an MD greater than or equal to the one for the given sample, assuming normally distributed data. The smaller the p-value, the less trustworthy the prediciton. For highly non-normal X properties (e.g., fingerprints), the MD p-value is wildly inaccurate.

Structural Similar Compounds

Name	2-Anthracenesulfonic acid, 1-amino-9,10-dihydro-9,10-dioxo-4-(2,4,6 -trimethylanilino)-, monosodium salt	2,2'-Stilbenedisulfonic acid, 4,4'-dinitro-	Benzenesulfonamide, 4-amino-N-(5,6-dimethoxy-4-pyrimidinyl)-
Structure	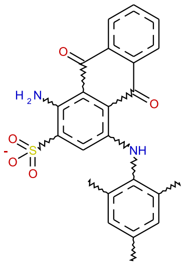	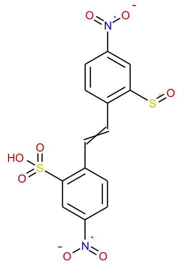	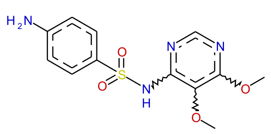
Actual Endpoint	Irritant	Irritant	Irritant
Predicted Endpoint	Non-Irritant	Irritant	Non-Irritant
Distance	0.677	0.705	0.798
Reference	85JCAE "Prehled Prumyslove Toxikologie; Organicke Latky," Marhold, J., Prague , Czechoslovakia, Avicenum, 1986 Volume(issue)/page/year: -,1327,1986	85JCAE "Prehled Prumyslove Toxikologie; Organicke Latky," Marhold, J., Prague , Czechoslovakia, Avicenum, 1986 Volume(issue)/page/year: -,1062,1986	FCTXAV 14,307,76

Model Applicability

Unknown features are fingerprint features in the query molecule, but not found or appearing too infrequently in the training set.

1. All properties and OPS components are within expected ranges.

Feature Contribution

Top features for positive contribution				
**Fingerprint**	**Bit/Smiles**	**Feature Structure**	**Score**	**Irritant in training set**

FCFP_12	5	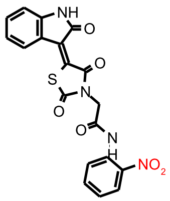 [*][O-]	0.0854	27 out of 27
FCFP_12	8	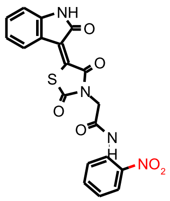 [*][N+](=[*])[*]	0.0843	20 out of 20
FCFP_12	-1986158408	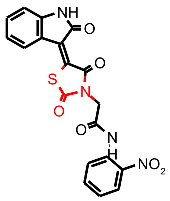 [*]N1[*][*]SC1=O	0.0821	13 out of 13

Top Features for negative contribution

Fingerprint	Bit/Smiles	Feature Structure	Score	Irritant in training set
FCFP_12	1175665944	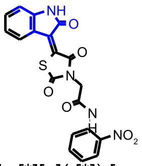 [*]C1=[*][c](:[*]):[c](NC1=O):[cH]:[*]	-1.02	2 out of 8
FCFP_12	1294255210	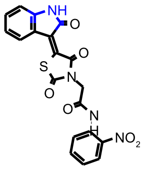 [*]C(=[*])N[c](:[*]): [*]	-0.486	12 out of 22

FCFP_12	-1724769936	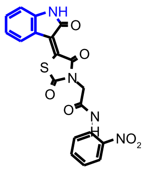 [*]N[c]1:[cH]:[cH]:[cH]:[cH]:[c]:1[*]	-0.475	11 out of 20

Sorafenib

TOPKAT_Skin_Irritancy_None_vs_Irritant

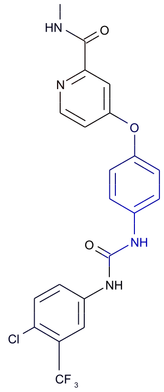

$C_{21}H_{16}ClF_3N_4O_3$
Molecular Weight: 464.82494
ALogP: 4.175
Rotatable Bonds: 6
Acceptors: 4
Donors: 3

Model Prediction

Prediction: Non-Irritant
Probability: 0.264
Enrichment: 0.287
Bayesian Score: -5.23
Mahalanobis Distance: 8.27
Mahalanobis Distance p-value: 0.791

Prediction: Positive if the Bayesian score is above the estimated best cutoff value from minimizing the false positive and false negative rate.
Probability: The esimated probability that the sample is in the positive category. This assumes that the Bayesian score follows a normal distribution and is different from the prediction using a cutoff.
Enrichment: An estimate of enrichment, that is, the increased likelihood (versus random) of this sample being in the category.
Bayesian Score: The standard Laplacian-modified Bayesian score.
Mahalanobis Distance: The Mahalanobis distance (MD) is the distance to the center of the training data. The larger the MD, the less trustworthy the prediction.
Mahalanobis Distance p-value: The p-value gives the fraction of training data with an MD greater than or equal to the one for the given sample, assuming normally distributed data. The smaller the p-value, the less trustworthy the prediciton. For highly non-normal X properties (e.g., fingerprints), the MD p-value is wildly inaccurate.

Structural Similar Compounds

Name	5-Norbornene-2,3-dicarboxylic acid, 1,4,5,6,7,7-hexachloro-	Benzenesulfonic acid, 2,2'-(4,4'-biphenylylenedivinylene)di-, disod ium salt	Sulfide, bis(4-t-butyl-m-cresyl)-
Structure	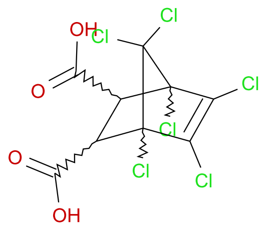	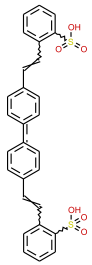	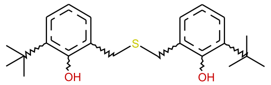
Actual Endpoint	Irritant	Irritant	Irritant
Predicted Endpoint	Irritant	Non-Irritant	Irritant
Distance	0.844	0.871	0.884
Reference	85JCAE "Prehled Prumyslove Toxikologie; Organicke Latky," Marhold, J., Prague , Czechoslovakia, Avicenum, 1986 Volume(issue)/page/year: -,581,1986	MVCRB3 MVC-Report. (Stockholm, Sweden) No.1-2, 1972-73. Discontinued. Volu me(issue)/page/year: 2,193,1973	AMIHBC AMA Archives of Industrial Hygiene and Occupational Medicine. (Chicago , IL) V.2-10, 1950-54. For publisher information, see AEHLAU. Volume(issue)/pag e/year: 5,311,1952

Model Applicability

Unknown features are fingerprint features in the query molecule, but not found or appearing too infrequently in the training set.

1. All properties and OPS components are within expected ranges.

Feature Contribution

Top features for positive contribution				
Fingerprint	Bit/Smiles	Feature Structure	Score	Irritant in training set

FCFP_12	-124655670	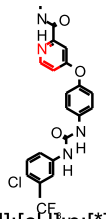 [*]:[cH]:[cH]:n:[*]	0.0821	13 out of 13
FCFP_12	-1539132615	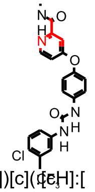 [*]C(=[*])[c](:[cH]:[*]):n:[*]	0.0795	9 out of 9
FCFP_12	-1695756380	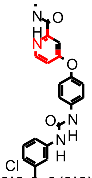 [*][c]1:[*]:[c]([*]):n:[cH]:[cH]:1	0.0772	7 out of 7

Top Features for negative contribution

Fingerprint	Bit/Smiles	Feature Structure	Score	Irritant in training set
FCFP_12	-789307649	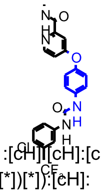 [*]O[c]1:[cH]:[cH]:[c](NC(=[*])[*]):[cH]:[cH]:1	-1.54	0 out of 4
FCFP_12	-1838187238	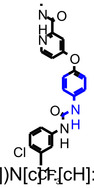 [*]C(=[*])N[c]1:[cH]:[cH]:[*]:[cH]:[cH]:1	-0.692	5 out of 12

FCFP_12	1294255210	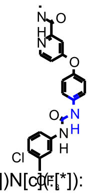 [*]C(=[*])N[c](:[*]):[*]	-0.486	12 out of 22

Sunitinib

TOPKAT_Skin_Irritancy_None_vs_Irritant

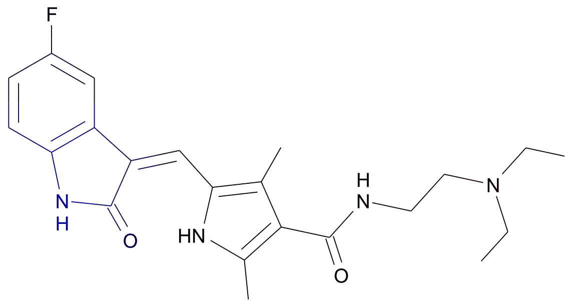

$C_{22}H_{27}FN_4O_2$

Molecular Weight: 398.47378

ALogP: 2.997

Rotatable Bonds: 7

Acceptors: 3

Donors: 3

Model Prediction

Prediction: Non-Irritant

Probability: 0.856

Enrichment: 0.93

Bayesian Score: -3.16

Mahalanobis Distance: 10.1

Mahalanobis Distance p-value: 0.0541

Prediction: Positive if the Bayesian score is above the estimated best cutoff value from minimizing the false positive and false negative rate.
Probability: The esimated probability that the sample is in the positive category. This assumes that the Bayesian score follows a normal distribution and is different from the prediction using a cutoff.
Enrichment: An estimate of enrichment, that is, the increased likelihood (versus random) of this sample being in the category.
Bayesian Score: The standard Laplacian-modified Bayesian score.
Mahalanobis Distance: The Mahalanobis distance (MD) is the distance to the center of the training data. The larger the MD, the less trustworthy the prediction.
Mahalanobis Distance p-value: The p-value gives the fraction of training data with an MD greater than or equal to the one for the given sample, assuming normally distributed data. The smaller the p-value, the less trustworthy the prediciton. For highly non-normal X properties (e.g., fingerprints), the MD p-value is wildly inaccurate.

Structural Similar Compounds

Name	5-Norbornene-2,3-dicarboxylic acid, 1,4,5,6,7,7-hexachloro-	Sulfide, bis(4-t-butyl-m-cresyl)-	p-Acetophenetidide, 3'-(bis(2-hydroxyethyl)amino)-
Structure	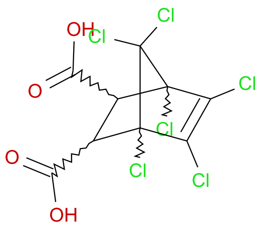	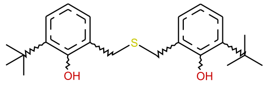	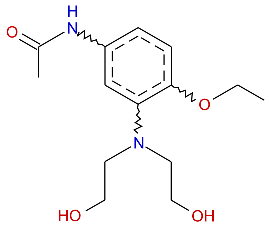
Actual Endpoint	Irritant	Irritant	Non-Irritant
Predicted Endpoint	Irritant	Irritant	Non-Irritant
Distance	0.818	0.860	0.863
Reference	85JCAE "Prehled Prumyslove Toxikologie; Organicke Latky," Marhold, J., Prague , Czechoslovakia, Avicenum, 1986 Volume(issue)/page/year: -,581,1986	AMIHBC AMA Archives of Industrial Hygiene and Occupational Medicine. (Chicago , IL) V.2-10, 1950-54. For publisher information, see AEHLAU. Volume(issue)/pag e/year: 5,311,1952	28ZPAK -,100,72

Model Applicability

Unknown features are fingerprint features in the query molecule, but not found or appearing too infrequently in the training set.

1. All properties and OPS components are within expected ranges.
2. Unknown FCFP_2 feature: 203707511: [*][c]1:[*]:[*]:[nH]:[c]:1C

Feature Contribution

Top features for positive contribution

Fingerprint	Bit/Smiles	Feature Structure	Score	Irritant in training set

FCFP_12	1852108031	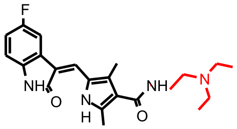<br>[*]CCN(CC)CC	0.0841	19 out of 19
FCFP_12	1851332093	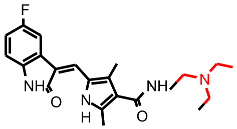<br>[*]CN(C[*])CC	0.0795	9 out of 9
FCFP_12	-371808660	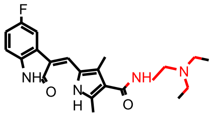<br>[*]CN(C[*])CCN[*]	0.0785	8 out of 8
**Top Features for negative contribution**				
**Fingerprint**	**Bit/Smiles**	**Feature Structure**	**Score**	**Irritant in training set**
FCFP_12	1175665944	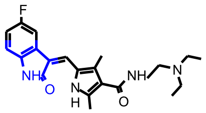<br>[*]C1=[*][c](:[*]):[c](NC1=O):[cH]:[*]	-1.02	2 out of 8
FCFP_12	1294255210	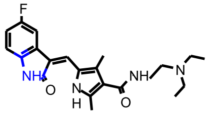<br>[*]C(=[*])N[c](:[*]): [*]	-0.486	12 out of 22

FCFP_12	-773983804	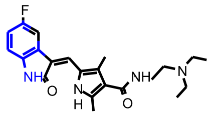 [*]N[c]1:[cH]:[cH]:[cH]:[*]:[c]:1[*]	-0.444	46 out of 79

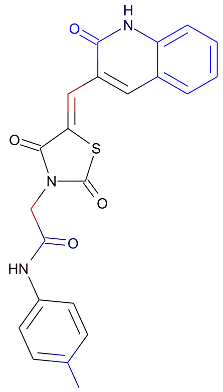

$C_{22}H_{17}N_3O_4S$
Molecular Weight: 419.45307
ALogP: 2.653
Rotatable Bonds: 4
Acceptors: 5
Donors: 2

Structural Similar Compounds

Name	Ochratoxin A	542	470
Structure	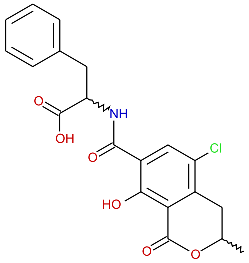	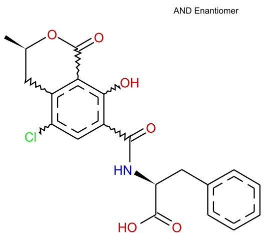	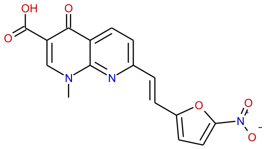
Actual Endpoint (-log C)	4.79932	4.79932	4.62839
Predicted Endpoint (-log C)	3.6353	3.6353	3.93264
Distance	0.651	0.651	0.704
Reference	CPDB	CPDB	CPDB

Model Prediction

Prediction: 51.6
Unit: mg/kg_body_weight/day
Mahalanobis Distance: 12.8
Mahalanobis Distance p-value: 4.74e-007

Mahalanobis Distance: The Mahalanobis distance (MD) is a generalization of the Euclidean distance that accounts for correlations among the X properties. It is calculated as the distance to the center of the training data. The larger the MD, the less trustworthy the prediction.
Mahalanobis Distance p-value: The p-value gives the fraction of training data with an MD greater than or equal to the one for the given sample, assuming normally distributed data. The smaller the p-value, the less trustworthy the prediciton. For highly non-normal X properties (e.g., fingerprints), the MD p-value is wildly inaccurate.

Model Applicability

Unknown features are fingerprint features in the query molecule, but not found or appearing too infrequently in the training set.

1. All properties and OPS components are within expected ranges.
2. Unknown ECFP_2 feature: 2131425032: [*]\C=C(\C=[*])/C(=[*])[*]
3. Unknown ECFP_2 feature: 1182722866: [*]C(=CC(=[*])[*])[*]
4. Unknown ECFP_2 feature: 1000552169: [*]\C=C\1/S[*][*]C1=[*]
5. Unknown ECFP_2 feature: -661097313: [*]CN1C(=[*])[*][*]C1=[*]
6. Unknown ECFP_2 feature: -37698365: [*]N([*])CC(=[*])[*]

Feature Contribution

Top features for positive contribution			
Fingerprint	Bit/Smiles	Feature Structure	Score
ECFP_6	1559650422	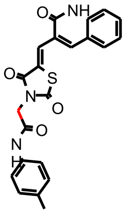 [*]C[*]	0.203

ECFP_6	-1925046727	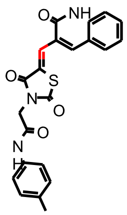 [*]C=[*]	0.145
ECFP_6	734603939	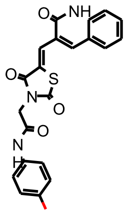 [*]C	0.0424
**Top Features for negative contribution**			
**Fingerprint**	**Bit/Smiles**	**Feature Structure**	**Score**
ECFP_6	2106656448	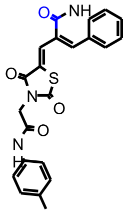 [*]C(=O)[*]	-0.275
ECFP_6	1996767644	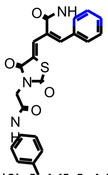 [*][c](:[*]):[cH]:[cH]:[*]	-0.251
ECFP_6	642810091	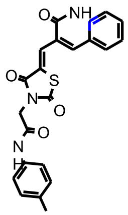 [*][c](:[*]):[*]	-0.247

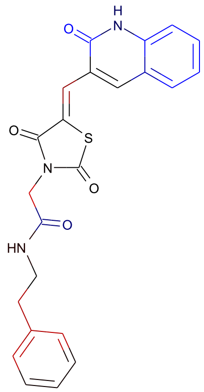

$C_{23}H_{19}N_3O_4S$

Molecular Weight: 433.47965

ALogP: 2.495

Rotatable Bonds: 6

Acceptors: 5

Donors: 2

Model Prediction

Prediction: 33.3

Unit: mg/kg_body_weight/day

Mahalanobis Distance: 11.6

Mahalanobis Distance p-value: 0.000156

Mahalanobis Distance: The Mahalanobis distance (MD) is a generalization of the Euclidean distance that accounts for correlations among the X properties. It is calculated as the distance to the center of the training data. The larger the MD, the less trustworthy the prediction.
Mahalanobis Distance p-value: The p-value gives the fraction of training data with an MD greater than or equal to the one for the given sample, assuming normally distributed data. The smaller the p-value, the less trustworthy the prediciton. For highly non-normal X properties (e.g., fingerprints), the MD p-value is wildly inaccurate.

Structural Similar Compounds

Name	Ochratoxin A	542	470
Structure	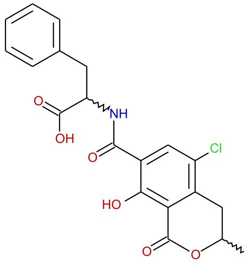	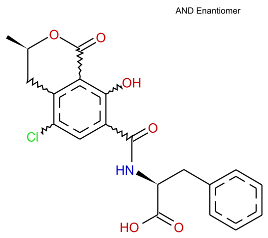	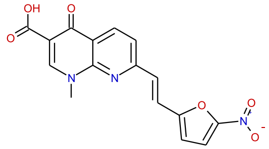
Actual Endpoint (-log C)	4.79932	4.79932	4.62839
Predicted Endpoint (-log C)	3.6353	3.6353	3.93264
Distance	0.641	0.641	0.732
Reference	CPDB	CPDB	CPDB

Model Applicability

Unknown features are fingerprint features in the query molecule, but not found or appearing too infrequently in the training set.

1. All properties and OPS components are within expected ranges.
2. Unknown ECFP_2 feature: 2131425032: [*]\C=C(\C=[*])/C(=[*])[*]
3. Unknown ECFP_2 feature: 1182722866: [*]C(=CC(=[*])[*])[*]
4. Unknown ECFP_2 feature: 1000552169: [*]\C=C\1/S[*][*]C1=[*]
5. Unknown ECFP_2 feature: -661097313: [*]CN1C(=[*])[*][*]C1=[*]
6. Unknown ECFP_2 feature: -37698365: [*]N([*])CC(=[*])[*]

Feature Contribution

Top features for positive contribution			
**Fingerprint**	**Bit/Smiles**	**Feature Structure**	**Score**
ECFP_6	1559650422	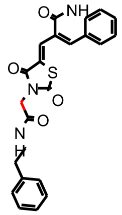 [*]C[*]	0.203

ECFP_6	-2024255407	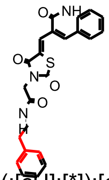 [*]C[c](:[cH]:[*]):[cH]:[*]	0.172
ECFP_6	-1925046727	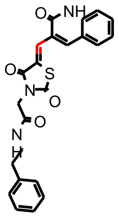 [*]C=[*]	0.145
**Top Features for negative contribution**			
**Fingerprint**	**Bit/Smiles**	**Feature Structure**	**Score**
ECFP_6	2106656448	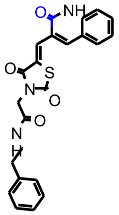 [*]C(=O)[*]	-0.275
ECFP_6	1996767644	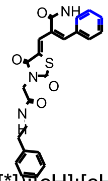 [*][c](:[*]):[cH]:[cH]:[*]	-0.251
ECFP_6	642810091	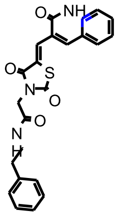 [*][c](:[*]):[*]	-0.247

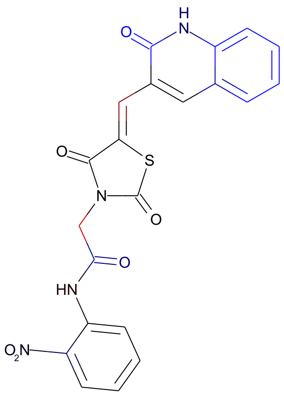

$C_{21}H_{14}N_4O_6S$
Molecular Weight: 450.42405
ALogP: 2.061
Rotatable Bonds: 5
Acceptors: 7
Donors: 2

Model Prediction

Prediction: 47.4
Unit: mg/kg_body_weight/day
Mahalanobis Distance: 12.8
Mahalanobis Distance p-value: 3.57e-007

Mahalanobis Distance: The Mahalanobis distance (MD) is a generalization of the Euclidean distance that accounts for correlations among the X properties. It is calculated as the distance to the center of the training data. The larger the MD, the less trustworthy the prediction.
Mahalanobis Distance p-value: The p-value gives the fraction of training data with an MD greater than or equal to the one for the given sample, assuming normally distributed data. The smaller the p-value, the less trustworthy the prediciton. For highly non-normal X properties (e.g., fingerprints), the MD p-value is wildly inaccurate.

Structural Similar Compounds

Name	Salicylazosulfapyridine	470	Ochratoxin A
Structure	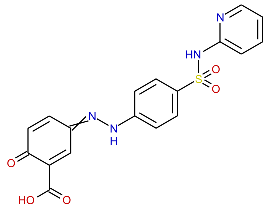	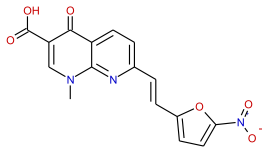	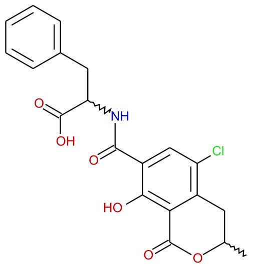
Actual Endpoint (-log C)	2.5034	4.62839	4.79932
Predicted Endpoint (-log C)	3.54214	3.93264	3.6353
Distance	0.673	0.715	0.761
Reference	CPDB	CPDB	CPDB

Model Applicability

Unknown features are fingerprint features in the query molecule, but not found or appearing too infrequently in the training set.

1. All properties and OPS components are within expected ranges.
2. Unknown ECFP_2 feature: 2131425032: [*]\C=C(\C=[*])/C(=[*])[*]
3. Unknown ECFP_2 feature: 1182722866: [*]C(=CC(=[*])[*])[*]
4. Unknown ECFP_2 feature: 1000552169: [*]\C=C\1/S[*][*]C1=[*]
5. Unknown ECFP_2 feature: -661097313: [*]CN1C(=[*])[*][*]C1=[*]
6. Unknown ECFP_2 feature: -37698365: [*]N([*])CC(=[*])[*]

Feature Contribution

Top features for positive contribution			
Fingerprint	Bit/Smiles	Feature Structure	Score
ECFP_6	1559650422	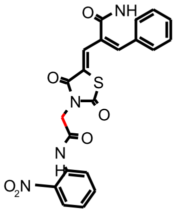 [*]C[*]	0.203

ECFP_6	-1925046727	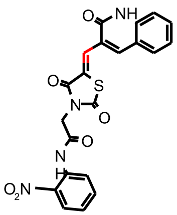 [*]C=[*]	0.145
ECFP_6	781519895	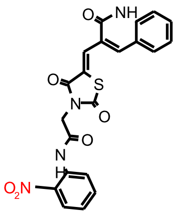 [*][O-]	0.0797

Top Features for negative contribution

Fingerprint	Bit/Smiles	Feature Structure	Score
ECFP_6	2106656448	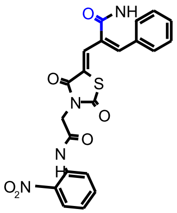 [*]C(=O)[*]	-0.275
ECFP_6	1996767644	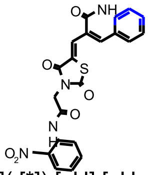 [*][c](:[*]):[cH]:[cH]:[*]	-0.251
ECFP_6	642810091	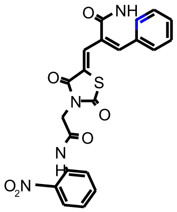 [*][c](:[*]):[*]	-0.247

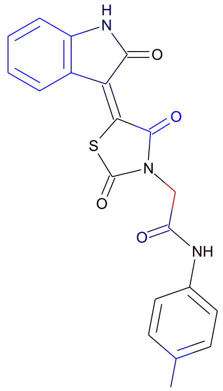

$C_{20}H_{15}N_3O_4S$
Molecular Weight: 393.4158
ALogP: 2.185
Rotatable Bonds: 3
Acceptors: 5
Donors: 2

Model Prediction

Prediction: 91.8
Unit: mg/kg_body_weight/day
Mahalanobis Distance: 12.7
Mahalanobis Distance p-value: 7.34e-007

Mahalanobis Distance: The Mahalanobis distance (MD) is a generalization of the Euclidean distance that accounts for correlations among the X properties. It is calculated as the distance to the center of the training data. The larger the MD, the less trustworthy the prediction.
Mahalanobis Distance p-value: The p-value gives the fraction of training data with an MD greater than or equal to the one for the given sample, assuming normally distributed data. The smaller the p-value, the less trustworthy the prediciton. For highly non-normal X properties (e.g., fingerprints), the MD p-value is wildly inaccurate.

Structural Similar Compounds

Name	542	Ochratoxin A	470
Structure	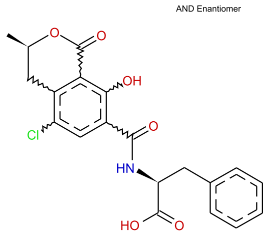	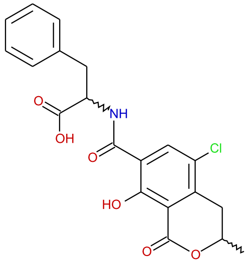	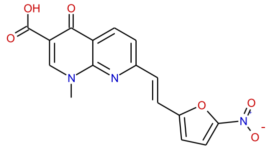
Actual Endpoint (-log C)	4.79932	4.79932	4.62839
Predicted Endpoint (-log C)	3.6353	3.6353	3.93264
Distance	0.674	0.674	0.697
Reference	CPDB	CPDB	CPDB

Model Applicability

Unknown features are fingerprint features in the query molecule, but not found or appearing too infrequently in the training set.

1. All properties and OPS components are within expected ranges.
2. Unknown ECFP_2 feature: -631778390: [*]C(=C1S[*][*]C1=[*])[*]
3. Unknown ECFP_2 feature: -661097313: [*]CN1C(=[*])[*][*]C1=[*]
4. Unknown ECFP_2 feature: -37698365: [*]N([*])CC(=[*])[*]

Feature Contribution

Top features for positive contribution			
Fingerprint	Bit/Smiles	Feature Structure	Score
ECFP_6	1559650422	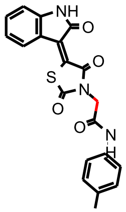 [*]C[*]	0.203

ECFP_6	734603939	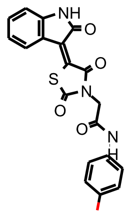 [*]C	0.0424
ECFP_6	-1897341097	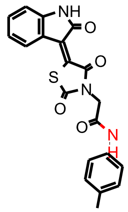 [*]N[*]	0.0284

Top Features for negative contribution

Fingerprint	Bit/Smiles	Feature Structure	Score
ECFP_6	2106656448	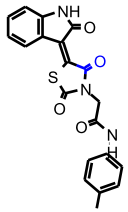 [*]C(=O)[*]	-0.275
ECFP_6	1996767644	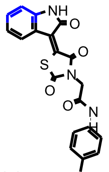 [*][c](:[*]):[cH]:[cH]:[*]	-0.251
ECFP_6	642810091	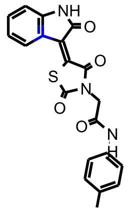 [*][c](:[*]):[*]	-0.247

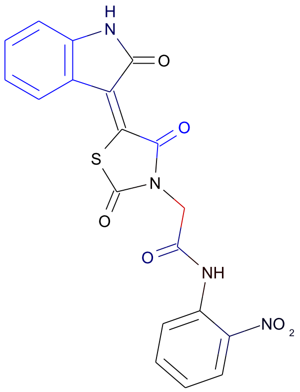

$C_{19}H_{12}N_4O_6S$
Molecular Weight: 424.38678
ALogP: 1.593
Rotatable Bonds: 4
Acceptors: 7
Donors: 2

Model Prediction

Prediction: 84.8
Unit: mg/kg_body_weight/day
Mahalanobis Distance: 12.9
Mahalanobis Distance p-value: 1.82e-007

Mahalanobis Distance: The Mahalanobis distance (MD) is a generalization of the Euclidean distance that accounts for correlations among the X properties. It is calculated as the distance to the center of the training data. The larger the MD, the less trustworthy the prediction.
Mahalanobis Distance p-value: The p-value gives the fraction of training data with an MD greater than or equal to the one for the given sample, assuming normally distributed data. The smaller the p-value, the less trustworthy the prediciton. For highly non-normal X properties (e.g., fingerprints), the MD p-value is wildly inaccurate.

Structural Similar Compounds

Name	Salicylazosulfapyridine	470	Ochratoxin A
Structure	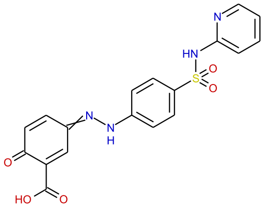	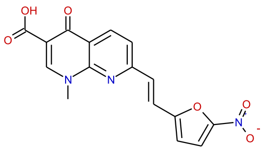	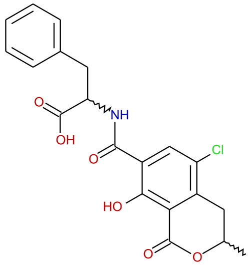
Actual Endpoint (-log C)	2.5034	4.62839	4.79932
Predicted Endpoint (-log C)	3.54214	3.93264	3.6353
Distance	0.686	0.699	0.770
Reference	CPDB	CPDB	CPDB

Model Applicability

Unknown features are fingerprint features in the query molecule, but not found or appearing too infrequently in the training set.

1. All properties and OPS components are within expected ranges.
2. Unknown ECFP_2 feature: -631778390: [*]C(=C1S[*][*]C1=[*])[*]
3. Unknown ECFP_2 feature: -661097313: [*]CN1C(=[*])[*][*]C1=[*]
4. Unknown ECFP_2 feature: -37698365: [*]N([*])CC(=[*])[*]

Feature Contribution

Top features for positive contribution

Fingerprint	Bit/Smiles	Feature Structure	Score
ECFP_6	1559650422	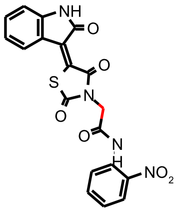 [*]C[*]	0.203

ECFP_6	781519895	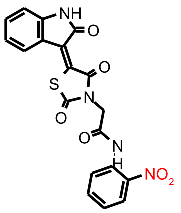 [*][O-]	0.0797
ECFP_6	1043790491	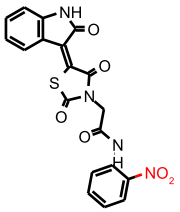 [*][N+](=[*])[*]	0.046
**Top Features for negative contribution**			
**Fingerprint**	**Bit/Smiles**	**Feature Structure**	**Score**
ECFP_6	2106656448	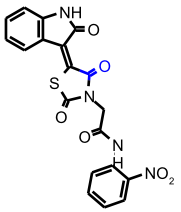 [*]C(=O)[*]	-0.275
ECFP_6	1996767644	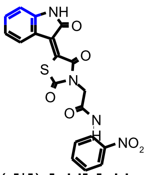 [*][c](:[*]):[cH]:[cH]:[*]	-0.251
ECFP_6	642810091	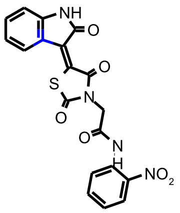 [*][c](:[*]):[*]	-0.247

Sorafenib

TOPKAT_Carcinogenic_Potency_TD50_Mouse

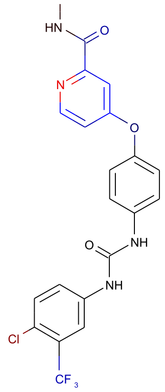

$C_{21}H_{16}ClF_3N_4O_3$

Molecular Weight: 464.82494

ALogP: 4.175

Rotatable Bonds: 6

Acceptors: 4

Donors: 3

Structural Similar Compounds

Name	Ochratoxin A	542	4-Chloro-6-(2,3-xylidino)-2-pyri-mi-dinylthio(N-b-hydroxy-ethyl) acetamide
Structure	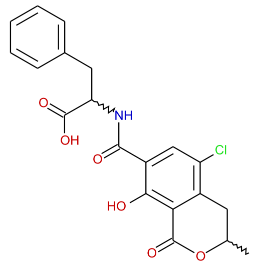	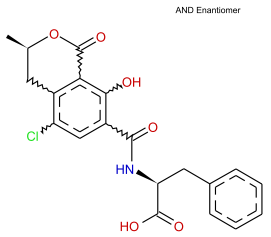	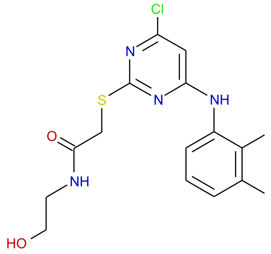
Actual Endpoint (-log C)	4.79932	4.79932	3.91517
Predicted Endpoint (-log C)	3.6353	3.6353	3.92186
Distance	0.718	0.718	0.738
Reference	CPDB	CPDB	CPDB

Model Prediction

Prediction: 19.2

Unit: mg/kg_body_weight/day

Mahalanobis Distance: 12.4

Mahalanobis Distance p-value: 2.94e-006

Mahalanobis Distance: The Mahalanobis distance (MD) is a generalization of the Euclidean distance that accounts for correlations among the X properties. It is calculated as the distance to the center of the training data. The larger the MD, the less trustworthy the prediction.
Mahalanobis Distance p-value: The p-value gives the fraction of training data with an MD greater than or equal to the one for the given sample, assuming normally distributed data. The smaller the p-value, the less trustworthy the prediciton. For highly non-normal X properties (e.g., fingerprints), the MD p-value is wildly inaccurate.

Model Applicability

Unknown features are fingerprint features in the query molecule, but not found or appearing too infrequently in the training set.

1. All properties and OPS components are within expected ranges.
2. Unknown ECFP_2 feature: 1413420509: [*]C(=[*])[c](:[cH]:[*]):n:[*]
3. Unknown ECFP_2 feature: 1338334141: [*]C(=[*])NC

Feature Contribution

Top features for positive contribution			
**Fingerprint**	**Bit/Smiles**	**Feature Structure**	**Score**
ECFP_6	655739385	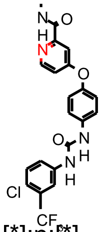 [*]:n:[*]	0.229

ECFP_6	-817402818	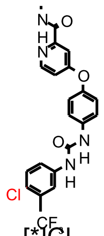 [*]Cl	0.129
ECFP_6	-176455838	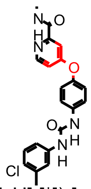 [*]O[c](:[cH]:[*]):[cH]:[*]	0.0818
**Top Features for negative contribution**			
**Fingerprint**	**Bit/Smiles**	**Feature Structure**	**Score**
ECFP_6	1996767644	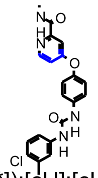 [*][c](:[*]):[cH]:[cH]:[*]	-0.251
ECFP_6	642810091	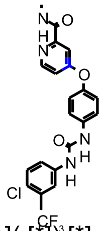 [*][c](:[*]):[*]	-0.247
ECFP_6	-182236392	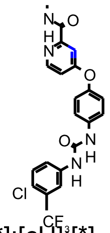 [*]:[cH]:[*]	-0.232

Sunitinib

TOPKAT_Carcinogenic_Potency_TD50_Mouse

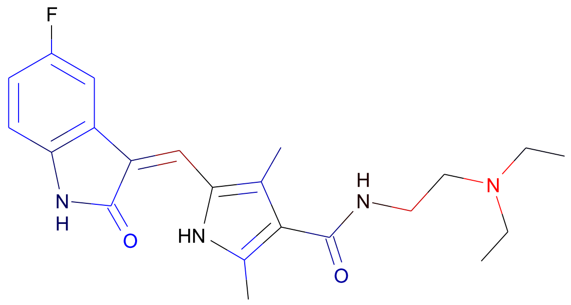

$C_{22}H_{27}FN_4O_2$

Molecular Weight: 398.47378

ALogP: 2.997

Rotatable Bonds: 7

Acceptors: 3

Donors: 3

Model Prediction

Prediction: 33.3

Unit: mg/kg_body_weight/day

Mahalanobis Distance: 13.2

Mahalanobis Distance p-value: 5.11e-008

Mahalanobis Distance: The Mahalanobis distance (MD) is a generalization of the Euclidean distance that accounts for correlations among the X properties. It is calculated as the distance to the center of the training data. The larger the MD, the less trustworthy the prediction.
Mahalanobis Distance p-value: The p-value gives the fraction of training data with an MD greater than or equal to the one for the given sample, assuming normally distributed data. The smaller the p-value, the less trustworthy the prediciton. For highly non-normal X properties (e.g., fingerprints), the MD p-value is wildly inaccurate.

Structural Similar Compounds

Name	455	4-Chloro-6-(2,3-xylidino)-2-pyri-mi-dinylthio(N-b-hydroxy-ethyl) acetamide	542
Structure	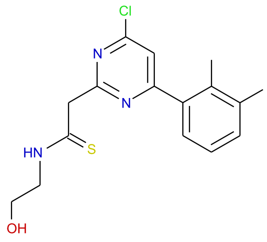	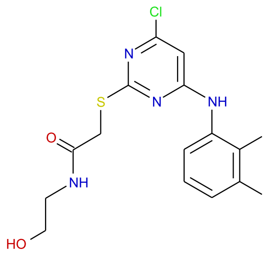	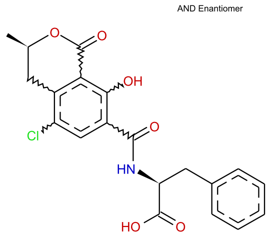
Actual Endpoint (-log C)	3.87681	3.91517	4.79932
Predicted Endpoint (-log C)	3.77582	3.92186	3.6353
Distance	0.676	0.729	0.751
Reference	CPDB	CPDB	CPDB

Model Applicability

Unknown features are fingerprint features in the query molecule, but not found or appearing too infrequently in the training set.

1. All properties and OPS components are within expected ranges.
2. Unknown ECFP_2 feature: 1791989338: [*][c]1:[*]:[*]:[nH]:[c]:1C
3. Unknown ECFP_2 feature: -1658273810: [*]C(=[*])[c]1:[c]([*]):[*]:[*]:[c]:1[*]
4. Unknown ECFP_2 feature: 980271847: [*][c]1:[*]:[*]:[nH]:[c]:1C=[*]
5. Unknown ECFP_2 feature: 1182722866: [*]C(=CC(=[*])[*])[*]
6. Unknown ECFP_2 feature: 1718013682: [*]\C=C\1/C(=[*])[*][*]:[c]1:[*]

Feature Contribution

Top features for positive contribution

Fingerprint	Bit/Smiles	Feature Structure	Score

ECFP_6	-1072294614	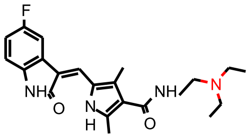 [*]N([*])[*]	0.428
ECFP_6	1559650422	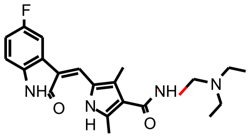 [*]C[*]	0.203
ECFP_6	-1925046727	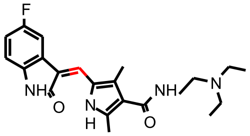 [*]C=[*]	0.145

Top Features for negative contribution

Fingerprint	Bit/Smiles	Feature Structure	Score
ECFP_6	2106656448	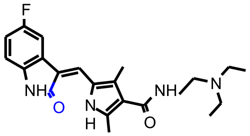 [*]C(=O)[*]	-0.275
ECFP_6	1996767644	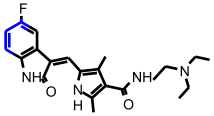 [*][c](:[*]):[cH]:[cH]:[*]	-0.251

ECFP_6	642810091	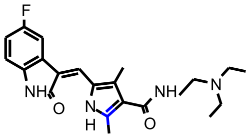 [*][c](:[*]):[*]	-0.247

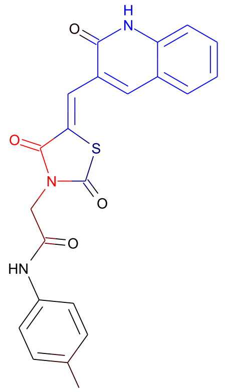

$C_{22}H_{17}N_3O_4S$
Molecular Weight: 419.45307
ALogP: 2.653
Rotatable Bonds: 4
Acceptors: 5
Donors: 2

Model Prediction

Prediction: 122
Unit: mg/kg_body_weight/day
Mahalanobis Distance: 12
Mahalanobis Distance p-value: 0.000607

Mahalanobis Distance: The Mahalanobis distance (MD) is a generalization of the Euclidean distance that accounts for correlations among the X properties. It is calculated as the distance to the center of the training data. The larger the MD, the less trustworthy the prediction.
Mahalanobis Distance p-value: The p-value gives the fraction of training data with an MD greater than or equal to the one for the given sample, assuming normally distributed data. The smaller the p-value, the less trustworthy the prediciton. For highly non-normal X properties (e.g., fingerprints), the MD p-value is wildly inaccurate.

Structural Similar Compounds

Name	Ochratoxin A	542	4,4´-Sulfonylbisacetanilide
Structure	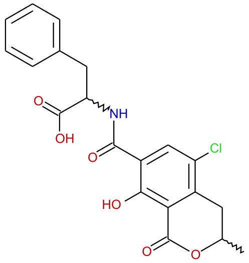	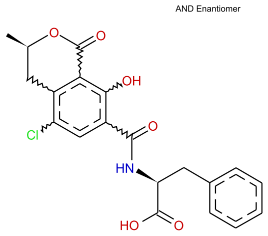	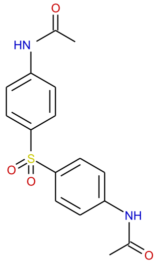
Actual Endpoint (-log C)	6.47264	6.59334	3.77655
Predicted Endpoint (-log C)	5.06501	5.06501	3.55337
Distance	0.629	0.629	0.663
Reference	CPDB	CPDB	CPDB

Model Applicability

Unknown features are fingerprint features in the query molecule, but not found or appearing too infrequently in the training set.

1. All properties and OPS components are within expected ranges.

Feature Contribution

Top features for positive contribution			
**Fingerprint**	**Bit/Smiles**	**Feature Structure**	**Score**
FCFP_6	565998553	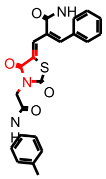 [*]N1[*][*]C(=[*])C1=O	0.357

FCFP_6	1	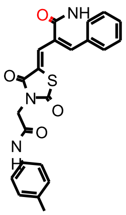 [*]=O	0.234
FCFP_6	203677720	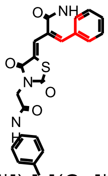 [*][c](:[*]):[c](C=[*]):[cH]:[*]	0.137

Top Features for negative contribution

Fingerprint	Bit/Smiles	Feature Structure	Score
FCFP_6	451847724	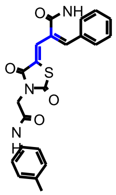 [*]C(=CC(=[*])[*])[*]	-0.436
FCFP_6	991735244	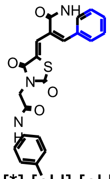 [*][c]1:[*]:[cH]:[cH] :[cH]:[cH]:1	-0.422
FCFP_6	436886043	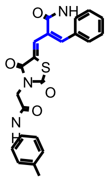 [*]\C=C(\C=[*])/C(=[*])[*]	-0.383

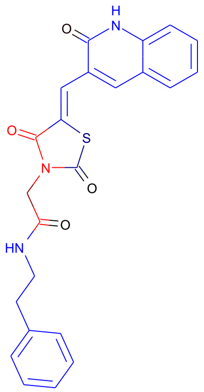

$C_{23}H_{19}N_{3}O_{4}S$

Molecular Weight: 433.47965

ALogP: 2.495

Rotatable Bonds: 6

Acceptors: 5

Donors: 2

Model Prediction

Prediction: 813

Unit: mg/kg_body_weight/day

Mahalanobis Distance: 14.3

Mahalanobis Distance p-value: 3.18e-009

Mahalanobis Distance: The Mahalanobis distance (MD) is a generalization of the Euclidean distance that accounts for correlations among the X properties. It is calculated as the distance to the center of the training data. The larger the MD, the less trustworthy the prediction.
Mahalanobis Distance p-value: The p-value gives the fraction of training data with an MD greater than or equal to the one for the given sample, assuming normally distributed data. The smaller the p-value, the less trustworthy the prediciton. For highly non-normal X properties (e.g., fingerprints), the MD p-value is wildly inaccurate.

Structural Similar Compounds

Name	Ochratoxin A	542	Salicylazosulfapyridine
Structure	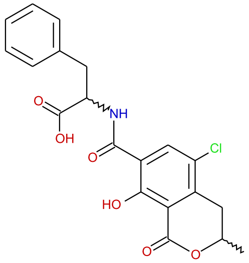	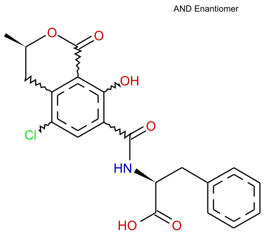	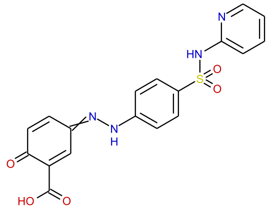
Actual Endpoint (-log C)	6.47264	6.59334	2.39891
Predicted Endpoint (-log C)	5.06501	5.06501	3.17598
Distance	0.614	0.614	0.704
Reference	CPDB	CPDB	CPDB

Model Applicability

Unknown features are fingerprint features in the query molecule, but not found or appearing too infrequently in the training set.

1. All properties and OPS components are within expected ranges.

Feature Contribution

Top features for positive contribution

Fingerprint	Bit/Smiles	Feature Structure	Score
FCFP_6	565998553	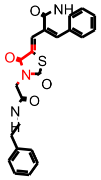 [*]N1[*][*]C(=[*])C1=O	0.357

FCFP_6	1	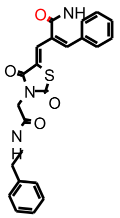 [*]=O	0.234
FCFP_6	-885550502	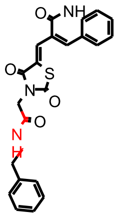 [*]CNC(=[*])[*]	0.229

Top Features for negative contribution

Fingerprint	Bit/Smiles	Feature Structure	Score
FCFP_6	-1272709286	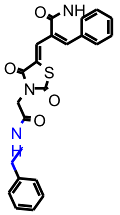 [*]CCN[*]	-0.526
FCFP_6	451847724	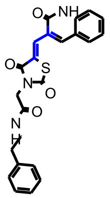 [*]C(=CC(=[*])[*])[*]	-0.436
FCFP_6	991735244	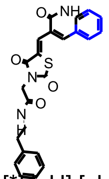 [*][c]1:[*]:[cH]:[cH] :[cH]:[cH]:1	-0.422

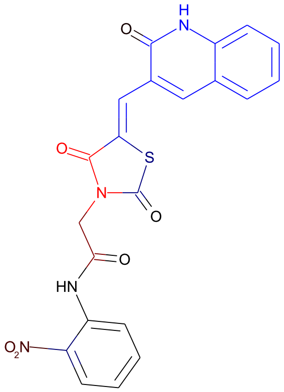

$C_{21}H_{14}N_4O_6S$
Molecular Weight: 450.42405
ALogP: 2.061
Rotatable Bonds: 5
Acceptors: 7
Donors: 2

Model Prediction

Prediction: 75.5
Unit: mg/kg_body_weight/day
Mahalanobis Distance: 13
Mahalanobis Distance p-value: 6.02e-006

Mahalanobis Distance: The Mahalanobis distance (MD) is a generalization of the Euclidean distance that accounts for correlations among the X properties. It is calculated as the distance to the center of the training data. The larger the MD, the less trustworthy the prediction.
Mahalanobis Distance p-value: The p-value gives the fraction of training data with an MD greater than or equal to the one for the given sample, assuming normally distributed data. The smaller the p-value, the less trustworthy the prediciton. For highly non-normal X properties (e.g., fingerprints), the MD p-value is wildly inaccurate.

Structural Similar Compounds

Name	623	Salicylazosulfapyridine	4-Bis(2-hydroxyethyl)amino-2-(5-nitro-2-thienyl)quinazoline
Structure	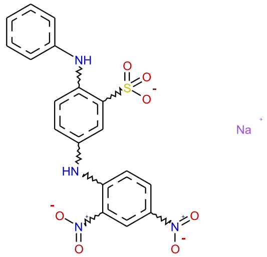	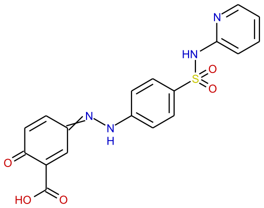	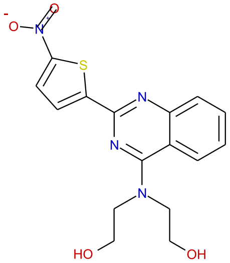
Actual Endpoint (-log C)	2.39985	2.39891	5.05984
Predicted Endpoint (-log C)	3.4177	3.17598	4.23808
Distance	0.633	0.643	0.708
Reference	CPDB	CPDB	CPDB

Model Applicability

Unknown features are fingerprint features in the query molecule, but not found or appearing too infrequently in the training set.

1. All properties and OPS components are within expected ranges.

Feature Contribution

Top features for positive contribution			
**Fingerprint**	**Bit/Smiles**	**Feature Structure**	**Score**
FCFP_6	5	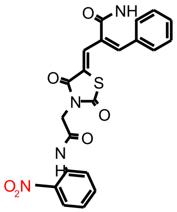 [*][O-]	0.431

FCFP_6	565998553	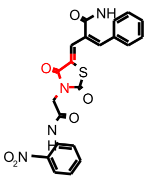<br>[*]N1[*][*]C(=[*])C1=<br>O	0.357
FCFP_6	8	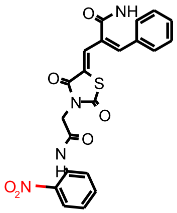<br>[*][N+](=[*])[*]	0.336

Top Features for negative contribution

Fingerprint	Bit/Smiles	Feature Structure	Score
FCFP_6	451847724	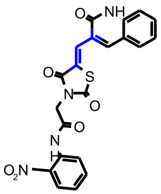<br>[*]C(=CC(=[*])[*])[*]	-0.436
FCFP_6	991735244	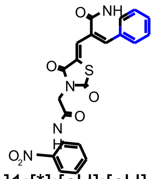<br>[*][c]1:[*]:[cH]:[cH]<br>:[cH]:[cH]:1	-0.422
FCFP_6	436886043	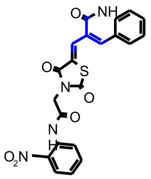<br>[*]\C=C(\C=[*])/C(=[*<br>])[*]	-0.383

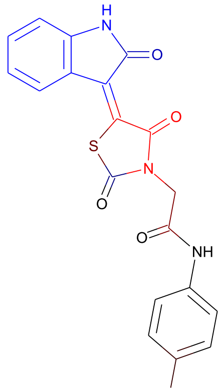

$C_{20}H_{15}N_{3}O_{4}S$
Molecular Weight: 393.4158
ALogP: 2.185
Rotatable Bonds: 3
Acceptors: 5
Donors: 2

Structural Similar Compounds

Name	4,4´-Sulfonylbisacetanilide	1,2-Dihydro-2-(5-nitro-2-thi-enyl) quinazolin-4(3H)-one	542
Structure	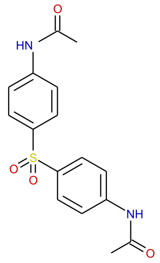	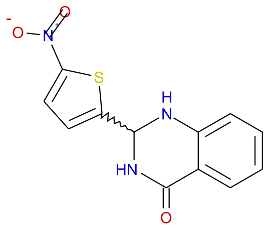	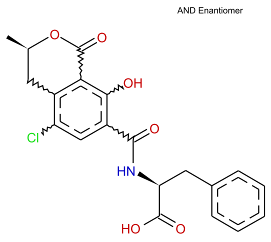
Actual Endpoint (-log C)	3.77655	5.25509	6.59334
Predicted Endpoint (-log C)	3.55337	3.89291	5.06501
Distance	0.629	0.637	0.647
Reference	CPDB	CPDB	CPDB

Model Prediction

Prediction: 18.3
Unit: mg/kg_body_weight/day
Mahalanobis Distance: 11.1
Mahalanobis Distance p-value: 0.0145

Mahalanobis Distance: The Mahalanobis distance (MD) is a generalization of the Euclidean distance that accounts for correlations among the X properties. It is calculated as the distance to the center of the training data. The larger the MD, the less trustworthy the prediction.
Mahalanobis Distance p-value: The p-value gives the fraction of training data with an MD greater than or equal to the one for the given sample, assuming normally distributed data. The smaller the p-value, the less trustworthy the prediciton. For highly non-normal X properties (e.g., fingerprints), the MD p-value is wildly inaccurate.

Model Applicability

Unknown features are fingerprint features in the query molecule, but not found or appearing too infrequently in the training set.

1. All properties and OPS components are within expected ranges.

Feature Contribution

Top features for positive contribution

Fingerprint	Bit/Smiles	Feature Structure	Score
FCFP_6	565998553	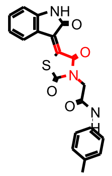 [*]N1[*][*]C(=[*])C1=O	0.357

FCFP_6	1	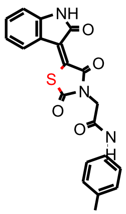<br>[*]=O	0.234
FCFP_6	203677720	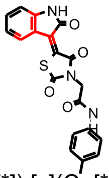<br>[*][c](:[*]):[c](C=[*]):[cH]:[*]	0.137
**Top Features for negative contribution**			
**Fingerprint**	**Bit/Smiles**	**Feature Structure**	**Score**
FCFP_6	991735244	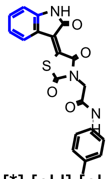<br>[*][c]1:[*]:[cH]:[cH] :[cH]:[cH]:1	-0.422
FCFP_6	16	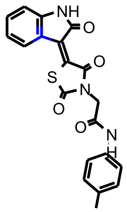<br>[*]:[cH]:[*]	-0.354
FCFP_6	590925877	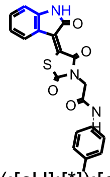<br>[*]N[c](:[cH]:[*]):[c]([*]):[*]	-0.323

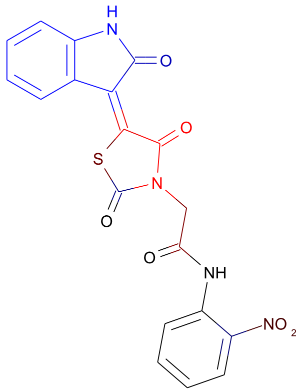

$C_{19}H_{12}N_4O_6S$
Molecular Weight: 424.38678
ALogP: 1.593
Rotatable Bonds: 4
Acceptors: 7
Donors: 2

Model Prediction

Prediction: 11.3
Unit: mg/kg_body_weight/day
Mahalanobis Distance: 12.3
Mahalanobis Distance p-value: 0.000148

Mahalanobis Distance: The Mahalanobis distance (MD) is a generalization of the Euclidean distance that accounts for correlations among the X properties. It is calculated as the distance to the center of the training data. The larger the MD, the less trustworthy the prediction.
Mahalanobis Distance p-value: The p-value gives the fraction of training data with an MD greater than or equal to the one for the given sample, assuming normally distributed data. The smaller the p-value, the less trustworthy the prediciton. For highly non-normal X properties (e.g., fingerprints), the MD p-value is wildly inaccurate.

Structural Similar Compounds

Name	623	Salicylazosulfapyridine	418
Structure	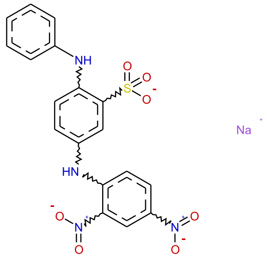	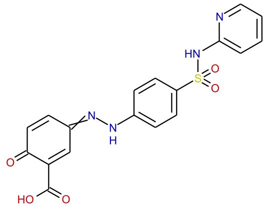	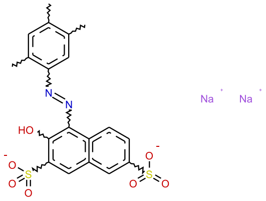
Actual Endpoint (-log C)	2.39985	2.39891	2.9349
Predicted Endpoint (-log C)	3.4177	3.17598	3.45907
Distance	0.642	0.653	0.708
Reference	CPDB	CPDB	CPDB

Model Applicability

Unknown features are fingerprint features in the query molecule, but not found or appearing too infrequently in the training set.

1. All properties and OPS components are within expected ranges.

Feature Contribution

Top features for positive contribution

Fingerprint	Bit/Smiles	Feature Structure	Score
FCFP_6	5	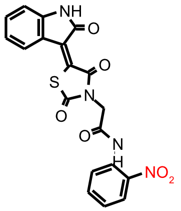 [*][O-]	0.431

FCFP_6	565998553	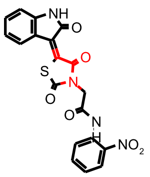 [*]N1[*][*]C(=[*])C1= O	0.357
FCFP_6	8	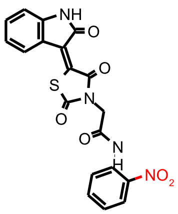 [*][N+](=[*])[*]	0.336
**Top Features for negative contribution**			
**Fingerprint**	**Bit/Smiles**	**Feature Structure**	**Score**
FCFP_6	991735244	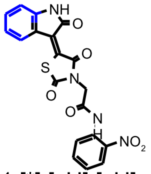 [*][c]1:[*]:[cH]:[cH] :[cH]:[cH]:1	-0.422
FCFP_6	16	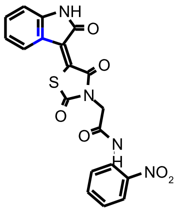 [*]:[cH]:[*]	-0.354
FCFP_6	590925877	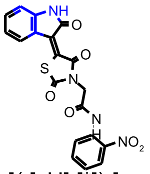 [*]N[c](:[cH]:[*]):[c]([*]):[*]	-0.323

Sorafenib

TOPKAT_Carcinogenic_Potency_TD50_Rat

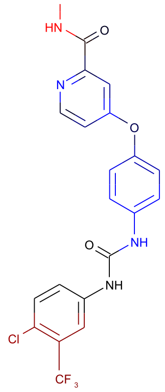

$C_{21}H_{16}ClF_3N_4O_3$

Molecular Weight: 464.82494

ALogP: 4.175

Rotatable Bonds: 6

Acceptors: 4

Donors: 3

Model Prediction

Prediction: 14.2

Unit: mg/kg_body_weight/day

Mahalanobis Distance: 20.4

Mahalanobis Distance p-value: 9.56e-031

Mahalanobis Distance: The Mahalanobis distance (MD) is a generalization of the Euclidean distance that accounts for correlations among the X properties. It is calculated as the distance to the center of the training data. The larger the MD, the less trustworthy the prediction.
Mahalanobis Distance p-value: The p-value gives the fraction of training data with an MD greater than or equal to the one for the given sample, assuming normally distributed data. The smaller the p-value, the less trustworthy the prediciton. For highly non-normal X properties (e.g., fingerprints), the MD p-value is wildly inaccurate.

Structural Similar Compounds

Name	Fluvastatin	913	Ochratoxin A
Structure	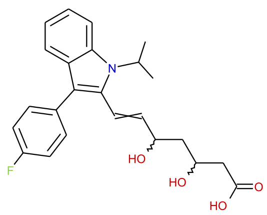	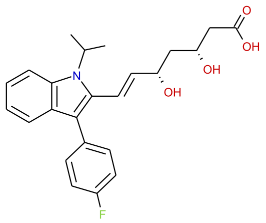	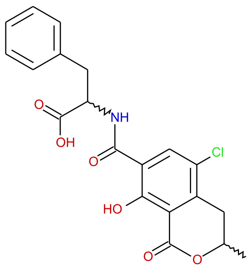
Actual Endpoint (-log C)	3.51742	3.51742	6.47264
Predicted Endpoint (-log C)	5.41573	5.41573	5.06501
Distance	0.597	0.597	0.666
Reference	CPDB	CPDB	CPDB

Model Applicability

Unknown features are fingerprint features in the query molecule, but not found or appearing too infrequently in the training set.

1. All properties and OPS components are within expected ranges.
2. Unknown FCFP_2 feature: -1029533685: [*]:[c](:[*])C(F)(F)F

Feature Contribution

Top features for positive contribution

Fingerprint	Bit/Smiles	Feature Structure	Score
FCFP_6	1	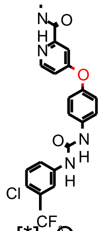 [*]=O	0.234

FCFP_6	-885550502	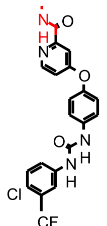 [*]CNC(=[*])[*]	0.229
FCFP_6	32	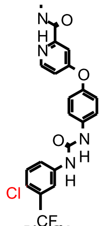 [*]Cl	0.154

Top Features for negative contribution

Fingerprint	Bit/Smiles	Feature Structure	Score
FCFP_6	16	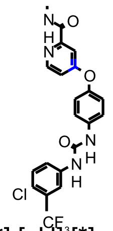 [*]:[cH]:[*]	-0.354
FCFP_6	590925877	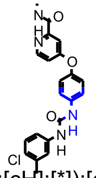 [*]N[c](:[cH]:[*]):[c]([*]):[*]	-0.323
FCFP_6	1674451008	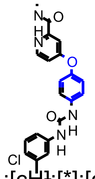 [*]O[c]1:[cH]:[*]:[c]([*]):[cH]:[cH]:1	-0.233

Sunitinib

TOPKAT_Carcinogenic_Potency_TD50_Rat

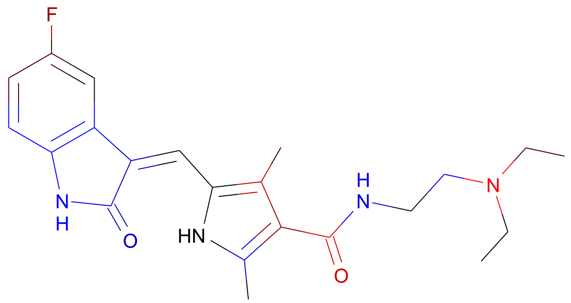

$C_{22}H_{27}FN_4O_2$
Molecular Weight: 398.47378
ALogP: 2.997
Rotatable Bonds: 7
Acceptors: 3
Donors: 3

Model Prediction

Prediction: 4.13
Unit: mg/kg_body_weight/day
Mahalanobis Distance: 14
Mahalanobis Distance p-value: 2.69e-008

Mahalanobis Distance: The Mahalanobis distance (MD) is a generalization of the Euclidean distance that accounts for correlations among the X properties. It is calculated as the distance to the center of the training data. The larger the MD, the less trustworthy the prediction.
Mahalanobis Distance p-value: The p-value gives the fraction of training data with an MD greater than or equal to the one for the given sample, assuming normally distributed data. The smaller the p-value, the less trustworthy the prediciton. For highly non-normal X properties (e.g., fingerprints), the MD p-value is wildly inaccurate.

Structural Similar Compounds

Name	913	Fluvastatin	542
Structure	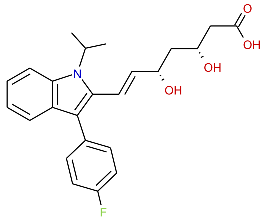	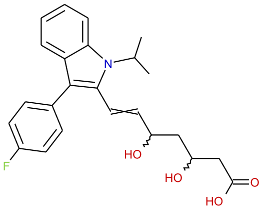	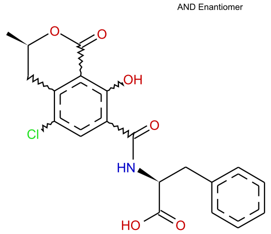
Actual Endpoint (-log C)	3.51742	3.51742	6.59334
Predicted Endpoint (-log C)	5.41573	5.41573	5.06501
Distance	0.636	0.636	0.671
Reference	CPDB	CPDB	CPDB

Model Applicability

Unknown features are fingerprint features in the query molecule, but not found or appearing too infrequently in the training set.

1. OPS PC18 out of range. Value: 6.1602. Training min, max, SD, explained variance: -4.1023, 4.8669, 1.305, 0.0164.

Feature Contribution

Top features for positive contribution

Fingerprint	Bit/Smiles	Feature Structure	Score
FCFP_6	9	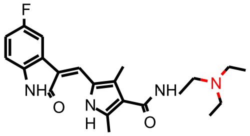 [*]N([*])[*]	0.385

FCFP_6	-587569116	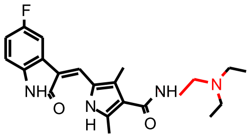<br>[*]CCN([*])[*]	0.319
FCFP_6	1	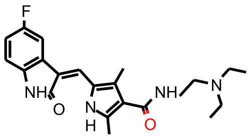<br>[*]=O	0.234

Top Features for negative contribution

Fingerprint	Bit/Smiles	Feature Structure	Score
FCFP_6	-1272709286	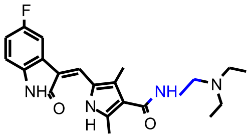<br>[*]CCN[*]	-0.526
FCFP_6	16	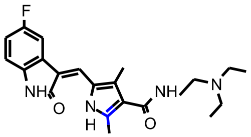<br>[*]:[cH]:[*]	-0.354
FCFP_6	590925877	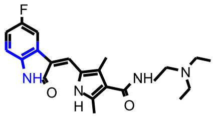<br>[*]N[c](:[cH]:[*]):[c]([*]):[*]	-0.323

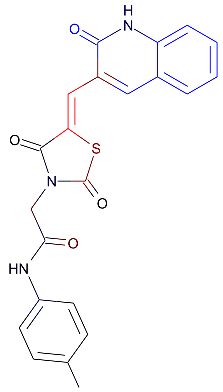

$C_{22}H_{17}N_3O_4S$
Molecular Weight: 419.45307
ALogP: 2.653
Rotatable Bonds: 4
Acceptors: 5
Donors: 2

Model Prediction

Prediction: 0.0195
Unit: g/kg_body_weight
Mahalanobis Distance: 30.1
Mahalanobis Distance p-value: 7.46e-025

Mahalanobis Distance: The Mahalanobis distance (MD) is a generalization of the Euclidean distance that accounts for correlations among the X properties. It is calculated as the distance to the center of the training data. The larger the MD, the less trustworthy the prediction.
Mahalanobis Distance p-value: The p-value gives the fraction of training data with an MD greater than or equal to the one for the given sample, assuming normally distributed data. The smaller the p-value, the less trustworthy the prediciton. For highly non-normal X properties (e.g., fingerprints), the MD p-value is wildly inaccurate.

Structural Similar Compounds

Name	CHLORSULFURON	DANTROLENE.NA	GLIPIZIDE
Structure	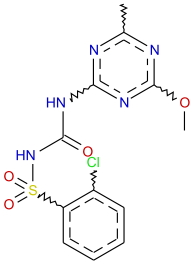	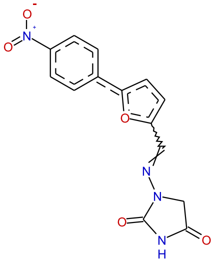	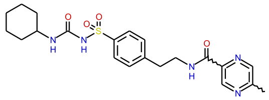
Actual Endpoint (-log C)	4.15566	4.19625	3.94991
Predicted Endpoint (-log C)	3.79771	4.62637	3.95594
Distance	0.625	0.628	0.638
Reference	EPA COVER SHEET 0027;880301;(1)	NDA-17443	NDA-17583

Model Applicability

Unknown features are fingerprint features in the query molecule, but not found or appearing too infrequently in the training set.

1. OPS PC22 out of range. Value: -6.3866. Training min, max, SD, explained variance: -4.3287, 5.3383, 1.588, 0.0110.
2. Unknown ECFP_6 feature: -154530762: [*]N[*]
3. Unknown ECFP_6 feature: 912478223: [*]S[*]
4. Unknown ECFP_6 feature: 1997021792: [*]:[cH]:[cH]:[cH]:[*]
5. Unknown ECFP_6 feature: 1335833675: [*]N[c](:[cH]:[*]):[c]([*]):[*]
6. Unknown ECFP_6 feature: 1336666212: [*][c](:[*]):[c](C=[*]):[cH]:[*]
7. Unknown ECFP_6 feature: -1699286547: [*]C(=[*])N[c](:[*]):[*]
8. Unknown ECFP_6 feature: 1298725959: [*]NC(=O)C(=[*])[*]
9. Unknown ECFP_6 feature: 2131425032: [*]\C=C(\C=[*])/C(=[*])[*]
10. Unknown ECFP_6 feature: 464808839: [*]C(=C[c](:[*]):[*])[*]
11. Unknown ECFP_6 feature: 1182722866: [*]C(=CC(=[*])[*])[*]
12. Unknown ECFP_6 feature: 1000552169: [*]\C=C\1/S[*][*]C1=[*]
13. Unknown ECFP_6 feature: 1945129186: [*]N1[*][*]C(=[*])C1=O
14. Unknown ECFP_6 feature: -661097313: [*]CN1C(=[*])[*][*]C1=[*]
15. Unknown ECFP_6 feature: 190445529: [*]N1[*][*]SC1=O
16. Unknown ECFP_6 feature: 2122741631: [*]=C1[*][*]C(=[*])S1
17. Unknown ECFP_6 feature: -37698365: [*]N([*])CC(=[*])[*]
18. Unknown ECFP_6 feature: 1731843802: [*]CC(=O)N[*]

19. Unknown ECFP_6 feature: -177077903: [*]N[c](:[cH]:[*]):[cH]:[*]
20. Unknown ECFP_6 feature: -179515162: [*]:[cH]:[c](C):[cH]:[*]

Feature Contribution

Top features for positive contribution

Fingerprint	Bit/Smiles	Feature Structure	Score
FCFP_6	451847724	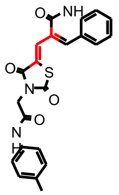 [*]C(=CC(=[*])[*])[*]	0.16
FCFP_6	-1143715940	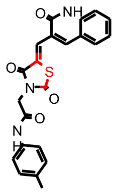 [*]=C1[*][*]C(=[*])S1	0.13
ECFP_6	1559650422	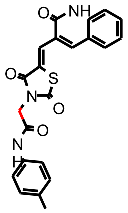 [*]C[*]	0.129

Top Features for negative contribution

Fingerprint	Bit/Smiles	Feature Structure	Score
FCFP_6	991735244	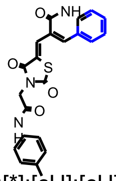 [*][c]1:[*]:[cH]:[cH]:[cH]:[cH]:1	-0.134

ECFP_6	1564392544	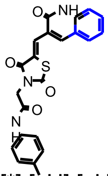 [*][c]1:[*]:[cH]:[cH]:[cH]:[cH]:1	-0.133
ECFP_6	2106656448	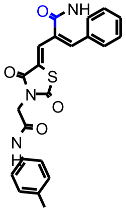 [*]C(=O)[*]	-0.11

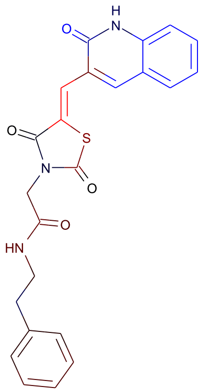

$C_{23}H_{19}N_3O_4S$
Molecular Weight: 433.47965
ALogP: 2.495
Rotatable Bonds: 6
Acceptors: 5
Donors: 2

Model Prediction

Prediction: 0.0298
Unit: g/kg_body_weight
Mahalanobis Distance: 31.7
Mahalanobis Distance p-value: 1.08e-027

Mahalanobis Distance: The Mahalanobis distance (MD) is a generalization of the Euclidean distance that accounts for correlations among the X properties. It is calculated as the distance to the center of the training data. The larger the MD, the less trustworthy the prediction.
Mahalanobis Distance p-value: The p-value gives the fraction of training data with an MD greater than or equal to the one for the given sample, assuming normally distributed data. The smaller the p-value, the less trustworthy the prediciton. For highly non-normal X properties (e.g., fingerprints), the MD p-value is wildly inaccurate.

Structural Similar Compounds

Name	GLIPIZIDE	GLYBURIDE	DANTROLENE.NA
Structure	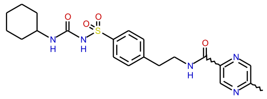	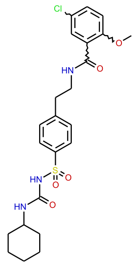	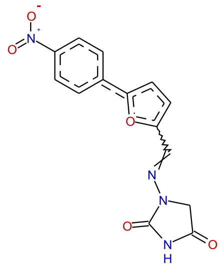
Actual Endpoint (-log C)	3.94991	4.21661	4.19625
Predicted Endpoint (-log C)	3.95594	4.21035	4.62637
Distance	0.561	0.617	0.669
Reference	NDA-17583	UPJ-26452	NDA-17443

Model Applicability

Unknown features are fingerprint features in the query molecule, but not found or appearing too infrequently in the training set.

1. OPS PC22 out of range. Value: -6.8468. Training min, max, SD, explained variance: -4.3287, 5.3383, 1.588, 0.0110.
2. Unknown ECFP_6 feature: -154530762: [*]N[*]
3. Unknown ECFP_6 feature: 912478223: [*]S[*]
4. Unknown ECFP_6 feature: 1997021792: [*]:[cH]:[cH]:[cH]:[*]
5. Unknown ECFP_6 feature: 1335833675: [*]N[c](:[cH]:[*]):[c]([*]):[*]
6. Unknown ECFP_6 feature: 1336666212: [*][c](:[*]):[c](C=[*]):[cH]:[*]
7. Unknown ECFP_6 feature: -1699286547: [*]C(=[*])N[c](:[*]):[*]
8. Unknown ECFP_6 feature: 1298725959: [*]NC(=O)C(=[*])[*]
9. Unknown ECFP_6 feature: 2131425032: [*]\C=C(\C=[*])/C(=[*])[*]
10. Unknown ECFP_6 feature: 464808839: [*]C(=C[c](:[*]):[*])[*]
11. Unknown ECFP_6 feature: 1182722866: [*]C(=CC(=[*])[*])[*]
12. Unknown ECFP_6 feature: 1000552169: [*]\C=C\1/S[*][*]C1=[*]
13. Unknown ECFP_6 feature: 1945129186: [*]N1[*][*]C(=[*])C1=O
14. Unknown ECFP_6 feature: -661097313: [*]CN1C(=[*])[*][*]C1=[*]
15. Unknown ECFP_6 feature: 190445529: [*]N1[*][*]SC1=O
16. Unknown ECFP_6 feature: 2122741631: [*]=C1[*][*]C(=[*])S1
17. Unknown ECFP_6 feature: -37698365: [*]N([*])CC(=[*])[*]
18. Unknown ECFP_6 feature: 1731843802: [*]CC(=O)N[*]
19. Unknown ECFP_6 feature: 497523368: [*]CNC(=[*])[*]

20. Unknown ECFP_6 feature: -1791034651: [*]CCN[*]
21. Unknown ECFP_6 feature: -1795525632: [*]CC[c](:[*]):[*]

Feature Contribution

Top features for positive contribution

Fingerprint	Bit/Smiles	Feature Structure	Score
FCFP_6	451847724	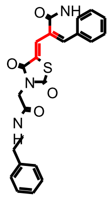<br>[*]C(=CC(=[*])[*])[*]	0.16
FCFP_6	-1143715940	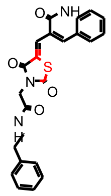<br>[*]=C1[*][*]C(=[*])S1	0.13
ECFP_6	1559650422	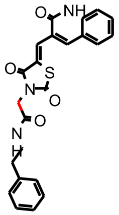<br>[*]C[*]	0.129

Top Features for negative contribution

Fingerprint	Bit/Smiles	Feature Structure	Score
FCFP_6	991735244	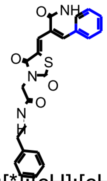<br>[*][c]1:[*]:[cH]:[cH]:[cH]:[cH]:1	-0.134

ECFP_6	1564392544	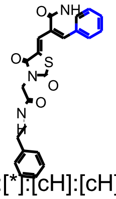 [*][c]1:[*]:[cH]:[cH]:[cH]:[cH]:1	-0.133
ECFP_6	2106656448	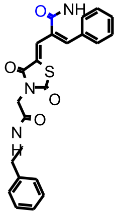 [*]C(=O)[*]	-0.11

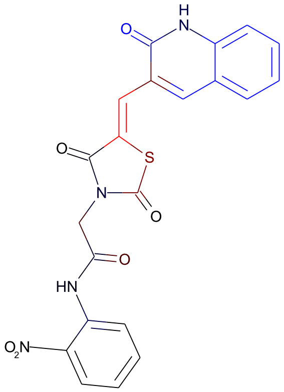

$C_{21}H_{14}N_4O_6S$
Molecular Weight: 450.42405
ALogP: 2.061
Rotatable Bonds: 5
Acceptors: 7
Donors: 2

Model Prediction

Prediction: 0.0142
Unit: g/kg_body_weight
Mahalanobis Distance: 33.6
Mahalanobis Distance p-value: 4.15e-031

Mahalanobis Distance: The Mahalanobis distance (MD) is a generalization of the Euclidean distance that accounts for correlations among the X properties. It is calculated as the distance to the center of the training data. The larger the MD, the less trustworthy the prediction.
Mahalanobis Distance p-value: The p-value gives the fraction of training data with an MD greater than or equal to the one for the given sample, assuming normally distributed data. The smaller the p-value, the less trustworthy the prediciton. For highly non-normal X properties (e.g., fingerprints), the MD p-value is wildly inaccurate.

Structural Similar Compounds

Name	GLIPIZIDE	ALLY	HARMONY
Structure	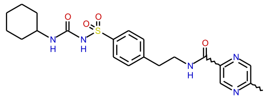	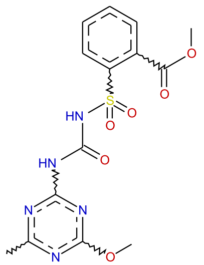	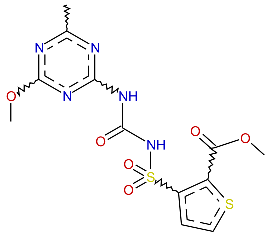
Actual Endpoint (-log C)	3.94991	3.1834	4.1902
Predicted Endpoint (-log C)	3.95594	3.59541	4.09361
Distance	0.623	0.636	0.651
Reference	NDA-17583	EPA COVER SHEET 0288;891101;(1)	EPA COVER SHEET 0337;881201;(1)

Model Applicability

Unknown features are fingerprint features in the query molecule, but not found or appearing too infrequently in the training set.

1. OPS PC22 out of range. Value: -5.8354. Training min, max, SD, explained variance: -4.3287, 5.3383, 1.588, 0.0110.
2. Unknown FCFP_2 feature: 5: [*][O-]
3. Unknown FCFP_2 feature: -828984032: [*][c](:[*]):[c](:[cH]:[*])[N+](=[*])[*]
4. Unknown FCFP_2 feature: -1338588315: [*]:[c](:[*])[N+](=O)[O-]
5. Unknown FCFP_2 feature: 1872392852: [*][N+](=O)[*]
6. Unknown FCFP_2 feature: 260476081: [*][N+](=[*])[O-]
7. Unknown ECFP_6 feature: -154530762: [*]N[*]
8. Unknown ECFP_6 feature: 912478223: [*]S[*]
9. Unknown ECFP_6 feature: 1043790491: [*][N+](=[*])[*]
10. Unknown ECFP_6 feature: 781519895: [*][O-]
11. Unknown ECFP_6 feature: 1997021792: [*]:[cH]:[cH]:[cH]:[*]
12. Unknown ECFP_6 feature: 1335833675: [*]N[c](:[cH]:[*]):[c]([*]):[*]
13. Unknown ECFP_6 feature: 1336666212: [*][c](:[*]):[c](C=[*]):[cH]:[*]
14. Unknown ECFP_6 feature: -1699286547: [*]C(=[*])N[c](:[*]):[*]
15. Unknown ECFP_6 feature: 1298725959: [*]NC(=O)C(=[*])[*]
16. Unknown ECFP_6 feature: 2131425032: [*]\C=C(\C=[*])/C(=[*])[*]
17. Unknown ECFP_6 feature: 464808839: [*]C(=C[c](:[*]):[*])[*]
18. Unknown ECFP_6 feature: 1182722866: [*]C(=CC(=[*])[*])[*]

19. Unknown ECFP_6 feature: 1000552169: [*]\C=C\1/S[*][*]C1=[*]
20. Unknown ECFP_6 feature: 1945129186: [*]N1[*][*]C(=[*])C1=O
21. Unknown ECFP_6 feature: -661097313: [*]CN1C(=[*])[*][*]C1=[*]
22. Unknown ECFP_6 feature: 190445529: [*]N1[*][*]SC1=O
23. Unknown ECFP_6 feature: 2122741631: [*]=C1[*][*]C(=[*])S1
24. Unknown ECFP_6 feature: -37698365: [*]N([*])CC(=[*])[*]
25. Unknown ECFP_6 feature: 1731843802: [*]CC(=O)N[*]
26. Unknown ECFP_6 feature: 1335108269: [*]N[c](:[cH]:[*]):[c]([*]):[*]
27. Unknown ECFP_6 feature: -1956535100: [*][c](:[*]):[c](:[cH]:[*])[N+](=[*])[*]
28. Unknown ECFP_6 feature: -215026467: [*]:[c](:[*])[N+](=O)[O-]
29. Unknown ECFP_6 feature: 2104376220: [*][N+](=O)[*]
30. Unknown ECFP_6 feature: -659271057: [*][N+](=[*])[O-]

Feature Contribution

Top features for positive contribution			
**Fingerprint**	**Bit/Smiles**	**Feature Structure**	**Score**
FCFP_6	451847724	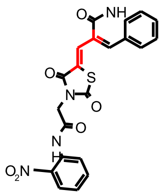 [*]C(=CC(=[*])[*])[*]	0.16
FCFP_6	-1143715940	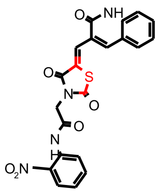 [*]=C1[*][*]C(=[*])S1	0.13
ECFP_6	1559650422	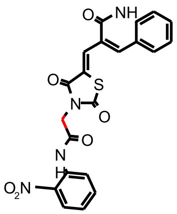 [*]C[*]	0.129

Top Features for negative contribution

Fingerprint	Bit/Smiles	Feature Structure	Score
FCFP_6	991735244	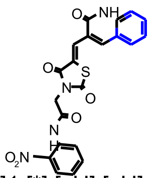<br>[*][c]1:[*]:[cH]:[cH]:[cH]:[cH]:1	-0.134
ECFP_6	1564392544	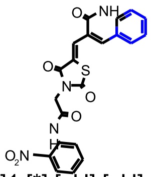<br>[*][c]1:[*]:[cH]:[cH]:[cH]:[cH]:1	-0.133
ECFP_6	2106656448	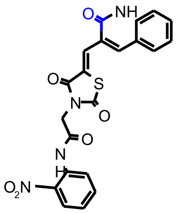<br>[*]C(=O)[*]	-0.11

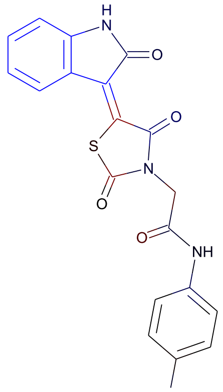

$C_{20}H_{15}N_3O_4S$
Molecular Weight: 393.4158
ALogP: 2.185
Rotatable Bonds: 3
Acceptors: 5
Donors: 2

Model Prediction

Prediction: 0.0456
Unit: g/kg_body_weight
Mahalanobis Distance: 29.6
Mahalanobis Distance p-value: 7.33e-024

Mahalanobis Distance: The Mahalanobis distance (MD) is a generalization of the Euclidean distance that accounts for correlations among the X properties. It is calculated as the distance to the center of the training data. The larger the MD, the less trustworthy the prediction.
Mahalanobis Distance p-value: The p-value gives the fraction of training data with an MD greater than or equal to the one for the given sample, assuming normally distributed data. The smaller the p-value, the less trustworthy the prediciton. For highly non-normal X properties (e.g., fingerprints), the MD p-value is wildly inaccurate.

Structural Similar Compounds

Name	PIROXICAM	DANTROLENE.NA	CHLORSULFURON
Structure	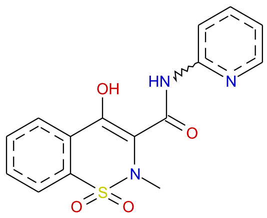	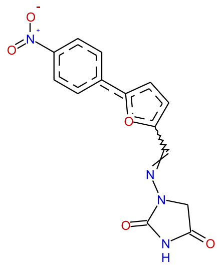	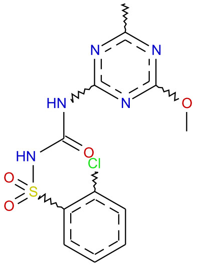
Actual Endpoint (-log C)	5.52028	4.19625	4.15566
Predicted Endpoint (-log C)	4.06087	4.62637	3.79771
Distance	0.565	0.578	0.586
Reference	NDA-18147	NDA-17443	EPA COVER SHEET 0027;880301;(1)

Model Applicability

Unknown features are fingerprint features in the query molecule, but not found or appearing too infrequently in the training set.

1. OPS PC22 out of range. Value: -5.1654. Training min, max, SD, explained variance: -4.3287, 5.3383, 1.588, 0.0110.
2. Unknown ECFP_6 feature: 912478223: [*]S[*]
3. Unknown ECFP_6 feature: -154530762: [*]N[*]
4. Unknown ECFP_6 feature: 1790105651: [*]C(=C1C(=[*])[*][*]:[c]1:[*])[*]
5. Unknown ECFP_6 feature: -631778390: [*]C(=C1S[*][*]C1=[*])[*]
6. Unknown ECFP_6 feature: 1945129186: [*]N1[*][*]C(=[*])C1=O
7. Unknown ECFP_6 feature: 190445529: [*]N1[*][*]SC1=O
8. Unknown ECFP_6 feature: 2122741631: [*]=C1[*][*]C(=[*])S1
9. Unknown ECFP_6 feature: -661097313: [*]CN1C(=[*])[*][*]C1=[*]
10. Unknown ECFP_6 feature: 1298725959: [*]NC(=O)C(=[*])[*]
11. Unknown ECFP_6 feature: -1699286547: [*]C(=[*])N[c](:[*]):[*]
12. Unknown ECFP_6 feature: 1335833675: [*]N[c](:[cH]:[*]):[c]([*]):[*]
13. Unknown ECFP_6 feature: 1997021792: [*]:[cH]:[cH]:[cH]:[*]
14. Unknown ECFP_6 feature: -37698365: [*]N([*])CC(=[*])[*]
15. Unknown ECFP_6 feature: 1731843802: [*]CC(=O)N[*]
16. Unknown ECFP_6 feature: -177077903: [*]N[c](:[cH]:[*]):[cH]:[*]
17. Unknown ECFP_6 feature: -179515162: [*]:[cH]:[c](C):[cH]:[*]

Feature Contribution

Top features for positive contribution

Fingerprint	Bit/Smiles	Feature Structure	Score
FCFP_6	-1143715940	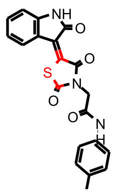 [*]=C1[*][*]C(=[*])S1	0.13
ECFP_6	1559650422	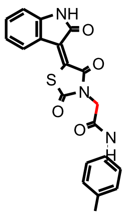 [*]C[*]	0.129
FCFP_6	3	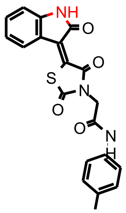 [*]N[*]	0.0924

Top Features for negative contribution

Fingerprint	Bit/Smiles	Feature Structure	Score
FCFP_6	991735244	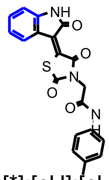 [*][c]1:[*]:[cH]:[cH]:[cH]:[cH]:1	-0.134

ECFP_6	1564392544	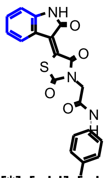 [*][c]1:[*]:[cH]:[cH]:[cH]:[cH]:1	-0.133
ECFP_6	2106656448	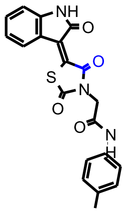 [*]C(=O)[*]	-0.11

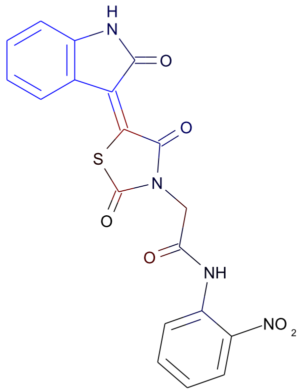

$C_{19}H_{12}N_4O_6S$
Molecular Weight: 424.38678
ALogP: 1.593
Rotatable Bonds: 4
Acceptors: 7
Donors: 2

Model Prediction

Prediction: 0.0386
Unit: g/kg_body_weight
Mahalanobis Distance: 32.8
Mahalanobis Distance p-value: 1.16e-029

Mahalanobis Distance: The Mahalanobis distance (MD) is a generalization of the Euclidean distance that accounts for correlations among the X properties. It is calculated as the distance to the center of the training data. The larger the MD, the less trustworthy the prediction.
Mahalanobis Distance p-value: The p-value gives the fraction of training data with an MD greater than or equal to the one for the given sample, assuming normally distributed data. The smaller the p-value, the less trustworthy the prediciton. For highly non-normal X properties (e.g., fingerprints), the MD p-value is wildly inaccurate.

Structural Similar Compounds

Name	ALLY	HARMONY	CHLORSULFURON
Structure	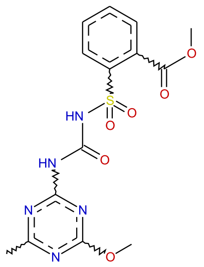	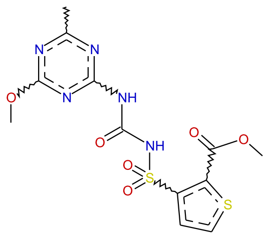	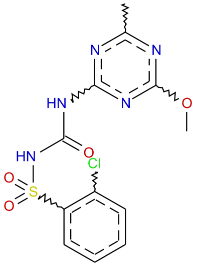
Actual Endpoint (-log C)	3.1834	4.1902	4.15566
Predicted Endpoint (-log C)	3.59541	4.09361	3.79771
Distance	0.613	0.626	0.653
Reference	EPA COVER SHEET 0288;891101;(1)	EPA COVER SHEET 0337;881201;(1)	EPA COVER SHEET 0027;880301;(1)

Model Applicability

Unknown features are fingerprint features in the query molecule, but not found or appearing too infrequently in the training set.

1. OPS PC22 out of range. Value: -4.7682. Training min, max, SD, explained variance: -4.3287, 5.3383, 1.588, 0.0110.
2. Unknown FCFP_2 feature: 5: [*][O-]
3. Unknown FCFP_2 feature: -828984032: [*][c](:[*]):[c](:[cH]:[*])[N+](=[*])[*]
4. Unknown FCFP_2 feature: -1338588315: [*]:[c](:[*])[N+](=O)[O-]
5. Unknown FCFP_2 feature: 1872392852: [*][N+](=O)[*]
6. Unknown FCFP_2 feature: 260476081: [*][N+](=[*])[O-]
7. Unknown ECFP_6 feature: 912478223: [*]S[*]
8. Unknown ECFP_6 feature: -154530762: [*]N[*]
9. Unknown ECFP_6 feature: 1043790491: [*][N+](=[*])[*]
10. Unknown ECFP_6 feature: 781519895: [*][O-]
11. Unknown ECFP_6 feature: 1790105651: [*]C(=C1C(=[*])[*][*]:[c]1:[*])[*]
12. Unknown ECFP_6 feature: -631778390: [*]C(=C1S[*][*]C1=[*])[*]
13. Unknown ECFP_6 feature: 1945129186: [*]N1[*][*]C(=[*])C1=O
14. Unknown ECFP_6 feature: 190445529: [*]N1[*][*]SC1=O
15. Unknown ECFP_6 feature: 2122741631: [*]=C1[*][*]C(=[*])S1
16. Unknown ECFP_6 feature: -661097313: [*]CN1C(=[*])[*][*]C1=[*]
17. Unknown ECFP_6 feature: 1298725959: [*]NC(=O)C(=[*])[*]
18. Unknown ECFP_6 feature: -1699286547: [*]C(=[*])N[c](:[*]):[*]

19. Unknown ECFP_6 feature: 1335833675: [*]N[c](:[cH]:[*]):[c]([*]):[*]
20. Unknown ECFP_6 feature: 1997021792: [*]:[cH]:[cH]:[cH]:[*]
21. Unknown ECFP_6 feature: -37698365: [*]N([*])CC(=[*])[*]
22. Unknown ECFP_6 feature: 1731843802: [*]CC(=O)N[*]
23. Unknown ECFP_6 feature: 1335108269: [*]N[c](:[cH]:[*]):[c]([*]):[*]
24. Unknown ECFP_6 feature: -1956535100: [*][c](:[*]):[c](:[cH]:[*])[N+](=[*])[*]
25. Unknown ECFP_6 feature: -215026467: [*]:[c](:[*])[N+](=O)[O-]
26. Unknown ECFP_6 feature: 2104376220: [*][N+](=O)[*]
27. Unknown ECFP_6 feature: -659271057: [*][N+](=[*])[O-]

Feature Contribution

Top features for positive contribution

Fingerprint	Bit/Smiles	Feature Structure	Score
FCFP_6	-1143715940	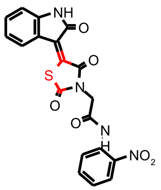 [*]=C1[*][*]C(=[*])S1	0.13
ECFP_6	1559650422	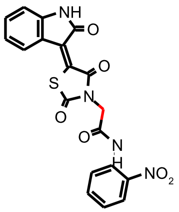 [*]C[*]	0.129
FCFP_6	3	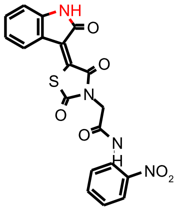 [*]N[*]	0.0924

Top Features for negative contribution

Fingerprint	Bit/Smiles	Feature Structure	Score

FCFP_6	991735244	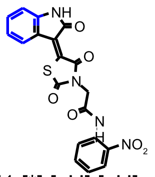 [*][c]1:[*]:[cH]:[cH]:[cH]:[cH]:1	-0.134
ECFP_6	1564392544	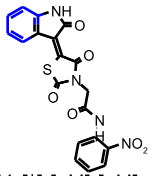 [*][c]1:[*]:[cH]:[cH]:[cH]:[cH]:1	-0.133
ECFP_6	2106656448	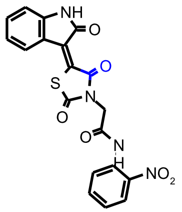 [*]C(=O)[*]	-0.11

Sorafenib

TOPKAT_Chronic_LOAEL

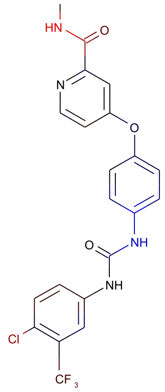

$C_{21}H_{16}ClF_3N_4O_3$

Molecular Weight: 464.82494

ALogP: 4.175

Rotatable Bonds: 6

Acceptors: 4

Donors: 3

Model Prediction

Prediction: 0.00483

Unit: g/kg_body_weight

Mahalanobis Distance: 30

Mahalanobis Distance p-value: 1.21e-024

Mahalanobis Distance: The Mahalanobis distance (MD) is a generalization of the Euclidean distance that accounts for correlations among the X properties. It is calculated as the distance to the center of the training data. The larger the MD, the less trustworthy the prediction.
Mahalanobis Distance p-value: The p-value gives the fraction of training data with an MD greater than or equal to the one for the given sample, assuming normally distributed data. The smaller the p-value, the less trustworthy the prediciton. For highly non-normal X properties (e.g., fingerprints), the MD p-value is wildly inaccurate.

Structural Similar Compounds

Name	GLYBURIDE	D & C RED 9	SODIUM ACIFLUORFEN
Structure	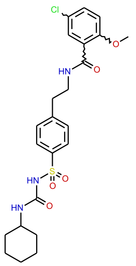	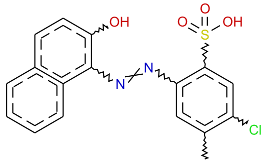	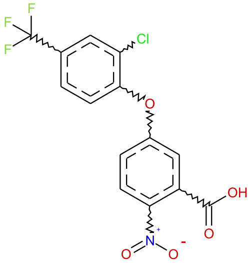
Actual Endpoint (-log C)	4.21661	3.87715	4.16036
Predicted Endpoint (-log C)	4.21035	3.6546	4.65915
Distance	0.636	0.722	0.736
Reference	UPJ-26452	NTP REPORT # 225	EPA COVER SHEET 0192;891101;(1)

Model Applicability

Unknown features are fingerprint features in the query molecule, but not found or appearing too infrequently in the training set.

1. All properties and OPS components are within expected ranges.
2. Unknown ECFP_6 feature: -1046436026: [*]F
3. Unknown ECFP_6 feature: 1305253718: [*]:[c](:[*])O[c](:[*]):[*]
4. Unknown ECFP_6 feature: 1413420509: [*]C(=[*])[c](:[cH]:[*]):n:[*]
5. Unknown ECFP_6 feature: -677309799: [*][c](:[*]):n:[cH]:[*]
6. Unknown ECFP_6 feature: 1996163143: [*]:[cH]:[cH]:n:[*]
7. Unknown ECFP_6 feature: 1430169877: [*]NC(=O)[c](:[*]):[*]
8. Unknown ECFP_6 feature: 1338334141: [*]C(=[*])NC
9. Unknown ECFP_6 feature: 864287155: [*]NC
10. Unknown ECFP_6 feature: -177077903: [*]N[c](:[cH]:[*]):[cH]:[*]
11. Unknown ECFP_6 feature: -649580166: [*]NC(=O)N[*]
12. Unknown ECFP_6 feature: 1336678434: [*][c](:[*]):[c](:[cH]:[*])C([*])([*])[*]
13. Unknown ECFP_6 feature: 99947387: [*]:[c](:[*])Cl
14. Unknown ECFP_6 feature: -1952889961: [*]:[c](:[*])C(F)(F)F
15. Unknown ECFP_6 feature: 226796801: [*]C([*])([*])F

Feature Contribution

Top features for positive contribution

Fingerprint	Bit/Smiles	Feature Structure	Score
ECFP_6	-176455838	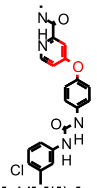 [*]O[c](:[cH]:[*]):[cH]:[*]	0.106
FCFP_6	32	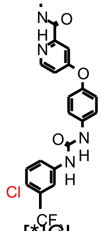 [*]Cl	0.101
FCFP_6	3	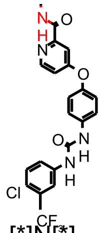 [*]N[*]	0.0924

Top Features for negative contribution

Fingerprint	Bit/Smiles	Feature Structure	Score
FCFP_6	1	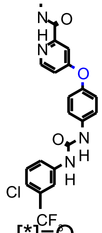 [*]=O	-0.102

ECFP_6	-1236483485	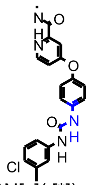 [*]C(=[*])N[c](:[*]):[*]	-0.0747
FCFP_6	203677720	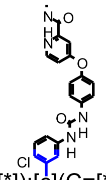 [*][c](:[*]):[c](C=[*]):[cH]:[*]	-0.0713

Sunitinib

TOPKAT_Chronic_LOAEL

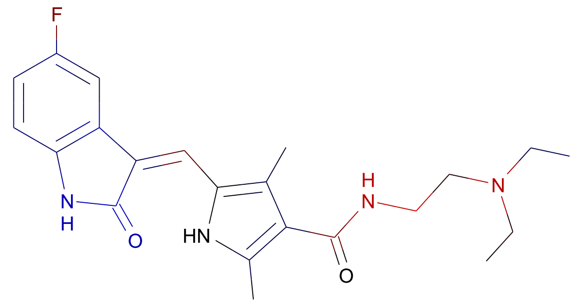

$C_{22}H_{27}FN_4O_2$
Molecular Weight: 398.47378
ALogP: 2.997
Rotatable Bonds: 7
Acceptors: 3
Donors: 3

Model Prediction

Prediction: 0.0397
Unit: g/kg_body_weight
Mahalanobis Distance: 38.1
Mahalanobis Distance p-value: 6.11e-039

Mahalanobis Distance: The Mahalanobis distance (MD) is a generalization of the Euclidean distance that accounts for correlations among the X properties. It is calculated as the distance to the center of the training data. The larger the MD, the less trustworthy the prediction.
Mahalanobis Distance p-value: The p-value gives the fraction of training data with an MD greater than or equal to the one for the given sample, assuming normally distributed data. The smaller the p-value, the less trustworthy the prediciton. For highly non-normal X properties (e.g., fingerprints), the MD p-value is wildly inaccurate.

Structural Similar Compounds

Name	METOCLOPRAMIDE	PROPAFENONE.HCL	ISOXABEN
Structure	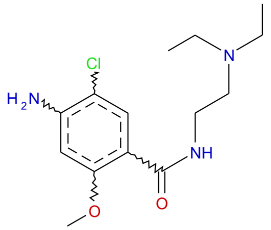	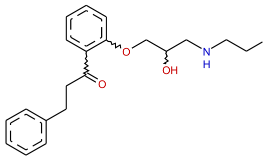	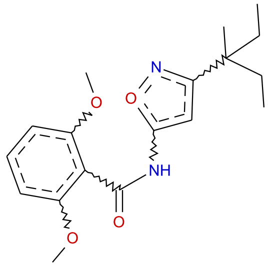
Actual Endpoint (-log C)	4.47683	3.10196	3.81665
Predicted Endpoint (-log C)	3.8785	2.93237	4.42315
Distance	0.630	0.693	0.693
Reference	NDA-17854	NDA-19151	EPA COVER SHEET 0339;881201;(1)

Model Applicability

Unknown features are fingerprint features in the query molecule, but not found or appearing too infrequently in the training set.

1. All properties and OPS components are within expected ranges.
2. Unknown ECFP_6 feature: -152683720: [*]:[nH]:[*]
3. Unknown ECFP_6 feature: -154530762: [*]N[*]
4. Unknown ECFP_6 feature: -1046436026: [*]F
5. Unknown ECFP_6 feature: 558201926: [*][c]1:[*]:[*]:[c]([*]):[nH]:1
6. Unknown ECFP_6 feature: 1791989338: [*][c]1:[*]:[*]:[nH]:[c]:1C
7. Unknown ECFP_6 feature: -1658273810: [*]C(=[*])[c]1:[c]([*]):[*]:[*]:[c]:1[*]
8. Unknown ECFP_6 feature: 1576255326: [*][c]1:[*]:[*]:[c]([*]):[c]:1C
9. Unknown ECFP_6 feature: 980271847: [*][c]1:[*]:[*]:[nH]:[c]:1C=[*]
10. Unknown ECFP_6 feature: 1430169877: [*]NC(=O)[c](:[*]):[*]
11. Unknown ECFP_6 feature: 497523368: [*]CNC(=[*])[*]
12. Unknown ECFP_6 feature: 1182722866: [*]C(=CC(=[*])[*])[*]
13. Unknown ECFP_6 feature: -1699286547: [*]C(=[*])N[c](:[*]):[*]
14. Unknown ECFP_6 feature: 1298725959: [*]NC(=O)C(=[*])[*]
15. Unknown ECFP_6 feature: 1718013682: [*]\C=C\1/C(=[*])[*][*]:[c]1:[*]
16. Unknown ECFP_6 feature: -176686665: [*]:[cH]:[c](F):[cH]:[*]
17. Unknown ECFP_6 feature: 1335833675: [*]N[c](:[cH]:[*]):[c]([*]):[*]
18. Unknown ECFP_6 feature: 220735655: [*]:[c](:[*])F
19. Unknown ECFP_6 feature: -1791034651: [*]CCN[*]

20. Unknown ECFP_6 feature: -1789942192: [*]CCN([*])[*]
21. Unknown ECFP_6 feature: -1866225067: [*]CN(C[*])C[*]
22. Unknown ECFP_6 feature: -949131419: [*]N([*])CC

Feature Contribution

Top features for positive contribution

Fingerprint	Bit/Smiles	Feature Structure	Score
ECFP_6	1559650422	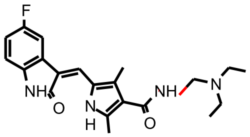 [*]C[*]	0.129
FCFP_6	32	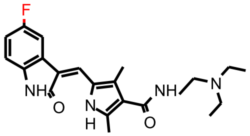 [*]Cl	0.101
FCFP_6	3	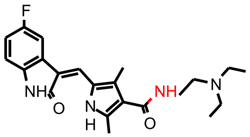 [*]N[*]	0.0924

Top Features for negative contribution

Fingerprint	Bit/Smiles	Feature Structure	Score

ECFP_6	2106656448	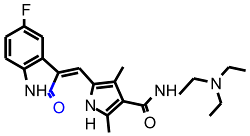 [*]C(=O)[*]	-0.11
FCFP_6	1	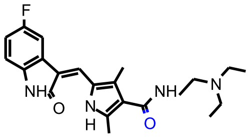 [*]=O	-0.102
FCFP_6	136597326	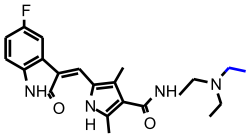 [*]CC	-0.0815

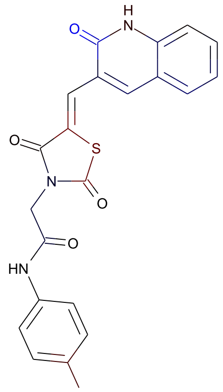

$C_{22}H_{17}N_3O_4S$

Molecular Weight: 419.45307

ALogP: 2.653

Rotatable Bonds: 4

Acceptors: 5

Donors: 2

Model Prediction

Prediction: 0.0378

Unit: g/kg_body_weight

Mahalanobis Distance: 10.9

Mahalanobis Distance p-value: 1.36e-006

Mahalanobis Distance: The Mahalanobis distance (MD) is a generalization of the Euclidean distance that accounts for correlations among the X properties. It is calculated as the distance to the center of the training data. The larger the MD, the less trustworthy the prediction.
Mahalanobis Distance p-value: The p-value gives the fraction of training data with an MD greater than or equal to the one for the given sample, assuming normally distributed data. The smaller the p-value, the less trustworthy the prediciton. For highly non-normal X properties (e.g., fingerprints), the MD p-value is wildly inaccurate.

Structural Similar Compounds

Name	FUROSEMIDE	ACETOHEXAMIDE	DAPSONE
Structure	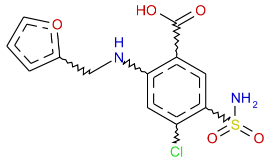	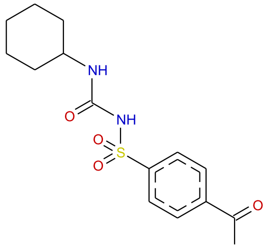	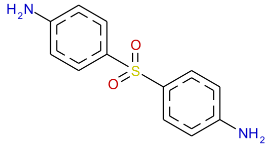
Actual Endpoint (-log C)	4.04236	2.55683	3.66258
Predicted Endpoint (-log C)	2.8614	3.62413	3.26993
Distance	0.647	0.664	0.673
Reference	NCI/NTP TR-356	NCI/NTP TR-050	NCI/NTP TR-20

Model Applicability

Unknown features are fingerprint features in the query molecule, but not found or appearing too infrequently in the training set.

1. All properties and OPS components are within expected ranges.

Feature Contribution

Top features for positive contribution

Fingerprint	Bit/Smiles	Feature Structure	Score
FCFP_2	-1143715940	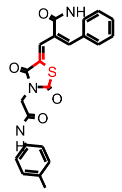 [*]=C1[*][*]C(=[*])S1	0.095

FCFP_2	3	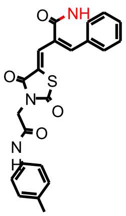 [*]N[*]	0.0737
FCFP_2	136120670	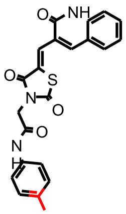 [*]:[c](:[*])C	0.064

Top Features for negative contribution

**Fingerprint**	**Bit/Smiles**	**Feature Structure**	**Score**
FCFP_2	1872154524	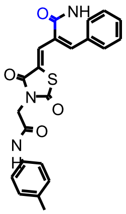 [*]C(=O)[*]	-0.105
FCFP_2	203677720	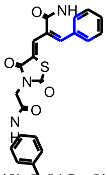 [*][c](:[*]):[c](C=[*]):[cH]:[*]	-0.0829
FCFP_2	1	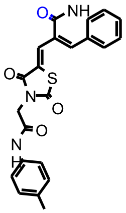 [*]=O	-0.0796

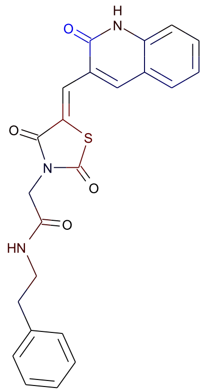

$C_{23}H_{19}N_3O_4S$
Molecular Weight: 433.47965
ALogP: 2.495
Rotatable Bonds: 6
Acceptors: 5
Donors: 2

Model Prediction

Prediction: 0.0401
Unit: g/kg_body_weight
Mahalanobis Distance: 11.8
Mahalanobis Distance p-value: 3.02e-008

Mahalanobis Distance: The Mahalanobis distance (MD) is a generalization of the Euclidean distance that accounts for correlations among the X properties. It is calculated as the distance to the center of the training data. The larger the MD, the less trustworthy the prediction.
Mahalanobis Distance p-value: The p-value gives the fraction of training data with an MD greater than or equal to the one for the given sample, assuming normally distributed data. The smaller the p-value, the less trustworthy the prediciton. For highly non-normal X properties (e.g., fingerprints), the MD p-value is wildly inaccurate.

Structural Similar Compounds

Name	FUROSEMIDE	ACETOHEXAMIDE	TOLAZAMIDE
Structure	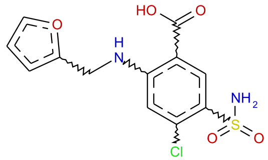	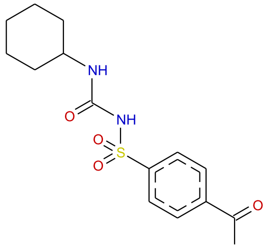	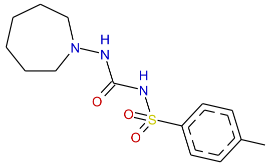
Actual Endpoint (-log C)	4.04236	2.55683	2.84011
Predicted Endpoint (-log C)	2.8614	3.62413	3.59315
Distance	0.663	0.682	0.734
Reference	NCI/NTP TR-356	NCI/NTP TR-050	NCI/NTP TR-051

Model Applicability

Unknown features are fingerprint features in the query molecule, but not found or appearing too infrequently in the training set.

1. All properties and OPS components are within expected ranges.

Feature Contribution

Top features for positive contribution			
**Fingerprint**	**Bit/Smiles**	**Feature Structure**	**Score**
FCFP_2	-885550502	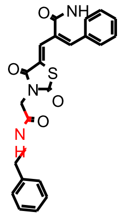 [*]CNC(=[*])[*]	0.115

FCFP_2	-1143715940	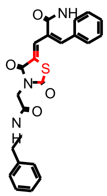<br>[*]=C1[*][*]C(=[*])S1	0.095
FCFP_2	3	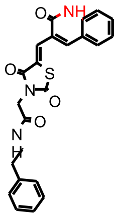<br>[*]N[*]	0.0737

Top Features for negative contribution

Fingerprint	Bit/Smiles	Feature Structure	Score
FCFP_2	1872154524	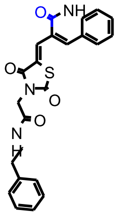<br>[*]C(=O)[*]	-0.105
FCFP_2	203677720	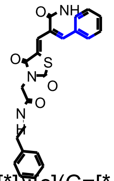<br>[*][c](:[*]):[c](C=[*<br>]):[cH]:[*]	-0.0829
FCFP_2	1	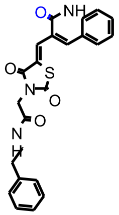<br>[*]=O	-0.0796

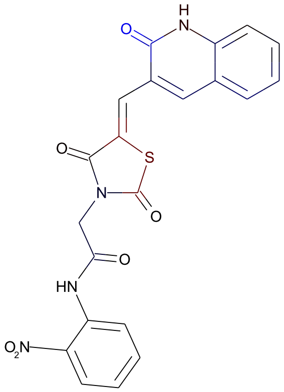

$C_{21}H_{14}N_4O_6S$

Molecular Weight: 450.42405

ALogP: 2.061

Rotatable Bonds: 5

Acceptors: 7

Donors: 2

Structural Similar Compounds

Name	SALICYLAZOSULFAPYRIDINE	FUROSEMIDE	4,4'-DIAMINO-2,2'-STILBENEDISULFONIC ACID.2NaSALT
Structure	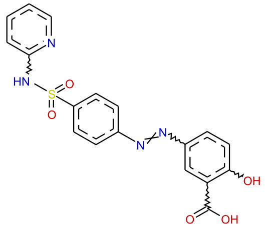	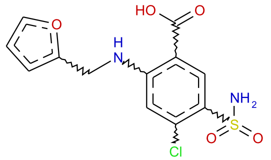	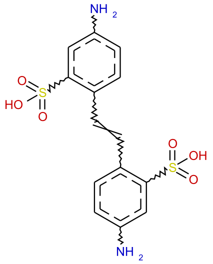
Actual Endpoint (-log C)	3.375	4.04236	2.50759
Predicted Endpoint (-log C)	2.80292	2.8614	3.26068
Distance	0.719	0.725	0.745
Reference	NCI/NTP TR-457	NCI/NTP TR-356	NCI/NTP TR-412

Model Prediction

Prediction: 0.034

Unit: g/kg_body_weight

Mahalanobis Distance: 11.4

Mahalanobis Distance p-value: 1.42e-007

Mahalanobis Distance: The Mahalanobis distance (MD) is a generalization of the Euclidean distance that accounts for correlations among the X properties. It is calculated as the distance to the center of the training data. The larger the MD, the less trustworthy the prediction.
Mahalanobis Distance p-value: The p-value gives the fraction of training data with an MD greater than or equal to the one for the given sample, assuming normally distributed data. The smaller the p-value, the less trustworthy the prediciton. For highly non-normal X properties (e.g., fingerprints), the MD p-value is wildly inaccurate.

Model Applicability

Unknown features are fingerprint features in the query molecule, but not found or appearing too infrequently in the training set.

1. All properties and OPS components are within expected ranges.
2. Unknown FCFP_2 feature: 8: [*][N+](=[*])[*]
3. Unknown FCFP_2 feature: 5: [*][O-]
4. Unknown FCFP_2 feature: -828984032: [*][c](:[*]):[c](:[cH]:[*])[N+](=[*])[*]
5. Unknown FCFP_2 feature: -1338588315: [*]:[c](:[*])[N+](=O)[O-]
6. Unknown FCFP_2 feature: 1872392852: [*][N+](=O)[*]
7. Unknown FCFP_2 feature: 260476081: [*][N+](=[*])[O-]

Feature Contribution

Top features for positive contribution			
**Fingerprint**	**Bit/Smiles**	**Feature Structure**	**Score**

FCFP_2	-1143715940	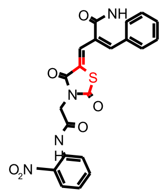<br>[*]=C1[*][*]C(=[*])S1	0.095
FCFP_2	3	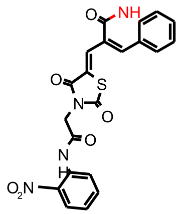<br>[*]N[*]	0.0737
FCFP_2	565998553	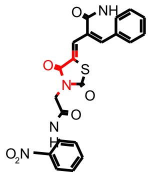<br>[*]N1[*][*]C(=[*])C1=<br>O	0.00813
**Top Features for negative contribution**			
**Fingerprint**	**Bit/Smiles**	**Feature Structure**	**Score**
FCFP_2	1872154524	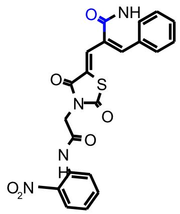<br>[*]C(=O)[*]	-0.105
FCFP_2	203677720	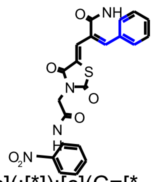<br>[*][c](:[*]):[c](C=[*<br>]):[cH]:[*]	-0.0829

FCFP_2	1	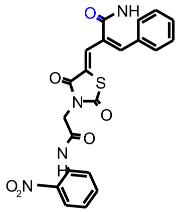<br>[*]=O	-0.0796

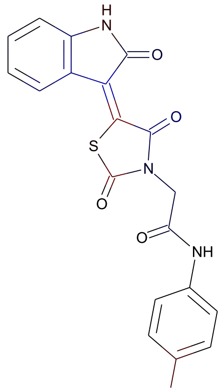

$C_{20}H_{15}N_3O_4S$

Molecular Weight: 393.4158

ALogP: 2.185

Rotatable Bonds: 3

Acceptors: 5

Donors: 2

Model Prediction

Prediction: 0.0398

Unit: g/kg_body_weight

Mahalanobis Distance: 10.8

Mahalanobis Distance p-value: 1.74e-006

Mahalanobis Distance: The Mahalanobis distance (MD) is a generalization of the Euclidean distance that accounts for correlations among the X properties. It is calculated as the distance to the center of the training data. The larger the MD, the less trustworthy the prediction.
Mahalanobis Distance p-value: The p-value gives the fraction of training data with an MD greater than or equal to the one for the given sample, assuming normally distributed data. The smaller the p-value, the less trustworthy the prediciton. For highly non-normal X properties (e.g., fingerprints), the MD p-value is wildly inaccurate.

Structural Similar Compounds

Name	DAPSONE	FUROSEMIDE	ACETOHEXAMIDE
Structure	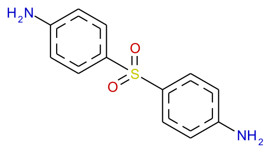	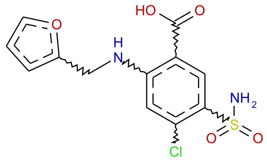	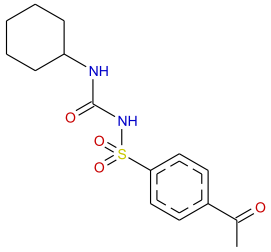
Actual Endpoint (-log C)	3.66258	4.04236	2.55683
Predicted Endpoint (-log C)	3.26993	2.8614	3.62413
Distance	0.594	0.621	0.634
Reference	NCI/NTP TR-20	NCI/NTP TR-356	NCI/NTP TR-050

Model Applicability

Unknown features are fingerprint features in the query molecule, but not found or appearing too infrequently in the training set.

1. All properties and OPS components are within expected ranges.

Feature Contribution

Top features for positive contribution			
**Fingerprint**	**Bit/Smiles**	**Feature Structure**	**Score**
FCFP_2	-1143715940	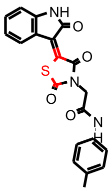 [*]=C1[*][*]C(=[*])S1	0.095

FCFP_2	3	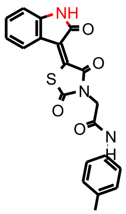 [*]N[*]	0.0737
FCFP_2	136120670	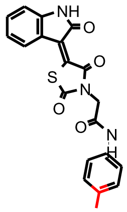 [*]:[c](:[*])C	0.064

Top Features for negative contribution

Fingerprint	Bit/Smiles	Feature Structure	Score
FCFP_2	1872154524	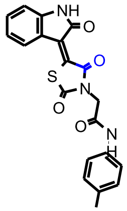 [*]C(=O)[*]	-0.105
FCFP_2	203677720	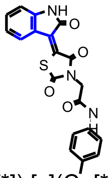 [*][c](:[*]):[c](C=[*<br>]):[cH]:[*]	-0.0829
FCFP_2	1	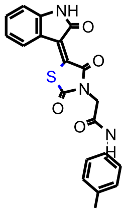 [*]=O	-0.0796

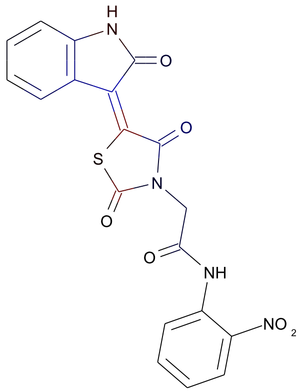

$C_{19}H_{12}N_4O_6S$
Molecular Weight: 424.38678
ALogP: 1.593
Rotatable Bonds: 4
Acceptors: 7
Donors: 2

Model Prediction

Prediction: 0.0359
Unit: g/kg_body_weight
Mahalanobis Distance: 11.4
Mahalanobis Distance p-value: 1.62e-007

Mahalanobis Distance: The Mahalanobis distance (MD) is a generalization of the Euclidean distance that accounts for correlations among the X properties. It is calculated as the distance to the center of the training data. The larger the MD, the less trustworthy the prediction.
Mahalanobis Distance p-value: The p-value gives the fraction of training data with an MD greater than or equal to the one for the given sample, assuming normally distributed data. The smaller the p-value, the less trustworthy the prediciton. For highly non-normal X properties (e.g., fingerprints), the MD p-value is wildly inaccurate.

Structural Similar Compounds

Name	FUROSEMIDE	4,4'-DIAMINO-2,2'-STILBENEDISULFONIC ACID.2NaSALT	SALICYLAZOSULFAPYRIDINE
Structure	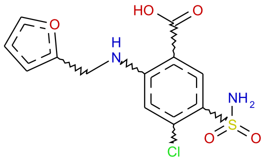	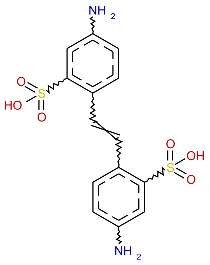	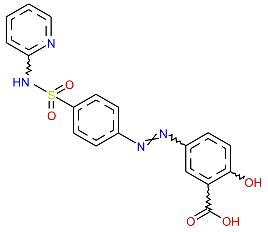
Actual Endpoint (-log C)	4.04236	2.50759	3.375
Predicted Endpoint (-log C)	2.8614	3.26068	2.80292
Distance	0.695	0.733	0.736
Reference	NCI/NTP TR-356	NCI/NTP TR-412	NCI/NTP TR-457

Model Applicability

Unknown features are fingerprint features in the query molecule, but not found or appearing too infrequently in the training set.

1. All properties and OPS components are within expected ranges.
2. Unknown FCFP_2 feature: 8: [*][N+](=[*])[*]
3. Unknown FCFP_2 feature: 5: [*][O-]
4. Unknown FCFP_2 feature: -828984032: [*][c](:[*]):[c](:[cH]:[*])[N+](=[*])[*]
5. Unknown FCFP_2 feature: -1338588315: [*]:[c](:[*])[N+](=O)[O-]
6. Unknown FCFP_2 feature: 1872392852: [*][N+](=O)[*]
7. Unknown FCFP_2 feature: 260476081: [*][N+](=[*])[O-]

Feature Contribution

Top features for positive contribution			
Fingerprint	Bit/Smiles	Feature Structure	Score

FCFP_2	-1143715940	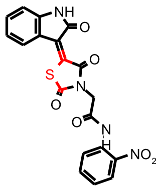 [*]=C1[*][*]C(=[*])S1	0.095
FCFP_2	3	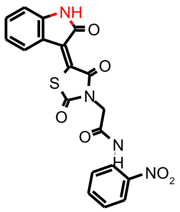 [*]N[*]	0.0737
FCFP_2	565998553	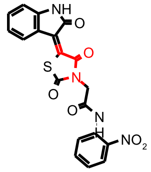 [*]N1[*][*]C(=[*])C1=O	0.00813

Top Features for negative contribution

Fingerprint	Bit/Smiles	Feature Structure	Score
FCFP_2	1872154524	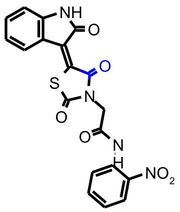 [*]C(=O)[*]	-0.105
FCFP_2	203677720	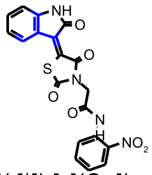 [*][c](:[*]):[c](C=[*]):[cH]:[*]	-0.0829

FCFP_2	1	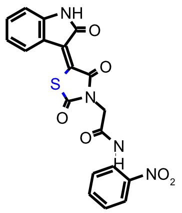 [*]=O	-0.0796

Sorafenib

TOPKAT_Rat_Maximum_Tolerated_Dose_Feed

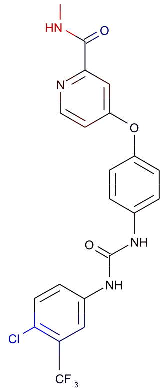

$C_{21}H_{16}ClF_3N_4O_3$

Molecular Weight: 464.82494

ALogP: 4.175

Rotatable Bonds: 6

Acceptors: 4

Donors: 3

Model Prediction

Prediction: 0.0885

Unit: g/kg_body_weight

Mahalanobis Distance: 12.4

Mahalanobis Distance p-value: 1.76e-009

Mahalanobis Distance: The Mahalanobis distance (MD) is a generalization of the Euclidean distance that accounts for correlations among the X properties. It is calculated as the distance to the center of the training data. The larger the MD, the less trustworthy the prediction.
Mahalanobis Distance p-value: The p-value gives the fraction of training data with an MD greater than or equal to the one for the given sample, assuming normally distributed data. The smaller the p-value, the less trustworthy the prediciton. For highly non-normal X properties (e.g., fingerprints), the MD p-value is wildly inaccurate.

Structural Similar Compounds

Name	FUROSEMIDE	PHENOLPHTHALEIN	DISPERSE YELLOW 3
Structure	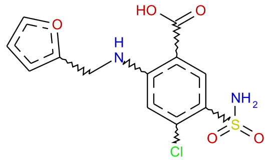	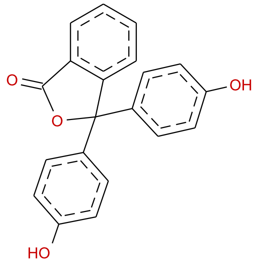	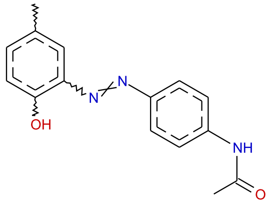
Actual Endpoint (-log C)	4.04236	2.20184	2.77703
Predicted Endpoint (-log C)	2.8614	2.8857	2.80195
Distance	0.741	0.780	0.799
Reference	NCI/NTP TR-356	NCI/NTP TR-465	NCI/NTP TR-222

Model Applicability

Unknown features are fingerprint features in the query molecule, but not found or appearing too infrequently in the training set.

1. All properties and OPS components are within expected ranges.

Feature Contribution

Top features for positive contribution

Fingerprint	Bit/Smiles	Feature Structure	Score
FCFP_2	-885550502	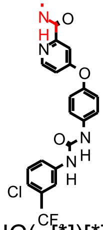 [*]CNC(=[*])[*]	0.115

FCFP_2	3	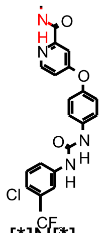 [*]N[*]	0.0737
FCFP_2	332760439	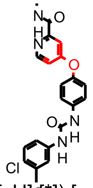 [*]O[c](:[cH]c[*]):[cH]:[*]	0.0611

Top Features for negative contribution

Fingerprint	Bit/Smiles	Feature Structure	Score
FCFP_2	71476542	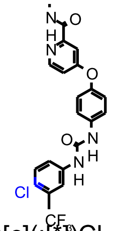 [*]:[c](:[*])Cl	-0.134
FCFP_2	1872154524	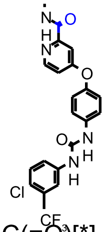 [*]C(=O)[*]	-0.105
FCFP_2	203677720	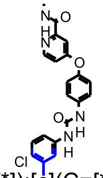 [*][c](:[*]):[c](C=[*]):[cH]:[*]	-0.0829

Sunitinib

TOPKAT_Rat_Maximum_Tolerated_Dose_Feed

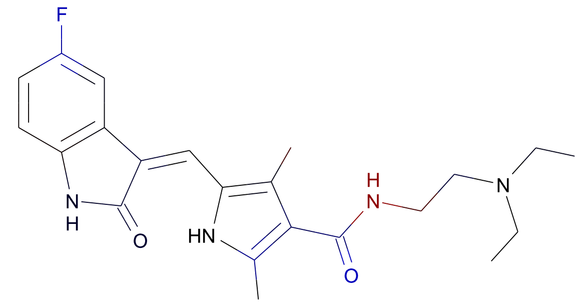

$C_{22}H_{27}FN_4O_2$

Molecular Weight: 398.47378

ALogP: 2.997

Rotatable Bonds: 7

Acceptors: 3

Donors: 3

Model Prediction

Prediction: 0.178

Unit: g/kg_body_weight

Mahalanobis Distance: 9.78

Mahalanobis Distance p-value: 9.94e-005

Mahalanobis Distance: The Mahalanobis distance (MD) is a generalization of the Euclidean distance that accounts for correlations among the X properties. It is calculated as the distance to the center of the training data. The larger the MD, the less trustworthy the prediction.
Mahalanobis Distance p-value: The p-value gives the fraction of training data with an MD greater than or equal to the one for the given sample, assuming normally distributed data. The smaller the p-value, the less trustworthy the prediciton. For highly non-normal X properties (e.g., fingerprints), the MD p-value is wildly inaccurate.

Structural Similar Compounds

Name	CHLORPROPAMIDE	DISPERSE YELLOW 3	TOLBUTAMIDE
Structure	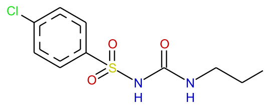	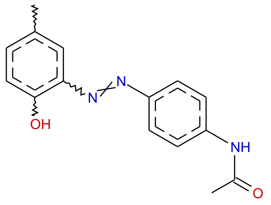	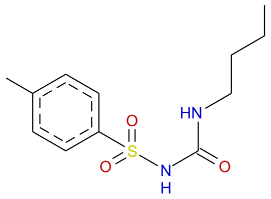
Actual Endpoint (-log C)	3.0107	2.77703	2.3985
Predicted Endpoint (-log C)	3.18321	2.80195	3.32272
Distance	0.657	0.661	0.672
Reference	NCI/NTP TR-045	NCI/NTP TR-222	NCI/NTP TR-031

Model Applicability

Unknown features are fingerprint features in the query molecule, but not found or appearing too infrequently in the training set.

1. All properties and OPS components are within expected ranges.

Feature Contribution

Top features for positive contribution

Fingerprint	Bit/Smiles	Feature Structure	Score
FCFP_2	-885550502	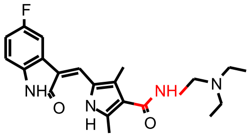 [*]CNC(=[*])[*]	0.115

FCFP_2	3	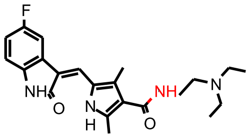 [*]N[*]	0.0737
FCFP_2	136120670	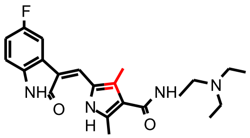 [*]:[c](:[*])C	0.064
**Top Features for negative contribution**			
**Fingerprint**	**Bit/Smiles**	**Feature Structure**	**Score**
FCFP_2	71476542	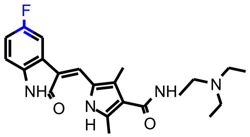 [*]:[c](:[*])Cl	-0.134
FCFP_2	1872154524	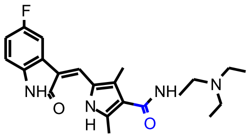 [*]C(=O)[*]	-0.105
FCFP_2	203677720	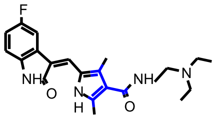 [*][c](:[*]):[c](C=[*]):[cH]:[*]	-0.0829

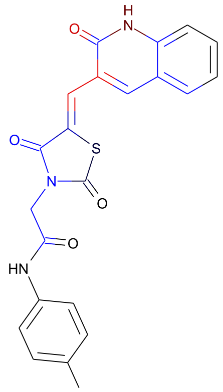

$C_{22}H_{17}N_3O_4S$
Molecular Weight: 419.45307
ALogP: 2.653
Rotatable Bonds: 4
Acceptors: 5
Donors: 2

Model Prediction

Prediction: 0.0943
Unit: g/kg_body_weight
Mahalanobis Distance: 8.87
Mahalanobis Distance p-value: 0.000181

Mahalanobis Distance: The Mahalanobis distance (MD) is a generalization of the Euclidean distance that accounts for correlations among the X properties. It is calculated as the distance to the center of the training data. The larger the MD, the less trustworthy the prediction.
Mahalanobis Distance p-value: The p-value gives the fraction of training data with an MD greater than or equal to the one for the given sample, assuming normally distributed data. The smaller the p-value, the less trustworthy the prediciton. For highly non-normal X properties (e.g., fingerprints), the MD p-value is wildly inaccurate.

Structural Similar Compounds

Name	OCHRATOXIN	PENICILLIN VK	SULFISOOXAZOLE
Structure	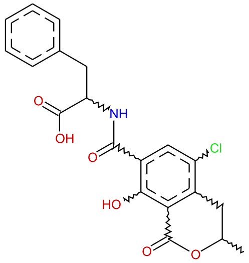	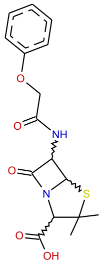	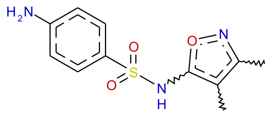
Actual Endpoint (-log C)	6.28396	2.54455	2.82494
Predicted Endpoint (-log C)	5.12358	3.9702	3.0705
Distance	0.651	0.693	0.706
Reference	NCI/NTP TR-358	NCI/NTP TR-336	NCI/NTP TR-138

Model Applicability

Unknown features are fingerprint features in the query molecule, but not found or appearing too infrequently in the training set.

1. All properties and OPS components are within expected ranges.
2. Unknown FCFP_2 feature: 436915834: [*]\C=C\1/S[*][*]C1=[*]
3. Unknown FCFP_2 feature: -1986158408: [*]N1[*][*]SC1=O

Feature Contribution

Top features for positive contribution			
**Fingerprint**	**Bit/Smiles**	**Feature Structure**	**Score**
FCFP_2	1	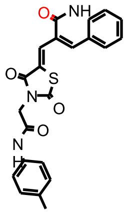 [*]=O	0.511

FCFP_2	451847724	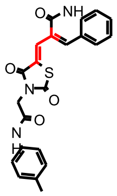 [*]C(=CC(=[*])[*])[*]	0.225
FCFP_2	3	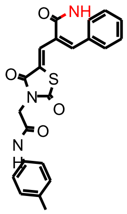 [*]N[*]	0.104

Top Features for negative contribution

**Fingerprint**	**Bit/Smiles**	**Feature Structure**	**Score**
FCFP_2	203677720	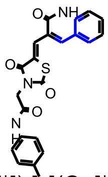 [*][c](:[*]):[c](C=[*]):[cH]:[*]	-0.406
FCFP_2	565998553	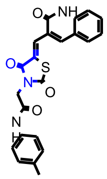 [*]N1[*][*]C(=[*])C1= O	-0.348
FCFP_2	1872154524	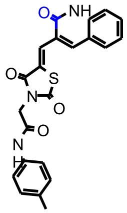 [*]C(=O)[*]	-0.307

2 TOPKAT_Rat_Maximum_Tolerated_Dose_Gavage

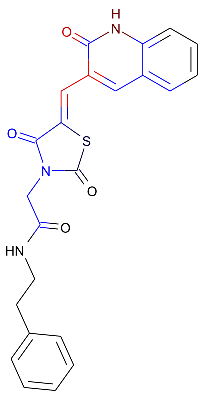

$C_{23}H_{19}N_3O_4S$
Molecular Weight: 433.47965
ALogP: 2.495
Rotatable Bonds: 6
Acceptors: 5
Donors: 2

Model Prediction

Prediction: 0.225
Unit: g/kg_body_weight
Mahalanobis Distance: 8.35
Mahalanobis Distance p-value: 0.000789

Mahalanobis Distance: The Mahalanobis distance (MD) is a generalization of the Euclidean distance that accounts for correlations among the X properties. It is calculated as the distance to the center of the training data. The larger the MD, the less trustworthy the prediction.
Mahalanobis Distance p-value: The p-value gives the fraction of training data with an MD greater than or equal to the one for the given sample, assuming normally distributed data. The smaller the p-value, the less trustworthy the prediciton. For highly non-normal X properties (e.g., fingerprints), the MD p-value is wildly inaccurate.

Structural Similar Compounds

Name	OCHRATOXIN	PENICILLIN VK	SULFISOOXAZOLE
Structure	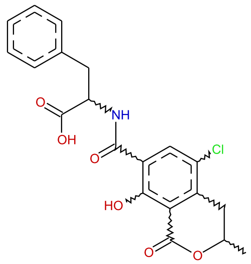	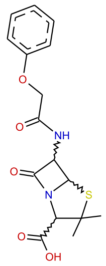	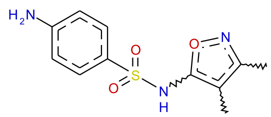
Actual Endpoint (-log C)	6.28396	2.54455	2.82494
Predicted Endpoint (-log C)	5.12358	3.9702	3.0705
Distance	0.633	0.688	0.797
Reference	NCI/NTP TR-358	NCI/NTP TR-336	NCI/NTP TR-138

Model Applicability

Unknown features are fingerprint features in the query molecule, but not found or appearing too infrequently in the training set.

1. All properties and OPS components are within expected ranges.
2. Unknown FCFP_2 feature: 436915834: [*]\C=C\1/S[*][*]C1=[*]
3. Unknown FCFP_2 feature: -1986158408: [*]N1[*][*]SC1=O

Feature Contribution

Top features for positive contribution

Fingerprint	Bit/Smiles	Feature Structure	Score
FCFP_2	1	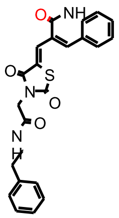 [*]=O	0.511

FCFP_2	451847724	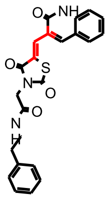 [*]C(=CC(=[*])[*])[*]	0.225
FCFP_2	3	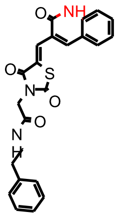 [*]N[*]	0.104

Top Features for negative contribution

Fingerprint	Bit/Smiles	Feature Structure	Score
FCFP_2	203677720	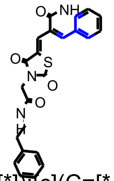 [*][c](:[*]):[c](C=[*])):[cH]:[*]	-0.406
FCFP_2	565998553	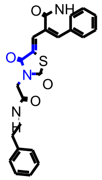 [*]N1[*][*]C(=[*])C1=O	-0.348
FCFP_2	1872154524	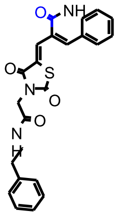 [*]C(=O)[*]	-0.307

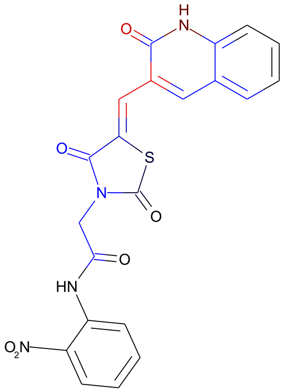

$C_{21}H_{14}N_4O_6S$
Molecular Weight: 450.42405
ALogP: 2.061
Rotatable Bonds: 5
Acceptors: 7
Donors: 2

Model Prediction

Prediction: 0.0376
Unit: g/kg_body_weight
Mahalanobis Distance: 10.6
Mahalanobis Distance p-value: 8.06e-007

Mahalanobis Distance: The Mahalanobis distance (MD) is a generalization of the Euclidean distance that accounts for correlations among the X properties. It is calculated as the distance to the center of the training data. The larger the MD, the less trustworthy the prediction.
Mahalanobis Distance p-value: The p-value gives the fraction of training data with an MD greater than or equal to the one for the given sample, assuming normally distributed data. The smaller the p-value, the less trustworthy the prediciton. For highly non-normal X properties (e.g., fingerprints), the MD p-value is wildly inaccurate.

Structural Similar Compounds

Name	PENICILLIN VK	OCHRATOXIN	AMPICILLIN TRIHYDRATE
Structure	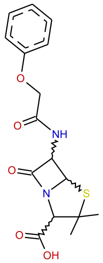	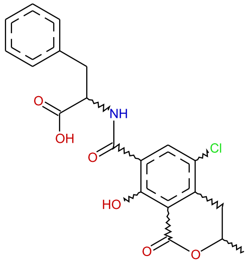	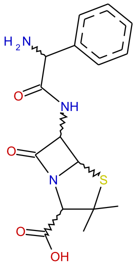
Actual Endpoint (-log C)	2.54455	6.28396	2.36724
Predicted Endpoint (-log C)	3.9702	5.12358	2.27651
Distance	0.853	0.861	1.002
Reference	NCI/NTP TR-336	NCI/NTP TR-358	NCI/NTP TR-318

Model Applicability

Unknown features are fingerprint features in the query molecule, but not found or appearing too infrequently in the training set.

1. Molecular_Weight out of range. Value: 450.42. Training min, max, mean, SD: 68.074, 434.63, 171.13, 85.06.
2. Num_H_Acceptors out of range. Value: 7. Training min, max, mean, SD: 0, 6, 1.6146, 1.644.
3. Molecular_PolarSASA out of range. Value: 250.85. Training min, max, mean, SD: 0, 223.97, 50.816, 55.15.
4. Molecular_PolarSurfaceArea out of range. Value: 166.7. Training min, max, mean, SD: 0, 138.03, 28.978, 32.1.
5. Unknown FCFP_2 feature: 8: [*][N+](=[*])[*]
6. Unknown FCFP_2 feature: 5: [*][O-]
7. Unknown FCFP_2 feature: 436915834: [*]\C=C\1/S[*][*]C1=[*]
8. Unknown FCFP_2 feature: -1986158408: [*]N1[*][*]SC1=O
9. Unknown FCFP_2 feature: -828984032: [*][c](:[*]):[c](:[cH]:[*])[N+](=[*])[*]
10. Unknown FCFP_2 feature: -1338588315: [*]:[c](:[*])[N+](=O)[O-]
11. Unknown FCFP_2 feature: 1872392852: [*][N+](=O)[*]
12. Unknown FCFP_2 feature: 260476081: [*][N+](=[*])[O-]

Feature Contribution

Top features for positive contribution			
Fingerprint	Bit/Smiles	Feature Structure	Score

FCFP_2	1	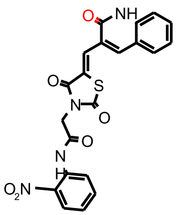 [*]=O	0.511
FCFP_2	451847724	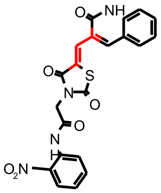 [*]C(=CC(=[*])[*])[*]	0.225
FCFP_2	3	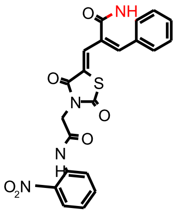 [*]N[*]	0.104

Top Features for negative contribution

Fingerprint	Bit/Smiles	Feature Structure	Score
FCFP_2	203677720	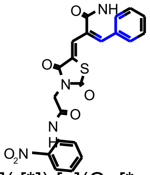 [*][c](:[*]):[c](C=[*]):[cH]:[*]	-0.406
FCFP_2	565998553	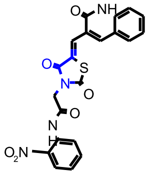 [*]N1[*][*]C(=[*])C1= O	-0.348

FCFP_2	1872154524	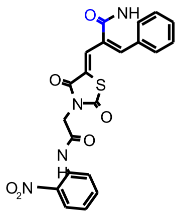[*]C(=O)[*]	-0.307

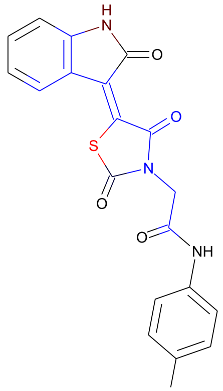

$C_{20}H_{15}N_3O_4S$
Molecular Weight: 393.4158
ALogP: 2.185
Rotatable Bonds: 3
Acceptors: 5
Donors: 2

Model Prediction

Prediction: 0.138
Unit: g/kg_body_weight
Mahalanobis Distance: 8
Mahalanobis Distance p-value: 0.00206

Mahalanobis Distance: The Mahalanobis distance (MD) is a generalization of the Euclidean distance that accounts for correlations among the X properties. It is calculated as the distance to the center of the training data. The larger the MD, the less trustworthy the prediction.
Mahalanobis Distance p-value: The p-value gives the fraction of training data with an MD greater than or equal to the one for the given sample, assuming normally distributed data. The smaller the p-value, the less trustworthy the prediciton. For highly non-normal X properties (e.g., fingerprints), the MD p-value is wildly inaccurate.

Structural Similar Compounds

Name	SULFISOOXAZOLE	PENICILLIN VK	OCHRATOXIN
Structure	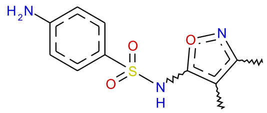	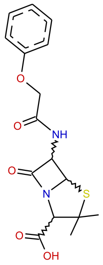	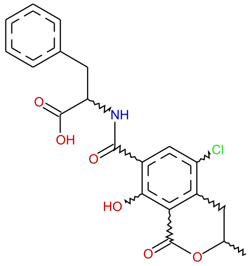
Actual Endpoint (-log C)	2.82494	2.54455	6.28396
Predicted Endpoint (-log C)	3.0705	3.9702	5.12358
Distance	0.618	0.657	0.682
Reference	NCI/NTP TR-138	NCI/NTP TR-336	NCI/NTP TR-358

Model Applicability

Unknown features are fingerprint features in the query molecule, but not found or appearing too infrequently in the training set.

1. All properties and OPS components are within expected ranges.
2. Unknown FCFP_2 feature: -1678275541: [*]C(=C1C(=[*])[*][*]:[c]1:[*])[*]
3. Unknown FCFP_2 feature: 436915834: [*]\C=C\1/S[*][*]C1=[*]
4. Unknown FCFP_2 feature: -1986158408: [*]N1[*][*]SC1=O

Feature Contribution

Top features for positive contribution

Fingerprint	Bit/Smiles	Feature Structure	Score
FCFP_2	1	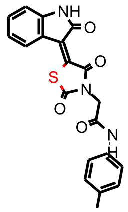 [*]=O	0.511

FCFP_2	3	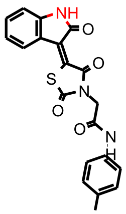<br>[*]N[*]	0.104
**Top Features for negative contribution**			
**Fingerprint**	**Bit/Smiles**	**Feature Structure**	**Score**
FCFP_2	203677720	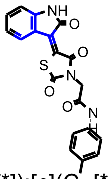<br>[*][c](:[*]):[c](C=[*]):[cH]:[*]	-0.406
FCFP_2	565998553	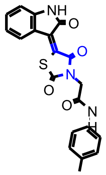<br>[*]N1[*][*]C(=[*])C1= O	-0.348
FCFP_2	1872154524	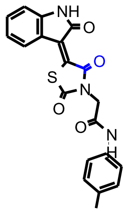<br>[*]C(=O)[*]	-0.307

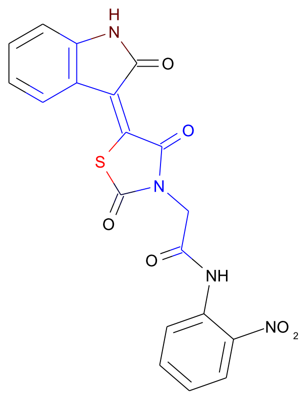

$C_{19}H_{12}N_4O_6S$

Molecular Weight: 424.38678

ALogP: 1.593

Rotatable Bonds: 4

Acceptors: 7

Donors: 2

Model Prediction

Prediction: 0.0551

Unit: g/kg_body_weight

Mahalanobis Distance: 10

Mahalanobis Distance p-value: 5.09e-006

Mahalanobis Distance: The Mahalanobis distance (MD) is a generalization of the Euclidean distance that accounts for correlations among the X properties. It is calculated as the distance to the center of the training data. The larger the MD, the less trustworthy the prediction.
Mahalanobis Distance p-value: The p-value gives the fraction of training data with an MD greater than or equal to the one for the given sample, assuming normally distributed data. The smaller the p-value, the less trustworthy the prediciton. For highly non-normal X properties (e.g., fingerprints), the MD p-value is wildly inaccurate.

Structural Similar Compounds

Name	PENICILLIN VK	OCHRATOXIN	AMPICILLIN TRIHYDRATE
Structure	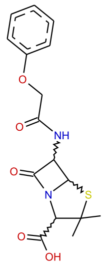	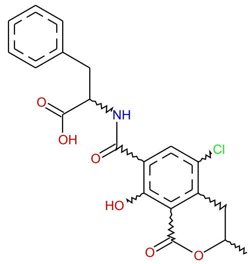	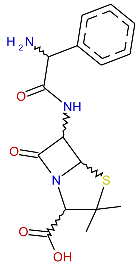
Actual Endpoint (-log C)	2.54455	6.28396	2.36724
Predicted Endpoint (-log C)	3.9702	5.12358	2.27651
Distance	0.818	0.869	0.937
Reference	NCI/NTP TR-336	NCI/NTP TR-358	NCI/NTP TR-318

Model Applicability

Unknown features are fingerprint features in the query molecule, but not found or appearing too infrequently in the training set.

1. Num_H_Acceptors out of range. Value: 7. Training min, max, mean, SD: 0, 6, 1.6146, 1.644.
2. Molecular_PolarSASA out of range. Value: 250.85. Training min, max, mean, SD: 0, 223.97, 50.816, 55.15.
3. Molecular_PolarSurfaceArea out of range. Value: 166.7. Training min, max, mean, SD: 0, 138.03, 28.978, 32.1.
4. Unknown FCFP_2 feature: 8: [*][N+](=[*])[*]
5. Unknown FCFP_2 feature: 5: [*][O-]
6. Unknown FCFP_2 feature: -1678275541: [*]C(=C1C(=[*])[*][*]:[c]1:[*])[*]
7. Unknown FCFP_2 feature: 436915834: [*]\C=C\1/S[*][*]C1=[*]
8. Unknown FCFP_2 feature: -1986158408: [*]N1[*][*]SC1=O
9. Unknown FCFP_2 feature: -828984032: [*][c](:[*]):[c](:[cH]:[*])[N+](=[*])[*]
10. Unknown FCFP_2 feature: -1338588315: [*]:[c](:[*])[N+](=O)[O-]
11. Unknown FCFP_2 feature: 1872392852: [*][N+](=O)[*]
12. Unknown FCFP_2 feature: 260476081: [*][N+](=[*])[O-]

Feature Contribution

Top features for positive contribution

Fingerprint	Bit/Smiles	Feature Structure	Score

FCFP_2	1	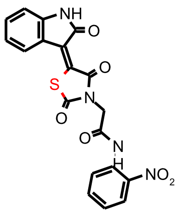 [*]=O	0.511
FCFP_2	3	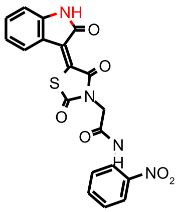 [*]N[*]	0.104
**Top Features for negative contribution**			
**Fingerprint**	**Bit/Smiles**	**Feature Structure**	**Score**
FCFP_2	203677720	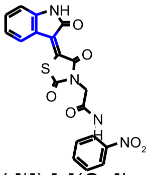 [*][c](:[*]):[c](C=[*]):[cH]:[*]	-0.406
FCFP_2	565998553	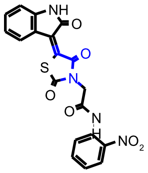 [*]N1[*][*]C(=[*])C1= O	-0.348
FCFP_2	1872154524	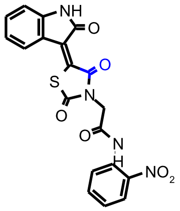 [*]C(=O)[*]	-0.307

Sorafenib

TOPKAT_Rat_Maximum_Tolerated_Dose_Gavage

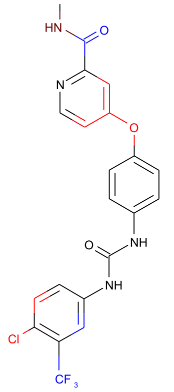

$C_{21}H_{16}ClF_3N_4O_3$

Molecular Weight: 464.82494

ALogP: 4.175

Rotatable Bonds: 6

Acceptors: 4

Donors: 3

Model Prediction

Prediction: 0.000918

Unit: g/kg_body_weight

Mahalanobis Distance: 12.2

Mahalanobis Distance p-value: 4.69e-009

Mahalanobis Distance: The Mahalanobis distance (MD) is a generalization of the Euclidean distance that accounts for correlations among the X properties. It is calculated as the distance to the center of the training data. The larger the MD, the less trustworthy the prediction.
Mahalanobis Distance p-value: The p-value gives the fraction of training data with an MD greater than or equal to the one for the given sample, assuming normally distributed data. The smaller the p-value, the less trustworthy the prediciton. For highly non-normal X properties (e.g., fingerprints), the MD p-value is wildly inaccurate.

Structural Similar Compounds

Name	OCHRATOXIN	SULFISOOXAZOLE	PENICILLIN VK
Structure	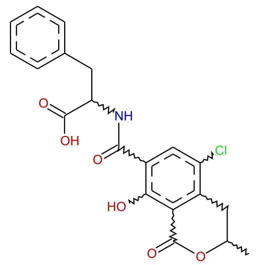	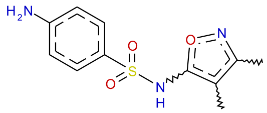	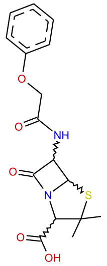
Actual Endpoint (-log C)	6.28396	2.82494	2.54455
Predicted Endpoint (-log C)	5.12358	3.0705	3.9702
Distance	0.758	0.997	1.159
Reference	NCI/NTP TR-358	NCI/NTP TR-138	NCI/NTP TR-336

Model Applicability

Unknown features are fingerprint features in the query molecule, but not found or appearing too infrequently in the training set.

1. Molecular_Weight out of range. Value: 464.82. Training min, max, mean, SD: 68.074, 434.63, 171.13, 85.06.
2. Num_AromaticRings out of range. Value: 3. Training min, max, mean, SD: 0, 2, 0.5625, 0.693.
3. OPS PC5 out of range. Value: -3.5737. Training min, max, SD, explained variance: -3.4, 4.1587, 1.489, 0.0686.
4. OPS PC7 out of range. Value: -3.8342. Training min, max, SD, explained variance: -2.8003, 2.9332, 1.16, 0.0416.
5. Unknown FCFP_2 feature: 136686699: [*]NC
6. Unknown FCFP_2 feature: 1499521844: [*]NC(=O)N[*]
7. Unknown FCFP_2 feature: -1029533685: [*]:[c](:[*])C(F)(F)F

Feature Contribution

Top features for positive contribution

Fingerprint	Bit/Smiles	Feature Structure	Score

FCFP_2	332760439	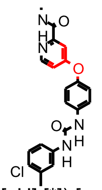 [*]O[c](:[cH]:[*]):[cH]:[*]	0.672
FCFP_2	32	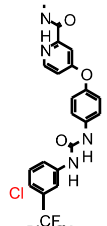 [*]Cl	0.526
FCFP_2	1	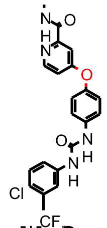 [*]=O	0.511

Top Features for negative contribution

Fingerprint	Bit/Smiles	Feature Structure	Score
FCFP_2	203677720	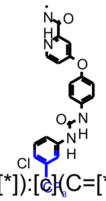 [*][c](:[*]):[c](C=[*]):[cH]:[*]	-0.406
FCFP_2	1872154524	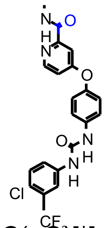 [*]C(=O)[*]	-0.307

FCFP_2	0	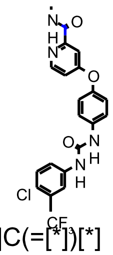 [*]C(=[*])[*]	-0.29

Sunitinib

TOPKAT_Rat_Maximum_Tolerated_Dose_Gavage

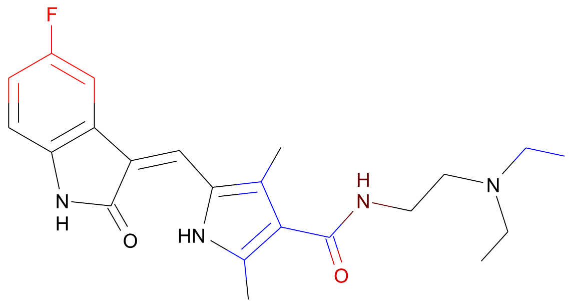

$C_{22}H_{27}FN_4O_2$

Molecular Weight: 398.47378

ALogP: 2.997

Rotatable Bonds: 7

Acceptors: 3

Donors: 3

Model Prediction

Prediction: 0.161

Unit: g/kg_body_weight

Mahalanobis Distance: 9.68

Mahalanobis Distance p-value: 1.53e-005

Mahalanobis Distance: The Mahalanobis distance (MD) is a generalization of the Euclidean distance that accounts for correlations among the X properties. It is calculated as the distance to the center of the training data. The larger the MD, the less trustworthy the prediction.
Mahalanobis Distance p-value: The p-value gives the fraction of training data with an MD greater than or equal to the one for the given sample, assuming normally distributed data. The smaller the p-value, the less trustworthy the prediciton. For highly non-normal X properties (e.g., fingerprints), the MD p-value is wildly inaccurate.

Structural Similar Compounds

Name	OCHRATOXIN	SULFISOOXAZOLE	PROBENECID
Structure	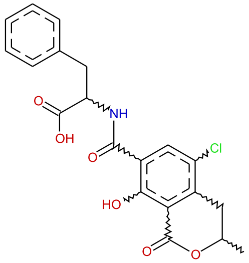	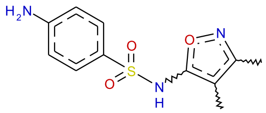	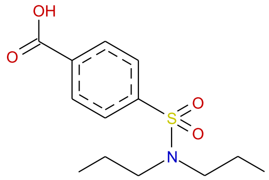
Actual Endpoint (-log C)	6.28396	2.82494	2.85333
Predicted Endpoint (-log C)	5.12358	3.0705	2.4258
Distance	0.784	0.908	0.912
Reference	NCI/NTP TR-358	NCI/NTP TR-138	NCI/NTP TR-395

Model Applicability

Unknown features are fingerprint features in the query molecule, but not found or appearing too infrequently in the training set.

1. OPS PC7 out of range. Value: -3.6239. Training min, max, SD, explained variance: -2.8003, 2.9332, 1.16, 0.0416.
2. Unknown FCFP_2 feature: 19: [*]:[nH]:[*]
3. Unknown FCFP_2 feature: 2005402822: [*][c]1:[*]:[*]:[c]([*]):[nH]:1
4. Unknown FCFP_2 feature: 203707511: [*][c]1:[*]:[*]:[nH]:[c]:1C
5. Unknown FCFP_2 feature: -1678275541: [*]C(=C1C(=[*])[*][*]:[c]1:[*])[*]

Feature Contribution

Top features for positive contribution

Fingerprint	Bit/Smiles	Feature Structure	Score
FCFP_2	32	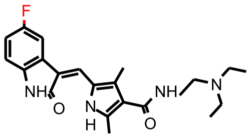 [*]Cl	0.526

FCFP_2	1	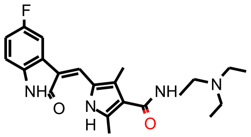 [*]=O	0.511
FCFP_2	367998008	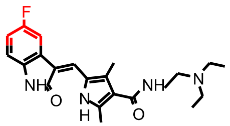 [*][c](:[*]):[c](Cl):[cH]:[*]	0.413
**Top Features for negative contribution**			
**Fingerprint**	**Bit/Smiles**	**Feature Structure**	**Score**
FCFP_2	136597326	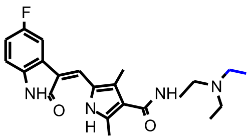 [*]CC	-0.489
FCFP_2	203677720	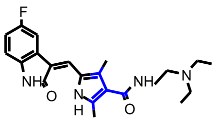 [*][c](:[*]):[c](C=[*]):[cH]:[*]	-0.406
FCFP_2	1872154524	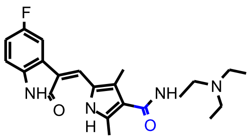 [*]C(=O)[*]	-0.307

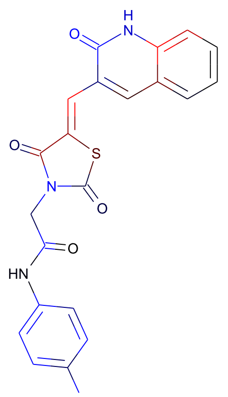

$C_{22}H_{17}N_3O_4S$

Molecular Weight: 419.45307

ALogP: 2.653

Rotatable Bonds: 4

Acceptors: 5

Donors: 2

Structural Similar Compounds

Name	PIRETANIDE	CARBAMIC ACID; [1-[(5-CYANOPENTYL)CARBAMOYL]BENZIMIDAZOL-2-YL]-; METHYL ESTER	OCHRATOXIN A
Structure	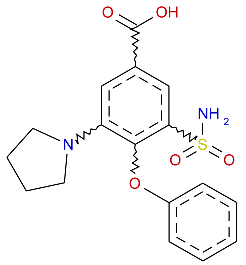	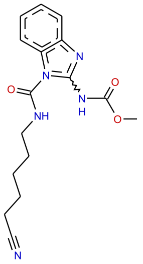	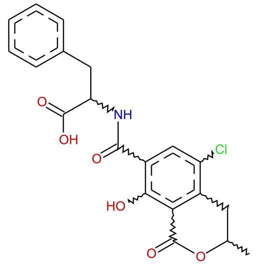
Actual Endpoint (-log C)	1.811	2.12	4.305
Predicted Endpoint (-log C)	1.83976	1.78415	3.03558
Distance	0.557	0.612	0.615
Reference	DRFUD4 2;393;77	85ARAE 4;118;76/77	FCTXAV 6;479;68

Model Prediction

Prediction: 2.01

Unit: g/kg_body_weight

Mahalanobis Distance: 23.7

Mahalanobis Distance p-value: 3.41e-024

Mahalanobis Distance: The Mahalanobis distance (MD) is a generalization of the Euclidean distance that accounts for correlations among the X properties. It is calculated as the distance to the center of the training data. The larger the MD, the less trustworthy the prediction.
Mahalanobis Distance p-value: The p-value gives the fraction of training data with an MD greater than or equal to the one for the given sample, assuming normally distributed data. The smaller the p-value, the less trustworthy the prediciton. For highly non-normal X properties (e.g., fingerprints), the MD p-value is wildly inaccurate.

Model Applicability

Unknown features are fingerprint features in the query molecule, but not found or appearing too infrequently in the training set.

1. All properties and OPS components are within expected ranges.
2. Unknown ECFP_2 feature: 2131425032: [*]\C=C(\C=[*])/C(=[*])[*]
3. Unknown ECFP_2 feature: 1000552169: [*]\C=C\1/S[*][*]C1=[*]
4. Unknown FCFP_6 feature: 16: [*]:[cH]:[*]
5. Unknown FCFP_6 feature: 1618154665: [*]:[cH]:[cH]:[cH]:[*]
6. Unknown FCFP_6 feature: 451371068: [*]C(=C[c](:[*]):[*])[*]

Feature Contribution

Top features for positive contribution

Fingerprint	Bit/Smiles	Feature Structure	Score

ECFP_6	642810091	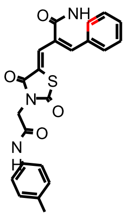 [*][c](:[*]):[*]	0.281
ECFP_6	-1897341097	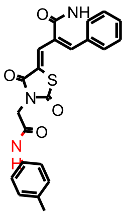 [*]N[*]	0.216
FCFP_6	436915834	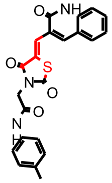 [*]\C=C\1/S[*][*]C1=[*]	0.184
**Top Features for negative contribution**			
**Fingerprint**	**Bit/Smiles**	**Feature Structure**	**Score**
ECFP_6	2106656448	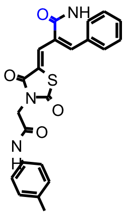 [*]C(=O)[*]	-0.352
FCFP_6	566058135	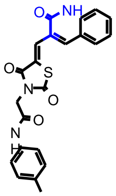 [*]NC(=O)C(=[*])[*]	-0.216

ECFP_6	734603939	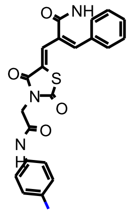 [*]C	-0.201

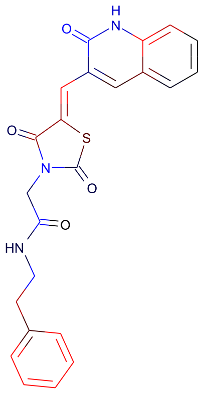

$C_{23}H_{19}N_3O_4S$

Molecular Weight: 433.47965

ALogP: 2.495

Rotatable Bonds: 6

Acceptors: 5

Donors: 2

Structural Similar Compounds

Name	PIRETANIDE	CARBAMIC ACID; [1-[(5-CYANOPENTYL)CARBAMOYL]BENZIMIDAZOL-2-YL]-; METHYL ESTER	OCHRATOXIN A
Structure	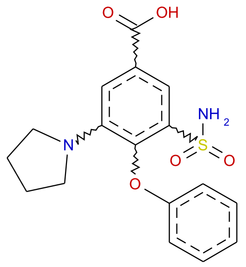	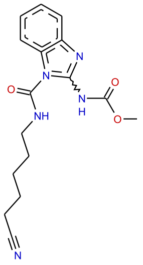	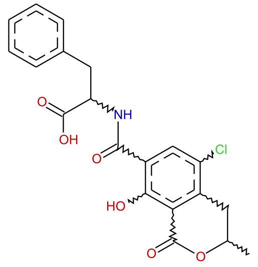
Actual Endpoint (-log C)	1.811	2.12	4.305
Predicted Endpoint (-log C)	1.83976	1.78415	3.03558
Distance	0.564	0.579	0.617
Reference	DRFUD4 2;393;77	85ARAE 4;118;76/77	FCTXAV 6;479;68

Model Prediction

Prediction: 0.954

Unit: g/kg_body_weight

Mahalanobis Distance: 25.2

Mahalanobis Distance p-value: 1.84e-032

Mahalanobis Distance: The Mahalanobis distance (MD) is a generalization of the Euclidean distance that accounts for correlations among the X properties. It is calculated as the distance to the center of the training data. The larger the MD, the less trustworthy the prediction.

Mahalanobis Distance p-value: The p-value gives the fraction of training data with an MD greater than or equal to the one for the given sample, assuming normally distributed data. The smaller the p-value, the less trustworthy the prediciton. For highly non-normal X properties (e.g., fingerprints), the MD p-value is wildly inaccurate.

Model Applicability

Unknown features are fingerprint features in the query molecule, but not found or appearing too infrequently in the training set.

1. All properties and OPS components are within expected ranges.
2. Unknown ECFP_2 feature: 2131425032: [*]\C=C(\C=[*])/C(=[*])[*]
3. Unknown ECFP_2 feature: 1000552169: [*]\C=C\1/S[*][*]C1=[*]
4. Unknown FCFP_6 feature: 16: [*]:[cH]:[*]
5. Unknown FCFP_6 feature: 1618154665: [*]:[cH]:[cH]:[cH]:[*]
6. Unknown FCFP_6 feature: 451371068: [*]C(=C[c](:[*]):[*])[*]

Feature Contribution

Top features for positive contribution

Fingerprint	Bit/Smiles	Feature Structure	Score

ECFP_6	642810091	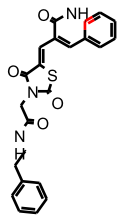 [*][c](:[*]):[*]	0.281
ECFP_6	-1897341097	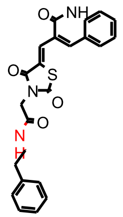 [*]N[*]	0.216
ECFP_6	1571214559	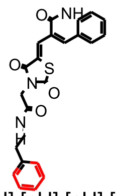 [*]1:[cH]:[cH]:[cH]:[cH]:[cH]:1	0.19
**Top Features for negative contribution**			
**Fingerprint**	**Bit/Smiles**	**Feature Structure**	**Score**
ECFP_6	2106656448	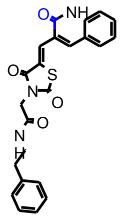 [*]C(=O)[*]	-0.352
ECFP_6	497523368	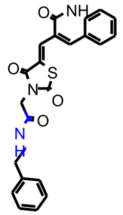 [*]CNC(=[*])[*]	-0.301

FCFP_6	566058135	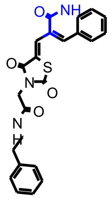<br>[*]NC(=O)C(=[*])[*]	-0.216

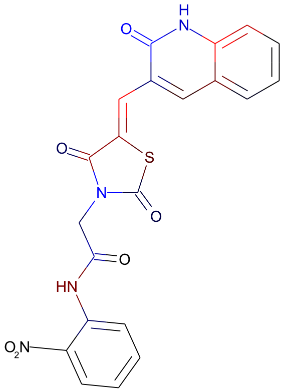

$C_{21}H_{14}N_4O_6S$
Molecular Weight: 450.42405
ALogP: 2.061
Rotatable Bonds: 5
Acceptors: 7
Donors: 2

Structural Similar Compounds

Name	LENAMPICILLIN .HCl (HCl STRIPPED)	PIRETANIDE	BENZOTHIAZOLE; 6-NITRO-2-(p-NITROBENZOYLAMINO)-
Structure	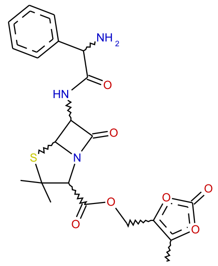	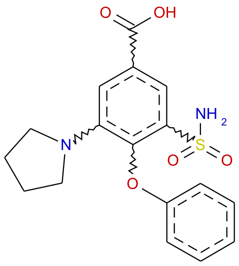	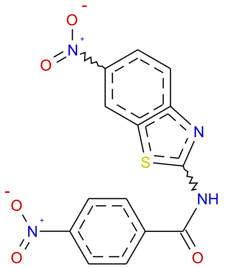
Actual Endpoint (-log C)	1.664	1.811	2.361
Predicted Endpoint (-log C)	2.37593	1.83976	2.96257
Distance	0.699	0.727	0.734
Reference	NKRZAZ 32(Suppl	DRFUD4 2;393;77	JPETAB 90;260;47

Model Prediction
Prediction: 0.767
Unit: g/kg_body_weight
Mahalanobis Distance: 25.8
Mahalanobis Distance p-value: 6.3e-036

Mahalanobis Distance: The Mahalanobis distance (MD) is a generalization of the Euclidean distance that accounts for correlations among the X properties. It is calculated as the distance to the center of the training data. The larger the MD, the less trustworthy the prediction.
Mahalanobis Distance p-value: The p-value gives the fraction of training data with an MD greater than or equal to the one for the given sample, assuming normally distributed data. The smaller the p-value, the less trustworthy the prediciton. For highly non-normal X properties (e.g., fingerprints), the MD p-value is wildly inaccurate.

Model Applicability
Unknown features are fingerprint features in the query molecule, but not found or appearing too infrequently in the training set.

1. All properties and OPS components are within expected ranges.
2. Unknown ECFP_2 feature: 1043790491: [*][N+](=[*])[*]
3. Unknown ECFP_2 feature: 781519895: [*][O-]
4. Unknown ECFP_2 feature: 2131425032: [*]\C=C(\C=[*])/C(=[*])[*]
5. Unknown ECFP_2 feature: 1000552169: [*]\C=C\1/S[*][*]C1=[*]
6. Unknown ECFP_2 feature: -1956535100: [*][c](:[*]):[c](:[cH]:[*])[N+](=[*])[*]
7. Unknown ECFP_2 feature: -215026467: [*]:[c](:[*])[N+](=O)[O-]
8. Unknown ECFP_2 feature: 2104376220: [*][N+](=O)[*]
9. Unknown ECFP_2 feature: -659271057: [*][N+](=[*])[O-]
10. Unknown FCFP_6 feature: 16: [*]:[cH]:[*]
11. Unknown FCFP_6 feature: 8: [*][N+](=[*])[*]
12. Unknown FCFP_6 feature: 5: [*][O-]
13. Unknown FCFP_6 feature: 1618154665: [*]:[cH]:[cH]:[cH]:[*]
14. Unknown FCFP_6 feature: 451371068: [*]C(=C[c](:[*]):[*])[*]
15. Unknown FCFP_6 feature: -828984032: [*][c](:[*]):[c](:[cH]:[*])[N+](=[*])[*]
16. Unknown FCFP_6 feature: -1338588315: [*]:[c](:[*])[N+](=O)[O-]
17. Unknown FCFP_6 feature: 1872392852: [*][N+](=O)[*]
18. Unknown FCFP_6 feature: 260476081: [*][N+](=[*])[O-]

Feature Contribution

Top features for positive contribution

Fingerprint	Bit/Smiles	Feature Structure	Score
ECFP_6	642810091	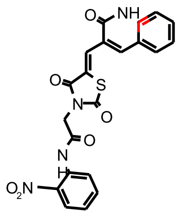 [*][c](:[*]):[*]	0.281
ECFP_6	-1897341097	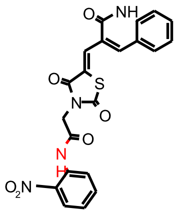 [*]N[*]	0.216
FCFP_6	436915834	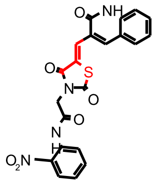 [*]\C=C\1/S[*][*]C1=[*]	0.184

Top Features for negative contribution

Fingerprint	Bit/Smiles	Feature Structure	Score
ECFP_6	2106656448	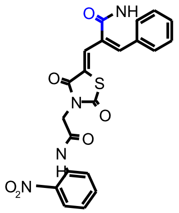 [*]C(=O)[*]	-0.352

FCFP_6	566058135	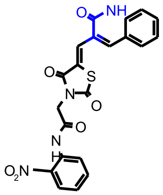 [*]NC(=O)C(=[*])[*]	-0.216
ECFP_6	670515721	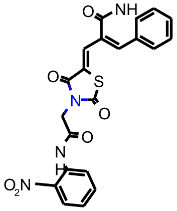 [*]N([*])[*]	-0.108

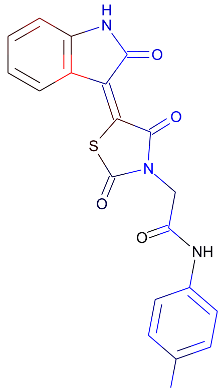

$C_{20}H_{15}N_3O_4S$
Molecular Weight: 393.4158
ALogP: 2.185
Rotatable Bonds: 3
Acceptors: 5
Donors: 2

Structural Similar Compounds

Name	PIRETANIDE	PIROXICAM	1H-1;4-BENZODIAZEPINE-1-CARBOXAMIDE; 2;3-DIHYDRO-N-METHYL-7-NITRO-2-OXO-5-PHENYL-
Structure	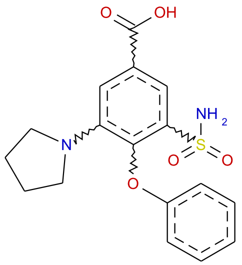	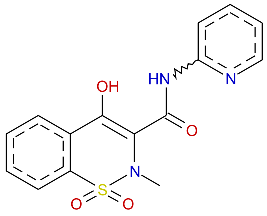	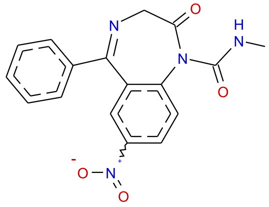
Actual Endpoint (-log C)	1.811	3.186	2.171
Predicted Endpoint (-log C)	1.83976	2.63418	2.64752
Distance	0.550	0.581	0.581
Reference	DRFUD4 2;393;77	ARZNAD 28;1714;78	TAKHAA 29;153;70

Model Prediction

Prediction: 4.07
Unit: g/kg_body_weight
Mahalanobis Distance: 21.7
Mahalanobis Distance p-value: 5.71e-015

Mahalanobis Distance: The Mahalanobis distance (MD) is a generalization of the Euclidean distance that accounts for correlations among the X properties. It is calculated as the distance to the center of the training data. The larger the MD, the less trustworthy the prediction.
Mahalanobis Distance p-value: The p-value gives the fraction of training data with an MD greater than or equal to the one for the given sample, assuming normally distributed data. The smaller the p-value, the less trustworthy the prediciton. For highly non-normal X properties (e.g., fingerprints), the MD p-value is wildly inaccurate.

Model Applicability

Unknown features are fingerprint features in the query molecule, but not found or appearing too infrequently in the training set.

1. All properties and OPS components are within expected ranges.
2. Unknown FCFP_6 feature: 16: [*]:[cH]:[*]
3. Unknown FCFP_6 feature: -1678275541: [*]C(=C1C(=[*])[*][*]:[c]1:[*])[*]
4. Unknown FCFP_6 feature: 1618154665: [*]:[cH]:[cH]:[cH]:[*]

Feature Contribution

Top features for positive contribution

Fingerprint	Bit/Smiles	Feature Structure	Score
ECFP_6	642810091	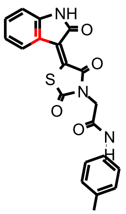 [*][c](:[*]):[*]	0.281

ECFP_6	-1897341097	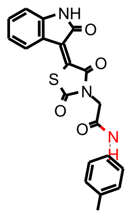 [*]N[*]	0.216
FCFP_6	436915834	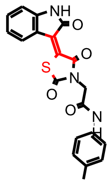 [*]\C=C\1/S[*][*]C1=[*]	0.184
**Top Features for negative contribution**			
**Fingerprint**	**Bit/Smiles**	**Feature Structure**	**Score**
ECFP_6	2106656448	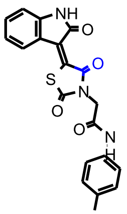 [*]C(=O)[*]	-0.352
FCFP_6	566058135	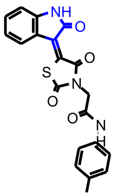 [*]NC(=O)C(=[*])[*]	-0.216
ECFP_6	734603939	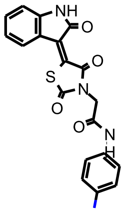 [*]C	-0.201

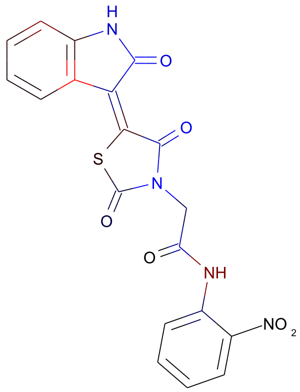

$C_{19}H_{12}N_4O_6S$
Molecular Weight: 424.38678
ALogP: 1.593
Rotatable Bonds: 4
Acceptors: 7
Donors: 2

Model Prediction

Prediction: 1.38
Unit: g/kg_body_weight
Mahalanobis Distance: 23.7
Mahalanobis Distance p-value: 2.56e-024

Mahalanobis Distance: The Mahalanobis distance (MD) is a generalization of the Euclidean distance that accounts for correlations among the X properties. It is calculated as the distance to the center of the training data. The larger the MD, the less trustworthy the prediction.
Mahalanobis Distance p-value: The p-value gives the fraction of training data with an MD greater than or equal to the one for the given sample, assuming normally distributed data. The smaller the p-value, the less trustworthy the prediciton. For highly non-normal X properties (e.g., fingerprints), the MD p-value is wildly inaccurate.

Structural Similar Compounds

Name	LENAMPICILLIN .HCl (HCl STRIPPED)	AZOSEMIDE	SUDOXICAM
Structure	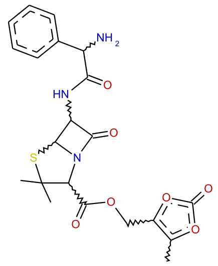	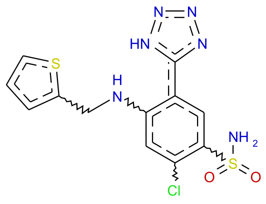	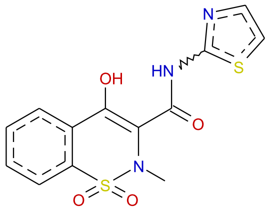
Actual Endpoint (-log C)	1.664	2.163	3.395
Predicted Endpoint (-log C)	2.37593	2.21052	2.46488
Distance	0.708	0.715	0.715
Reference	NKRZAZ 32(Suppl	IYKEDH 18;666;87	ARZNAD 28;1714;78

Model Applicability

Unknown features are fingerprint features in the query molecule, but not found or appearing too infrequently in the training set.

1. All properties and OPS components are within expected ranges.
2. Unknown ECFP_2 feature: 1043790491: [*][N+](=[*])[*]
3. Unknown ECFP_2 feature: 781519895: [*][O-]
4. Unknown ECFP_2 feature: -1956535100: [*][c](:[*]):[c](:[cH]:[*])[N+](=[*])[*]
5. Unknown ECFP_2 feature: -215026467: [*]:[c](:[*])[N+](=O)[O-]
6. Unknown ECFP_2 feature: 2104376220: [*][N+](=O)[*]
7. Unknown ECFP_2 feature: -659271057: [*][N+](=[*])[O-]
8. Unknown FCFP_6 feature: 16: [*]:[cH]:[*]
9. Unknown FCFP_6 feature: 8: [*][N+](=[*])[*]
10. Unknown FCFP_6 feature: 5: [*][O-]
11. Unknown FCFP_6 feature: -1678275541: [*]C(=C1C(=[*])[*][*]:[c]1:[*])[*]
12. Unknown FCFP_6 feature: 1618154665: [*]:[cH]:[cH]:[cH]:[*]
13. Unknown FCFP_6 feature: -828984032: [*][c](:[*]):[c](:[cH]:[*])[N+](=[*])[*]
14. Unknown FCFP_6 feature: -1338588315: [*]:[c](:[*])[N+](=O)[O-]
15. Unknown FCFP_6 feature: 1872392852: [*][N+](=O)[*]
16. Unknown FCFP_6 feature: 260476081: [*][N+](=[*])[O-]

Feature Contribution

Top features for positive contribution

Fingerprint	Bit/Smiles	Feature Structure	Score
ECFP_6	642810091	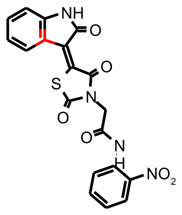<br>[*][c](:[*]):[*]	0.281
ECFP_6	-1897341097	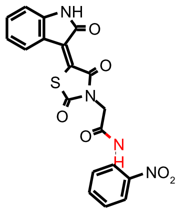<br>[*]N[*]	0.216
FCFP_6	436915834	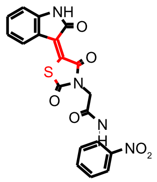<br>[*]\C=C\1/S[*][*]C1=[*]	0.184

Top Features for negative contribution

Fingerprint	Bit/Smiles	Feature Structure	Score
ECFP_6	2106656448	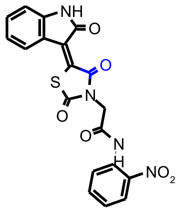<br>[*]C(=O)[*]	-0.352

FCFP_6	566058135	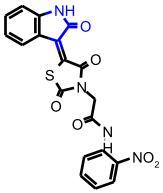 [*]NC(=O)C(=[*])[*]	-0.216
ECFP_6	670515721	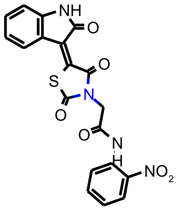 [*]N([*])[*]	-0.108

Sorafenib

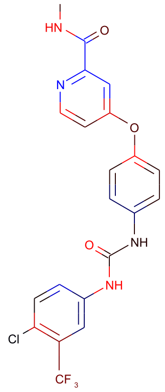

$C_{21}H_{16}ClF_3N_4O_3$
Molecular Weight: 464.82494
ALogP: 4.175
Rotatable Bonds: 6
Acceptors: 4
Donors: 3

Structural Similar Compounds

Name	FLUBENDAZOLE	PHOSPHORAMIDOTHIOIC ACID; ACETIMIDOYL-; O;O-bis-(p-CHLOROPHENYL)ESTER	BEZAFIBRATE
Structure	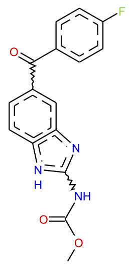	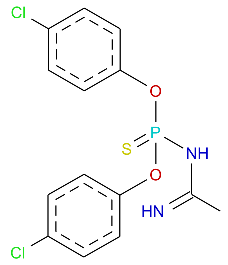	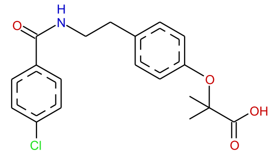
Actual Endpoint (-log C)	2.088	5.006	1.946
Predicted Endpoint (-log C)	2.69288	3.23989	2.54395
Distance	0.697	0.703	0.721
Reference	YRTMA6 9;11;78	FMCHA2 -;C149;89	ARZNAD 30;2023;80

Model Prediction

Prediction: 0.823
Unit: g/kg_body_weight
Mahalanobis Distance: 21
Mahalanobis Distance p-value: 1.93e-012

Mahalanobis Distance: The Mahalanobis distance (MD) is a generalization of the Euclidean distance that accounts for correlations among the X properties. It is calculated as the distance to the center of the training data. The larger the MD, the less trustworthy the prediction.
Mahalanobis Distance p-value: The p-value gives the fraction of training data with an MD greater than or equal to the one for the given sample, assuming normally distributed data. The smaller the p-value, the less trustworthy the prediciton. For highly non-normal X properties (e.g., fingerprints), the MD p-value is wildly inaccurate.

Model Applicability

Unknown features are fingerprint features in the query molecule, but not found or appearing too infrequently in the training set.

1. All properties and OPS components are within expected ranges.
2. Unknown FCFP_6 feature: 16: [*]:[cH]:[*]
3. Unknown FCFP_6 feature: 1618154665: [*]:[cH]:[cH]:[cH]:[*]
4. Unknown FCFP_6 feature: 1747237384: [*][c](:[*]):n:[cH]:[*]
5. Unknown FCFP_6 feature: 136686699: [*]NC
6. Unknown FCFP_6 feature: 71476542: [*]:[c](:[*])Cl

Feature Contribution

Top features for positive contribution

Fingerprint	Bit/Smiles	Feature Structure	Score

FCFP_6	71953198	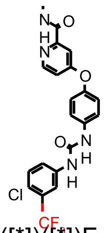 [*]C([*])([*])F	0.392
ECFP_6	-1046436026	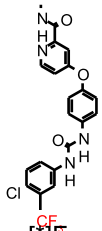 [*]F	0.349
ECFP_6	642810091	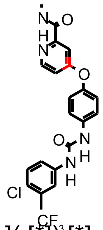 [*][c](:[*]):[*]	0.281

Top Features for negative contribution

**Fingerprint**	**Bit/Smiles**	**Feature Structure**	**Score**
ECFP_6	226796801	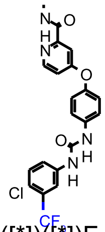 [*]C([*])([*])F	-0.32
ECFP_6	-817402818	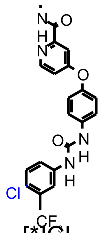 [*]Cl	-0.263

ECFP_6	-176455838	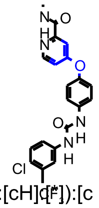<br>[*]O[c](:[cH]c[*]):[cH]:[*]	-0.257

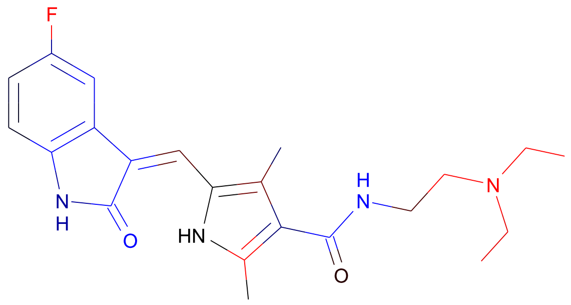

$C_{22}H_{27}FN_4O_2$

Molecular Weight: 398.47378

ALogP: 2.997

Rotatable Bonds: 7

Acceptors: 3

Donors: 3

Model Prediction

Prediction: 2.88

Unit: g/kg_body_weight

Mahalanobis Distance: 23.7

Mahalanobis Distance p-value: 3.38e-024

Mahalanobis Distance: The Mahalanobis distance (MD) is a generalization of the Euclidean distance that accounts for correlations among the X properties. It is calculated as the distance to the center of the training data. The larger the MD, the less trustworthy the prediction.
Mahalanobis Distance p-value: The p-value gives the fraction of training data with an MD greater than or equal to the one for the given sample, assuming normally distributed data. The smaller the p-value, the less trustworthy the prediciton. For highly non-normal X properties (e.g., fingerprints), the MD p-value is wildly inaccurate.

Structural Similar Compounds

Name	METHYLERGONOVINE	CLEBOPRIDE; MALATE SALT (MALATE STRIPPED)	DOMPERIDONE
Structure	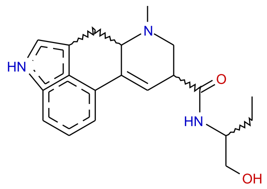	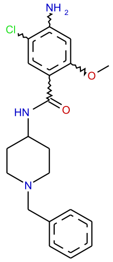	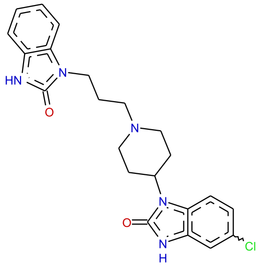
Actual Endpoint (-log C)	3.562	2.168	1.91
Predicted Endpoint (-log C)	2.94539	2.89926	2.49077
Distance	0.567	0.599	0.601
Reference	27ZIAQ -;163;73	OYYAA2 25;803;83	YACHDS 8;3991;80

Model Applicability

Unknown features are fingerprint features in the query molecule, but not found or appearing too infrequently in the training set.

1. All properties and OPS components are within expected ranges.
2. Unknown ECFP_2 feature: 980271847: [*][c]1:[*]:[*]:[nH]:[c]:1C=[*]
3. Unknown FCFP_6 feature: 19: [*]:[nH]:[*]
4. Unknown FCFP_6 feature: 16: [*]:[cH]:[*]
5. Unknown FCFP_6 feature: 2005402822: [*][c]1:[*]:[*]:[c]([*]):[nH]:1
6. Unknown FCFP_6 feature: 203707511: [*][c]1:[*]:[*]:[nH]:[c]:1C
7. Unknown FCFP_6 feature: 451371068: [*]C(=C[c](:[*]):[*])[*]
8. Unknown FCFP_6 feature: -1678275541: [*]C(=C1C(=[*])[*][*]:[c]1:[*])[*]
9. Unknown FCFP_6 feature: 1618154665: [*]:[cH]:[cH]:[cH]:[*]
10. Unknown FCFP_6 feature: 71476542: [*]:[c](:[*])Cl

Feature Contribution

Top features for positive contribution			
**Fingerprint**	**Bit/Smiles**	**Feature Structure**	**Score**

ECFP_6	-1046436026	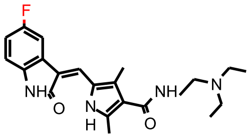<br>[*]F	0.349
ECFP_6	642810091	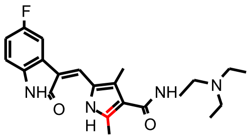<br>[*][c](:[*]):[*]	0.281
FCFP_6	1852108031	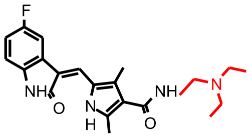<br>[*]CCN(CC)CC	0.226

Top Features for negative contribution

Fingerprint	Bit/Smiles	Feature Structure	Score
ECFP_6	2106656448	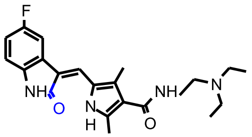<br>[*]C(=O)[*]	-0.352
ECFP_6	497523368	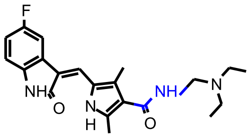<br>[*]CNC(=[*])[*]	-0.301

FCFP_6	566058135	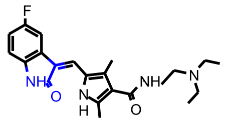 [*]NC(=O)C(=[*])[*]	-0.216
